# Carbon-Substituted Amines of the Cobalt Bis(dicarbollide) Ion: Stereochemistry and Acid–Base Properties

**DOI:** 10.1021/acs.inorgchem.4c03257

**Published:** 2024-10-11

**Authors:** Ece Zeynep Tüzün, Lucia Pazderová, Dmytro Bavol, Miroslava Litecká, Drahomír Hnyk, Zdeňka Růžičková, Ondřej Horáček, Radim Kučera, Bohumír Grűner

**Affiliations:** † Institute of Inorganic Chemistry of the Czech Academy of Sciences, 25068 Řež, Czech Republic; ‡ Dpt. of Inorganic Chemistry, Faculty of Science, Charles University, Hlavova 2030/8, 128 43 Prague, Czech Republic; § Dpt. of General and Inorganic Chemistry, Faculty of Chemical Technology, University of Pardubice, Studentská 95, 53210 Pardubice, Czech Republic; ∥ Faculty of Pharmacy, Charles University, Akademika Heyrovského 1203, 500 05 Hradec Králové, Czech Republic

## Abstract

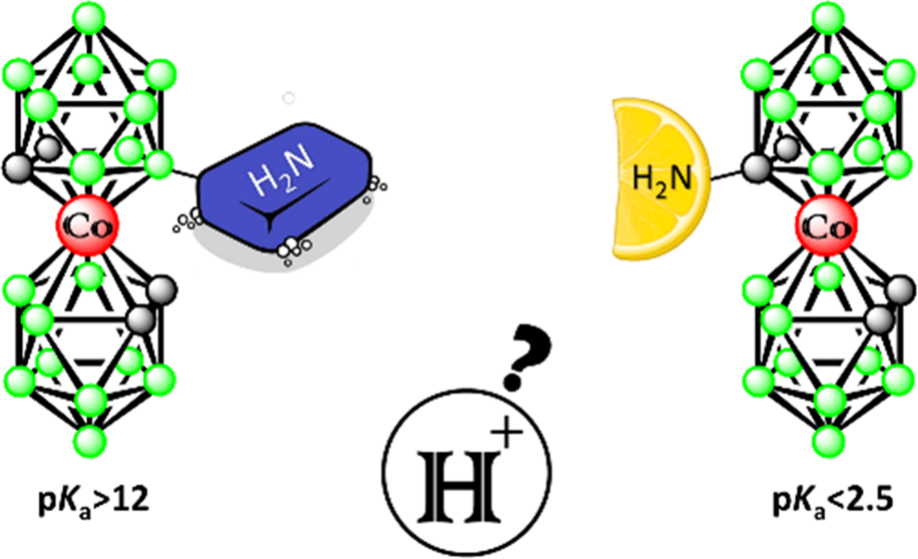

Organic amines are found to be abundant in natural living systems. They also constitute an inestimable family of building blocks available in drug design. Considering the man-made cluster [(1,2-C_2_B_9_H_11_)_2_-3,3′-Co(III)]^−^ ion (**1-**) and its application as an emerging unconventional pharmacophore, the availability of the corresponding amines has been limited and those with amino groups attached directly to carbon atoms have remained unknown. This paper describes the synthesis of compounds containing one or two primary amino groups attached to the carbon atoms of the cobaltacarborane cage that are accessible via the reduction of newly synthesized azides or via the Curtius rearrangement of the corresponding acyl azide. This substitution represents the first members of the series of azides and primary amines with functional groups bound directly to the carbon atoms of the cage. As expected, the absence of the linker along with the presence of the bulky anionic polyhedral ion leads to a significant alteration of the chemical and physicochemical properties. On a broader series of amines of the ion **1**^**–**^ we have thus observed significant differences in the acidity of the amino groups, depending on whether these are attached to the carbon or boron atoms of the cage, or the C-substituted amines contain an aliphatic linker of variable length. The compounds are relevant for potential use as cobalt bis(dicarbollide) structural blocks in medicinal chemistry and material science. Our study includes single-crystal X-ray diffraction (XRD) structures of both amines and a discussion of their stereochemical and structural features.

## Abstract 2

Synthesis of carbon-substituted derivatives of the cobalt bis(dicarbollide) ion containing one or two amino groups has been reported using two independent methods. The measures on the acid−base properties of NH_2_ groups are considerably different from those of previously known boron-substituted congeners.

## Introduction

Organic amines^1^ represent ubiquitous basic construction units present in nature that are involved in a wide variety of physiological functions in living systems including metabolic, enzymatic, and regulatory processes. They show a wide range of basicity and nucleophilic properties due to the lone electron pair on the nitrogen atom and participate in the formation of hydrogen bonds. Because of their functions in living nature, amines have earned a privileged role in drug design and their synthesis has become of high concern and interest.^2^ Boron clusters such as *closo*-borate ions, dicarba-*closo*-dodecaboranes and icosahedral metallacarboranes are man-made species based on unusual three-center two-electron (3c–2e^–^) bonding^3^ that are often considered as unconventional isosteres of aromatic rings or polycyclic hydrocarbons in drug design.^4−7^ These species display distinctive properties such as 3D-aromaticity,^8^ a slightly larger size than a rotated phenyl ring, however, they exhibit significantly higher steric requirements, high chemical and thermal stability, unusual types of interactions such as the formation of dihydrogen bonds^9^ and lipophilic properties that are connected with the hydridic character of the boron–hydrogen bonds present on the surface of the polyhedral cages. Boron cluster anions have their charge delocalized over a large surface area, show amphiphilic properties^10^ and behave as superchaotropes in solution.^11,12^

During the last few years, our and other research groups have been involved in the contemporary search for new methods to incorporate boron clusters, and particularly their metal complexes (metallacarboranes), as nontraditional pharmacophores into drug design. Nevertheless, the synthetic tools for the incorporation of the most studied cobalt bis(dicarbollide) anion^13,14^ [(1,2-C_2_B_9_H_11_)_2_-3,3′-Co(III)]^−^ (**1**^**–**^) into functional molecules are still considerably off the pace when compared to the methods available in organic or organometallic chemistry. This is related to the quite limited selection of the substitution sites that can be addressed, as well as to the possibilities of tuning the distance of the group from the cage and physicochemical properties. The currently available methods offer efficient boron B(8) substitution of the **1**^**–**^ ion using the cleavage of a cyclic ether ring moiety with a large scope of nucleophilic reagents^15,16^ or via the introduction of ammonium^17^ or hydroxy groups^18^ into this boron site. This chemistry has been used in the design of compounds for biomedical applications such as specific inhibitors of HIV-protease,^19^ carbonic anhydrase (CA-IX),^20,21^ cytotoxic compounds,^22^ compounds with antimicrobial and antiparasitic properties,^6,23^ boron carriers in BNCT,^24^ cell membrane penetrating vesicles and carriers,^12,25^ redox labels for nucleosides and nucleotides,^26^ molecular imaging,^27^ and several other medicinal applications that are subject of several recent reviews and book chapters.^7,28^

More recently, methods for C-substitutions on the cobalt bis(dicarbollide) ion with groups such as carboxyalkyl,^29^ hydroxyalkyl,^30^ mesyl esters, and alkylamines,^20,29^ which can be easily further modified, have been developed as part of the structural optimization of metallacarborane inhibitors of the CA-IX enzyme and this chemistry has also been the subject of a recent review article.^14^ These methods consist of the lithiation of C–H vertices with RLi under carefully controlled low-temperature conditions and their subsequent reactions with electrophiles providing carbon–silicon,^31^ carbon–phosphorus,^32^ or carbon–carbon bonds.^30^ However, the introduction of a carbon–nitrogen bond seems to present more peculiarities, and no direct substitution has been reported yet.

Herein, we present the synthesis of new primary amino derivatives of anion **1**^**–**^ accessible using two independent synthetic routes. The first one proceeds via the reaction of the lithiated cage with toluenesulfonyl (tosyl) azide and provides access to carbon-substituted mono- and diazides, which could be reduced to the corresponding amines with the amino group on C(1) or C(1,1′) atoms. The second method producing only the monosubstituted species, consists of Curtius rearrangement of acyl azide prepared from a C(1) substituted carboxylic derivative. This paper also reports the distinctive effect of the cage on several observed irregular pathways as well as the chemical properties of the azido group(s) attached directly to the carbon atoms that have served as precursors to amines. Moreover, it compares and discusses the differences between the experimentally determined p*K*_a_ values and the calculated gas-phase proton acidities observed within a series of carbon-substituted amines, carbon-substituted alkylamines, and boron-substituted congeners. The azide and amine substitution provides a new type of functional group at the cage applicable as a platform in the medicinal chemistry of boron. Based on p*K*_a_ values and single-crystal X-ray structures, we discuss the fundamental characteristics arising from azide and amine substitution on carbon atom(s), i.e., stability, the ability to undergo typical organic reactions, the presence of hydrochlorides in the crystals, the stereochemistry of disubstituted derivatives, and the chirality of all new carbon-substituted azides and amines.

## Experimental Section

### Materials

The starting cesium salt of the cobalt bis(dicarbollide) anion was purchased from Katchem Ltd., Czech Republic. The starting carboxylic acid and mesyl esters were prepared according to previously described procedures. Ethylene glycol dimethyl ether (DME) was distilled from sodium diphenyl ketyl. Dimethylformamide (DMF, dry, Aldrich) was used as purchased and maintained under argon. Dried acetonitrile (molecular sieve 4 Å, Fluka) was used. The other chemical reagents and solvents were purchased from Aldrich, Merck Lachema a.s., and Penta Ltd., Czech Republic, and used without purification. Analytical thin-layer chromatography (TLC) was carried out on Silufol TLC plates (silica gel, Lachema, Czech Republic) in a CH_2_Cl_2_/CH_3_CN: 3/1 mixture. Liquid chromatography (LC) was performed on high-purity silica gel (Merck grade, Type 7754, 70-230 mesh, 60 Å) or a C18-modified silica gel support (Lichloprep RP18, Merck) in reverse phase mode in aqueous MeOH (typically 50%). All reactions were performed using standard Schlenk vacuum-inert gas techniques, although purifications by column chromatography and crystallization were carried out under an ambient atmosphere. All the newly synthesized compounds were characterized by the combination of ^11^B, ^1^H, and ^13^C NMR spectral data, high-resolution mass spectrometry (HRMS, four decimal place resolution), and IP reversed phase high-performance liquid chromatography (RP-HPLC) analysis.

***Caution!*** Appropriate personal protective equipment (PPE) including eye protection, suitable gloves, and a lab coat must be worn when operating a Schlenk line. Risk assessments and appropriate training must be completed before operating a Schlenk line. Also, extreme care should be taken both in the handling of the cryogenic liquid nitrogen and its use in the Schlenk line trap to avoid the condensation of oxygen from air. Wear cold insulating gloves and face shields during handling.

### Instrumental and Computational Techniques

#### NMR Spectroscopy

Nuclear magnetic resonance spectroscopy measurements were carried out on a JEOL 600 MHz spectrometer. The spectra of all compounds were measured after dissolution in deuterated acetone or methanol, unless otherwise stated. ^11^B NMR (193 MHz) chemical shifts are given in ppm to high-frequency (low field) to F_3_B·OEt_2_ as the external reference. Residual solvent ^1^H resonances were used as the internal secondary standards. The NMR data are presented in the text as follows: ^11^B NMR: ^11^B chemical shifts δ (ppm), multiplicity. ^1^H NMR (600 MHz) and ^13^C (151 MHz): chemical shifts δ are given in ppm relative to the standard Me_4_Si (0 ppm); coupling constants *J*(H,H) and *J*(B,H) are given in Hz.

#### HRMS

High-resolution mass spectrometry (HRMS) spectra were recorded by an Orbitrap Exploris 120 spectrometer equipped with heated electrospray ionization (HESI) in negative mode using nitrogen (5.0 Messer) as a collision gas. For HESI-MS, solutions of concentration approximately 100 ng·mL^–1^ in acetonitrile were introduced by infusion into the ion source from a syringe. Molecular ions [M]^−^ were detected for all univalent anions as base peaks in the spectra. By comparison, the experimental isotopic distribution in the boron plot of the peaks in the measured spectra corresponded fully to the calculated spectral pattern. The data are presented for the most abundant mass in the boron distribution plot (100%) and for the signal corresponding to the *m*/*z* value. Conditions used for the HESI interface: vaporizer temperature 50 °C; N_2_ (isolated from air in a Genius XE35, Peak Scientific) as a nebulizing sheath gas and auxiliary gas, flow 3.22 and 6.12 L·min^–1^, respectively; an ion spray voltage of 3500 V; a capillary temperature of 280 °C, and a mass range from 100 to 1200.

#### HPLC

A Merck-Hitachi LaChrom Series 7000 HPLC system equipped with a Diode-Array D 7450 detector and an L7250 autoinjector and software was used for monitoring the composition of the reaction mixture and the purity control of the isolated reaction intermediates and the final products. Analytical separations were carried out using a previously reported Ion-Pair RP method with recent updates.^33^ A polymeric Separon RPS column, 5 μm (300 mm × 3 mm I. D.), Tessek Prague in RP mode, and hexylamine acetate 4.5 mmol in 65% aqueous CH_3_CN (pH 5.9) as the mobile phase were utilized for the analyses. The solutes were detected using DAD detection at fixed wavelengths of 235, 280, 285, and 312 nm.

#### Single-Crystal X-ray Diffraction

Data of compounds **4**^**–**^, **5**^**–**^, and **10**^**–**^ to **12**^**–**^ were collected on the Rigaku Synergy S XtaLAB diffractometer equipped with microfocus with Cu radiation (Cu/Kα λ = 1.54184 Å) and a Hybrid Pixel Array Detector (HyPix-6000HE). An Oxford Cryosystems (Cryostream 800) cooling device was used for data collection and the crystals were kept at 100.00(10) K during data collection. CrysAlisPro software^34^ was used for data collection, cell refinement, data reduction, and absorption correction. Data were corrected for absorption effects using empirical absorption correction (spherical harmonics), implemented in a SCALE3 ABSPACK scaling algorithm, and numerical absorption correction based on the Gaussian integration over a multifaceted crystal model.^35^ The structures of compounds **4**^**–**^, **5**^**–**^, and **10**^**–**^ to **12**^**–**^ were solved using the ShelXT^36^ structure solution program using Intrinsic Phasing and refined with the SHELXL^37^ refinement package using least-squares minimization implemented in Olex2.^38^ Anisotropic displacement parameters were refined for all non-H atoms. The hydrogen atoms were calculated to idealized positions or were found in the residual electron density map. For crystallographic data and the structure refinement see Tables S1–S10 in Supporting Information. Molecular graphics for the structures of **4**^**–**^, **5**^**–**^, **6**^**–**^, and **10**^**–**^ to **12**^**–**^ in the Supporting Information (SI) were generated using DIAMOND software (Version 4.6.8).^39^ Selected interatomic distances and angles are given in Figures 2–7 captions, and the details about the particular structures are provided in the Supporting Information. Crystallographic parameters for each compound are given in Tables S1 and S10 and more details about the structures are displayed in Figures S4–S23.

#### Determination of p*K*_a_ Values Using Capillary Electrophoresis

The values of p*K*_a_ were measured by using capillary electrophoresis (CE) and pressure-assisted capillary electrophoresis in positive polarity. Analytical conditions were adapted from the work of Wan,^40^ Geiser,^41^ and Šolínová.^42^ The experiments were carried out with an Agilent 7100 CE (Agilent, Santa Clara, USA) equipped with a DAD detector. The electropherograms were then evaluated at 210 and 290 nm for detection of the electroosmotic flow (DMSO) and the analytes, respectively. The analytes were injected by applying 50 mbar to the inlet vial for 5 s. The applied voltage was 25 kV and the temperature of the capillary cassette was set to 25 °C. The experiments were carried out in the fused-silica capillary (Microsolv Technology, USA) (ID 50 μm, OD 375 μm), with a total length of 51.0 and 42.5 cm to the detector. The newly prepared capillary was sequentially flushed with 1 M NaOH, ultrapure Milli-Q water, and BGE for 30, 10, and 10 min, respectively. The capillary was flushed after switching buffer solutions for 5 min of ultrapure Milli-Q water, 0.1 M NaOH, ultrapure Milli-Q water, and BGE. Between individual analyses, the capillary was flushed with BGE for 2 min. Fresh buffer was used for each run to avoid electrolysis. Analytes were dissolved in a mixture of MeOH-H_2_O–DMSO (1:1:0.1, *v/v/v*) at a concentration of 0.2 mg/mL. The buffers were prepared according to previously published work, with 0.5 pH unit increment for each analyte.^41^ The effective mobilities were calculated from the migration times. The quadruplicate analysis was measured for each pH level. The values of effective mobilities were plotted against the pH values in GraphPad Prism 10.0 (GraphPad Software, San Diego, USA). The p*K*_a_ values were determined as the inflex point of the curve using nonlinear regression.

#### Chiral Separations

Chiral separation of **4**^**–**^ and **5**^**–**^ has been carried out using the chiral column Reprosil chiral-β-CD (250 mm × 4.6 mm; particle size 5 μm) purchased from Dr. Maisch (Ammerbuch-Entringen, Germany). Chromatographic separations were performed on a Shimadzu chromatograph (Kyoto, Japan), composed of a GT–154 solvent degasser, one LC–20AD pump with a quaternary gradient module GT 154, a SIL–20A HT autosampler, a CTO–10AC column oven, an SPD–20A UV detector, CBM–20A system controller, and circular dichroism HPLC detector CD–1595 from Jasco, Japan. The chromatographic data were recorded and analyzed with Clarity software (DataApex, Prague, Czech Republic). The injection volume of all samples dissolved in MeOH (1 mg/mL) was 1 μL. The mobile phase was a mixture of ACN-H_2_O 35:65, *v/v* with 10 mM NaClO_4_ in both solvents; flow rate 1.0 mL/min and column temperature 40 °C.

#### Quantum-Chemical Computations

Stationary points were optimized at the BP86/AE1 level, that is, by employing the exchange and correlation functional of Becke and Perdew, respectively, and all-electron the basis of the augmented Wachter’s basis^i^ on Co and 6-31G* basis on all other elements. Gaussian16 suit of programs^43^ was utilized for all computations. The IAO/IBO method^44^ was used to connect quantitative SCF wave functions to a qualitative chemical picture, the nature of the orbitals naturally emerges. The IBOview program was used.^44^ The corresponding input files for the latter were generated at the B3LYP/def2-TZVP//B3LYP/6-311+G** level using the Turbomole7.3^45^ program package.

### Synthesis

#### [(1-N_3_-1,2-C_2_B_9_H_10_)(1′,2′-C_2_B_9_H_11_)-3,3′-Co]Me_4_N (Me_4_N.**2**) and [(1,1′-N_3_-C_2_B_9_H_10_)_2_-3,3′-Co] (Me_4_N.**3**)

In a typical experiment, cesium salt of cobalt bis(dicarbollide) (Cs**1**, 1.0 g, 3.29 mmol) was dried for 8 h in a vacuum at 195 °C in a Schlenk-type flask and then cooled down to room temperature. The salt was dissolved under an argon atmosphere in 40 mL of freshly distilled 1,2-dimethoxyethane (DME) added through a rubber septum, and the solution was cooled to −82 °C under stirring in a cooling bath containing a mixture of C*O*_2_(*s*) and acetone; then *n*-BuLi (2.5 M in hexane, 3.1 mL, 7.75 mmol) was added dropwise from the syringe over 5 min and the stirring in the cooling bath was continued over 30 min. The bath was then removed and the reaction mixture was left to warm up to room temperature for over 60 min. The dark reaction mixture was cooled once more to −82 °C and then toluenesulfonyl azide (13% solution in toluene, Aldrich, dried over CaH_2_, 11 mL, approximately 7.2 mmol) was added dropwise from a syringe. The content of the flask was stirred for 45 min at −82 °C, then the cooling bath was replaced with another one containing only C*O*_2_(*s*) and the reaction mixture was left to warm up spontaneously to room temperature over 12 h. The reaction was quenched with the careful addition of H_2_O from a syringe (40 mL), and the organic solvent was then distilled off by using a rotary evaporator. The dark coloration persisted even after quenching. The bath temperature was maintained below 35 °C during the evaporation. The mixture of the products was extracted into diethyl ether (6 × 25 mL), water (10 mL) was added to the combined ether fractions, and the organic solvent was removed in a vacuum. The darkly colored, green-black aqueous phase was discarded. The small volume of MeOH was added for dissolution of a dark orange *semi*-solid, and an excess of an aqueous solution of Me_4_NCl was added to precipitate the crude product. The precipitate was left to settle down for 10 min, and then the solid was rapidly filtered and dried for 10 min in vacuum. The separation of compounds **2**^**–**^ and **3**^**–**^ was performed using flash chromatography on a Bchi 120 g C18 reverse phase column starting with 50% MeOH and increasing the methanol content to 70%; flow rate 20 mL/min. The fractions containing the respective products **2**^**–**^ and **3**^–^ were combined, and MeOH was quickly evaporated. Then the products were precipitated again with Me_4_NCl, washed with water (3 × 10 mL), quickly filtered, and dried in a vacuum. Yield of Me_4_N**2**: 555 mg, 58%, bright-yellow solid; HRMS (ESI^–^) *m*/*z* 368.2749 (M^–^, 10%), 365.2857 (100%), calcd. 368.2756 (M^–^, 10%), and 365.2859 (100%). ^1^H NMR (600 MHz, Methanol-D_4_) δ 4.85 (3H, s), 4.09 (1H, s, C–H_carborane_), 3.65 (1H, s, C–H_carborane_), 3.53 (s, 1H, C–H_carborane_), 3.17 (12H, s, Me_4_N^+^). ^11^B NMR (193 MHz, Methanol-D_4_) δ 5.29 (1B, d, *J* = 168 Hz, B8′), 4.18 (1B, d, *J* = 174 Hz, B8), 0.80 (1B, d, *J* = 145 Hz, B10′), −1.92 (1B, d, *J* = 147 Hz, B10), −5.85 (4B, d, *J* = 147 Hz), (B9,9′,12,12′), −7.12 (2B, d, *J* = 158 Hz, B4′,7′), −8.48 (1B, d, *J* = 147 Hz, B7), −10.33 (1B, d, *J* = 145 Hz, B4), −18.48 (4B, m, B5,5′,11,11′), −20.46 (1B, d, *J* = 172 Hz, B6′), −23.60 (1B, d, *J* = 170 Hz, B6). ^13^C {^1^H} NMR (151 MHz, Methanol-D_4_) δ 86.03 (1C, C_carborane_), 56.30 (1C, CH_carborane_), 54.59 (4C, Me_4_N^+^), 53.99, 53.82 (2C, CH_carborane_). The compound gradually acquires gray-green color upon standing at ambient temperature or even when stored as solid at −33 °C in a refrigerator; a better stability was observed for rapidly frozen concentrated aqueous solutions, which were stored at −33 °C.

##### Yield of Me_4_N**3**

325 mg, 31%, dark orange solid; HRMS *m*/*z* (ESI^–^) 409.2769 (M^–^, 9%), 406.2878 (100%), calcd. 409.2772 (M^–^) and 406.2873 (100%). ^1^H NMR (600 MHz, Methanol-D_4_) δ 4.88 (HDO), 3.80 (2H, s, CH_carborane_), 3.16 (1H, s, Cage-CH, d, *J* = 0.4 Hz), 2.87 (12C, s, Me_4_N^+^); δ B–H from ^1^H{^11^B}the -(from 2D-COSY NMR): δ 3.61 (2H, s, B(8,8′)-H), 3.11 (2H, s, B(12,12′)-H), 2.71(2H, s, B(10,10′)-H), 2.52 (2H, s, B(4,4′)-H), 2.04 (2H, s, B(7,7′)-H), 1.90 (2H, s, B(6,6′)-H), 1.62 (4H, br s, B(9,9′,5,5′)-H), 1.22 (2H, B(11,11′)-H); ^11^B NMR (193 MHz, Methanol-D_4_) δ 5.33 (2B, d, *J* = 149 Hz, B8,8′), −1.72 (2B, d, *J* = 143 Hz, B10,10′), −5.85 (2B, d, *J* = 112 Hz, B4,4′), −6.49 (2B, d, *J* = 135 Hz, B7,7′), −7.62 (2B, d, *J* = 164 Hz, B9,9′), −11.23 (2B, d, *J* = 143.8 Hz, B12,12′), −16.98 (2B, d, *J* = 164 Hz, B5,5′), −18.28 (2B, d, *J* = 149 Hz, B11,11′), −18.70 (2B, d, *J* = 102.3 Hz, B6,6′); ^13^C {^1^H} NMR (151 MHz, Methanol-D_4_) δ 82.91 (2C, C_carborane_), 55.46 (4C, Me_4_N^+^), 54.57 (2C, CH_carborane_), 44.16. The stability of this compound is even slightly lower than that of the monosubstituted Me_4_N**2**. Optimal conditions for longer storage (month) comprise freezing in ice at low temperatures below −33 °C; however, even then a slow decomposition characterized by changing the yellow or orange color to dark and the presence of *nido*-species in the sample occurs (for more details on decomposition see the text and Figures S1–S3 in the Supporting Information).

***Caution!*** This procedure contains the use of a millimolar concentration of highly flammable chemical *n*-BuLi (*n*-butyllithium). *n*-BuLi must be kept away from heat, hot surfaces, sparks, open flames, and other ignition sources. Handle and store contents under inert gas. Protect from moisture. Wear protective gloves/protective clothing/eye protection/face protection. The azides **2**^**–**^ and **3**^**–**^ are closely related to organic azides with known toxicity issues. In the solid state, they may also be potentially heat- and shock-sensitive, even though we did not observe such a tendency during our handling. However, it is recommended to perform any handling in a well-ventilated hood and wear a protective shield or laboratory goggles and latex gloves.

#### Synthesis of Amines [(1-NH_2_-1,2-C_2_B_9_H_10_)(1′,2′-C_2_B_9_H_11_)-3,3′-Co]^−^ (**4**^–^) and [(1-NH_2_–1,2-C_2_B_9_H_10_)_2_-C_2_B_9_H_11_)-3,3′-Co]^−^ (**5**^–^) via Reduction of the Corresponding Azides **2**^–^ and **3**^–^

##### Amine **4**^–^

The Me_4_N**2** (400 mg, 0.68 mmol) was dissolved in 50% aqueous MeOH (25 mL) in a 100 mL open flask with a wide neck equipped with a stirring bar. The flask was placed into a water bath, and solid CoCl_2_·6H_2_O (50 mg, 2.1 mmol) was added under vigorous stirring followed up with the addition of solid NaBH_4_ (A.R.C., Amsterdam, The Netherlands, 500 mg, 13 mmol). The reaction mixture turned dark immediately and the evolution of hydrogen gas started and ceased within approximately 30 min. The stirring was continued for 2 h when the analysis by HPLC and MS analysis showed almost complete conversion to the amine **4**^**–**^. Methanol was evaporated in a vacuum, the aqueous solution was carefully acidified with diluted HCl (1 M, 10 mL), the amine was extracted into Et_2_O (4 × 20 mL), water (10 mL) was added to the combined ether extracts, and the organic solvent was evaporated in a vacuum with no heating of the flask. Then MeOH and diluted HCl (3 M, 3 mL) were repeatedly added (4 × 20 and 5 mL) to the residue and MeOH was evaporated in a vacuum (bath temperature 35 °C). The crude product was precipitated with an excess of aqueous Me_4_NCl, washed with water (3 × 10 mL), filtered, and dried. Final purification was performed by flash chromatography on Bűchi 80 g RP C18 column using 50% MeOH; flow rate 10 mL/min. MeOH was evaporated from the fraction containing the product, along with part of water, the aqueous solution was acidified with a few drops of 3 M HCl, and the compound was precipitated with Me_4_NCl. Crystals for X-ray diffraction (XRD) were grown by dissolving the compound (10 mg) in CH_2_Cl_2_, layering in a glass vial with hexane, and leaving it to crystallize for 4 days. Me_4_N**4**.HCl; yield 320 mg, 78%; HRMS(ESI^–^): *m*/*z* 342.2832 (M^–^, 9%), 339.2942 (100%), calcd. 342.2850 (M^–^) and 339.2953 (100%); ^1^H NMR (600 MHz, Acetone-D_6_) δ 5.59 (s, NH_2_), 4.08 (1H, s, CH_carborane_), 3.86 (1H, s, CH_carborane_), 3.79, (1H, s, CH_carborane_), 3.42 (12H, s, Me_4_N^+^). ^1^H–B{^11^B}NMR (from 2D-COSY-NMR): δ 3.46 (1H, s, B(8′)-H), 3.42 (1H, overlap, B(8)-H), 2.84 (1H, br. s, B(10′)-H), 2.75 (1H, s, B(10)-H), 1.94 (1H, br s, B(9′,12′)-H), 1.75 (1H, br. s, B(7)-H), 1.60 (1H, br s, B(5)-H); ^11^B NMR (193 MHz, Acetone-D_6_) δ 4.49 (2B, 2d, *J* = 156.3 Hz, *J* = 158.3 Hz, B8′,8), −1.33 (2B, 2d, *J* = 158.3 Hz, *J* = 156.3 Hz, B10′,10), −5.7 (2B, d, *J* = 156.3 Hz, B4,9), −7.30 (5B, overlap, B4′,7′,12,9′,12′), −12.9 (2B, 2d, *J* = 125.5 Hz, *J*_2_ = 131.2 Hz, B7,5), −18.74 (3B, d, *J* = 145 Hz, B11′,5′,11 overlap), −19.42 (1B, d, *J* = 119.7 Hz, B6), −24.0 (1B, d, *J* = 169.8 Hz, B6′; ^13^C {^1^H} NMR (151 MHz, Acetone-D_6_) δ 85.61 (C_carborane_), 59.71 (C_carborane_), 55.22 (CH, Me_4_N^+^), 54.85 (CH_carborane_), 53.92 (CH_carborane_). In another experiment, after evaporation of MeOH the crude product was precipitated with an excess of aqueous Me_4_NCl without acidification, washed with water (3 × 10 mL), filtered, and dried in a vacuum. Pure compound was isolated by flash chromatography on Bűchi 120 g RP C18 column using 50% MeOH; flow rate 20 mL/min. Fractions containing the product were combined, MeOH was evaporated and the compound was dried in a vacuum. Yield of Me_4_N**4**: 290 mg, 62%. Crystals for XRD were grown by dissolving the compound (10 mg) in CH_2_Cl_2_ in a glass vial, and placing this vial into a larger one filled with hexane at the bottom. This was closed by a screw cup and the compound was left to crystallize for 10 days.

##### Amine **5**^–^

The diazide Me_4_N**3** (250 mg, 0.42 mmol) was reduced and the corresponding diamine was isolated by a procedure analogous to that used for the above-described monosubstituted amine hydrochloride; the identical amounts of CoCl_2_·6H_2_O (50 mg, 2.1 mmol) an NaBH_4_ (500 mg, 13 mmol) were used. The final purification was made using flash chromatography on a Bchi 80 g RP C18 column using 45% MeOH and the product was crystallized from CH_2_Cl_2_-hexane. Yield of Me_4_N**5**.HCl: 195 mg, 81%, an orange-red solid; HRMS (ESI^–^): *m*/*z* 357.2945 (M^–^, 9%), 354.3054 (100%), calcd. 357.2960 (M^–^) and 354.3063 (100%); NMR: ^11^B NMR (193 MHz, Acetone-D_6_) δ 4.57 (d, J = 148.0 Hz), −2.60 (d, J = 142.0 Hz), −6.59 (d, J = 125.2 Hz), −12.78 (2d = 147.9 Hz), −19.10 (t, 2d = 142.3 Hz). ^1^H NMR (600 MHz, Acetone-D_6_) δ 3.98 (s, 2H), 3.20–3.11 (m, 16H), 2.56 (s, 1H), 2.39 (s, 2H). ^13^C NMR (151 MHz, Acetone-D_6_) δ: 129.58, 126.97, 85.15, 70.82, 62.57, 57.76, 45.29, 29.56, 20.56.

#### Attempt to Synthesize the Azides Using Me_3_SiN_3_, Isolation of [(1-Me_3_Si–1,2-C_2_B_9_H_10_)(1′,2′-C_2_B_9_H_11_)-3,3′-Co]Me_4_N (**6**^–^) and [(1,1′-Me_3_Si–C_2_B_9_H_10_)_2_-3,3′-Co]Me_4_N (**7**^–^)

The cesium salt of cobalt bis(dicarbollide) (Cs**1**, 1.0 g, 2.20 mmol) was metalated with *n*-BuLi (2.5 M in hexane, 2.1 mL, 2.42 mmol) using the procedure described above applied in the synthesis of **2**^**–**^ and **3**^**–**^. Then, trimethylsilyl azide (Aldrich, 0.65 mL, 2.42 mmol) was added dropwise from a syringe, and the reaction was continued as in the above case. The reaction mixture turned to violet and only two products could be distinguished by MS and HPLC analysis as the mono and disilyl derivatives. The reaction was quenched with the careful addition of H_2_O from a syringe (25 mL), and the organic solvent was distilled off using a rotary evaporator, keeping the temperature of a water bath below 35 °C. The mixture of the products was extracted into diethyl ether (3 × 25 mL), water (10 mL) was added to the combined ether fractions, and the organic solvent was removed in a vacuum. The minimum volume of MeOH was added for the dissolution of a purple *semi*-solid material, and an excess of aqueous solution of Me_4_NCl was added to precipitate the crude product. The precipitate was rapidly filtered, and dried for 10 min in a vacuum. The separation of compounds **6**^**–**^ and **7**^**–**^ was performed using flash chromatography on a Bchi 120 g C18 RP column starting with 60% MeOH and increasing the methanol content to 75%; flow rate 20 mL/min. The fractions containing the individual products **6**^**–**^ and **7**^**–**^ were combined, and MeOH was quickly evaporated. Then the products were precipitated again with Me_4_NCl, washed with water (3 × 10 mL), quickly filtered and dried in a vacuum. Yield of Me_4_N**6**: 185 mg, 18%, pale yellow solid; HRMS (ESI^–^): *m*/*z* 399.3142 (M̅, 14%), 396.3239 (100%), calcd. 399.3155 (M^–^) and 396.3244 (100%). ^1^H NMR (600 MHz, Acetone-D_6_) δ 4.07 (1H, s, −CH_carborane_), 3.88, 3.77 (2H, 2s, CH_carborane_), 3.50 (12H, s, Me_4_N^+^), 0.33 (9H, s, -SiMe_3_). ^1^H{^11^B}-(from selective 2D-NMR): δ 3.56 (1H, br s, B(8′)-H), 3.16 (1H, br s, B(10)-H), 2.96 (1H, br s, B(10′)-H), 2.31 (1H, br s, B(7)-H), 2.25 (1H, br, B(4)-H), 2.01 (3H, overlap, B(4,7,7′)-H), 1.85 (4B, overlap, B(9,9′,12,12′)-H), 1.61 (1H, br s, B(5′)-H), 1.56 (1H, overlap, B(5)-H), 1.54 (1B, overlap, B(11′)-H), 1.47 (1B, s, B(11)-H). ^11^B NMR (193 MHz, Acetone-D_6_) δ 7.21 (1B, d, *J* = 127.4 Hz, B8), 5.02 (1B, d, *J* = 27.02, B8′), 2.52 (1B, d, *J* = 135.1 Hz, B10), 0.25 (1B, d, *J* = 137 Hz, B10′), −2.94 (1B, d, *J* = 140.9 Hz, B4), −5.51 (1B, d, *J* = 196.7 Hz, B7), −6.48 (2B, d, *J* = 175.6 Hz, B4′7′), −7.42 (2B, d, *J* = 182.6 Hz, B9′,12′), −8.32 (2B, d, *J* = 154.4 Hz, B9,12), −15.2 (1B, s, *J* = 166.0 Hz, B5), −16.07 (1B, d, *J* = 169.8 Hz, B11′), −18.52 (1B, d, *J* = 167.9 Hz, B11), −19.38 (1B, d, *J* = 164.0 Hz, B5′), −22.28 (1B, d, *J* = 162.1 Hz, B6), −24.00 (1B, d, *J* = 179.5, B6′). ^13^C NMR (151 MHz, Acetone-D_6_) δ 59.08 (C_carborane_), 57.21 (C_carborane_), 55.20 (4C, Me_4_N^+^), 48.70 (CH_carborane_), 46.88 (CH_carborane_). ^29^Si NMR (119 MHz, Acetone-D_6_) δ 10.06 (1Si, Me_3_Si).

##### Yield of Me_4_N**7**

870 mg, 73%, purple solid; HRMS (ESI^–^) *m*/*z* 471.3551 (M^–^, 18%), 468.3639 (100%), calcd. 471.3561 (M^–^, 18%) and 468.3643 (100%); ^1^H NMR (600 MHz, Acetone-D_6_) δ 3.80 (2H, CH_carborane_), 3.48 (12H, m, Me_4_N^+^), 0.34 (18H, s, (Me_3_Si)_2_). ^1^H{^11^B}-(from selective 2D-NMR): δ 3.81 (1H, s, B(8′)H), 3.15 (3H, s, B(10,10′,4)-H), 3.14 (2H, s, B(12, 12′)-H), 3.00 (1H, B(4′)-H), 2.40 (2H, br, B(7,7′)-H), 2.19 (2H, br s, B(9,9′)-H, 1.60 (4H, br s, overlap, B(5,5′,11,11′)-H). ^11^B NMR (193 MHz, Acetone–D_6_) δ 6.30 (2B, d, *J* = 148.6 Hz, B8,8′), 1.88 (2B, d, *J* = 142.8 Hz, B10,10′), −4.06 (3B, d, *J* = 129.3 Hz, B4,7,7′), −4.74 (3B, d, *J* = 129.3 Hz, B4′9,9′), −8.46 (2B, d, *J* = 148.6 Hz, B12,12′), −15.14 (2B, d, *J* = 150.5 Hz, B5,5′), −16.90 (2B, d, *J* = 156.3 Hz, B11,11′), −21.30 (2B, d, *J* = 167.9 Hz, B6,6′). ^13^C NMR (151 MHz, Acetone-D_6_) δ 55.92 (1C, C_carborane_), 55.23 (12C, Me_4_N^+^), 54.64 (C_carborane_), 52.03 (CH_carborane_), 3.28 (Me_3_Si). ^29^Si NMR (119 MHz, Acetone-D_6_) δ 10.93 (2Si, (Me_3_Si)_2_).

#### [Me_4_N][(1-N_3_-(CH_2_)_2_–1,2-C_2_B_9_H_10_)(1′,2′-C_2_B_9_H_11_)-3,3′-Co(III)] (Me_4_N**8**)

Me_4_N[(1-(CH_3_SO_2_O–C_2_H_4_–1,2-C_2_B_9_H_10_)(1,2-C_2_B_9_H_11_)-3,3′-Co(III)] (1.207 g, 2.32 mmol) was dried at room temperature together with sodium azide (0.50 g, 7.8 mmol) for 4 h. Then, DMF (15 mL, anhydrous, Sigma-Aldrich) was injected, and the reaction mixture was stirred at 45 °C overnight. The excess inorganic azide was removed by filtration, the solid was washed with DMF (2 × 3 mL), and the organic solvent was removed in vacuum at 45 °C. The crude product was chromatographed on a C18-modified silica gel column in reverse phase mode by 50% aqueous acetone (v/v). Pure fractions were combined, and dissolved in a minimal volume of MeOH and the product was precipitated with an excess of aqueous Me_4_NCl, filtered, and immediately dried in a vacuum. Me_4_N**8**, an orange solid, yield 697 mg (64%). *m*/*z* (ESI^–^) 396.3038 (M^–^, 10%), 393.3142 (100%), calcd. 396.3075 (M^–^) and 393.3173 (100%). ^1^H NMR (600 MHz, Acetone-D_6_): δ 4.12 (1H, s, CH_carborane_), 3.81 (1H, s, CH_carborane_), 3.73 (1H, s, CH_carborane_), 3.52–3.46 (2H, m, C*H*_2_-N_3_), 3.44 (12H, s, Me_4_N^+^), 3.10–3.04 (1H, m, C*H*_2_-), 2.66–2.60 (1H, m, C*H*_2_). ^1^H{^11^B} NMR (600 MHz, Acetone-D_6_): δ 4.12 (1H, s, Cage-CH_carborane_), 3.81 (1H, s, CH_carborane_), 3.74 (1H, br s, B–H), 3.73 (1H, s, CH_carborane_), 3.58 (1H, br s, B–H), 2.96 (1H, br s, B–H), 2.91 (1H, br s, B–H), 2.80 (1H, br s, B–H), 2.71 (4H, br s, B–H), 1.97 (2H, br s, B–H), 1.92 (2H, br s, B–H), 1.64 (4H, br s, B–H), 1.54 (1H, br s, B–H). ^11^B NMR (192 MHz, Acetone-D_6_; Et_2_O.BF_3_): δ 5.71 (2B, d, *J* = 140 Hz, B8,8′), 0.07 (1B, d, *J* = 140 Hz, B10), −0.32 (1B, d, *J* = 137 Hz, B10′), −6.09, −6.89 (7B, 3d, overlap, B4,4′, B7,7′, 9,9′, 12), −8.07 (1B, d, *J* = 143 Hz, B12′), −16.38 (1B, d, *J* = 166 Hz, B5), −17.33 (1B, br d, B5′), −18.62 (2B, d, *J* = 155 Hz, B11,11′), −20.63 (1B, d, *J* = 159 Hz, B6), −24.05 (1B, d, *J* = 164 Hz, B6′). ^13^C{^1^H} NMR (150 MHz, Acetone-D_6_): δ 66.61 (1C, C_carborane_), 57.45 (1C, C_carborane_), 56.02 (4C, Me_4_N^+^), 53.81 (1C, CH_carborane_), 52.17 (1C, *C*H_2_–N_3_), 51.92 (1C, CH_carborane_), 39.32 (1C, *C*H_2_).

#### [Me_4_N][(1-N_3_-(CH_2_)_3_-(1,2-C_2_B_9_H_10_)(1′,2′-C_2_B_9_H_11_)-3,3′-Co(III)] (Me_4_N.**9**)

The compound was prepared using Me_4_N[(1-CH_3_SO2O–C_3_H_6_–1,2-C_2_B_9_H_10_)(1,2-C_2_B_9_H_11_)-3,3′-Co(III)] (500 mg, 0.94 mmol) and sodium azide (0.50 g, 7.8 mmol) for 4 h. The reaction conditions and isolation were the same as those described for Me_4_N**8**. Me_4_N**9**: an orange solid, yield 416 mg, 92%; HRMS (ESI^–^): 410.3228 (M^–^, 10%), 407.3332 (100%), calcd. 410.3234 (M^–^) and 407.3330 (100%); ^1^H NMR (600 MHz, Acetone-D_6_): δ 4.08 (1H, s, CH_carborane_), 3.73 (1H, s, CH_carborane_), 3.64 (1H, s, CH_carborane_), 3.45 (12H, s, Me_4_N^+^), 3.39–3.35 (1H, m, C*H*_2_-N_3_), 3.32–3.28 (1H, m, C*H*_2_-N_3_), 2.86–3.78 (1H, m, C*H*_2_-C_carborane_), 2.46–2.41 (1H, m, C*H*_2_-C_carborane_), 1.88–1.77 (2H, m, C*H*_2_-CH_2_−). ^1^H{^11^B} NMR (600 MHz, Acetone-D_6_): δ 4.08 (1H, s, CH_carborane_), 3.75 (1H, br s, B–H), 3.73 (1H, s, CH_carborane_), 3.64 (1H, s, CH_carborane_), 3.58 (1H, s, B–H), 2.12 (1H, br s, B–H), 2.93 (2H, br s, B–H), 2.86 (1H, br s, B–H), 2.65 (2H, br s, B–H), 1.97 (2H, br s, B–H), 1.93 (2H, s, B–H), 1.71 (2H, br s, B–H), 1.63 (2H, br s, B–H), 1.49 (2H, br s, B–H); ^11^B NMR (192 MHz; Acetone-D_6_; Et_2_O.BF_3_): δ 5.68 (1B, d, *J* = 143 Hz, B8), 5.26 (1B, d, *J* = 140 Hz, B8′), −0.24 (2B, d, *J* = 142 Hz, B10,10′), −6.21 (2B, br s, B4,4′), −7.01 (5B, br d, 7,7′, 9,9′, 12), −8.07 (1B, br s, B12′), −16.03 (1B, d, *J* = 154 Hz, B5), −17.35 (1B, d, *J* 157, B5′), −18.86 (2B, d, *J* = 154 Hz, B11,11′), −20.39 (1B, d, *J* = 167 Hz, B6), −24.12 (1B, d, *J* = 165 Hz, B6′); ^13^C{^1^H} NMR (150 MHz; (CD_3_)_2_CO): δ 69.20 (1C, C_carborane_), 57.70 (1C, C_carborane_), 56.02 (4C, Me_4_N^+^), 53.87 (1C, CH_carborane_), 51.73 (1C, CH_carborane_), 51.65 (1C, *C*H_2_–N_3_), 37.78 (1C, *C*H_2_–C_carborane_), 30.85 (1C, *C*H_2_–CH_2_–C_carborane_).

#### [Me_4_N][1,1′-(N_3_–C_2_H_4_–1,2-C_2_B_9_H_10_)_2_-3,3′-Co(III)] (Me_4_N.**10**)

Me_4_N[(1-(CH_3_SO_2_O–C_2_H_4_–1,2-C_2_B_9_H_10_)_2_-3,3′-Co(III)] (1.0 g, 1.56 mmol) was dried and reacted with sodium azide (1.0 g, 35.50 mmol) in DMF (10 mL, anhydrous - Sigma-Aldrich) under similar conditions, which were used for the monosubstituted derivatives **8**^**–**^ and **9**^**–**^. The excess inorganic azide was removed by filtration, the solid residue was washed with dry Et_2_O (3 × 5 mL), the organic extracts were combined and the solvents were removed in a vacuum increasing the bath temperature gradually to 50 °C. The crude product was chromatographed twice on a silica gel column 20 × 2.5 cm I.D. using a CH_2_Cl_2_-MeOH solvent mixture increasing the MeOH content from 8 to 12%. The main red band was collected, the volatiles were evaporated, and the solid was treated with hexane (2 × 10 mL) and dried again. The residue was dissolved in a minimal volume of MeOH and precipitated with an excess of aqueous Me_4_NCl, filtered and immediately dried in a vacuum. Me_4_N**10**, red solid, yield 570 mg (68%); HRMS (ESI^–^): *m*/*z* 465.3397 (M^–^, 11%), 462.3497 (100%), calcd. 465.3407 (M^–^) and 462.3501 (100%); ^1^H NMR (600 MHz; (CD_3_)_2_CO): δ 3.82 (2H, br s, CH_carborane_), 3.51–3.40 (4H, m, C*H*_2_-N_3_), 3.44 (12H, s, Me_4_N^+^), 3.40–3.34 (2H, m, C*H*_2_), 2.75–2.70 (2H, m, C*H*_2_). ^1^H{^11^B} NMR (600 MHz, Acetone-D_6_): δ 4.06 (2H, br s, B–H), 3.82 (2H, s, CH_carborane_), 2.86 (2H, br s, B–H), 2.43 (2H, br s, B–H), 2.18 (2H, br s, B–H), 1.79 (5H, br s, B–H), 1.67 (5H, br s, B–H). ^11^B NMR (192 MHz, Acetone-D_6_; Et_2_O.BF_3_): δ 7.37 (2B, d, *J* = 144 Hz, B8,8′), −0.81 (2B, d, *J* = 141 Hz, B10,10′), −4.41 (2B, d, *J* = 143 Hz, B4,4′), −5.93 (2B, d, *J* = 145 Hz, B7,7′), −7.66 (2B, d, *J* = 148 Hz, B9,9′), −9.45 (2B, d, *J* = 141 Hz, B12,12′), −15.49 (2B, d, *J* = 154 Hz, B5,5′), −16.95 (2B, d, *J* = 155 Hz, B11,11′), −20.57 (2B, d, *J* = 157 Hz, B6,6′). ^13^C{^1^H} NMR (150 MHz, Acetone-D_6_): δ 67.58 (2C, C_carborane_), 58.67 (2C, CH_carborane_), 56.01 (4C, Me_4_N^+^), 52.28 (2C, *C*H_2_–N_3_), 39.63 (2C, *C*H_2_).

#### [Me_4_N][1,1′-(Ph-triazolyl-C_2_H_4_–1,2-C_2_B_9_H_10_) (1′,2′-C_2_B_9_H_11_)-3,3′-Co(III)] (Me_4_N**11**)

Me_4_N**8** (84 mg, 0.180 mmol) was dried under a vacuum at room temperature for 4 h. Dry Ethanol (20 mL) was added followed by phenylacetylene (0.1 mL, 0.912 mmol), CuI (6 mg, 0.030 mmol) and diisopropyl amine (DIPEA, 0.4 mL, 2.256 mmol). The reaction mixture was stirred at 40 °C for 2 days. Analysis of the reaction mixture by HRMS showed only the presence of the expected click product (95% purity based on MS). The pure product was obtained after dissolving the crude product in a minimum volume of MeOH and precipitation by an excess of aqueous Me_4_NCI, and crystallization from CH_2_Cl_2_-hexane. Me_4_N**11**, and orange solid, yield: 0.097 g, 95%; MS (ESI) *m*/*z* 498.3546 (M^–^, 12%), 495.3636 (100%), calcd. 498.3563 (M^–^) and 495.3647 (100%); ^11^B NMR δ_B_(192 MHz; (CD_3_)_2_CO; Et_2_O.BF_3_): 5.80 (2B, d, *J* 140, B8,8′), 0.18 (1B, d, J 136, B10), −0.18 (1B, d, J 139, B10′), −6.79 (7B, d, J 140, B4,4′, 7,7′, 9,9′, 12), −7.91 (1B, br d, B12′), −16.60 (1B, br d, B5), −17.37 (1B, d, J 159, B5′), −18.58 (2B, d, J 159, B11,11′), −20.67 (1B, d, J 159, B6), −24.02 (1B, d, J 153, B6′). 1H NMR δ_H_ (600 MHz; (CD_3_)_2_CO): 8.37 (1H, s, CH_triazole_), 7.86 (2H, d, ^3^J_H–H_ 7.71, Ph*H*, CH_triazole_), 7.43 (2H, t, ^3^J_H–H_ 7.86, Ph*H*), 7.31 (1H, t, ^3^J_H–H_ 7.52, Ph*H*), 4.67–4.55 (2H, m, C*H*_2_-N_triazole_), 4.20 (1H, s, C*H*_carborane_), 3.83 (1H, s, C*H*_carborane_), 3.78 (1H, s, C*H*_carborane_), 3.49–3.46 (1H, m, C*H*_2_–C(C*H*_carborane_), 3.45 (12H, s, Me_4_N^+^), 3.07–3.01 (1H, m, C*H*_2_); ^13^C{^1^H} NMR δ_C_ (150 MHz; (CD_3_)_2_CO): 147.68 (1C, *C*_triazole_), 132.24 (1C, Ph*C*), 129.62 (2C, Ph*C*), 128.55 (1C, Ph*C*), 126.13 (2C, Ph*C*), 121.57 (1C, *C*H_triazole_), 66.10 (1C, *C*_carborane_), 57.64 (1C, *C*H_carborane_), 56.00 (4C, Me_4_N^+^), 53.95 (1C, *C*H_carborane_), 52.00 (1C, *C*H_carborane_), 51.16 (1C, *C*H_2_–N_triazole_), 40.49 (1C, −CH_2_–*C*H_2_–C_carborane_).

#### [Me_4_N][1,1′-(Ph-triazolyl-C_3_H_6_–1,2-C_2_B_9_H_10_) (1′,2′-C_2_B_9_H_11_)-3,3′-Co(III)] (Me_4_N**12**)

Me_4_N**9** (177 mg, 0.368 mmol) was dried under a vacuum at room temperature for 4 h. Dry Ethanol (25 mL) was added followed by phenylacetylene (0.15 mL, 1.366 mmol), CuI (6 mg, 0.030 mmol) and DIPEA (0.7 mL, 4.019 mmol). The reaction mixture was stirred at 40 °C for 5 days. Analysis of the reaction mixture by HRMS showed high conversion to the expected click product (90%). The crude product was purified on C18-modified silica in reverse phase mode using 55% aqueous MeOH. Pure fractions (according to HRMS analysis) were combined and evaporated to dryness. The product was dissolved in a minimal volume of MeOH and precipitated using an excess of aqueous Me_4_NCl. The solid was filtered, dried, and crystallized from CH_2_Cl_2_-hexane. Me_4_N**12**, Yield: 0.190 g (88%); MS (ESI) *m*/*z* 512.3707 (M^–^, 12%), 509.3801 (100%), calcd. 512.3722 (M^–^) and 509.3805 (100%); ^11^B NMR δ_B_(192 MHz; (CD_3_)_2_CO; Et_2_O.BF_3_): 5.51 (2B, br d, B8,8′), −0.21 (2B, d, *J* 141, B10,10′), −7.01 (7B, d, *J* 144, B4,4′, 7,7′, 9,9′, 12), −8.04 (1B, br d, B12′), −16.21 (1B, br d, B5), −17.35 (1B, d, *J* 162, B5′), −18.84 (2B, d, *J* 153, B11,11′), −20.48 (1B, d, *J* 155, B6), −24.11 (1B, d, *J* 158, B6′); ^1^H NMR δ_H_(600 MHz; (CD_3_)_2_CO): 8.36 (1H, s, C*H*_triazole_), 7.88 (2H, d, ^3^J_HH_ 7.65, Ph*H*), 7.42 (2H, t, ^3^J_HH_ 7.95, Ph*H*), 7.31 (1H, t, ^3^J_HH_ 7.36, Ph*H*), 4.49–4.41 (2H, m, C*H*_2_-N_triazole_), 4.06 (1H, s, C*H*_carborane_), 3.67 (1H, s, C*H*_carborane_), 3.57 (1H, s, C*H*_carborane_), 3.44 (12H, s, Me_4_N^+^), 2.87–2.82 (1H, m, CH_2_C*H*_2_-C_carborane_), 2.45–2.40 (1H, m, CH_2_C*H*_2_), 2.27–2.14 (2H, m, C*H*_2_-CH_2_). ^13^C{^1^H} NMR δ_C_ (150 MHz; (CD_3_)_2_CO): 147.85 (1C, *C*_triazole_), 132.28 (1C, Ph), 129.55 (2C, Ph), 128.53 (1C, Ph), 126.25 (2C, Ph), 121.35 (1C, *C*H_triazole_), 68.86 (1C, *C*_carborane_), 57.93 (1C, *C*H_carborane_), 56.01 (4C, Me_4_N^+^), 54.04 (1C, *C*H_carborane_), 51.78 (1C, *C*H_carborane_), 50.34 (1C, *C*H_2_–N_triazole_), 37.57 (1C, *C*H_2_–C_carborane_), 32.19 (1C, *C*H_2_–CH_2_−).

#### Synthesis of the Amine **4**^–^ via [(1-N_3_OC–1,2-C_2_B_9_H_10_)(1′,2′-C_2_B_9_H_11_)-3,3′-Co]^−^ Intermediate Corresponding to Curtius Rearrangement

The carboxylic acid of formula [(1-HOOC–1,2-C_2_B_9_H_10_)(1′,2′-C_2_B_9_H_11_)-3,3′-Co]Cs prepared according to ref^46^ (300 mg, 0.60 mmol) was dissolved under stirring in anhydrous dioxane (10 mL) under argon in a Schlenk-type flask. Anhydrous pyridine (0.5 mL, mmol) followed by SOCl_2_ (0.60 mL, mmol) were injected through a rubber septum. The reaction mixture was stirred for 4 h and then the volatiles were removed under vacuum. Fresh dioxane (12 mL) and water (2 mL) were injected followed up with the addition of an excess of solid NaN_3_ (0.5 g, mmol), and the reaction slurry was stirred at 45 °C for 6h. MS analysis showed almost complete conversion to [(1-N_3_OC–1,2-C_2_B_9_H_10_)(1′,2′-C_2_B_9_H_11_)-3,3′-Co]^−^ along with a smaller amount of starting acid and amine **4**^**–**^ (less than ca. 5% relative intensity). The volatiles were removed in a vacuum and the product was isolated by flash chromatography on Bűchi 80 g RP C18 column using 50 to 55% aqueous acetone (visual detection and UV at 310 nm), flow rate 10 mL/min. The organic solvent was rapidly removed using a rotary evaporator (at bath temperature 35 °C maximum); the compound was then extracted from water to Et_2_O (4 × 20 mL), water (5 mL) was added to combined ether extracts, and the organic solvent was quickly removed in a vacuum without heating the flask. Methanol was dropwise added to a *semi*-solid residue until complete dissolution and the compound was precipitated with an excess of aqueous solution of Me_4_NCl. After standing for 10 min, the precipitate was collected by filtration and dried in a vacuum. Yield of the acyl azide Me4N.**13**: 210 mg, 75%. HRMS (ESI^–^): *m*/*z* 396.2682 (M^–^, 10%), 393.2795 (100%), calcd. 396.2697 (M^–^, 10%) and 393.2806 (100%); ^1^H NMR (600 MHz, Acetone-D_6_) δ 4.02, 3.93 (2H, CH_carborane_), 3.68 (1H, CH_carborane_), 3.49 (12H, Me_4_N^+^). ^11^B NMR (193 MHz, Acetone-D_6_) δ 6.62 (1B, d, *J* = 152.5 Hz, B8), 6.20 (1B, d, *J* = 148.6 Hz, B8′), 1.98 (1B, d, *J* = 140.9 Hz, B10), −0.19 (1B, d, *J* = 146.7 Hz, B10′), −5.78 (3B, d, *J* = 144.8 Hz, B4,7,9), −6.53 (2B, d, *J* = 146.7 Hz, B4′7′), −6.95 (3B, d, *J* = 150.5 Hz, B9′,12,12′), −16.68 (1B, d, *J* = 167.9 Hz, B5), −17.00 (1B, d, *J* = 158.3 Hz, B11), −17.52 (1B, d, *J* = 158.3 Hz, B5′), −19.22 (1B, d, *J* = 156.3 Hz, B11′), −20.57 (1B, d, *J* = 173.7 Hz, B6), −23.61 (1B, d, *J* = 169.8 Hz, B6′). ^13^C NMR (151 MHz, Acetone-D_6_) δ 175.86 (1C, C=O), 63.95 (1C, C_carborane_), 55.21 (4C, Me_4_N^+^), 54.64 (CH_carborane_), 53.98 (CH_carborane_), 48.41 (CH_carborane_).

##### Thermal Rearrangement and Hydrolysis to Amine **4**^–^

The acyl azide Me_4_N**13** 100 mg was dissolved in freshly distilled dioxane 22 mL to which diluted HCl (3 M, 3.0 mL) was added. The solution was transferred to a pressure ACE tube, and the tube was closed with a screw cap and heated to 100 °C for 5.5 h. After the mixture was cooled down to room temperature, the screw cap was carefully removed and the solution was evaporated in vacuum. The product was purified by flash chromatography on a Bchi 80 g RP C18 column using 50 to 55% aqueous MeOH, flow rate of 15 mL/min. The orange band corresponding to amine **4**^**–**^ was collected, the volume of the effluent was reduced to 10 mL in a vacuum, MeOH and a few drops of 3 M HCl were added and the compound was precipitated with an excess of Me_4_NCl. The precipitate was collected by filtration and dried in a vacuum. Yield of Me_4_**4**.HCl 75 mg, 78%. The compound showed identical NMR and MS spectral properties with the amine prepared using the route described above via azide **2**^**-**^.

***Caution!*** Highly flammable hydrogen gas is evolved when reductions are performed with NaBH_4_. The reaction must be carried out in a well-ventilated hood avoiding contact with an open source of the heat. Appropriate personal protective equipment (PPE) including eye protection, suitable gloves, and a lab coat must be worn.

## Results and Discussion

As known from substitution chemistry on carboranes, the compounds with functional groups attached directly to cage atoms often show quite different chemical and physicochemical properties from those bound via a pendant group.^5^ This is attributable to the electronic and steric effects of bulky boron cages in the proximity. In addition, there are differences between the physicochemical properties of carbon and boron-substituted species.^5,47^

Although B(8) substituted primary amines of the cobalt bis(dicarbollide) ion were reported a relatively long time ago,^3,13,17^ the compounds that would contain cage carbon–nitrogen bonds remained unknown. Therefore, we focus herein on the compounds, which represent the first members of the series of azides and primary amines, while outlining the chemical, spectral, structural, and acid–base properties (of the amines).

**Scheme 1:**
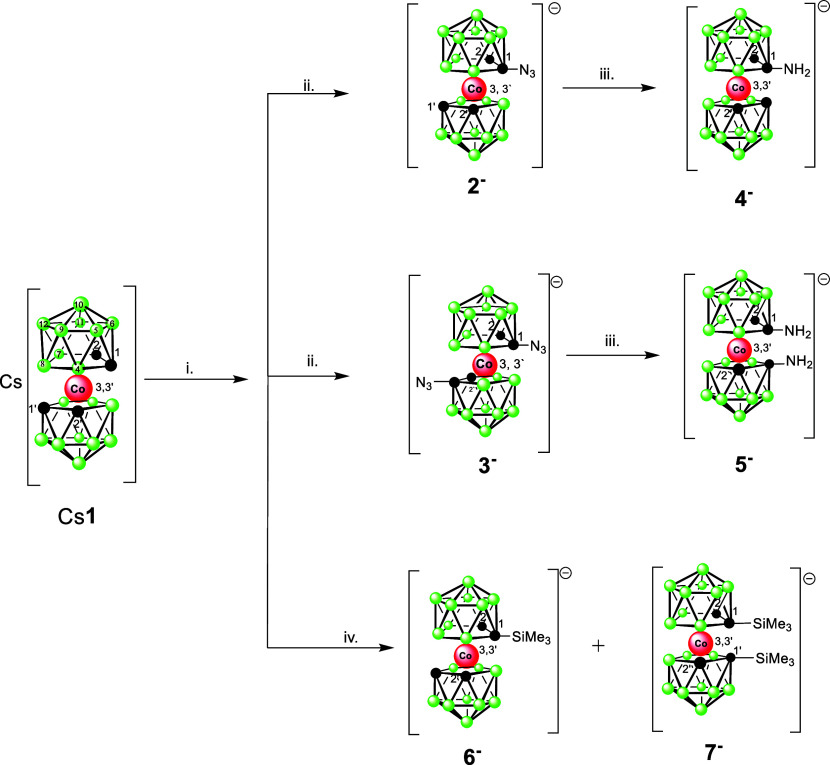
Synthesis of Amines and Other Products Using Reagents with Azido Groups Conditions: i. BuLi, DME, −78° C; ii. TsN_3_ in toluene −78 to 25° C, 16h, extraction, separation using RP chromatography; iii. NaBH_4_/CoCl_2_ in 50% MeOH, r.t., 2 h; iv. BuLi, DME, −78° C, then Me_3_SiN_3_ in toluene −78 to 25° C, extraction, RP chromatography.

Two independent methods for the synthesis of mono- and diamines have been adapted from organic protocol.^1,48^ The first method comprises the low-temperature reaction of lithiated cobalt bis(dicarbollide) with organic azides and the subsequent reduction of the azido group. This procedure produces either mono or diamine in two reaction steps followed with the isolation of corresponding products (Scheme 1). The second approach suitable for the synthesis of monosubstituted amines is based on Curtius rearrangement of the corresponding acyl azide. It should be noted that similar methods were previously applied in the synthesis of azides,^49^ acyl azides,^50^ and corresponding amines^51^ of neutral dicarba-*closo*-dodecaborates (carboranes).

### Synthesis of Azides and Related Irregular Pathways

**Figure 1:**
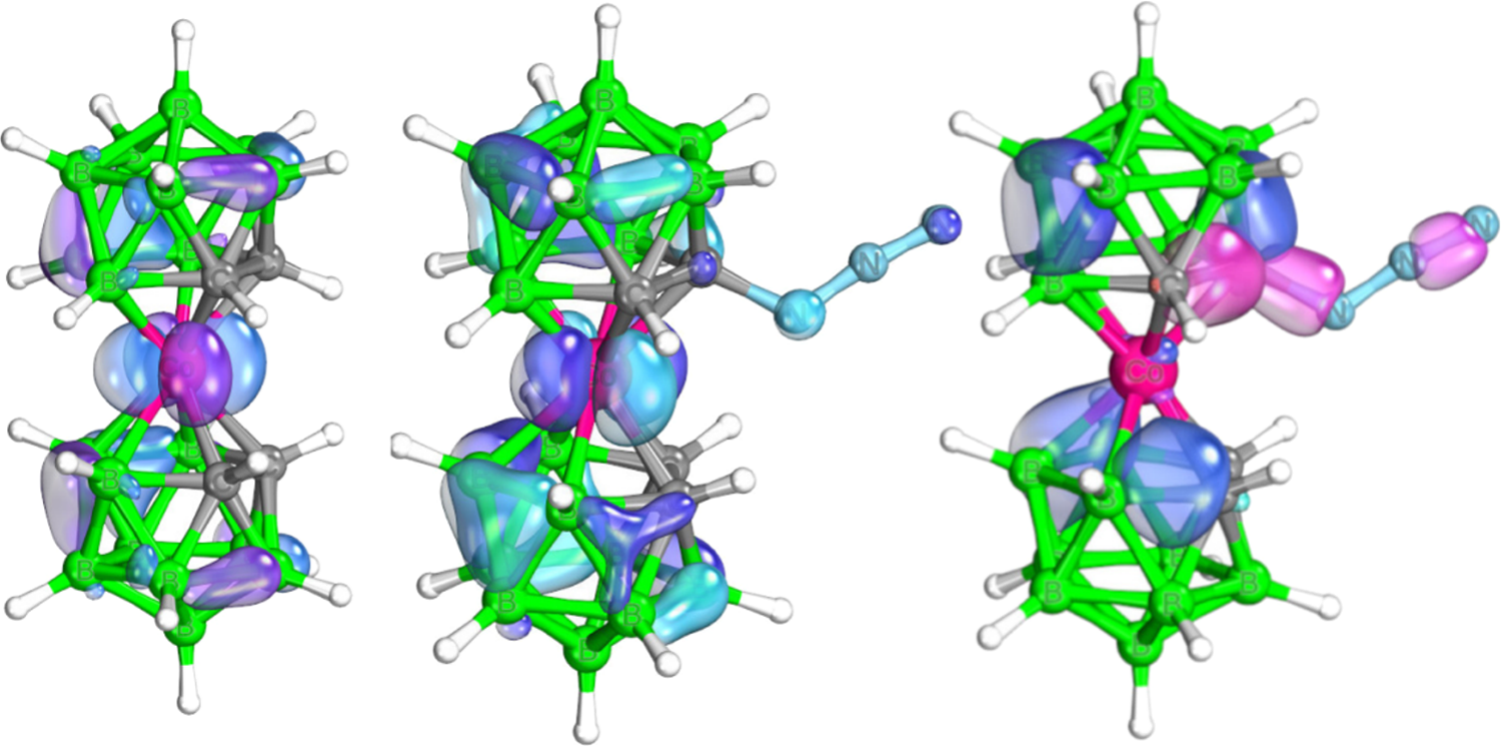
From left to the right: Kohn–Sham HOMO orbital of COSAN, COSAN-azide (light blue and dark blue are referred to regions in which the HOMO function is negative and positive, respectively), and examples of the IBO orbitals (see the Computational Details section) classified as the 2c–2e (pink) pattern and 3c–2e (blue) pattern.

The output of the first step leading to azides strongly depends on the polarization of the leaving group present in the organic azide, namely, on its ability to act as a sufficiently strong electrophile (see Scheme 1). Consequently, the low-temperature reaction of tosyl azide produces high yields of the ions of the formulas [(1-N_3_–1,2-C_2_B_9_H_10_)(1′,2′-C_2_B_9_H_11_)-3,3′-Co(III)]^−^ (**2**^–^) and *rac*-[1,1′-(N_3_–1,2-C_2_B_9_H_10_)_2_-3,3′-Co(III)]^−^ (**3**^**–**^) with one or two diazo groups attached to the cage carbon atoms C(1) and C(1′). The proportion of the products in the reaction mixture depends on the BuLi ratios used for metalation. However, the monosubstituted ion **2**^**–**^ was always observed as the main product resulting from this reaction, even with the ratio of the starting reagents being 1:2.2. According to MS spectra, the mixture also contained the unreacted starting ion (**1**^**–**^) and a small quantity of the amino derivative. The bright-yellow monosubstituted ion **2**^**–**^ and the orange disubstituted species **3**^**–**^ could be isolated in good purity using liquid–liquid extraction into ether followed by flash RP chromatography and fully characterized using spectral methods, although their stability was surprisingly low (in comparison with similar species comprising the N_3_ group separated from cage by an alkyl pendant group, which are presented in the following section). The slow decomposition (days) in solution was observed, occurring more slowly when they were stored as solids at −33 °C (weeks). This process resulted in the formation of a gray solid, apparently elemental cobalt, and the presence of parent and N_3_- substituted *nido*-C_2_B_9_H_12_^–^ ions was confirmed using MS. Figure 1 reveals the nature of the HOMO of the parent cobalt bis(dicarbollide) ion and its azide derivative, with the difference between then being quite apparent. This difference thus indicates the character of bonding of the azide derivative in relation to its parent counterpart. The IBO charge analysis revealed an interesting feature. Whereas the q(IBO) of C in the parent cobalt bis(dicarbollide) ion was computed as −0.22, that of C to which N_3_ is bonded amounts to 0.00, with the electron-withdrawing character of the azide group in this system being quite significant. The IBO HOMO–LUMO difference in derivative **2**^**–**^ amounts to 4.37 eV, which indicates a smaller stability of the azide with respect to the parent ion with the IBO HOMO–LUMO gap of 4.52 eV. Figure 1 also provides examples of the IBO localized orbitals^44^ projected from the canonical ones (HOMO in Figure 1a) and illustrates examples of both 2c–2e^–^ and 3c–2e^–^. Note that the B–C–Co IBO is of almost 3c–2e^–^ nature with an expansion coefficient on the Co atom equal to 0.13. This may result in a reductive opening of the cage from which the cobalt ion is cleaved by a hydrolytic attack. The possibility of cage opening and contraction by a nucleophilic attack was described by Hawthorne et al. already long ago.^52^ After exposure to the air, this is accompanied by color changes to blue or green. Accordingly, the gradual development of green coloration has been observed in our studies. However, it should be noted, no similar products containing *closo*-*commo*-sandwich compounds with mixed 11- and 12-vertex ligands were observed by MS. The degradation corresponds better to dissociation of the complex and a release of the central cobalt ion into solution, which might be supported by the presence of [C_2_B_9_H_12_]^−^ and [N_3_–C_2_B_9_H_11_]^−^ molecular ions observed using HRMS in the partially degraded product (see Figures S1–S3 in the Supporting Information).

Reasonably good conditions for longer-time (weeks) storage are provided by keeping the Me_4_N^+^ salts frozen in an aqueous solution at −33 °C. The structure of disubstituted species **3**^**–**^ corresponds to the *racemic* form, as verified by its NMR spectra and the X-ray structure of the amine obtained after its reduction (see the section below). Despite the low steric crowding assumed for the linear shape of the azide group, the reaction to disubstituted species is stereospecific because neither ion-pair RP-HPLC analysis nor ^11^B NMR spectroscopy has shown any other isomer in the crude product.

**Figure 2:**
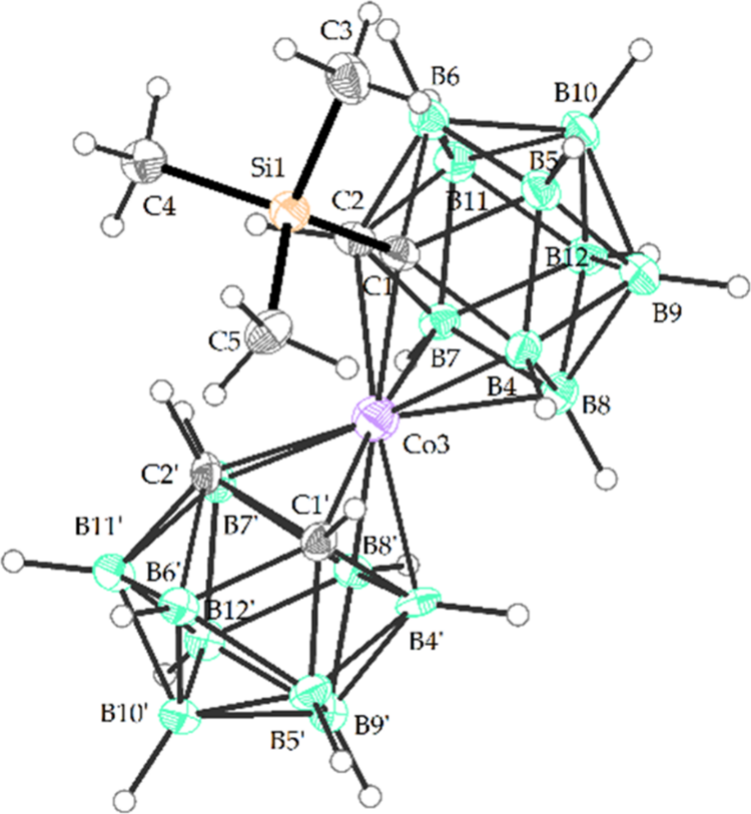
Crystal structure of Me_4_N[(1-Me_3_Si-1,2-C_2_B_9_H_10_)(1′,2′-C_2_B_9_H_11_)-3,3′-Co(III)] (Me_4_N**6**) (ORTEP view, 30% probability level). The cation is omitted for clarity. Selected interatomic distances (Å) and angles (deg): Si1–C1 1.933 (11), C1–C2 1.640(13), C1′-C2′ 1.628(13), Co(3)-C1 2.127(14), Co(3)-C2 2.066(14), Co(3)-C1′ 2.075(13), Co(3)-C2′ 2.089(13), C1–B4 1.730(4), C1–B5 1.712(13), C1–B6 1.731(14), C1′-B4′ 1.700(15), C1′-B5′ 1.683(14), C1′-B6′ 1.705(14), C2–B7 1.720(14), C2–B11 1.686(14), C2–B6 1.714(14), Si1 C1 C2 119.3(6), C3 Si1 C1 110.2(4), C4 Si1 C1 107.9(4), C5 Si1 C1 114.6(4), Si1 C1 Co3 119.2(4), Si1 C1 B4 128.9(6), Si1 C1 B5 113.9(6), Si1 C1 B6 106.1(6), C3 Si1 C1 110.2(4), C4 Si1 C1 107.9(4), C5 Si1 C1 114.6(4), C2′ Co3 C1 101.1(4), C2′ Co3 B8 170.5(4), C2′ Co3 B4 128.8(4).

Similar reaction with Me_3_SiN_3_ led to an irregular pathway resulting in an essentially quantitative conversion to new C(1) monosubstituted Me_3_Si-derivative and the known C(1), C(1′) disubstituted^53^ compound of the formulas [(1-Me_3_Si–1,2-C_2_B_9_H_10_)(1′,2′-C_2_B_9_H_11_)-3,3′-Co(III)]^−^ (**6**^**–**^) and [1,1′-(Me_3_Si–1,2-C_2_B_9_H_10_)_2_-3,3′-Co(III)]^−^ (**7**^**–**^). Both compounds were isolated and characterized using HRMS and NMR, and by X-ray crystallography (Scheme 1). Therefore, the Me_3_Si^δ+^ particle reacts as the preferred electrophile in this reaction, and there is essentially no sign (HPLC and MS analyses) of the expected substitution with the azido group. It should be noted here that trimethylsilyl azide is the reagent widely used for the introduction of azido groups into organic molecules, however, its ambiguous action producing high yields of some trimethylsilylated alcohols or phenols is also known.^54^ The synthesis of the purple *rac*-isomer of disubstituted species **7**^**–**^ available *via* the reaction of a more conventional silylating reagent such as Me_3_SiCl with the lithiated ion **1**^**–**^ had been already reported by Teixidor et al.,^53^ along with the XRD structure of the Me_4_N^+^ salt. The spectral properties and X-ray diffraction data observed in this study have undoubtedly confirmed that the disubstituted ion **7**^**–**^ corresponds to identical species. Likewise in the current case, the reaction is stereospecific, producing the *rac*-isomer only. The yellow-orange monosubstituted derivative **6**^**–**^ has not been isolated before and we present here its full characterization including its sc-XRD structure (see Figures 2 and S17, S18 and Table S7 in the Supporting Information). It seems that the introduction of the first substituent increases the reaction rate, as a result of which the disubstituted anion prevails in the mixture of products, even if the ratio of Cs**1** to BuLi is equal to or below 1.5. The mixture of the parent anion **1**^**–**^, with **6**^**–**^ and **7**^**–**^ in an approximate ratio of 3:20:77 was observed from relative peak areas on an HPLC chromatogram for the reagent’s ratio 1:2.

### Chemical Properties of the Carbon-Substituted Azides

**Scheme 2:**
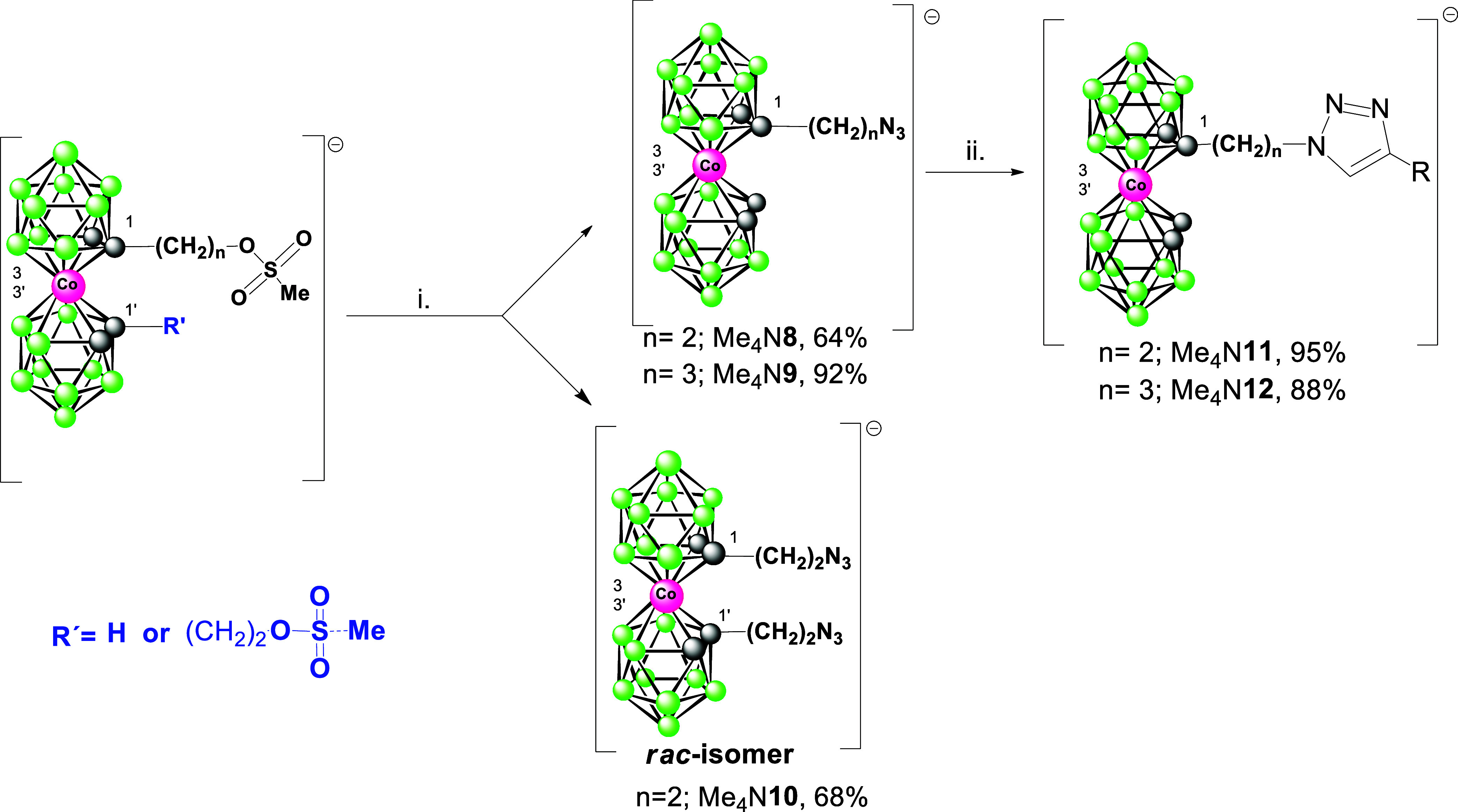
Synthesis of Alkylazide and Triazole Derivatives of the Cobalt Bis(dicarbollide) Ion The starting mesyl esters were prepared according to previously published procedures.^20,29^ Reaction conditions: i. NaN_3_, DMF, r.t., 12 h; ii. Ph-alkyne, CuI, DIPEA, DMF, 40 °C.

The reaction of azides **2**^**–**^ and **3**^**–**^ with norbornadiene as well as the Huisgen–Sharpless dipolar cycloadditions with a series of organic alkynes were tested. Nevertheless, the results have shown a strongly reduced reactivity of the N_3_ groups that are directly attached to the bulky boron-cage anion. For a better understanding of the effect of the boron cage, new azides containing ethyl and propyl linkers, namely [(1-N_3_–C*_n_*H_2*n*_–1′,2′-C_2_B_9_H_10_) (1,2-C_2_B_9_H_11_)-3,3′-Co(III)]^−^ (*n* = 2 and 3, **8**^**–**^, **9**^**–**^) and *rac*-[1,1′-(N_3_–C_2_H_4_–1,2-C_2_B_9_H_10_)_2_-3,3′-Co(III)]^−^ (**10**^–^) were prepared using method starting with known methylsulfonyl esters^29^ and NaN_3_ in DMF and were fully characterized and studied for comparison (Scheme 2). The stability of these compounds in solution and in the solid state is incomparably higher than that of the first members of the series **2**^**–**^ and **3**^**–**^; no decomposition was observed after long (months) of storage under an inert atmosphere at room temperature.

**Figure 3:**
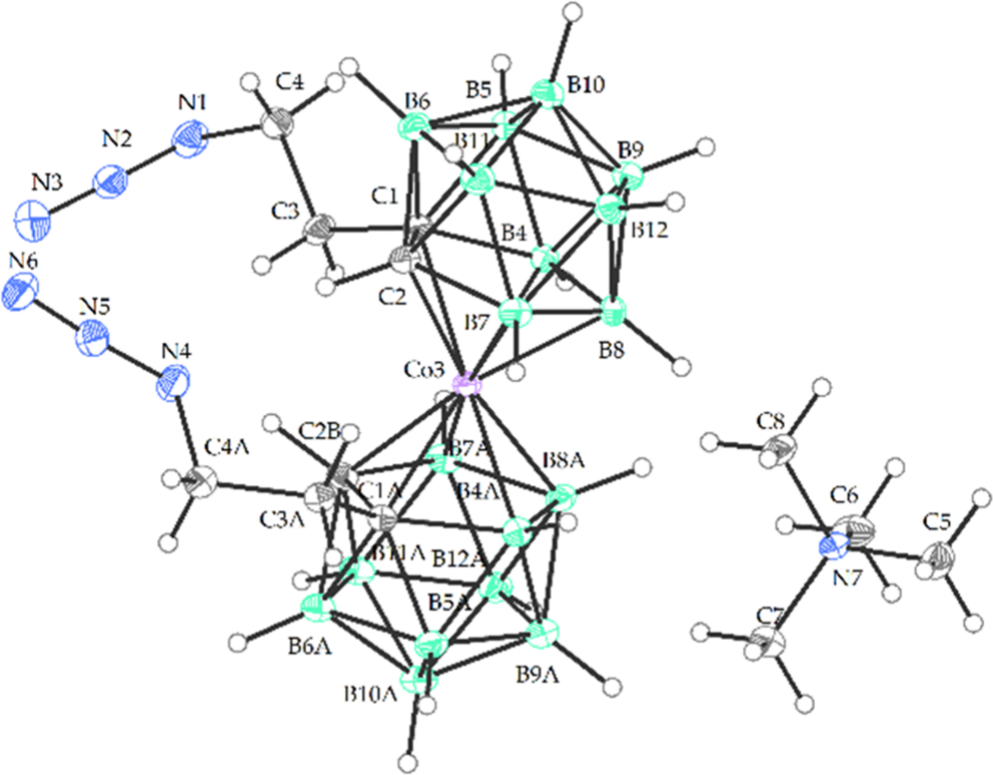
Crystal structure of the *rac*-isomer of Me_4_N10 (ORTEP view, 30% probability level). Selected interatomic distances [Å] and angles [°]: C4 N1 1.483(4), N1 N2 1.241 (4), N2 N3 1.138 (4), C4A N4 1.490 (4), N4 N5 1.243 (4), N5 N6 1.127(4), C3 C4 1.522(4), C1 C3 1.534 (4), C1 C2 1.623(4), C1 B4 1.732(4), C1 B5 1.711(4), C1 B6 1.742(4), C1 Co3 2.116 (3), C2 B11 1.720(4), C2 B7 1.701(4), C2 Co3 2.088(3), Co3 C1A 2.129(3), Co3 B4 2.100(3), Co3 B4A 2.094(3), Co3 B7 2.114(3), Co3 B7A 2.118(3), Co3 B8 2.122(3), Co3 B8A 2.112(3), N1 N2 N3 172.9(3), N1 N2 N3 172.9(3) C2 C1 C3 117.8(2), B4 C1 C3 124.9(2), C3 C4 N1 110.7(2), C4 N1 N2 114.1(2), C2 C1 C3 117.8(2), C3 C4 N1 110.7(2), C4 N1 N2 114.1(3), C4A N4 N5 114.091(3) C3A C4A N4 111.2(3).

**Figure 4:**
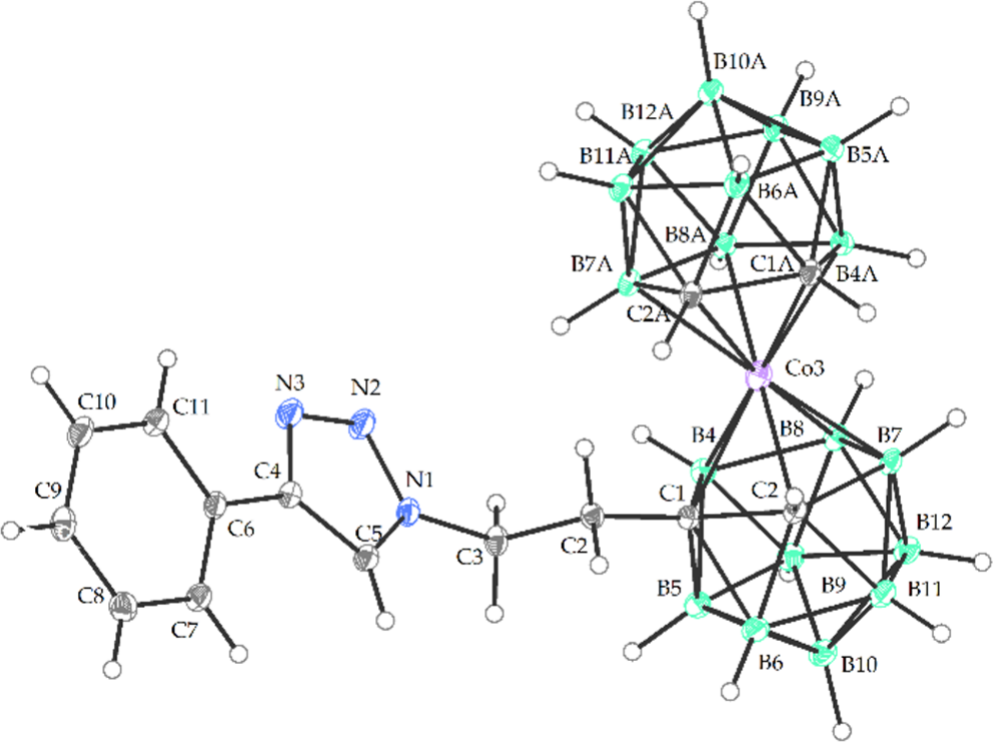
Crystal structure of Me_4_N**11** (ORTEP view, 30% probability level). The Me_4_N^+^ cation and solvent molecule have been omitted for the sake of clarity. Selected interatomic distances [Å] and angles [°]: C3 N1 1.464(2), N1 C5 1.346(2), N1 N2 1.341(2), N2 N3 1.317(2), N3 C4 1.360(2), C4 C6 1.376(2), C2 C1 1.531(2), C5 N1 1.346(2), C1 C2 (cage) 1.619(2), C1 B5 1.710(2), C1 B4 1.731(2), C1 B6 1.754(2), C1 Co3 2.103(1), C2 B11 1.702(2), C2 B6 1.732(2), C2 Co3 2.068(1), Co3 C1A 2.053(1), Co3 B4A 2.091(2), Co3 B4 2.089(2), Co3 B7 2.092(2), Co3 B7A 2.108(2), Co3 B8A 2.118(2), Co3 B8 2.101(2), N1 N2 N3 107.21(12), N2 N1 C3 119.22(12), C3 N1 C5 129.82(12), C4 N3 N2 109.18(12), Co1 C1 C2 112.80(12), B5 C1 C2 122.05(9), B6 C1 C2 109.92(11), C1 Co3 C1A 103.40(), C1 Co1 C2A 134.52(5), B4 C1 C2 126.80(11).

**Figure 5:**
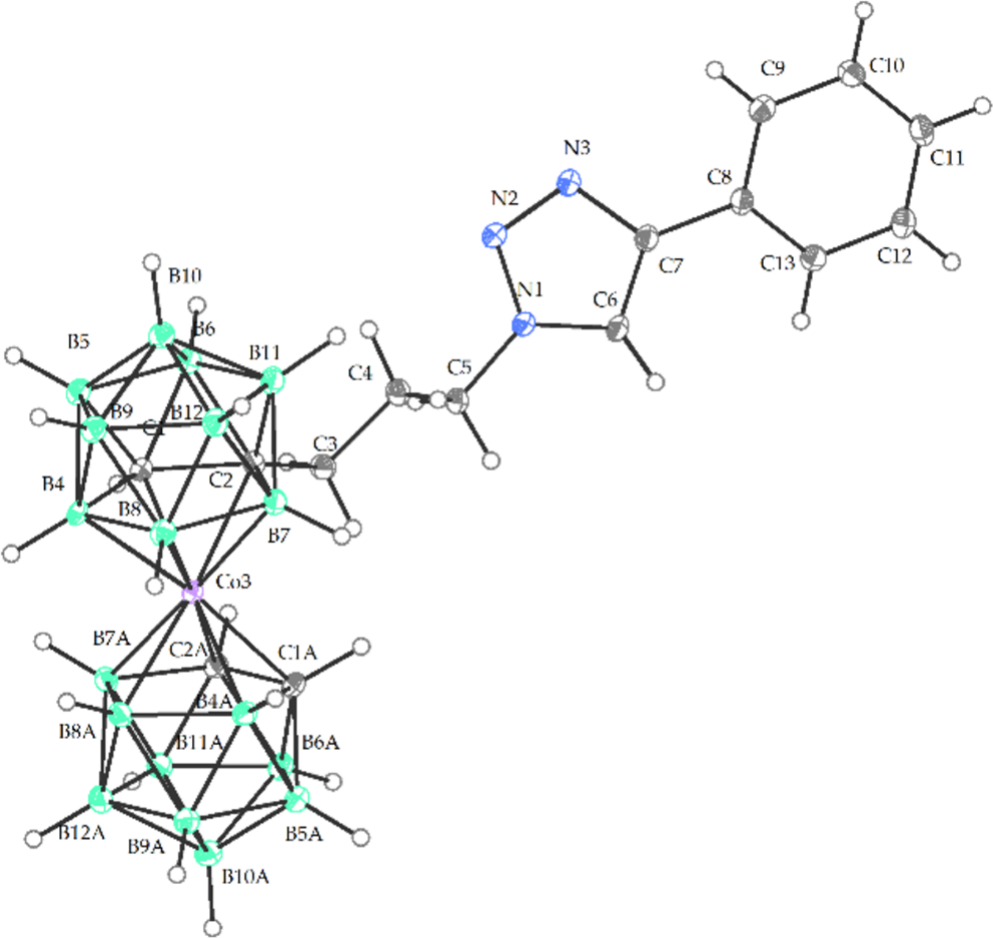
Crystal structure of Me_4_N**12** (ORTEP view, 30% probability level). The Me_4_N^+^ cation is omitted for clarity. C5 N1 1.463(3), N1 C6 1.348(3), N1 N2 1.345(2), N2 N3 1.315(3), N3 C7 1.371(3), C4 C5 1.515(3), C1 C2 1.643(3), C6 N1 1.348(3), C2 C3 1.536(3), C2 B11 1.720(3), C1 B4 1.720(3), C2 B6 1.734(3), C1 Co3 2.045(2), C1 B5 1.703(3), C1 B6 1.721(3), C2 Co3 2.100(2), Co3 C1A 2.060(2), Co3 B4A 2.097(2), Co3 B4 2.083(2), Co3 B7 2.103(2), Co3 B7A 2.102(2), Co3 B8A 2.123(2), Co3 B8 2.110(2), N1 N2 N3 107.38 (17), N2 N1 C5 120.99(17), C5 N1 C6 128.36(18), N1 C6 C7 105.36(18), Co3 C1 C1A 134.75(8), C1 C2 B5 112.13 (16), C3 C2 Co3 112.81(14), C2 Co3 C2A 102.51(8), C1 Co1 C1A 134.75(8), C2 C1 B4 113.47(16).

**Scheme 3:**
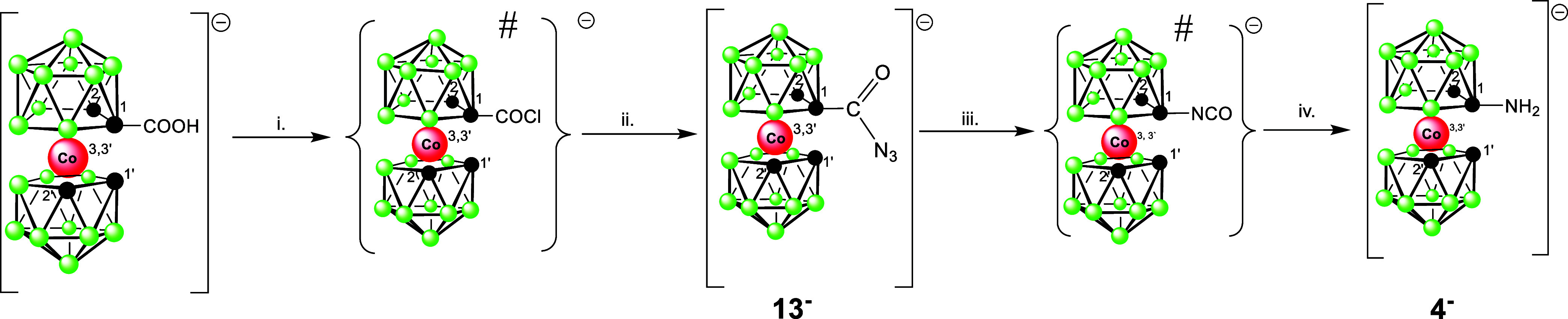
Alternative Synthesis of the Monosubstituted Amine **4**^**–**^*via* Curtius Rearrangement Conditions: i. dioxane, SOCl_2_, 3 h ii. dioxane-water, NaN_3_, 16 h, extraction, RP chromatography; iii. and iv. dioxane, 1 M HCl, 100° C, 12 h, extraction, RP chromatography. # Intermediate products that could not be isolated.

Although the diagnostic reaction (for organic azides) with norbornadiene did not proceed for any starting azide derivative of the ion **1**^**–**^, the copper-catalyzed dipolar cycloaddition reaction, which was tested using phenylacetylene as a probe, provided the expected triazine products, however only when the azido group was attached *via* a longer pendant arm (Scheme 3). The products could be isolated in good yield and were fully characterized. The molecular structures of the compounds **10**^–^, **11**^–^, and **12**^–^ were determined by XRD. The molecular structures of all compounds (see Figures 3, 4, and 5 and S4–S9 in SI) clearly confirmed the presence of the assumed substitution. The rotation angles between the ligands C1–C2–C1A–C2A in **10**^–^, **11**^–^, and **12**^–^ correspond to 24.9, 35.7, and 36.5°, respectively, which is close to *cisoid*-arrangement of ligand planes.

These results underline the possibility of convenient interconnection of the cage with organic fragments and biomolecules *via* orthogonal Huisgen–Sharpless click reactions. Considering the smooth course of the reaction and good yields of anticipated products, these results closely parallel those from a similar dipolar cycloaddition of the propyl azides of the cobalt bis(dicarbollide) ion **1**^**–**^ with a broader scale of alkynes published in our recent communication.^55^ This however covered a different class of derivatives of the ion **1**^**–**^ with restrained geometry locked in the *cisoid*-conformation, due to the presence of an oxygen bridge. On the other hand, the present, as well as previous results, again demonstrate that the electronic and steric shielding effects of the cobalt bis(dicarbollide) cage disfavor the reactions of the groups directly bound to the cage atoms.

### Synthesis and Crystal Structures of Carbon-Substituted Amines

The isolated azides **2**^**–**^ and **3**^**–**^ could not be reduced to the corresponding amines using LiAlH_4_ in ether solvent because of the decomposition to an unidentified black solid material observed in our experiments. However, with an advantage and without the need for drying of the products isolated using RP chromatography, the azides **2**^**–**^ and **3**^**–**^ could be reduced to respective amines [(1-H_2_N–1,2-C_2_B_9_H_10_)(1′,2′-C_2_B_9_H_11_)-3,3′-Co(III)]^−^ (**4**^**–**^) and [1,1′-(H_2_N–1,2-C_2_B_9_H_10_)_2_-3,3′-Co(III)]^−^ (**5**^**–**^) using an excess of NaBH_4_ under CoCl_2_ catalysis in 50% aqueous MeOH (Scheme 1). This reduction adapted from the organic protocol^48^ proceeds within 2 h at room temperature. Apparently, according to ^11^B NMR evidence, an extra hydridic boron remains bound, probably as a BH_3_–NH_2_–C_cage_ moiety, located in a cavity formed between two cluster anions or within a more complex particle. Such shielding from water contributes to hydrolytic stability; therefore, some B–H-containing residues can overpass the first chromatographic purification step and are observed by NMR as impurities in crude amines, together with some borates. Finally, the exoskeletal boron could be removed by repeated acid hydrolysis using 3 M HCl and the released borate could be distilled off with MeOH. The amines **4**^**–**^ and **5**^**–**^ were characterized by HRMS, NMR, and sc-XRD methods. This isolation provided protonated amines in the form of the respective hydrochlorides Me_4_N**4**.HCl and Me_4_N**5**.HCl. In addition, the anionic form of the monosubstituted amine Me_4_N**4** was isolated directly from the reaction mixture using flash LC chromatography on the RP phase and aqueous MeOH as the mobile phase. Both forms of this amine were structurally characterized.

The alternative method of synthesis of the amine **4**^–^ consists in the conversion of the known carboxylic derivative^46^ [(1-HOOC–1,2-C_2_B_9_H_10_)(1′,2′-C_2_B_9_H_11_)-3,3′-Co(III)]^−^ to acyl chloride and the subsequent reaction with sodium azide, which smoothly produces the corresponding acyl azide [(1-N_3_OC–1,2-C_2_B_9_H_10_)(1′,2′-C_2_B_9_H_11_)-3,3′-Co(III)]^−^ (**13**^**–**^). This compound is then converted *in situ* to isocyanate (not isolated) via Curtius rearrangement and finally to the corresponding amine, which is obtained by the acid hydrolysis of this intermediate species upon heating. The product was characterized by ^11^B, ^1^H, and ^13^C NMR and HRMS. However, problems concerning the reproducibility of this procedure were encountered, in particular, when scaling up the synthesis. This was reflected in the formation of side products with the initially used DMF solvent, or different products after thermal treatment, such as amide derivative. The problems have been solved by changing the reaction conditions and the solvent used for acyl azide formation (aqueous dioxane) as well as the conditions for thermal rearrangement. The method requires careful isolation of the acyl azide intermediate by RP chromatography before the thermal rearrangement to an amine. The optimal reaction conditions comprise the formation of acyl chloride from the carboxylic acid using an excess of SOCl_2_ in anhydrous dioxane, evaporation of volatiles, then additions of NaN_3_ dissolved in aqueous dioxane, and heating the reaction mixture at 50 °C for 10 h. These two reaction steps are essentially quantitative. The acyl azide is isolated by flash RP before its thermal rearrangement to an amine in 1 M HCl at 100 °C. The product is isolated by extraction into Et_2_O, evaporation of ether solution, and precipitation from aqueous MeOH with an excess of Me_4_NCl, followed by liquid chromatography on a silica gel column. The compound was characterized by MS and NMR as being identical to Me_4_N**4**.

**Figure 6:**
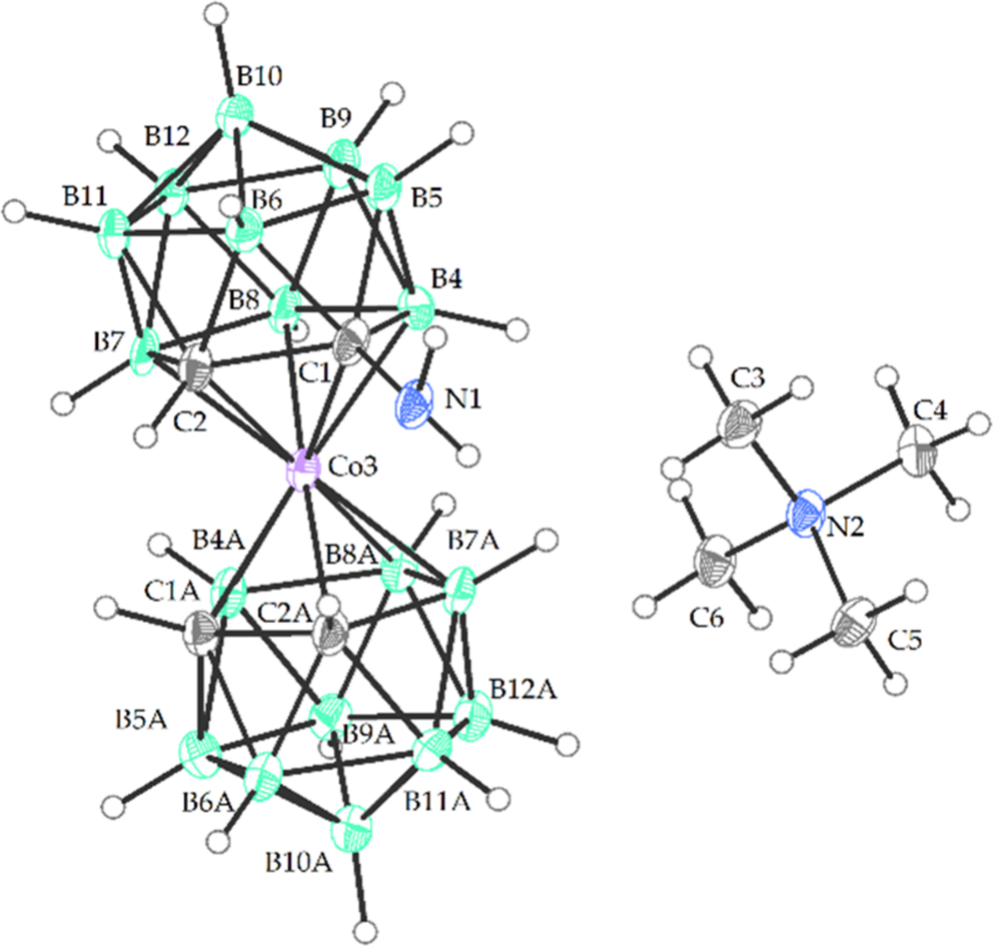
Crystal structure of the anionic form of the amine Me_4_N**4** (ORTEP view, 30% probability level). Selected interatomic distances [Å] and angles [°]: C1 N1 1.436(9), C1 C2 1.653(9), C1A C2A 1.613(9), C1 B4 1.716(11), C1A B4A 1.710(10), C2 B7 1.718(9), C2A B7A 1.705(10), C1 B5 1.701(11), C1 B6 1.727(10), C1A B6A 1.733(10), C2 B6 1.713(10), C2A B6A 1.720(11), C1 Co3 2.077(7), C1A Co3 2.044(7), C2 B11 1.702(10), C2 Co3 2.049(6), C2A Co3 2.0431(7), Co3 B4 2.089(7), Co3 B7 2.102(7), Co3 B7 2.097(4), Co3 B7 2.081(7), Co3 B8 2.105(8), Co3 B8A 2.115(7), B9 B12 1.803(9), C2 C1 N1 116.6(5), N1 C1 B6 110.7(5), N1 C1 B5 119.5(6), N1 C1 B4 126.7(5), C1 Co3 C1A 128.2(3), C2 Co3 C2A 100.8(3), C1 C2 B7 112.4(5), C1 C2 B11 112.1(5), B5 C1 Co3 123.2(5), B4 C1 B6 114.3(5).

**Figure 7:**
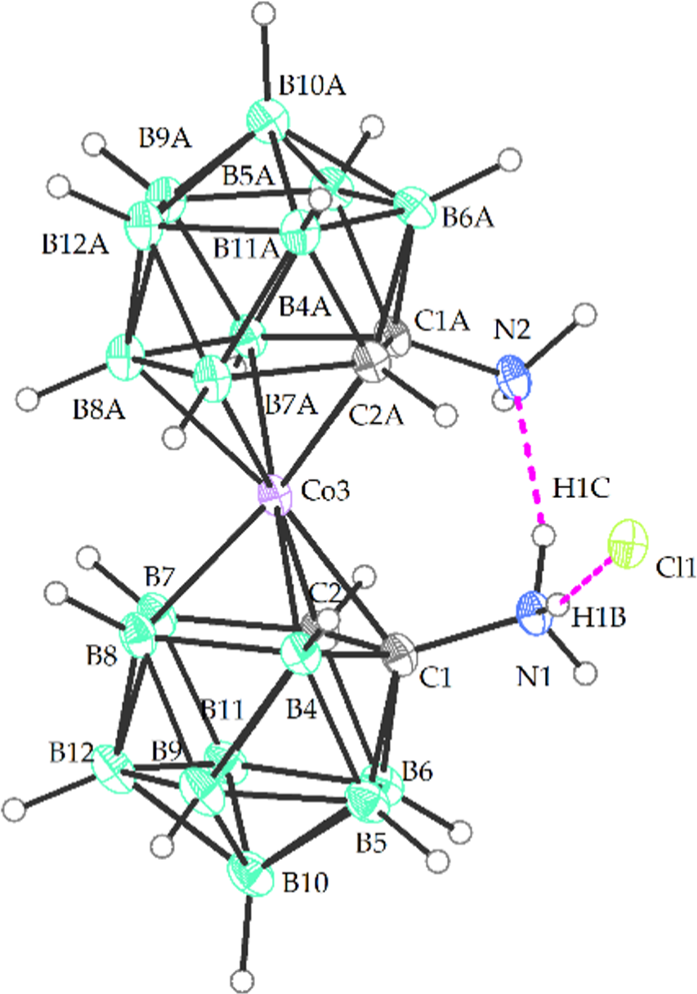
Crystal structure of the protonated form of the diamine Me_4_N**5**.HCl (ORTEP view, 30%). The solvating acetone molecule and the Me_4_N^+^ cation have been omitted for clarity. Selected interatomic distances [Å] and angles [°]: C1 N1 1.466(4), C1A N2 1.442(4), C1 C2 1.621(4), C1A C2A 1.639(4), C1 B4 1.698(5), C1A B4A 1.703(5), C2 B7 1.716(5), C2A C7A 1.714(5), C1 B5 1.696(5),1.711(5), C2 Co3 2.064(3), C1A Co3 2.103(3), Co3 B4 2.102(4), Co3 B7A 2.073(4), Co3 B7 2.093(4), Co3 B8 2.119(4), Co3 B8A 2.108(4), C2 C1 N1 119.1(3), C2A C1A N2 117.6(3), N1 C1 B4 122.0(3), N2 C1A B4A 124.8(3), N1 C1 B6 111.1(3), N2 C1A B6A 111.4(3), N1 C1 B5 115.1(3), N2 C1A B5A 118.4(3), C1 Co3 C1A 104.46(13), C1 Co3 C2A 104.28(13), C2 Co3 C2A 137.17(13), C1 C2 B7 112.1(3), C1A C2A B7A 112.8(2).

The sc-XRD structures of Me_4_N**4** and Me4N**5**.HCl are shown in Figures 6, 7. Both structures confirm the presence of the NH_2_ group covalently bound at the cage carbon atoms C(1) and C(1), C(1′), respectively. For selected interatomic distances and angles, see the Figures 6, 7 captions. The distances in N–C bonds 1.436 and 1.466 Å are closely comparable. These lengths parallel those found in the amino derivatives of the neutral carboranes observed within the interval of 1.430–1.478 Å and are significantly shorter than those previously observed for the boron-substituted ammonium derivative B(8)-NHBn_2_ (1.599 Å), and the interval of distances (1.559–1.633 Å) found in two published structures,^56^ where ammonium or iminium groups are adjacent directly to metallacarborane clusters with different central atoms. Considering the positions of the skeletal carbons, the ligand planes in the structure of Me_4_N**4** (anionic form) adopt the mutual rotation angle C1–C2–C1A-C2A of 30.6° (or 34.6° in the second independent molecule in the racemic crystal), which can be considered to fall into the *cisoid*-arrangement. In contrast, the torsion angle of 38.2° in the solid-state structure of the protonated form of Me_4_N**5**.HCl is slightly larger, close to the *gauche*- arrangement. The skeletal bond distances and angles in **4**^**–**^ and **5**^**–**^ fall within the limits usual for the carbon-substituted cobalt bis(dicarbollide) ion. The Me_4_N^+^ salt of the anion **4**^**–**^ was crystallized in the anionic form of Me_4_N**4** depicted in Figure 6, as well as in the form of the corresponding protonated hydrochloride Me_4_N**4**.HCl (see Tables S4, S5 and Figures S10–S14 in the SI). Interestingly, the two forms crystallize in different crystal systems, corresponding to orthorhombic *Pna*2_1_ and triclinic *P*1̅/*P* space groups. When these two crystal structures are compared, the interatomic distance C1–N1 in the protonated form of Me_4_N**4**.HCl is only slightly increased to 1.48 Å. More significant could be an increase in the torsion angle C1–C2–C1A–C2A to 37.9°, which closely parallels the value observed for the protonated diamine. The structure of Me_4_N**4**.HCl is characterized by the formation of intramolecular and intermolecular hydrogen bonds with the chloride ion N(1)-H^a^.-Cl. The shortest interatomic distance between chloride ions and protons in amino groups H^a^ is 2.47 Å. Correspondingly, the two amino groups in the structure of the protonated diamine Me_4_N**5**.HCl are also involved in the hydrogen bonds between the amino groups and the hydrochloride moiety N(1,2)-H^a^.-Cl and lies within the interval of 2.20–2.57 Å, and it is also possible to observe intramolecular hydrogen bonding N1–H^a^.-N2 at a short distance of 1.80 Å (see Figure 7). Then two and two identical enantiomers are arranged face to face with amino functions and held together via hydrogen bonds, in the racemic crystal unit cell of **5**^–^, which contains eight boron cluster units (see Table S6 and Figures S15 and S16 in the Supporting Information).

### Amine Chirality

**Figure 8:**
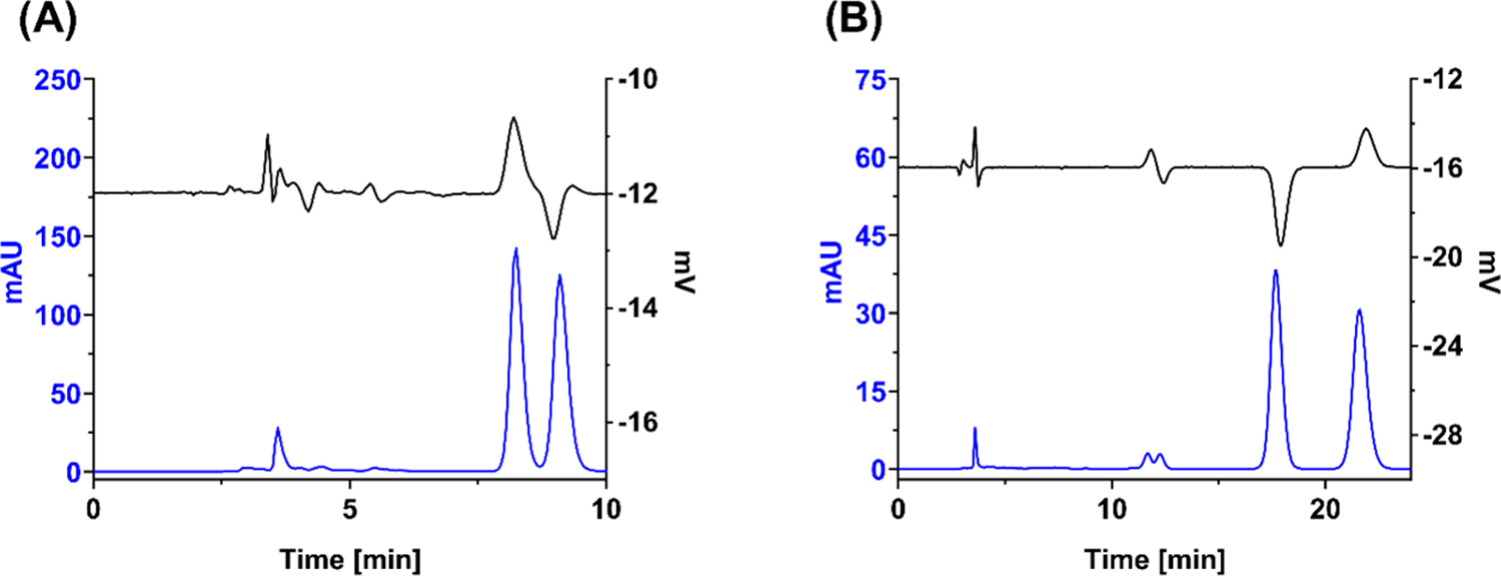
Chiral HPLC separations of the enantiomers of ions **4**^**–**^ (A) and **5**^**–**^ (B). The enantiomers were detected by UV (blue trace) and CD (black trace) detection at wavelengths of 290 and 281 nm, respectively.

As follows from the substitution on C(1) or C(1,1′) sites, the isolated mono- and disubstituted amines **4**^**–**^ and **5**^**–**^ have asymmetric structures corresponding to *C*_*s*_ and *C*_2_ (neglecting the position of the proton in the protonated form) point group symmetry, and display planar and axial chirality, respectively, like their azide precursors **2**^**–**^ and **3**^**–**^. Furthermore, the sc-XRD determined crystal structures show the presence of both enantiomers in racemic crystals. The chirality of the compounds has been confirmed by chiral analytical HPLC separation using Reprosil chiral-β-CD column and tandem UV and circular dichroism (CD) detection. An example of analytical enantioseparation is presented in Figure 8. Hence, the enantiomers of **4**^**–**^ and **5**^**–**^ can potentially serve as versatile chiral structural blocks for the synthesis of metal complexes for enantioselective catalysis or as chiral selectors.

### Acidity of -NH_2_ Groups on the Cobalt Bis(dicarbollide) Cage

It is known from the chemistry of neutral dicarba-*closo*-dodecaboranes that the substitution of boron or carbon sites might significantly alter the acidity of the respective groups.^5^ This has been demonstrated for −SH and −COOH functional groups.^5^ On the other hand, the p*K*_a_ values of amino derivatives have not been reported in the literature, yet. Nevertheless, the proton affinity and the acidity constants of several isomeric NH_2_-derivatives of 1-carba-*closo*-dodecaborate ion [CB_11_H_12_]^−^ substituted at different sites of the cage, namely C(1), B(2), B(7), and B(12) were reported by Finze et al.^47^ The experimental p*K*_a_ values determined using the titration method correspond to the value of 6.0 for the [1-NH_2_–CB_11_H_12_]^−^ derivative and are significantly higher for boron substitution with the highest value over 11.0 observed for the isomer substituted in the antipodal position B(12). The ion [CB_11_H_12_]^−^ belongs to a class of low nucleophilic, low coordinating anions with similar amphiphilic behavior to the bulkier cobalt bis(dicarbollide). The latter ion has however a lower charge to surface area ratio and the electron density at boron and carbon sites is perturbed by the presence of the central cobalt ion.

**Chart 1:**
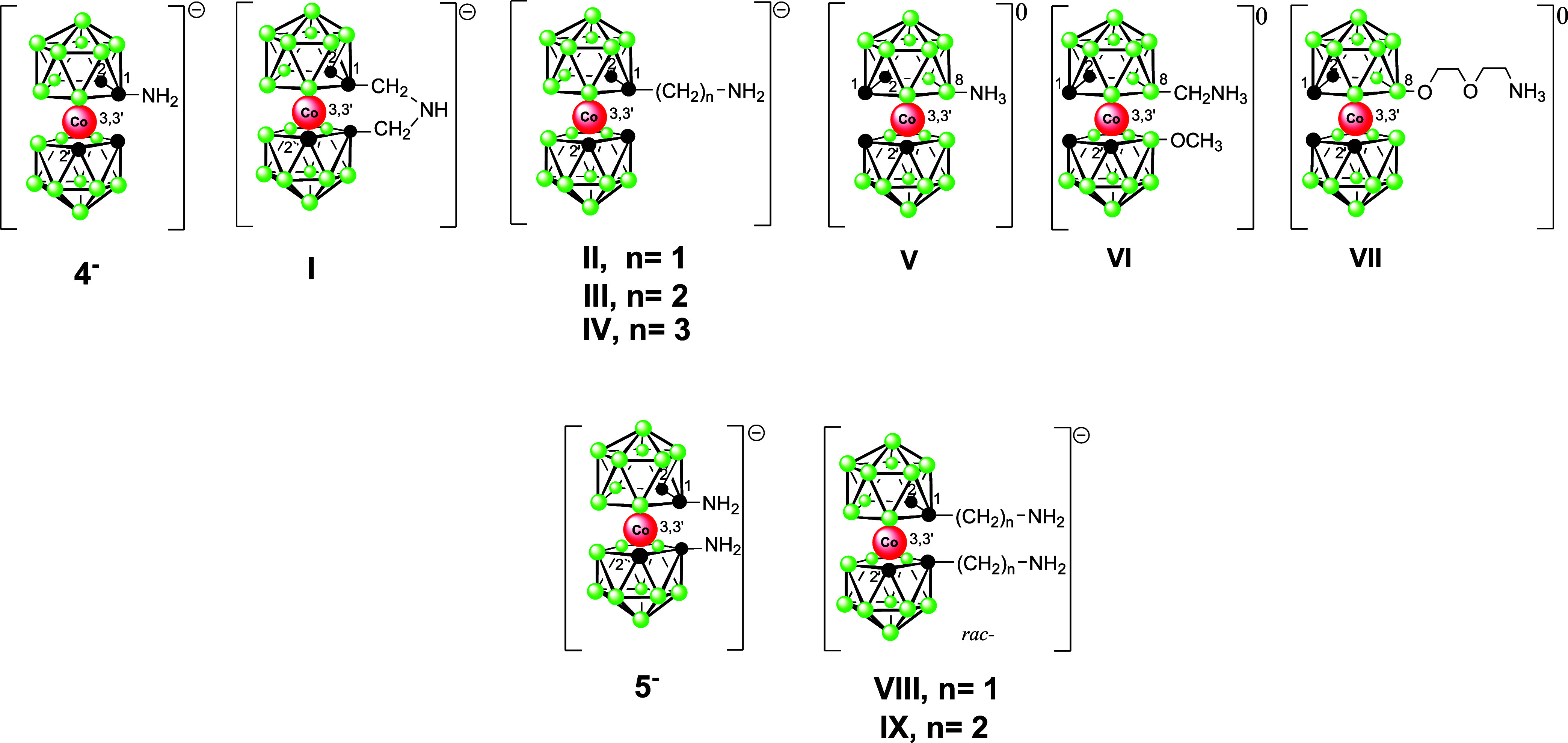
Schematic Structures of the Compounds Studied for the Determination of Proton Dissociation Constants

The thermodynamic acidity constants (p*K*_aH_) of the new amines were determined by capillary electrophoresis and compared with the series of ten synthetically available boron and carbon-substituted amines on the cobalt bis(dicarbollide) cage; see Chart 1 and Table 1. The compounds, which were prepared using methods previously reported in the literature, are numbered in Roman numerals **I** to **X**, for clarity; the references on synthesis are given in Table 1. The mixed acidity constants, p*K*a_Hmix_, of the NH_2_ groups on the cage and their actual ionic mobilities were determined by the nonlinear regression analysis of the dependence of the effective electrophoretic mobilities of the compounds on the pH in the range from 1.9 to more than 12.0, at constant ionic strength (20 mM), and constant temperature (25 °C). The mono- and disubstituted amines with NH_2_- groups on C(1), C(1′) atoms **4**^**–**^ and **5**^**–**^ show low p*K*_a_ values of 2.50 and 4.14, respectively, apparently due to a transfer of the electrons from the nitrogen site to the adjacent electron-withdrawing cage. Therefore, according to experimental data, the proton is easily dissociated, which results in even more acidic properties than in the corresponding carborane derivative [1-H_2_N-CB_11_H_12_]^−^.^47^ The observed p*K*_a_ value for the amine **4**^**–**^ is close to the weakest organic bases such as 3-nitro-aniline (p*K*_a_ of 2.46), lower than those of hydrofluoric acid (p*K*_a_ of 3.17), the majority of organic carboxylic acids and comparable to phosphoric acid (the first dissociation constant of 2.15). It is worth mentioning that the secondary bridging >NH function in **I** is even more acidic with the p*K*_a_ value of 1.9. As expected, the p*K*_a_ values increase with the length of the alkyl pendant group. Relatively low values, 7.71 and 7.31, can still be observed for methylene linkers in **II** and **VIII**, which may indicate that some electronic communication with the cage still proceeds via the methylene unit. The p*K*_a_s observed for compounds with the ethylene pendant group, 9.84 for mono- **III** and 9.33 for the disubstituted compound **IX** are closely comparable to those of ammonia. The corresponding values of the boron-substituted compounds **V** and **VI** exceed 12.0 and thus show distinctive basic properties comparable to or even higher than that of Proton Sponge (p*K*_a_ of 12.1). Even compound **VII** with the NH_2_ group attached via the six-atom diethylene glycol linker on boron site B(8) shows a quite high p*K*_a_ value of 11.44. These experimental observations are in good agreement with the quantum-chemical computations of electron densities, which indicate significantly higher delocalization of electrons from the lone pair on NH_2_ over the cage atoms when the NH_2_ group is located on a carbon.^58^ Preliminary data on the gas-phase proton affinities of NH_2_ groups in a series of isomeric amino derivatives of the cobalt bis(dicarbollide) ion were computed using quantum-chemical computations and based on density functional theory (DFT) methods by Oliva-Enrich et al.^58^ In order to gain deeper insight into this category of amines, we extended the computations of such gas-phase proton affinities for the whole series of compounds listed in Table 1. In analogy with the solid-state structures currently reported, we considered *cisoid*-like arrangements of the C–C–C–C moiety in such computations. Moreover, *cisoid*-like structures offer more intramolecular contacts (hydrogen bonds and dihydrogen bonds) that stabilize the corresponding rotamers. As clearly revealed, the gas-phase proton affinities in such systems in which the organic alkane linker with the terminal amino function is attached to B(8) are higher than in those with functionalized carbon atoms with such organic chains. Indeed, the substituents bonded to boron atoms are of electron-withdrawing nature. The point is that the B(8)-C bonds are of 2c–2e character and the classical electronegativity concept is valid in this case (carbon in the C-chain is more electronegative than the cage C). This was unambiguously confirmed in terms of ^11^B NMR spectroscopy for methylated icosahedral and bicapped-square antiprismatic carboranes, in which methyl-substituted boron atoms are downfield resonated with respect to the parent carboranes.^59^

**Table 1:** Thermodynamic Acidity Constants (p*K*a_H_) of the New Amines Determined by Capillary Electrophoresis and Proton Affinity Values Based on Quantum-Chemical Computations

formula	no.	refs on synthesis	p*K*_a_	proton affinity [kJ mol^–1^], at BP86/AE1 level^a^
[1-NH_2_–1,2-C_2_B_9_H_10_)(1′,2′-C_2_B_9_H_11_)-3,3′-Co(III)].Me_4_N.HCl	**4**^**–**^	-	<2.5	1105
[1,1′-μ-HN-(−CH_2_–1,2-C_2_B_9_H_10_)_2_-3,3′-Co]Me_4_N	**I**	(29)	<1.9	1138
[(1-H_2_NCH_2_–1,2-C_2_B_9_H_10_)(1′,2′-C_2_B_9_H_11_)-3,3′-Co].Me_4_N.HCl	**II**	(29)	7.7	1139
[(1-H_2_N(CH_2_)_2_–1,2-C_2_B_9_H_10_)(1′,2′-C_2_B_9_H_11_)-3,3′-Co].Me_4_N.HCl	**III**	(29)	9.8	1089
[(1-H_2_N(CH_2_)_3_–1,2-C_2_B_9_H_10_)(1′,2′-C_2_B_9_H_11_)-3,3′-Co].Me_4_N.HCl	**IV**	(29)	10.4	1143
[(8-H_3_N–1,2-C_2_B_9_H_10_)(1′,2′-C_2_B_9_H_11_)-3,3′-Co]^0^	**V**	(17)	>12	1282
[(8-H_3_NCH_2_–1,2-C_2_B_9_H_10_)(8′-CH_3_O–1,2′-C_2_B_9_H_10_)-3,3′-Co]	**VI**	(16)	>12	1314
[(8-H_3_NC_2_H_4_OC_2_H_4_O–1,2-C_2_B_9_H_10_)(1′,2′-C_2_B_9_H_11_)-3,3′-Co]^0^	**VII**	(57)	11.4	1208^b^
*rac-*[(1,1′-(NH_2_)_2_–1,2-C_2_B_9_H_10_)_2_-3,3′-Co(III)].Me_4_N.HCl	**5**^**–**^	-	4.1	1193
*rac-*[(1,1′-(H_2_NCH_2_–1,2-C_2_B_9_H_10_)_2_-3,3′-Co].Me_4_N.HCl	**VIII**	(21)	7.3	1139
*rac*-[(1,1′-(H_2_N(CH_2_)_2_–1,2-C_2_B_9_H_10_)_2_-3,3′-Co].Me_4_N.HCl	**IX**	(20)	9.3	1160

a The proton affinity (PA) of an anion or a neutral molecule is the negative of the enthalpy change in the reaction between the chemical species and a proton in the gas phase. The higher the proton affinity (in kJ mol^–1^), the stronger the base and the weaker the conjugate acid.

b Proton affinity is related to the protonation of the oxygen atom adjacent to the B(8) atom of the cage. PA with respect to the nitrogen amounts to 820 kJ mol^–1^ only.

A different character was revealed for **VII**, where the N-based proton affinity of **VII** is compromised by the presence of O, which tends to be protonated, making the N-based PA extremely small. Conceivably, the high stability of the O-protonized form is attributable to the presence of a very strong dihydrogen H(-O)^δ+^...H(-B)^δ−^ bond computed at 1.385 Å.

The experimental and computational results also account for the typical behavior of these two classes of compounds in aqueous solutions. Consequently, compounds with NH_2_ groups directly attached to carbon atoms are anionic in a broad range of pH and induce the cluster properties of acidic sites rather than the behavior of basic groups. In turn, the amino groups on boron atoms are strongly basic, form zwitterionic −NH_3_^+^ species in almost the entire pH range, and can be deprotonated only using strong bases. As follows from the data in Table 1, the substitutions with NH_2_ groups in different skeletal sites along with aliphatic pendant groups offer wide possibilities for scalable acidity properties. This must also be taken into account when considering the particular amine as a building block for tuning interaction with biological targets in drug design.

The easy dissociation of a proton from carbon-bound amino groups has a practical consequence, which can be seen in the solution as well as in the solid-state XRD structures. Even the amines with longer linkers, which are precipitated from the acidic solutions of diluted HCl, remain anionic and show the simultaneous presence of hydrochloride group(s) and a cation for charge compensation in their molecular structures. In this respect, the carbon-bound amines of the cobalt bis(dicarbollide) ion better resemble organic primary amines than the previously known zwitterionic boron cluster compounds with −NH_3_^+^ groups on the boron atom. Here we present the crystal structures of the Me_4_N**4** and Me_4_N**5** determined using sc-XRD along with those that were newly determined for previously reported salts^20,29^ of the alkylamines Me_4_N**VIII** and Me_4_N**III**, which contain methylene and ethylene pendant groups (see Tables S8–S10 and Figures S19–S23 in Supporting Information).

## Conclusions

In this study, synthetic procedures have been developed that lead to the direct synthesis of cobalt bis(dicarbollide) ions containing skeletal carbon to nitrogen atom bonds, namely, azides and amines. Unlike the congeners with an aliphatic linker prepared in parallel, azides containing a direct carbon–nitrogen bond have been shown to have limited stability and significantly reduced reactivity toward multiple bonds. However, the reduction to amino groups has proven to be feasible and provided good yields of the corresponding amines. Along with this, an irregular pathway has been observed for the reaction of the lithiated ion **1**^**–**^ with trimethylsilyl azide, which can be considered a consequence of the bulkiness of the cobalt bis(dicarbollide)(1-) ion and the delocalization of its charge density over high surface area. Consequently, the trimethylsilylium ion, as a bulky and positively charged particle, enters into the reaction more readily as the competing electrophile than the smaller and more polarized azide moiety.

All the isolated compounds feature asymmetric structures, either due to the substitution of the carbon C(1) in monosubstituted compounds or due to disubstitution at positions C(1) and C(1′) adopting the largest mutual separation and thus pertaining to the category of *rac-*isomers. The chirality of the C-substituted amines has been confirmed using chiral HPLC with CD detection and from the XRD structures of racemic crystals. Therefore, the compounds can potentially serve as a platform for asymmetric reactions, chiral selectors, or in the studies of chiral interactions with components of living systems.

Understanding the proton equilibria in boron cluster amines seems essential for a better understanding of the molecular interactions; however, the available data have remained quite limited. Therefore, this report presents a detailed experimental study of the acid–base properties of a wider series of carbon- and boron-substituted amino derivatives of the cobalt bis(dicarbollide) ion. Due to bonding via the cage carbon atoms, the newly synthesized amines **4**^**–**^ and **5**^**–**^ exhibit remarkably high acidity, which is comparable to that of inorganic acids of weak or medium strength such as HF and H_3_PO_4_. It has also been demonstrated that depending on the substitution, the p*K*_a_ values of amines can be increased to the interval of moderate acidities via the presence of an alkyl linker or they can be moved to a strongly basic range applying the B-substitution. The availability of quantitative measures on the acid–base properties of the carbon- and boron-substituted amines might play a considerable role in understanding hydrogen-bonding ability and intermolecular interactions of the cobalt bis(dicarbollide) derivatives with biological targets. Considering the use of amines as building blocks in drug design, this study demonstrates that the currently available selection of the primary amines of ion **1**^**-**^ can already offer solutions for tunable acidity properties over the entire pH range.

## Supporting Information Available

The main file contains crystallographic parameters, details on particular structures, and structure refinement, including the structures of the carbon-substituted alkylamines III and VIII, which are presented in the Supporting Information only; ^11^B, ^1^H, ^13^C, and ^29^Si NMR of the newly prepared compounds, and their HRMS spectra (PDF)

## Abbreviations

CA: carbonic anhydrase
BNCT: boron neutron capture therapy
CCDC: the Cambridge Crystallographic Data Center
DAD: diode-array detection
DME: ethylene glycol dimethyl ether
DMF: dimethylformamide
ESI: electrospray ionization
HPLC: high-performance liquid chromatography
RP: reversed phase
LC: liquid chromatography
NMR: nuclear magnetic resonance
HRMS: high-resolution mass spectrometry
Ph: phenyl
THF: tetrahydrofuran
TLC: thin-layer chromatography
XRD: single-crystal X-ray diffraction
CD: circular dichroism
