# Supplementary material for: Carbon-Substituted Amines of the Cobalt Bis(dicarbollide) Ion: Stereochemistry and Acid–Base Properties
Source: Inorg Chem. 2024 Oct 11;63(43):20600–16. doi: 10.1021/acs.inorgchem.4c03257 (PMC11523243; doi:10.1021/acs.inorgchem.4c03257)
Supplement: Supplementary file 1 — ic4c03257_si_001.pdf [file ic4c03257_si_001.pdf]

Supporting information

Carbon Substituted Amines of the Cobalt Bis(dicarbollide) Ion; Stereochemistry and Acid-Base Properties

Ece Zeynep Tüzün,[1,2‡] Lucia Pazderová,[1‡] Dmytro Bavol,[1] Miroslava Litecká,[1] Drahomir Hnyk,[1] Zdeňka Růžičková,[3] Ondřej Horáček,[4] Radim Kučera[4] and Bohumír Grűner[1*]

[1]Institute of Inorganic Chemistry of the Czech Academy of Sciences, Hlavní 1001, Husinec-Řež 25068, Czech Republic

[2]Department of Inorganic Chemistry, Faculty of Science, Charles University, Hlavova 2030, 128 40 Prague, Czech Republic

[3]Dpt. of Inorganic and General Chemistry, Faculty of Chemical Technology, University of Pardubice, Studentská 95, 532 10 Pardubice, Czech Republic

[4]Faculty of Pharmacy, Charles University, Akademika Heyrovskeho 1203, 500 05 Hradec Králové, Czech Republic

*e-mail: gruner@iic.cas.cz

Table of Contents

Experimental Instrumental Techniques....5

Single Crystal X-Ray Diffraction....5

NMR Spectroscopy....6

HR-MS....6

I. Single Crystal X-Ray Diffraction....10

Crystal data and structure refinement for $Me_4N[(1,1'-N_3C_2H_4-1,2-C_2B_9H_{11})_2-3,3'-Co(III)]$ (Me_4N10)....10

Crystal data and structure refinement for $[Me_4N][(1-(4-Ph-Triazolyl)-C_2H_4-1,2-C_2B_9H_{10})(1',2'-C_2B_9H_{11})-3,3'-Co(III)]$ (Me_4N11)....11

Crystal data and structure refinement for $[Me_4N][(1-(4-Ph-Triazolyl)-C_6H_6-1,2-C_2B_9H_{10})(1',2'-C_2B_9H_{11})-3,3'-Co(III)]$ (Me_4N12)....13

Crystal data and structure refinement for anionic $Me_4N[(1-NH_2-1,2-C_2B_9H_{10})(1',2'-C_2B_9H_{11})-3,3'-Co(III)]$ (Me_4N4) and protonated form $Me_4N[(1-NH_2-1,2-C_2B_9H_{10})(1',2'-C_2B_9H_{11})-3,3'-Co(III)].HCl.1/2MeOH$ ($Me_4N4.HCl$)....15

A. Anionic $Me_4N[(1-NH_2-1,2-C_2B_9H_{10})(1',2'-C_2B_9H_{11})-3,3'-Co(III)]$ (Me_4N4)....15

B. Protonated form [Me_4N][(1-NH_2-1,2-$C_2B_9H_{10}$)(1',2'-$C_2B_9H_{11}$)-3,3'-Co(III)].HCl (Me_4N4.HCl) ... 17

Crystal data and structure refinement for Me_4N[(1,1'-H_2N-1,2-$C_2B_9H_{11}$)$_2$-3,3'-Co(III)] (Me_4N5) ... 20

Crystal data and structure refinement for [Me_4N][(1-H_2N-CH_2-1,2-$C_2B_9H_{10}$)$_2$-3,3'-Co(III)].2HCl (VIII) ... 24

Crystal data and structure refinement for [Me_4N][(1-H_2N-C_2H_4-1,2-$C_2B_9H_{10}$)(1',2'-$C_2B_9H_{11}$)-3,3'-Co(III)].HCl (Me_4NIII) ... 27

II. NMR Spectra ... 30

NMR Spectra of [Me_4N][(1-N_3-1,2-$C_2B_9H_{10}$)(1',2'-$C_2B_9H_{11}$)-3,3'-Co(III)] (Me_4N2) ... 30

NMR Spectra of [Me_4N][(1,1'-N_3-1,2-$C_2B_9H_{10}$)$_2$-3,3'-Co(III)] (Me_4N3) ... 33

NMR Spectra of [Me_4N][(1-H_2N-1,2-$C_2B_9H_{10}$)(1',2'-$C_2B_9H_{11}$)-3,3'-Co(III)] (Me_4N4) ... 36

NMR Spectra of [Me_4N][(1,1'-NH_2-1,2-$C_2B_9H_{10}$)$_2$-3,3'-Co(III)] (Me_4N5) ... 39

NMR Spectra of [Me_4N][(1-Me_3Si-1,2-$C_2B_9H_{10}$)(1',2'-$C_2B_9H_{11}$)-3,3'-Co(III)] (Me_4N6) ... 42

NMR Spectra of [Me_4N][(1,1'-Me_3Si-1,2-$C_2B_9H_{10}$)$_2$-3,3'-Co(III)] (Me_4N7) ... 45

NMR Spectra of [Me_4N][(1-N_3.C_2H_4-1,2-$C_2B_9H_{10}$)(1',2'-$C_2B_9H_{11}$)-3,3'-Co(III)] (Me_4N8) ... 49

NMR Spectra of [Me_4N][(1-N_3-C_3H_6-1,2-$C_2B_9H_{10}$)(1',2'-$C_2B_9H_{11}$)-3,3'-Co(III)] (Me_4N9) ... 52

NMR Spectra of [Me_4N][(1,1'-N_3-C_2H_4-1,2-$C_2B_9H_{10}$)$_2$-3,3'-Co(III)] (Me_4N10) ... 54

NMR Spectra of [Me_4N][(1-(4-Ph-Triazolyl)-C_2H_4-1,2-$C_2B_9H_{10}$)(1',2'-$C_2B_9H_{11}$)-3,3'-Co(III)] (Me_4N11) ... 57

NMR Spectra of [Me_4N][(1-(4-Ph-Triazolyl)-C_3H_6-1,2-$C_2B_9H_{10}$)(1',2'-$C_2B_9H_{11}$)-3,3'-Co(III)] (Me_4N12) ... 60

NMR Spectra of [Me_4N][(1-(CO)N_3-1,2-$C_2B_9H_{10}$)(1',2'-$C_2B_9H_{11}$)-3,3'-Co(III)] (Me_4N13) ... 62

III. HR-MS SPECTRA ... 65

HRMS Spectrum of [(1,2-$C_2B_9H_{11}$)$_2$-3,3'-Co(III)]$^-$ (1^-) ... 65

HRMS Spectrum of [(1-N_3-1,2-$C_2B_9H_{10}$)(1',2'-$C_2B_9H_{11}$)-3,3'-Co(III)]$^-$ (2^-) ... 66

HRMS Spectrum of [(1,1'-N_3-1,2-$C_2B_9H_{10}$)$_2$-3,3'-Co(III)]$^-$ (3^-) ... 67

HRMS Spectrum of [(1-H_2N-1,2-$C_2B_9H_{10}$)(1',2'-$C_2B_9H_{11}$)-3,3'-Co(III)]$^-$ (4^-) ... 68

HRMS Spectrum of [(1,1'-NH_2-1,2-$C_2B_9H_{10}$)$_2$-3,3'-Co(III)]$^-$ (5^-) ... 69

HRMS Spectrum of [(1-Me_3Si-1,2-$C_2B_9H_{10}$)(1',2'-$C_2B_9H_{11}$)-3,3'-Co(III)]$^-$ (6^-) ... 70

HRMS Spectrum of [(1,1'-Me_3Si-1,2-$C_2B_9H_{10}$)$_2$-3,3'-Co(III)]$^-$ (7^-) ... 71

HRMS Spectrum of [(1-N_3-C_2H_4-1,2-$C_2B_9H_{10}$)(1',2'-$C_2B_9H_{11}$)-3,3'-Co(III)]$^-$ (8^-) ... 72

HRMS Spectrum of [(1-N_3-C_3H_6-1,2-$C_2B_9H_{10}$)(1',2'-$C_2B_9H_{11}$)-3,3'-Co(III)]$^-$ (9^-) ... 73

HRMS Spectrum of [(1,1'-N_3-C_2H_4-1,2-$C_2B_9H_{10}$)$_2$-3,3'-Co(III)]$^-$ (10^-) ... 74

HRMS Spectrum of [(1-(4-Ph-Triazolyl)-C_2H_4-1,2-$C_2B_9H_{10}$)(1',2'-$C_2B_9H_{11}$)-3,3'-Co(III)]$^-$ (11^-). 75

HRMS Spectrum of [(1-(4-Ph-Triazolyl)-C_3H_6-1,2-$C_2B_9H_{10}$)(1',2'-$C_2B_9H_{11}$)-3,3'-Co(III)]$^-$ (12^-) . 76

HRMS Spectrum of [(1-(CO)N_3-1,2-$C_2B_9H_{10}$)(1',2'-$C_2B_9H_{11}$)-3,3'-Co(III)]$^-$ (13^-) ... 77

List of Figures

Figure S1. The crystal structure of $[Me_4N][(1,1'-N_3C_2H_4-1,2-C_2B_9H_{11})_2-3,3'-Co(III)]$ (Me_4N**10**). 11
Figure S2. The crystal packing in the structure of $Me_4N[(1,1'-N_3C_2H_4-1,2-C_2B_9H_{11})_2-3,3'-Co(III)]$ (Me_4N**10**) 11
Figure S3. The crystal structure of $[Me_4N][(1-(4-Ph-Triazolyl)-C_3H_6-1,2-C_2B_9H_{10})(1',2'-C_2B_9H_{11})-3,3'-Co(III)].CH_2Cl_2$ 13
Figure S4. The crystal packing in the structure of $[Me_4N][(1-(4-Ph-Triazolyl)-C_2H_4-1,2-C_2B_9H_{10})(1',2'-C_2B_9H_{11})-3,3'-Co(III)]$ 13
Figure S5. The crystal structure of $Me_4N[(1-(4-Ph-Triazolyl)-C_3H_6-1,2-C_2B_9H_{10})(1',2'-C_2B_9H_{11})-3,3'-Co(III)].CH_2Cl_2$ 14
Figure S6. The crystal packing in the structure of $Me_4N[(1-(4-Ph-Triazolyl)-C_3H_6-1,2-C_2B_9H_{10})(1',2'-C_2B_9H_{11})-3,3'-Co(III)].CH_2Cl_2$ 15
Figure S7. The crystal structure of the anionic form of $Me_4N[(1-NH_2-1,2-C_2B_9H_{10})$ $(1',2'-C_2B_9H_{11})-3,3'-Co(III)]$($Me_4N$**4**). 17
Figure S8. The crystal packing in the structure of racemic crystal of $Me_4N[(1-NH_2-1,2-C_2B_9H_{10})$ $(1',2'-C_2B_9H_{11})-3,3'-Co(III)]$ (Me_4N**4**) 17
Figure S9. The crystal structure of $[Me_4N][(1-NH_2-1,2-C_2B_9H_{10})$ $(1',2'-C_2B_9H_{11})-3,3'-Co(III)].HCl$ (Me_4N**4**).HCl. 19
Figure S10. The major (left) and minor (right) components in the structure of $[Me_4N][(1-NH_2-1,2-C_2B_9H_{10})$ $(1',2'-C_2B_9H_{11})-3,3'-Co(III)].HCl$ (Me_4N**4**).HCl 19
Figure S11. Crystal packing in the structure of $[Me_4N][(1-NH_2-1,2-C_2B_9H_{10})$ $(1',2'-C_2B_9H_{11})-3,3'-Co(III)].HCl$ (Me_4N**4**).HCl. 20
Figure S12. The crystal structure of the protonated form of $Me_4N[(1,1'-NH_2-1,2-C_2B_9H_{10})_2-3,3'-Co(III)].HCl$ (Me_4N**5**).HCl 22
Figure S13. The crystal packing in the structure of $Me_4N[(1,1'-NH_2-1,2-C_2B_9H_{10})_2-3,3'-Co(III)].HCl$ (Me_4N**5**).HCl. 22
Figure S14. The crystal structure of the protonated form of $[Me_4N][(1,1'-NH_2-CH_2-1,2-C_2B_9H_{10})_2-3,3'-Co(III)].HCl$ (Me_4N**5**).HCl 26
Figure S15. The formation of hydrogen bonds between the amino groups and the hydrochloride moiety in the structure of $Me_4N[(1-H_2N-CH_2-1,2-C_2B_9H_{10})_2-3,3'-Co(III)].2HCl.2H_2O$ 27
Figure S16. The crystal packing in the structure of $Me_4N[(1-H_2N-CH_2-1,2-C_2B_9H_{11})_2-3,3'-Co(III)].HCl$ 27
Figure S17. The presence of hydrogen bonds between the amino groups and the chloride anion in the structure of $Me_4N[(1-H_2N-C_2H_4-1,2-C_2B_9H_{10})(1',2'-C_2B_9H_{11})-3,3'-Co(III)].HCl$. 29
Figure S18. The crystal packing in the structure of $[Me_4N][(1-H_2N-C_2H_4-1,2-C_2B_9H_{11})(1',2'-C_2B_9H_{12})-3,3'-Co]\cdot HCl$ 29
Figure S19. $^{11}B\{^1H\}$ NMR Spectrum of Me_4N**2** in CD_3OD 30
Figure S20. ^{11}B NMR Spectrum of Me_4N**2** in CD_3OD 30
Figure S21. ^1H NMR Spectrum of Me_4N**2** in CD_3OD 31
Figure S22. $^1H\{^{11}B\}$ NMR Spectrum of Me_4N**2** in CD_3OD 32
Figure S23. $^{13}C\{^1H\}$ NMR Spectrum of Me_4N**2** in CD_3OD 32
Figure S24. $^{11}B\{^1H\}$ NMR Spectrum of Me_4N**3** in CD_3OD 33
Figure S25. ^{11}B NMR Spectrum of Me_4N**3** in CD_3OD 33
Figure S26. ^1H NMR Spectrum of Me_4N**3** in CD_3OD 34
Figure S27. $^1H\{^{11}B\}$ NMR Spectrum of Me_4N**3** in CD_3OD 35
Figure S28. $^{13}C\{^1H\}$ NMR Spectrum of Me_4N**3** in CD_3OD 35
Figure S29. $^{11}B\{^1H\}$ NMR Spectrum of Me_4N**4** in $(CD_3)_2CO$ 36
Figure S30. ^{11}B NMR Spectrum of Me_4N**4** in $(CD_3)_2CO$ 36
Figure S31. ^1H NMR Spectrum of Me_4N**4** in $(CD_3)_2CO$ 37
Figure S32. $^1H\{^{11}B\}$ NMR Spectrum of Me_4N**4** in $(CD_3)_2CO$ 38

Figure S33. $^{13}C\{^1H\}$ NMR Spectrum of Me_4N**4** in $(CD_3)_2CO$......38
Figure S34. $^{11}B\{^1H\}$ NMR Spectrum of Me_4N**5** in $(CD_3)_2CO$......39
Figure S35. ^{11}B NMR Spectrum of Me_4N**5** in $(CD_3)_2CO$......39
Figure S36. ^1H NMR Spectrum of Me_4N**5** in $(CD_3)_2CO$......40
Figure S37. $^1H\{^{11}B\}$ NMR Spectrum of Me_4N**5** in $(CD_3)_2CO$......41
Figure S38. $^{13}C\{^1H\}$ NMR Spectrum of Me_4N**5** in $(CD_3)_2CO$......41
Figure S39. $^{11}B\{^1H\}$ NMR Spectrum of Me_4N**6** in $(CD_3)_2CO$......42
Figure S40. ^{11}B NMR Spectrum of Me_4N**6** in $(CD_3)_2CO$......42
Figure S41. ^1H NMR Spectrum of Me_4N**6** in $(CD_3)_2CO$......43
Figure S42. $^1H\{^{11}B\}$ NMR Spectrum of Me_4N**6** in $(CD_3)_2CO$......43
Figure S43. $^{13}C\{^1H\}$ NMR Spectrum of Me_4N**6** in $(CD_3)_2CO$......44
Figure S44. $^{29}Si\{^1H\}$ NMR Spectrum of Me_4N**6** in $(CD_3)_2CO$......44
Figure S45. $^{11}B\{^1H\}$ NMR Spectrum of Me_4N**7** in $(CD_3)_2CO$......45
Figure S46. ^{11}B NMR Spectrum of Me_4N**7** in $(CD_3)_2CO$......45
Figure S47. ^1H NMR Spectrum of Me_4N**7** in $(CD_3)_2CO$......46
Figure S48. $^1H\{^{11}B\}$ NMR Spectrum of Me_4N**7** in $(CD_3)_2CO$......47
Figure S49. $^{13}C\{^1H\}$ NMR Spectrum of Me_4N**7** in $(CD_3)_2CO$......48
Figure S50. $^{29}Si\{^1H\}$ NMR Spectrum of Me_4N**7** in $(CD_3)_2CO$......48
Figure S51. ^{11}B $\{^1H\}$ NMR spectrum of Me_4N**8** in $(CD_3)_2CO$......49
Figure S52. ^{11}B NMR spectrum of Me_4N**8** in $(CD_3)_2CO$......49
Figure S53. ^1H NMR spectrum of Me_4N**8** in $(CD_3)_2CO$......50
Figure S54. $^1H\{^{11}B\}$ NMR Spectrum of Me_4N**8** in $(CD_3)_2CO$......50
Figure S55. $^{13}C\{^1H\}$ NMR spectrum of Me_4N**8** in $(CD_3)_2CO$......51
Figure S56. ^{11}B $\{^1H\}$ NMR spectrum of Me_4N**9** in $(CD_3)_2CO$......52
Figure S57. ^{11}B NMR spectrum of Me_4N**9** in $(CD_3)_2CO$......52
Figure S58. ^1H NMR spectrum of Me_4N**9** in $(CD_3)_2CO$......53
Figure S59. $^1H\{^{11}B\}$ NMR Spectrum of Me_4N**9** in $(CD_3)_2CO$......53
Figure S60. ^{13}C $\{^1H\}$ NMR spectrum of Me_4N**9** in $(CD_3)_2CO$......54
Figure S61. ^{11}B $\{^1H\}$ NMR spectrum of Me_4N**10** in $(CD_3)_2CO$......54
Figure S62. ^{11}B NMR spectrum of Me_4N**10** in $(CD_3)_2CO$......55
Figure S63. ^1H NMR spectrum of Me_4N**10** in $(CD_3)_2CO$......55
Figure S64. $^1H\{^{11}B\}$ NMR Spectrum of Me_4N**10** in $(CD_3)_2CO$......56
Figure S65. $^{13}C\{^1H\}$ NMR spectrum of Me_4N**10** in $(CD_3)_2CO$......56
Figure S66. $^{11}B\{^1H\}$ NMR spectrum of Me_4N**11** in $(CD_3)_2CO$......57
Figure S67. ^{11}B NMR spectrum of Me_4N**11** in $(CD_3)_2CO$......57
Figure S68. ^1H NMR spectrum of Me_4N**11** in $(CD_3)_2CO$......58
Figure S69. $^1H\{^{11}B\}$ NMR Spectrum of Me_4N**11** in $(CD_3)_2CO$......58
Figure S70. $^{13}C\{^1H\}$ NMR spectrum of Me_4N**11** in $(CD_3)_2CO$......59
Figure S71. $^{11}B\{^1H\}$ NMR spectrum of Me_4N**12** in $(CD_3)_2CO$......60
Figure S72. ^{11}B NMR spectrum of Me_4N**12** in $(CD_3)_2CO$......60
Figure S73. ^1H NMR spectrum of Me_4N**12** in $(CD_3)_2CO$......61
Figure S74. $^1H\{^{11}B\}$ NMR spectrum of Me_4N**12** in $(CD_3)_2CO$......61
Figure S75. $^{13}C\{^1H\}$ NMR spectrum of Me_4N**12** in $(CD_3)_2CO$......62
Figure S76. $^{11}B\{^1H\}$ NMR spectrum of Me_4N**13** in $(CD_3)_2CO$......62
Figure S77. ^{11}B NMR spectrum of Me_4N**13** in $(CD_3)_2CO$......63
Figure S78. ^1H NMR Spectrum of Me_4N**13** in $(CD_3)_2CO$......63
Figure S79. $^1H\{^{11}B\}$ NMR Spectrum of Me_4N**13** in $(CD_3)_2CO$......64
Figure S80. $^{13}C\{^1H\}$ NMR spectrum of Me_4N**13** in $(CD_3)_2CO$......64
Figure S81. HRMS spectrum of $\mathbf{1}^-$ with a calculated isotopic pattern......65
Figure S82. HRMS spectrum of $\mathbf{2}^-$ with a calculated isotopic pattern......66
Figure S83. HRMS spectrum of $\mathbf{3}^-$ with a calculated isotopic pattern......67

Figure S84. HRMS spectrum of **4**$^-$ with a calculated isotopic pattern. 68
Figure S85. HRMS spectrum of **5**$^-$ with a calculated isotopic pattern. 69
Figure S86. HRMS spectrum of **6**$^-$ with a calculated isotopic pattern. 70
Figure S87. HRMS spectrum of **7**$^-$ with a calculated isotopic pattern. 71
Figure S88. HRMS spectrum of **8**$^-$ with a calculated isotopic pattern. 72
Figure S89. HRMS spectrum of **9**$^-$ with a calculated isotopic pattern. 73
Figure S90. HRMS spectrum of **10**$^-$ with a calculated isotopic pattern. 74
Figure S91. HRMS spectrum of **11**$^-$ with a calculated isotopic pattern. 75
Figure S92. HRMS spectrum of **12**$^-$ with a calculated isotopic pattern. 76
Figure S93. HRMS spectrum of **13**$^-$ with a calculated isotopic pattern. 77

Experimental

Instrumental Techniques

Single Crystal X-Ray Diffraction

Data of compounds **4**$^-$, **5**$^-$, and **10**$^-$ to **12**$^-$ and **III** were collected on the Rigaku XtaLAB Synergy S diffractometer equipped with micro-focus with Cu radiation (Cu/Kα λ = 1.54184 Å) and a Hybrid Pixel Array Detector (HyPix-6000HE). An Oxford Cryosystems (Cryostream 800) cooling device was used for data collection and the crystals were kept at 100.00(10) K during data collection. CrysAlisPro software[1] was used for data collection, cell refinement and data reduction and absorption correction. Data were corrected for absorption effects using empirical absorption correction (spherical harmonics), implemented in SCALE3 ABSPACK scaling algorithm and numerical absorption correction based on Gaussian integration over a multifaceted crystal model.[2] The structures of compounds **4**$^-$, **5**$^-$, and **10**$^-$ to **12**$^-$ were solved with the ShelXT[3] structure solution program using Intrinsic Phasing and refined with the SHELXL[4] refinement package using Least Squares minimisation implemented in Olex2.[5] Anisotropic displacement parameters were refined for all non-H atoms. The hydrogen atoms were calculated to idealized positions in the residual electron density map. For crystallographic data and structure refinement see ESI, Tables S1 to S6. Molecular graphics for the structures of **4**$^-$, **5**$^-$, and **10**$^-$ to **12**$^-$ and **III**$^-$ in ESI were generated using DIAMOND software (Version 4.6.8).[6] The crystallographic data of the structural analysis have been deposited with the Cambridge Crystallographic Data Centre, CCDC Nos. 2359154 to 2359160.

The full set of diffraction data for **VIII** was collected at 150(2)K with a Bruker D8-Venture diffractometer equipped with Mo (Mo/K_α radiation; λ = 0.71073 Å) microfocus X-ray (IμS) source, Photon III CMOS detector and Oxford Cryosystems cooling device was used for data collection. The frames were integrated with the Bruker SAINT software package using a narrow-frame algorithm. Data were corrected for absorption effects using the Multi-Scan method (SADABS). Obtained data were treated by XT-version 2019/1 and SHELXL-2019/3 software implemented in APEX4 v2022.2-0 (Bruker AXS) system. Hydrogen atoms were mostly localized on a difference Fourier map, however to ensure uniformity of treatment of crystal, all hydrogens were recalculated into idealized positions (riding model) and assigned temperature factors $H_{iso}(H)$ = 1.2 U_{eq} (pivot atom) or of 1.5U_{eq} (methyl). H atoms in methyl, methylene moieties and hydrogen atoms in B-H or C-H moieties of clusters were placed with C–H or B-H distances of 0.96, 0.97 and 1.1 Å, and refined freely for all N–H and O-H atoms. The crystallographic data of the structural analysis have been deposited with the Cambridge Crystallographic Data Centre, CCDC No. 2374900.

Selected interatomic distances and angles are given in Figs. 2 to 6 captions and the details about the particular structures are provided in Supporting Information. Crystallographic parameters for each

compound given in Tables S1 to S9 and the crystal sctructures and crystal packing are depicted in Figures SF1 to SF18. The deposit nos. in CCDC are given as a reference in Tables S1 to S9.

Supplementary crystallographic data can be obtained free of charge via www.ccdc.cam.ac.uk/data_request/cif, or by emailing data_request@ccdc.cam.ac.uk, or by contacting The Cambridge Crystallographic Data Centre, 12 Union Road, Cambridge CB2 1EZ, UK; fax: + 44 1223 336033.

NMR Spectroscopy

Nuclear magnetic resonance spectroscopy measurements were done on JEOL 600 MHz spectrometer. The spectra of all compounds were measured after dissolution in deuterated-acetone unless otherwise stated. ^{11}B NMR (193 MHz) chemical shifts are given in ppm to high-frequency (low field) to $F_3B.OEt_2$ as the external reference. Residual solvent ^{1}H resonances were used as internal secondary standards. The NMR data are presented in the text as follows: ^{11}B NMR: ^{11}B chemical shifts δ (ppm), multiplicity. ^{1}H NMR (600 MHz) and ^{13}C (151 MHz): chemical shifts δ are given in ppm relative to the standard Me_4Si (0 ppm), coupling constants J(H,H) are in Hz.

HR-MS

High-Resolution Mass Spectrometry (HRMS) spectra were recorded by an Orbitrap ExplorisTM 120 spectrometer equipped with heated electrospray ionization (HESI) in negative mode using nitrogen (5.0 Messer) as a collision gas. For HESI-MS, solutions of concentration approximately 100 ng·mL^{-1} in acetonitrile were introduced by infusion into the ion source from a syringe. Molecular ions $[M]^-$ were detected for all univalent anions as base peaks in the spectra. By comparison, the experimental isotopic distribution in the boron plot of the peaks in the measured spectra corresponded fully to the calculated spectral pattern. The data are presented for the most abundant mass in the boron distribution plot (100%) and for the signal corresponding to the m/z value. Conditions used for the HESI interface: vaporizer temperature 50 °C; N_2 (isolated from air in Genius XE35, Peak Scientific) as a nebulizing sheath gas and auxiliary gas, flow 3.22 L·min^{-1} and 6.12 L·min^{-1}, respectively; ion spray voltage 3500 V; capillary temperature 280 °C and mass range from 100 to 1200.

I. Experimental

Degradation of the azide ion 2^- in solution.

1. Weighed sample of the azide, Me_4N**2** (1.0 mg) was dissolved in 1.0 mL of 50% aqueous MeOH in a screw-cap vial of volume 1.6 mL and the vial was shaken for 5 days. Samples for analysis were taken every 24h and then analyzed using LC-MS system Orbitrap ExplorisTM 120. Analytical results after 2nd and 5th day are depicted in Figure S1. It can be seen, that the compound was almost completely degraded after five days (Figure S1). The main products of degradation identified by HRMS of the column effluent at maximum peak height include ions $[C_2B_9H_{12}]^-$, $[C_2B_9H_{11}N_3]^-$ and $[(1\text{-}NH_2\text{-}1,2\text{-}C_2B_9H_{10})(1',2'\text{-}C_2B_9H_{11})\text{-}3,3'\text{-}Co(III)]^-$ (Figure S2). The composition of some smaller peaks could not be identified using HRMS.
2. The sample of Me_4N**2** (50 mg) was dissolved in 50% aqueous MeOH (15 mL) in a screw-cap vial of volume 20 mL and the solution was stirred for 3 days at room temperature. Then the solution was concentrated on a rotary evaporated and dissolved in minimum volume of MeOH and injected onto LC column Buchi RP (80 g) for flash chromatography and eluted with 50% aqueous MeOH. The first fraction contained the main fraction of the 11-vertex *nido*-species along with some other degradation products. The composition was analyzed by HRMS and the

spectrum is shown in Figure S3. Unfortunately, further attempt to isolate pure compounds from this fractions were unsuccessful.

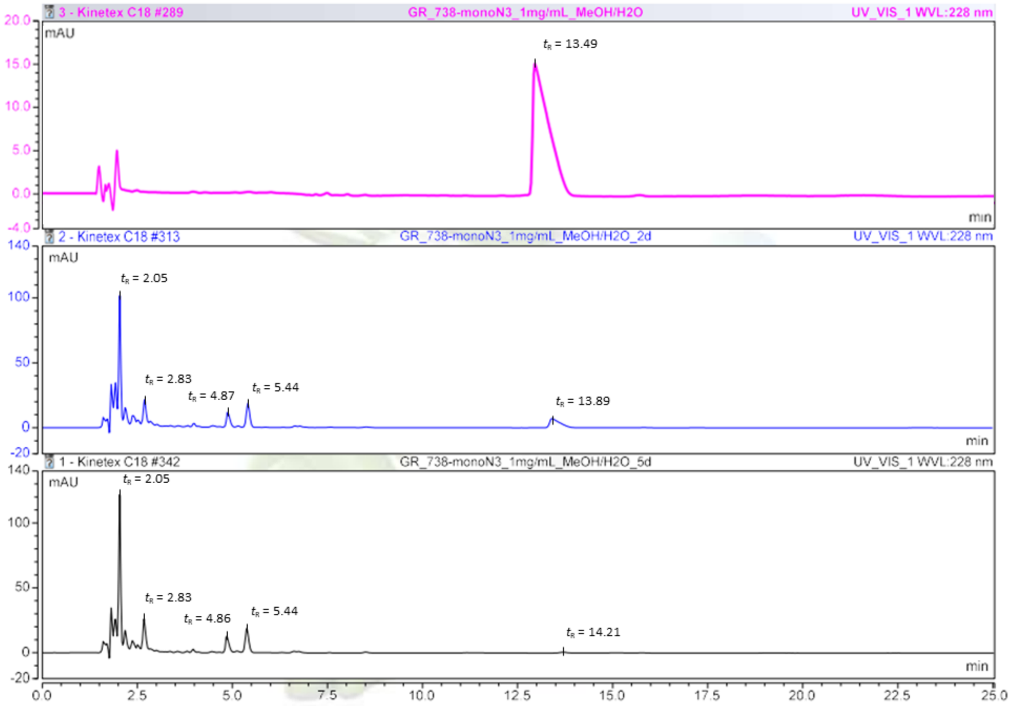

Figure S1. LC−MS chromatograms showing analytical separation of the degradation products in the sample of Me_4N**2** (t_R= 13.49 min.) of concentration 1mg/mL in MeOH/H_2O (1:1, b. v.) after 0, 2, and 5 days. Chromatographic conditions: column Kinetex C18 100 Å (2.6 µm, 150 × 2.1 mm I.D.), mobile phase: 5.0 mM propyl amime acetate (pH: 4.5) in 65% aqueous CH_3CN, flow rate, 0.15 mL/min., detection DAD at 228 nm.

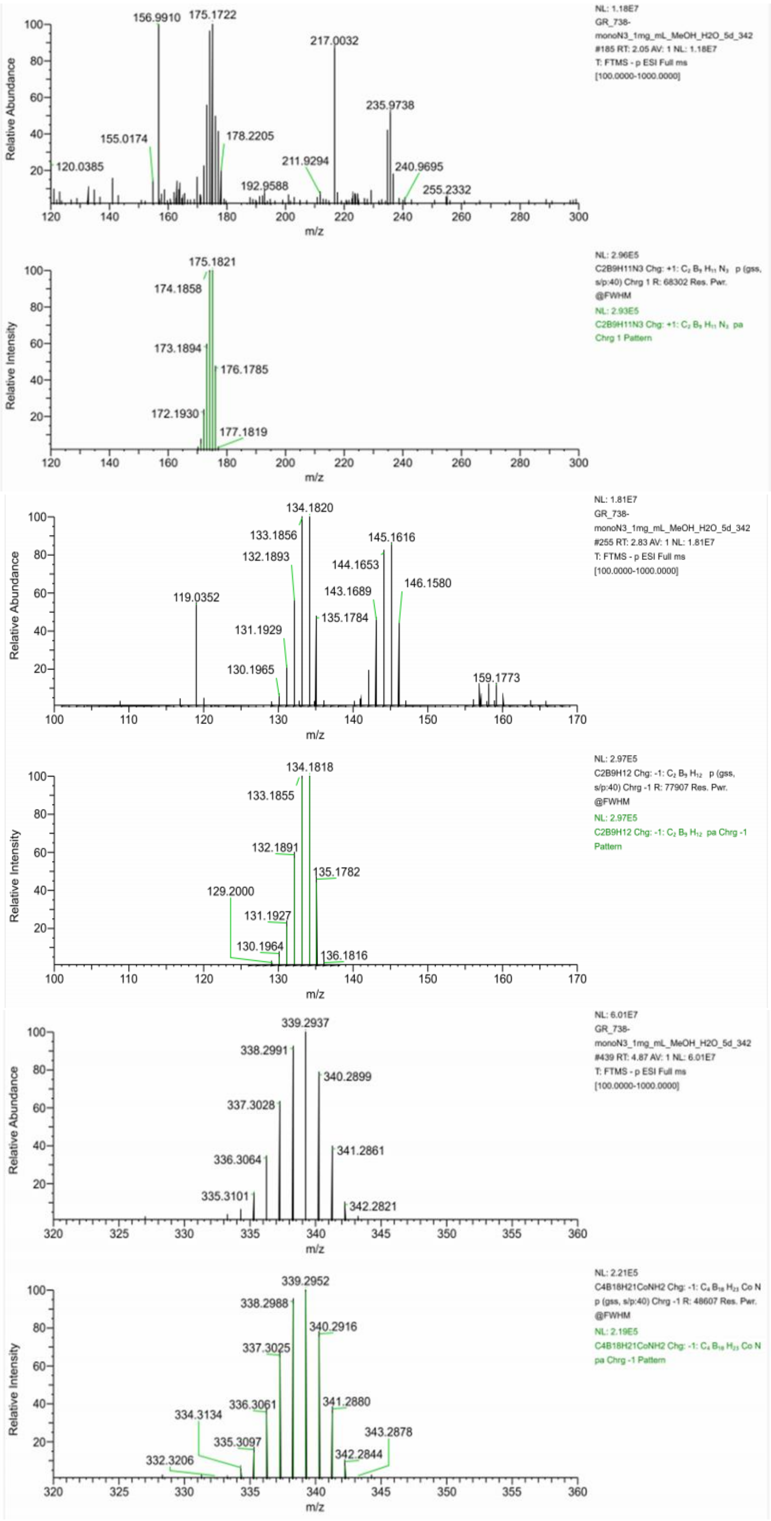

Figure S2. HRMS analysis of the column effluent corresponding to the peaks of main degradation products, from top to bottom: $[C_2B_9H_{11}N_3]^-$ (t_R= 2.05 min.), $[C_2B_9H_{12}]^-$ (t_R= 2.83 min.), and $[(1\text{-}NH_2\text{-}1,2\text{-}C_2B_9H_{10})(1',2'\text{-}C_2B_9H_{11})\text{-}3,3'\text{-}Co(III)]^-$ (t_R= 5.44 min.). The calculated MS spectra of the individual degradation products are shown in green color below each experimentally measured spectrum (in black).

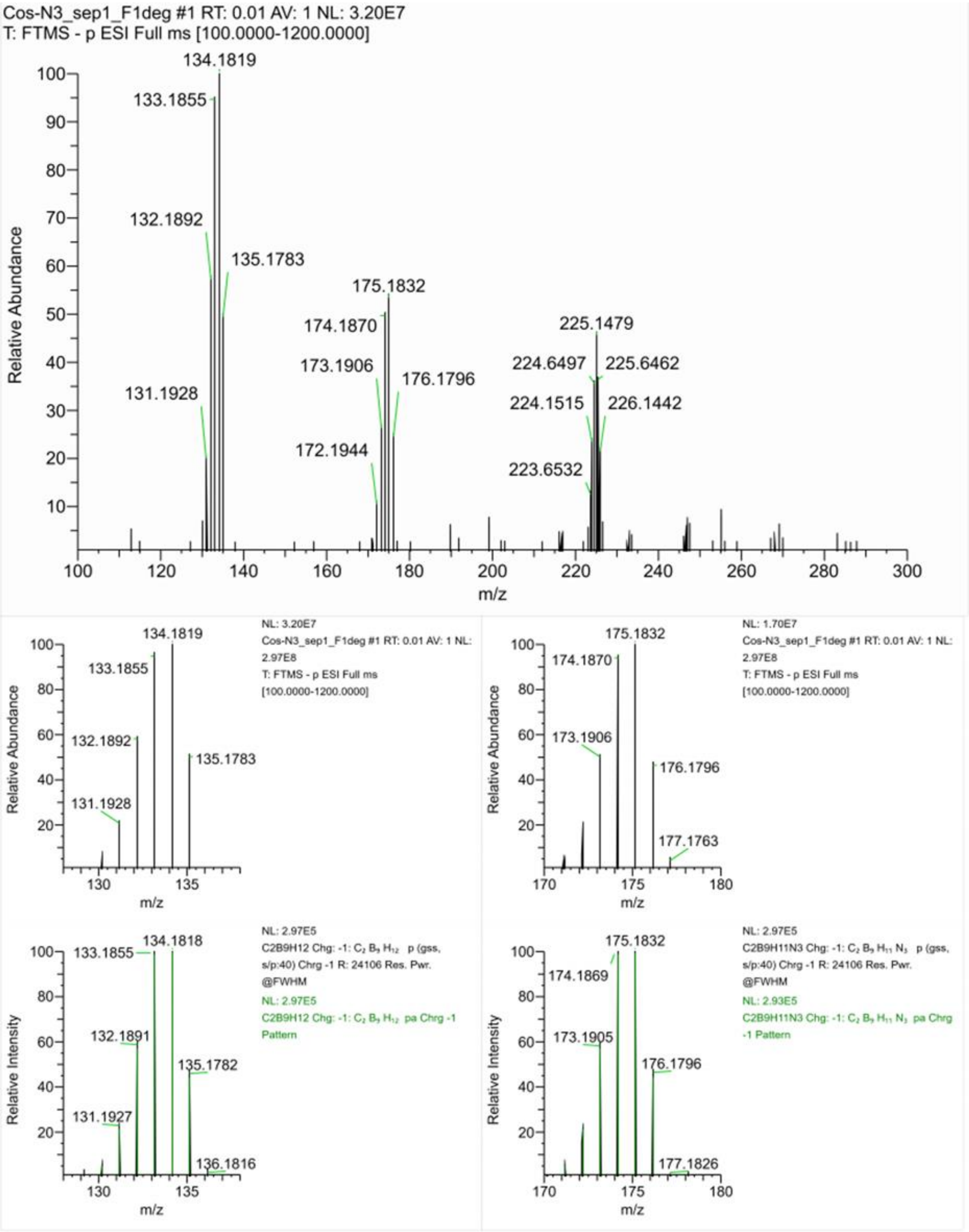

Figure S3. HRMS analysis of the first chromatographic fraction from RP-chromatography of the larger sample of Me_4N**2** (on top). The bottom part shows experimental (black) and calculated boron plot corresponding to the individual molecular peaks of the *nido*-anions $[C_2B_9H_{11}N_3]^-$ and $[C_2B_9H_{12}]^-$; the calculated peaks are shown in green color and given below the each experimentally determined molecular peak.

II. Single Crystal X-Ray Diffraction

Crystal data and structure refinement for $Me_4N[(1,1'-N_3C_2H_4-1,2-C_2B_9H_{11})_2-3,3'-Co(III)]$ (Me_4N**10**)

Experimental

Single crystals of $C_{12}H_{40}B_{18}CoN_7$ were grown from CH_2Cl_2-Hexane. A suitable crystal was selected and measured on a XtaLAB Synergy, Dualflex, HyPix diffractometer, Rigaku. The crystal was kept at 100.00(10) K during data collection. Using Olex2 [1], the structure was solved with the SHELXT [2] structure solution program using Intrinsic Phasing and refined with the SHELXL [3] refinement package using Least Squares minimisation.

Crystal Data for $C_{12}H_{40}B_{18}CoN_7$ (M =536.02 g/mol): triclinic, space group P-1 (no. 2), a = 7.3363(4) Å, b = 12.9448(4) Å, c = 14.5047(5) Å, α = 93.565(3)°, β = 92.462(4)°, γ = 93.027(4)°, V = 1371.40(10) Å^3, Z = 2, T = 100.00(10) K, μ(Cu Kα) = 5.026 mm^{-1}, $Dcalc$ = 1.298 g/cm^3, 16517 reflections measured (6.112° ≤ 2Θ ≤ 154.7°), 5363 unique (R_{int} = 0.0550, R_{sigma} = 0.0537) which were used in all calculations. The final R_1 was 0.0511 (I > 2σ(I)) and wR_2 was 0.1483 (all data).

Table S1 Crystal data and structure refinement for Me_4N**10**.

**Crystal data**	
Identification code	CCDC 2359156
Empirical formula	$C_{12}H_{40}B_{18}CoN_7$
Formula weight	536.02
Temperature/K	100.00(10)
Crystal system	triclinic
Space group	P-1
a/Å	7.3363(4)
b/Å	12.9448(4)
c/Å	14.5047(5)
α/°	93.565(3)
β/°	92.462(4)
γ/°	93.027(4)
Volume/Å^3	1371.40(10)
Z	2
ρ_{calc}g/cm^3	1.298
μ/mm^{-1}	5.026
F(000)	556.0
Crystal size/mm^3	0.22 × 0.15 × 0.1
**Data collection and refinement**	
Radiation	Cu Kα (λ = 1.54184)
2Θ range for data collection/°	6.112 to 154.7
Index ranges	-9 ≤ h ≤ 9, -15 ≤ k ≤ 10, -17 ≤ l ≤ 17
Reflections collected	16517
Independent reflections	5363 [R_{int} = 0.0550, R_{sigma} = 0.0537]
Data/restraints/parameters	5363/0/347
Goodness-of-fit on F^2	1.097
Final R indexes [I>=2σ (I)]	R_1 = 0.0511, wR_2 = 0.1392
Final R indexes [all data]	R_1 = 0.0600, wR_2 = 0.1483
Largest diff. peak/hole / e Å^{-3}	0.50/-0.76

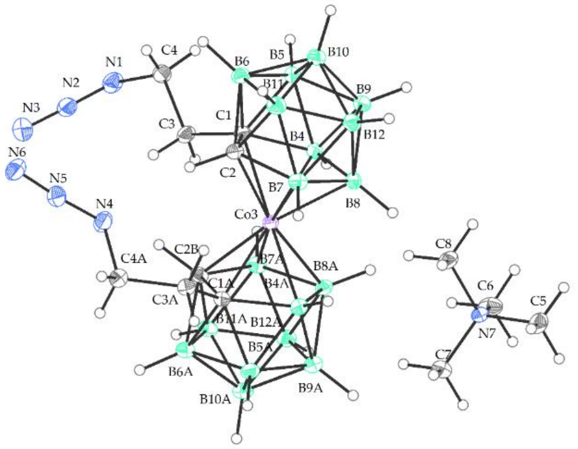

Figure S4. The crystal structure of [Me_4N][(1,1'-$N_3C_2H_4$-1,2-$C_2B_9H_{11}$)$_2$-3,3'-Co(III)] (Me_4N**10**) (ORTEP view, 30% probability level).

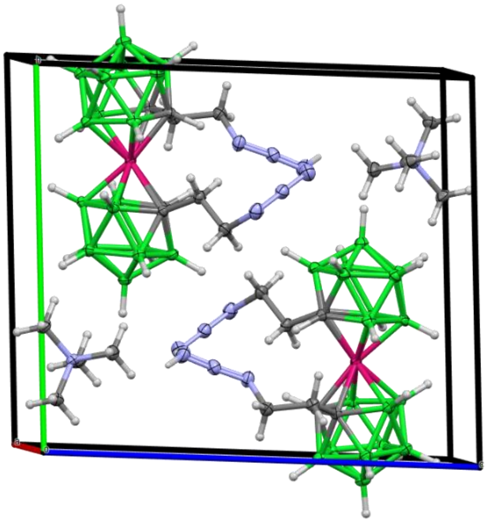

Figure S5. The crystal packing in the structure of Me_4N[(1,1'-$N_3C_2H_4$-1,2-$C_2B_9H_{11}$)$_2$-3,3'-Co(III)] (Me_4N**10**) containing a pair of enantiomers. (ORTEP view, 40% probability level).

Crystal data and structure refinement for [Me_4N][(1-(4-Ph-Triazolyl)-C_2H_4-1,2-$C_2B_9H_{10}$)(1',2'-$C_2B_9H_{11}$)-3,3'-Co(III)] (Me_4N**11**)

Experimental

Single crystals of $C_{20}H_{47}B_{18}Cl_2CoN_4$ were grown from CH_2Cl_2-Hexane. A suitable crystal was selected and measured on a XtaLAB Synergy, Dualflex, HyPix diffractometer, Rigaku. The crystal was kept at 100.00(10) K during data collection. Using Olex2 [1], the structure was solved with the SHELXT [2] structure solution program using Intrinsic Phasing and refined with the SHELXL [3] refinement package using Least Squares minimisation.

Crystal Data for $C_{18}H_{43}B_{18}CoN_4$ (M =569.07 g/mol): monoclinic, space group P2$_1$/c (no. 14), a = 13.14020(10) Å, b = 18.42780(10) Å, c = 12.74000(10) Å, β = 95.6700(10)°, V = 3069.83(4) Å^3, Z = 4, T = 100.00(10) K, μ(Cu Kα) = 4.493 mm^{-1}, $Dcalc$ = 1.231 g/cm^3, 121290 reflections measured (6.76° ≤ 2Θ ≤ 161.148°), 6668 unique (R_{int} = 0.0449, R_{sigma} = 0.0142) which were used in all calculations. The final R_1 was 0.0332 (I > 2σ(I)) and wR_2 was 0.0940 (all data).

Table S2 Crystal data and structure refinement for Me_4N**11**.

**Crystal data**	
Identification code	CCDC 2359158
Empirical formula	$C_{18}H_{43}B_{18}CoN_4$
Formula weight	569.07
Temperature/K	100.00(10)
Crystal system	monoclinic
Space group	$P2_1/c$
a/Å	13.14020(10)
b/Å	18.42780(10)
c/Å	12.74000(10)
α/°	90
β/°	95.6700(10)
γ/°	90
Volume/Å^3	3069.83(4)
Z	4
ρ_{calc}g/cm^3	1.231
μ/mm^{-1}	4.493
F(000)	1184.0
Crystal size/mm^3	0.26 × 0.15 × 0.1

**Data collection and refinement**	
Radiation	Cu Kα (λ = 1.54184)
2Θ range for data collection/°	6.76 to 161.148
Index ranges	$-16 \leq h \leq 16$, $-23 \leq k \leq 23$, $-16 \leq l \leq 16$
Reflections collected	121290
Independent reflections	6668 [R_{int} = 0.0449, R_{sigma} = 0.0142]
Data/restraints/parameters	6668/0/374
Goodness-of-fit on F^2	1.088
Final R indexes [I>=2σ (I)]	R_1 = 0.0332, wR_2 = 0.0931
Final R indexes [all data]	R_1 = 0.0344, wR_2 = 0.0940
Largest diff. peak/hole / e Å^{-3}	0.33/-0.39

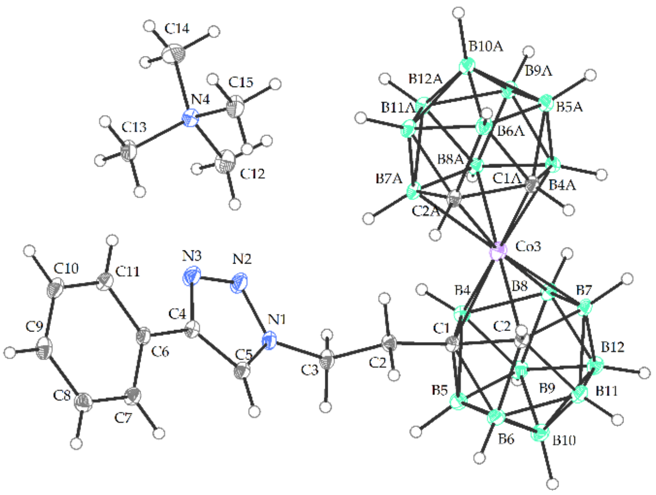

Figure S6. The crystal structure of $[Me_4N][(1\text{-}(4\text{-}Ph\text{-}Triazolyl)\text{-}C_3H_6\text{-}1,2\text{-}C_2B_9H_{10})(1',2'\text{-}C_2B_9H_{11})\text{-}3,3'\text{-}Co(III)].CH_2Cl_2$ (ORTEP view, 30% probability level).

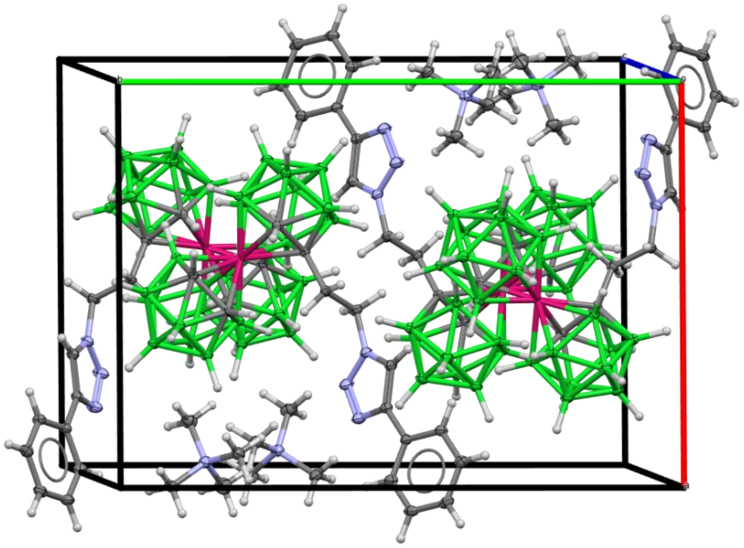

Figure S7. The crystal packing in the structure of $[Me_4N][(1\text{-}(4\text{-}Ph\text{-}Triazolyl)\text{-}C_2H_4\text{-}1,2\text{-}C_2B_9H_{10})(1',2'\text{-}C_2B_9H_{11})\text{-}3,3'\text{-}Co(III)]$ showing the presence of two independent enantiomers (ORTEP view, 40% probability level).

Crystal data and structure refinement for $[Me_4N][(1\text{-}(4\text{-}Ph\text{-}Triazolyl)\text{-}C_6H_6\text{-}1,2\text{-}C_2B_9H_{10})(1',2'\text{-}C_2B_9H_{11})\text{-}3,3'\text{-}Co(III)]$ (Me_4N**12**)

Single crystals of $C_{20}H_{47}B_{18}Cl_2CoN_4$ were grown from CH_2Cl_2-Hexane. A suitable crystal was selected and measured on a XtaLAB Synergy, Dualflex, HyPix diffractometer, Rigaku. The crystal was kept at 100.00(10) K during data collection. Using Olex2 [1], the structure was solved with the SHELXT [2] structure solution program using Intrinsic Phasing and refined with the SHELXL [3] refinement package using Least Squares minimisation.

Crystal Data for $C_{20}H_{47}B_{18}Cl_2CoN_4$ (M =668.02 g/mol): orthorhombic, space group Pbca (no. 61), a = 13.8107(2) Å, b = 16.8363(2) Å, c = 29.2184(5) Å, V = 6793.89(17) Å^3, Z = 8, T = 99.99(10) K, μ(Cu Kα) = 5.552 mm^{-1}, $Dcalc$ = 1.306 g/cm^3, 55240 reflections measured (6.05° ≤ 2Θ ≤ 160.458°), 7296 unique (R_{int} = 0.0519, R_{sigma} = 0.0294) which were used in all calculations. The final R_1 was 0.0431 (I > 2σ(I)) and wR_2 was 0.1203 (all data).

Table S3. Crystal data and structure refinement for Me_4N**12**.

**Crystal data**	
Identification code	CCDC 2359157
Empirical formula	$C_{20}H_{47}B_{18}Cl_2CoN_4$
Formula weight	668.02
Temperature/K	99.99(10)
Crystal system	orthorhombic
Space group	Pbca
a/Å	13.8107(2)
b/Å	16.8363(2)
c/Å	29.2184(5)
α/°	90
β/°	90
γ/°	90
Volume/Å^3	6793.89(17)
Z	8
ρ_{calc}g/cm^3	1.306
μ/mm^{-1}	5.552
F(000)	2768.0
Crystal size/mm^3	0.175 × 0.155 × 0.098

**Data collection and refinement**	
Radiation	Cu Kα (λ = 1.54184)
2Θ range for data collection/°	6.05 to 160.458
Index ranges	$-17 \leq h \leq 17$, $-21 \leq k \leq 21$, $-37 \leq l \leq 35$
Reflections collected	55240
Independent reflections	7296 [R_{int} = 0.0519, R_{sigma} = 0.0294]
Data/restraints/parameters	7296/0/410
Goodness-of-fit on F^2	1.086
Final R indexes [I>=2σ (I)]	R_1 = 0.0431, wR_2 = 0.1169
Final R indexes [all data]	R_1 = 0.0480, wR_2 = 0.1203
Largest diff. peak/hole / e Å^{-3}	0.47/-0.43

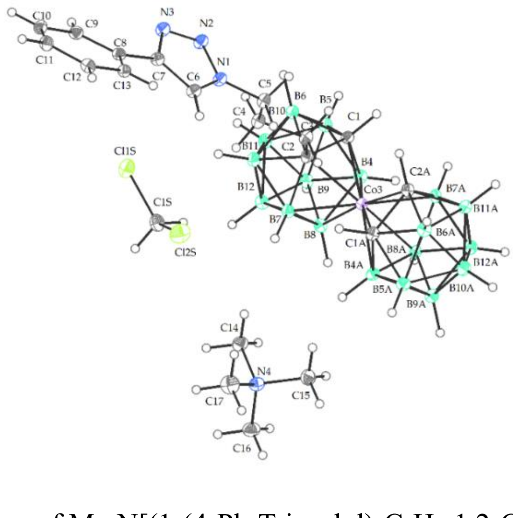

Figure S8. The crystal structure of $Me_4N[(1\text{-}(4\text{-Ph-Triazolyl})\text{-}C_3H_6\text{-}1,2\text{-}C_2B_9H_{10})(1',2'\text{-}C_2B_9H_{11})\text{-}3,3'\text{-}Co(III)].CH_2Cl_2$ (ORTEP view, 30% probability level).

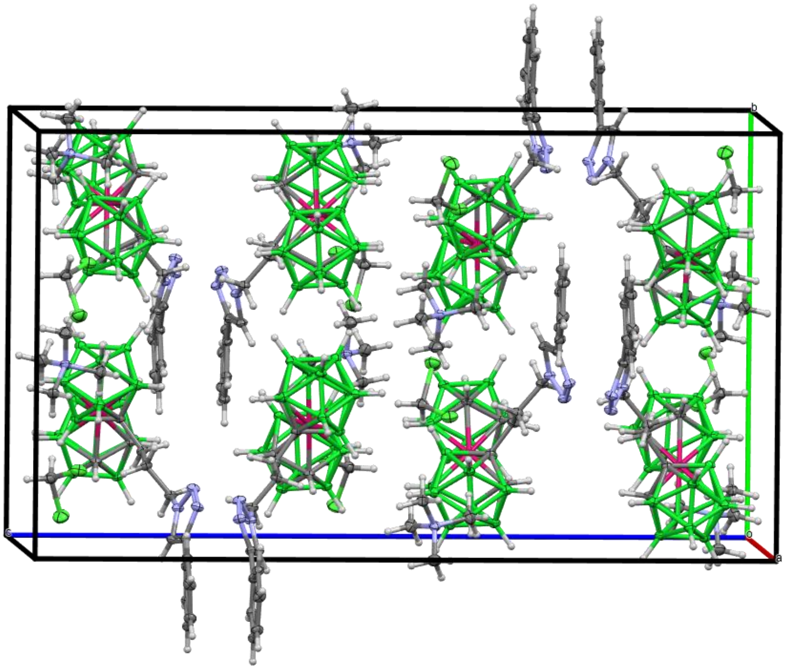

Figure S9. The crystal packing in the structure of $Me_4N[(1\text{-}(4\text{-}Ph\text{-}Triazolyl)\text{-}C_3H_6\text{-}1,2\text{-}C_2B_9H_{10})(1',2'\text{-}C_2B_9H_{11})\text{-}3,3'\text{-}Co(III)].CH_2Cl_2$ showing columns of the cobalt bis(dicarbollide) ions separated by layers of triazine rings. (ORTEP view, 40% probability level).

Crystal data and structure refinement for anionic $Me_4N[(1\text{-}NH_2\text{-}1,2\text{-}C_2B_9H_{10})$ $(1',2'\text{-}C_2B_9H_{11})\text{-}3,3'\text{-}Co(III)]$ (Me_4N4) and protonated form $Me_4N[(1\text{-}NH_2\text{-}1,2\text{-}C_2B_9H_{10})$ $(1',2'\text{-}C_2B_9H_{11})\text{-}3,3'\text{-}Co(III)].HCl.1/2MeOH$ ($Me_4N4.HCl$)

A. Anionic $Me_4N[(1\text{-}NH_2\text{-}1,2\text{-}C_2B_9H_{10})$ $(1',2'\text{-}C_2B_9H_{11})\text{-}3,3'\text{-}Co(III)]$ (Me_4N4)

Experimental

This structure corresponds to anionic, unprotonated form isolated from experiment, when the reaction mixture was not acidified with diluted HCl and the product was isolated only using flash RP-chromatography and precipitated with Me_4NCl in water. Single crystals of $C_8H_{35}B_{18}CoN_2$ were grown from CH_2Cl_2-Hexane. A suitable crystal was selected and measured on a XtaLAB Synergy, Dualflex, HyPix diffractometer, Rigaku. The crystal was kept at 100.00(10) K during data collection. Using Olex2 [1], the structure was solved with the SHELXT [2] structure solution program using Intrinsic Phasing and refined with the SHELXL [3] refinement package using Least Squares minimisation.

Crystal Data for $C_8H_{35}B_{18}CoN_2$ (M =412.89 g/mol): orthorhombic, space group $Pna2_1$ (no. 33), a = 12.0186(7) Å, b = 25.1173(15) Å, c = 7.3524(4) Å, V = 2219.5(2) Å^3, Z = 4, T = 100.00(10) K, μ(Cu Kα) = 5.990 mm^{-1}, $Dcalc$ = 1.236 g/cm^3, 13448 reflections measured (7.038° ≤ 2Θ ≤ 156.248°), 4023 unique (R_{int} = 0.0691, R_{sigma} = 0.0402) which were used in all calculations. The final R_1 was 0.0753 (I > 2σ(I)) and wR_2 was 0.2098 (all data).

Table S4. Crystal data and structure refinement for $Me_4N[(1\text{-}NH_2\text{-}1,2\text{-}C_2B_9H_{10})(1',2'\text{-}C_2B_9H_{11})\text{-}3,3'\text{-}Co(III)]$ (Me_4N**4**).

**Crystal data**	
Identification code	CCDC 2359160
Empirical formula	$C_8H_{35}B_{18}CoN_2$
Formula weight	412.89
Temperature/K	100.00(10)
Crystal system	orthorhombic
Space group	$Pna2_1$
a/Å	12.0186(7)
b/Å	25.1173(15)
c/Å	7.3524(4)
α/°	90
β/°	90
γ/°	90
Volume/Å^3	2219.5(2)
Z	4
ρ_{calc}g/cm^3	1.236
μ/mm^{-1}	5.990
F(000)	856.0
Crystal size/mm^3	0.33 × 0.2 × 0.16

**Data collection and refinement**	
Radiation	Cu Kα (λ = 1.54184)
2Θ range for data collection/°	7.038 to 156.248
Index ranges	$-14 \leq h \leq 10$, $-30 \leq k \leq 31$, $-9 \leq l \leq 7$
Reflections collected	13448
Independent reflections	4023 [R_{int} = 0.0691, R_{sigma} = 0.0402]
Data/restraints/parameters	4023/1/275
Goodness-of-fit on F^2	1.075
Final R indexes [I>=2σ (I)]	R_1 = 0.0753, wR_2 = 0.2005
Final R indexes [all data]	R_1 = 0.0798, wR_2 = 0.2098
Largest diff. peak/hole / e Å^{-3}	1.00/-0.49
Flack parameter	0.081(9)

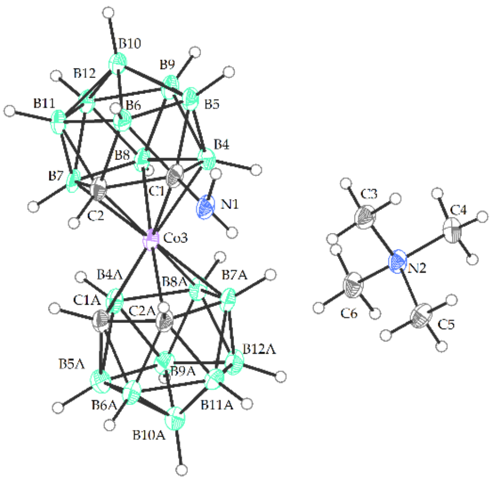

Figure S10. The crystal structure of the anionic form of $Me_4N[(1\text{-}NH_2\text{-}1,2\text{-}C_2B_9H_{10})$ $(1',2'\text{-}C_2B_9H_{11})$-3,3'-Co(III)]($Me_4N$**4**)**.** (ORTEP view, 30% probability level).

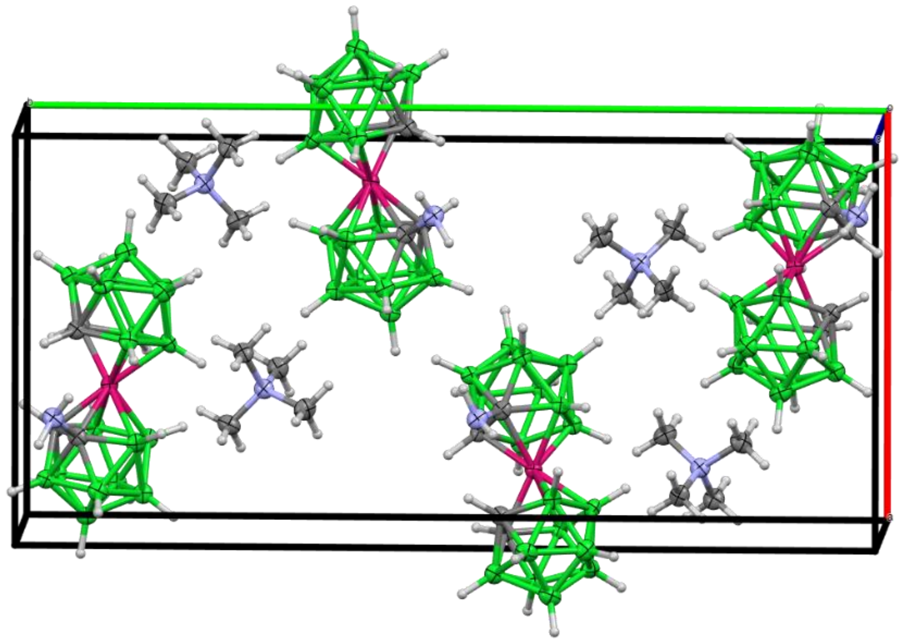

Figure S11. The crystal packing in the structure of racemic crystal of $Me_4N[(1\text{-}NH_2\text{-}1,2\text{-}C_2B_9H_{10})$ $(1',2'\text{-}C_2B_9H_{11})$-3,3'-Co(III)] ($Me_4N$**4**) showing presence of two pairs of enantiomers**.** (ORTEP view, 40% probability level).

B. Protonated form $[Me_4N][(1\text{-}NH_2\text{-}1,2\text{-}C_2B_9H_{10})(1',2'\text{-}C_2B_9H_{11})$-3,3'-Co(III)].HCl ($Me_4N$**4**.HCl)

Experimental

This structure corresponds to protonated form isolated from experiment, when the reaction mixture was acidified with diluted HCl (3M) and the product was isolated by extraction into Et_2O followed with flash RP-chromatography and precipitation with Me_4NCl in 3M HCl in 50% aqueous MeOH. Single crystals corresponding to composition $C_9H_{40}B_{18}ClCoN_2O$ were grown from CH_2Cl_2-Hexane. A suitable crystal was selected and measured on a XtaLAB Synergy, Dualflex, HyPix diffractometer, Rigaku. The crystal was kept at 100.00(10) K during data collection. Using Olex2 [1], the structure was solved with the

SHELXT [2] structure solution program using Intrinsic Phasing and refined with the SHELXL [3] refinement package using Least Squares minimisation.

Crystal Data for $C_9H_{40}B_{18}ClCoN_2O$ (M=481.39 g/mol): triclinic, space group P-1 (no. 2), a = 7.18070(10) Å, b = 12.3450(2) Å, c = 14.7955(2) Å, α = 102.8510(10)°, β = 93.5410(10)°, γ = 97.8660(10)°, V = 1260.89(3) Å^3, Z = 2, T = 99.99(10) K, μ(Cu Kα) = 6.324 mm^{-1}, $Dcalc$ = 1.268 g/cm^3, 38805 reflections measured (6.156° ≤ 2Θ ≤ 160.362°), 5309 unique (R_{int} = 0.0552, R_{sigma} = 0.0289) which were used in all calculations. The final R_1 was 0.0489 (I > 2σ(I)) and wR_2 was 0.1420 (all data).

Table S5. Crystal data and structure refinement for $[Me_4N][(1\text{-}NH_2\text{-}1,2\text{-}C_2B_9H_{10})(1',2'\text{-}C_2B_9H_{11})\text{-}3,3'\text{-}Co(III)].HCl$ (Me_4N**4.**HCl).

**Crystal data**	
Identification code	CCDC 2359155
Empirical formula	$C_9H_{40}B_{18}ClCoN_2O$
Formula weight	481.39
Temperature/K	99.99(10)
Crystal system	triclinic
Space group	P-1
a/Å	7.18070(10)
b/Å	12.3450(2)
c/Å	14.7955(2)
α/°	102.8510(10)
β/°	93.5410(10)
γ/°	97.8660(10)
Volume/Å^3	1260.89(3)
Z	2
ρ_{calc}g/cm^3	1.268
μ/mm^{-1}	6.324
F(000)	500.0
Crystal size/mm^3	0.26 × 0.108 × 0.083

**Data collection and refinement**	
Radiation	Cu Kα (λ = 1.54184)
2Θ range for data collection/°	6.156 to 160.362
Index ranges	-7 ≤ h ≤ 8, -15 ≤ k ≤ 15, -18 ≤ l ≤ 18
Reflections collected	38805
Independent reflections	5309 [R_{int} = 0.0552, R_{sigma} = 0.0289]
Data/restraints/parameters	5309/6/409
Goodness-of-fit on F^2	1.092
Final R indexes [I>=2σ (I)]	R_1 = 0.0489, wR_2 = 0.1389
Final R indexes [all data]	R_1 = 0.0529, wR_2 = 0.1420
Largest diff. peak/hole / e Å^{-3}	0.50/-0.59

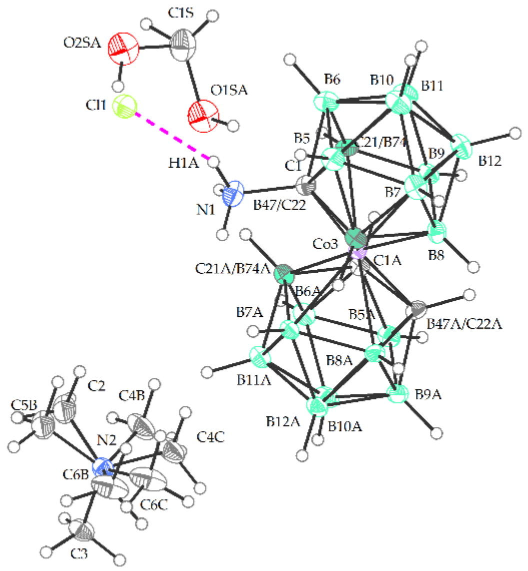

Figure S12. The crystal structure of [Me_4N][(1-NH_2-1,2-$C_2B_9H_{10}$) (1',2'-$C_2B_9H_{11}$)-3,3'-Co(III)].HCl (Me_4N**4**).HCl. The structure contains a disordered molecule of MeOH (ORTEP view, 30% probability level).

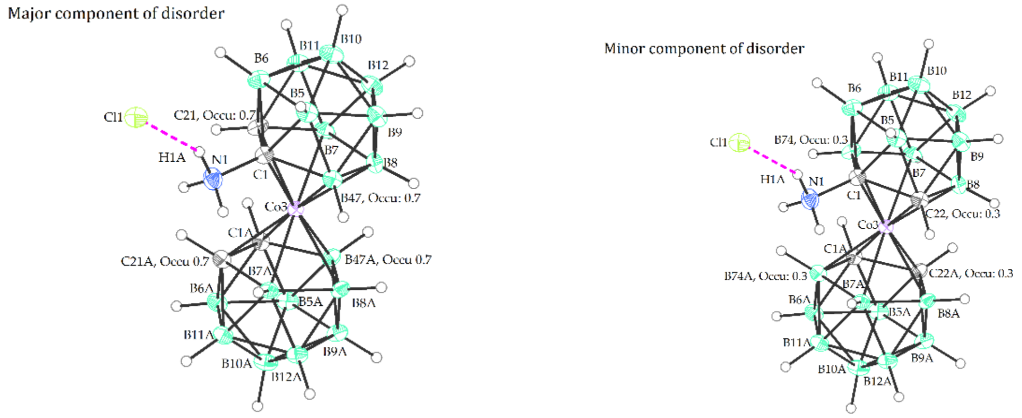

Figure S13. The major (left) and minor (right) components of disorder in the structure of [Me_4N][(1-NH_2-1,2-$C_2B_9H_{10}$) (1',2'-$C_2B_9H_{11}$)-3,3'-Co(III)].HCl (Me_4N**4**).HCl (ORTEP view, 30% probability level).

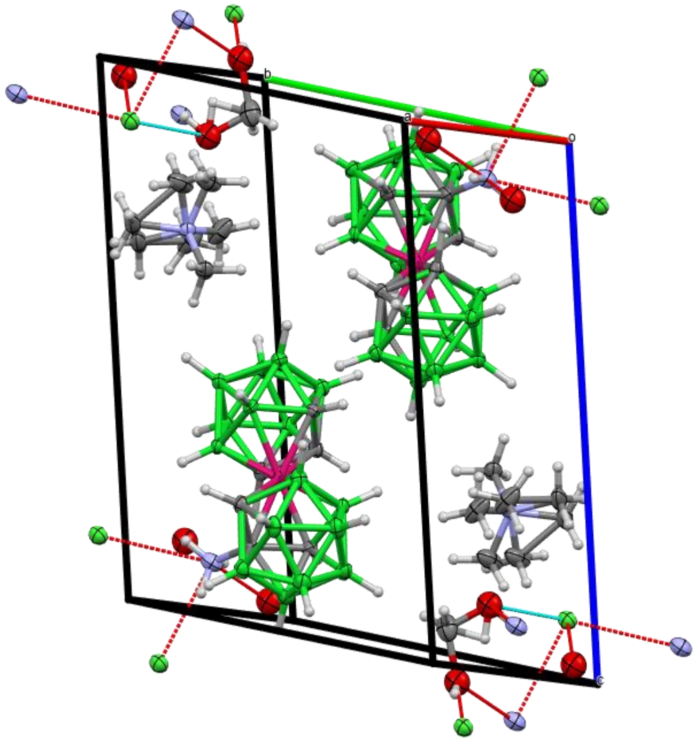

Figure S14. Crystal packing in the structure of $[Me_4N][(1\text{-}NH_2\text{-}1,2\text{-}C_2B_9H_{10})(1',2'\text{-}C_2B_9H_{11})\text{-}3,3'\text{-}Co(III)].HCl$ (Me_4N**4**).HCl. The structure contains both enantiomers in the unit cell, and two disordered Me_4N^+cations along with hydrochloride moieties participating on hydrogen bonding with neighboring NH_2 groups, and a disordered molecule of MeOH (ORTEP view, 40% probability level).

Crystal data and structure refinement for $Me_4N[(1,1'\text{-}H_2N\text{-}1,2\text{-}C_2B_9H_{10})_2\text{-}3,3'\text{-}Co(III)]$ (Me_4N**5**)

Experimental

Single crystals corresponding to the formulation of $C_{11}H_{43}B_{18}ClCoN_{3.03}O$ were grown from CH_2Cl_2-Hexane. A few drops of acetone were added to CH_2Cl_2 for dissolution. A suitable crystal was selected and measured on a XtaLAB Synergy, Dualflex, HyPix diffractometer, Rigaku. The crystal was kept at 100.00(10) K during data collection. Using Olex2 [1], the structure was solved with the SHELXT [2] structure solution program using Intrinsic Phasing and refined with the SHELXL [3] refinement package using Least Squares minimisation.

Crystal Data for $C_{11}H_{43}B_{18}ClCoN_{3.03}O$ (M =522.79 g/mol): monoclinic, space group C2/c (no. 15), a = 34.0535(11) Å, b = 7.03111(18) Å, c = 24.4552(9) Å, β = 103.615(3)°, V = 5690.9(3) Å^3, Z = 8, T = 100.00(10) K, μ(Cu Kα) = 5.656 mm^{-1}, $Dcalc$ = 1.220 g/cm^3, 74727 reflections measured (5.34° ≤ 2Θ ≤ 156.434°), 5854 unique (R_{int} = 0.1277, R_{sigma} = 0.0532) which were used in all calculations. The final R_1 was 0.0614 (I > 2σ(I)) and wR_2 was 0.1578 (all data).

Table S6. Crystal data and structure refinement for $Me_4N[(1,1'-H_2N-1,2-C_2B_9H_{11})_2-3,3'-Co(III)]$

**Crystal data**	
Identification code	CCDC 2359159
Empirical formula	$C_{11}H_{43}B_{18}ClCoN_{3.02}O$
Formula weight	522.79
Temperature/K	100.00(10)
Crystal system	monoclinic
Space group	C2/c
a/Å	34.0535(11)
b/Å	7.03111(18)
c/Å	24.4552(9)
α/°	90
β/°	103.615(3)
γ/°	90
Volume/Å^3	5690.9(3)
Z	8
ρ_{calc}g/cm^3	1.220
μ/mm^{-1}	5.656
F(000)	2177.0
Crystal size/mm^3	0.395 × 0.043 × 0.033

**Data collection and refinement**	
Radiation	Cu Kα (λ = 1.54184)
2Θ range for data collection/°	5.34 to 156.434
Index ranges	$-42 \leq h \leq 41$, $-8 \leq k \leq 8$, $-29 \leq l \leq 30$
Reflections collected	74727
Independent reflections	5854 [R_{int} = 0.1277, R_{sigma} = 0.0532]
Data/restraints/parameters	5854/0/332
Goodness-of-fit on F^2	1.049
Final R indexes [I>=2σ (I)]	R_1 = 0.0614, wR_2 = 0.1461
Final R indexes [all data]	R_1 = 0.0831, wR_2 = 0.1578
Largest diff. peak/hole / e Å^{-3}	0.60/-0.47

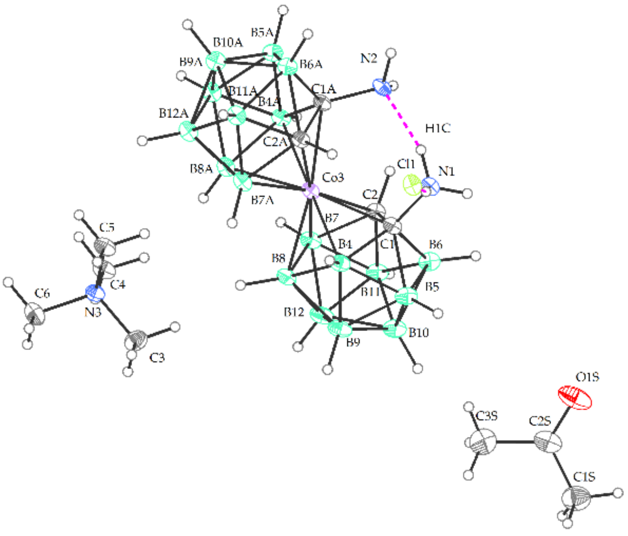

Figure S15. The crystal structure of the protonated form of $Me_4N[(1,1'\text{-}NH_2\text{-}1,2\text{-}C_2B_9H_{10})_2\text{-}3,3'\text{-}Co(III)].HCl$ (Me_4N**5**).HCl (ORTEP view, 30% probability level). The structure contains a hydrochloride moiety and a molecule of acetone used for dissolution.

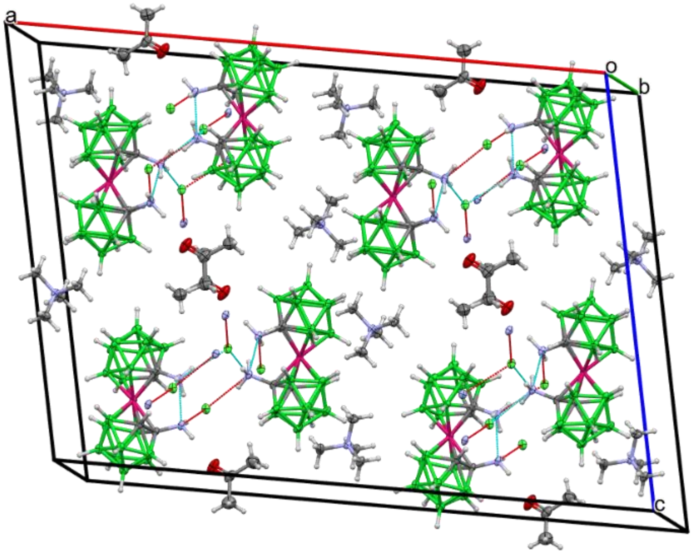

Figure S16. The crystal packing in the structure of $Me_4N[(1,1'\text{-}NH_2\text{-}1,2\text{-}C_2B_9H_{10})_2\text{-}3,3'\text{-}Co(III)].HCl$ (Me_4N**5**).HCl, which contains 8 boron clusters along with charge compensating Me_4N^+ ions and acetone molecules. The racemic crystal contains both enantiomers in the unit cell. (ORTEP view, 40% probability level).

Crystal data and structure refinement for $Me_4N[(1\text{-}Me_3Si\text{-}1,2\text{-}C_2B_9H_{10})(1',2'\text{-}C_2B_9H_{11})\text{-}3,3'\text{-}Co(III)]$ (Me_4N**6**)

Experimental

Single crystals corresponding to the formulation of $C_{11}H_{42}B_{18}CoNSi$ were grown from CH_2Cl_2-Hexane. A few drops of acetone were added to CH_2Cl_2 for dissolution. A suitable crystal was selected

and measured on a XtaLAB Synergy, Dualflex, HyPix diffractometer, Rigaku. The crystal was kept at 100.00(10) K during data collection. Using Olex2 [1], the structure was solved with the SHELXT [2] structure solution program using Intrinsic Phasing and refined with the SHELXL [3] refinement package using Least Squares minimisation.

Crystal Data for $C_{11}H_{42}B_{18}CoNSi$ (M =470.05 g/mol): orthorhombic, space group $Pca2_1$ (no. 29), a = 31.698(4) Å, b = 7.0938(7) Å, c = 11.6377(13) Å, V = 2616.9(5) Å^3, Z = 4, T = 99.99(11) K, μ(Cu Kα) = 5.550 mm^{-1}, $Dcalc$ = 1.193 g/cm^3, 25530 reflections measured (9.428° ≤ 2Θ ≤ 155.954°), 5037 unique (R_{int} = 0.1385, R_{sigma} = 0.1059) which were used in all calculations. The final R_1 was 0.0647 (I > 2σ(I)) and wR_2 was 0.1723 (all data).

Table S7. Crystal data and structure refinement for $Me_4N[(1\text{-}Me_3Si\text{-}1,2\text{-}C_2B_9H_{10})(1',2'\text{-}C_2B_9H_{11})\text{-}3,3'\text{-}Co(III)]$

**Crystal data**	
Identification code	CCDC 2375075
Empirical formula	$C_{11}H_{42}B_{18}CoNSi$
Formula weight	470.05
Temperature/K	99.99(11)
Crystal system	orthorhombic
Space group	$Pca2_1$
a/Å	31.698(4)
b/Å	7.0938(7)
c/Å	11.6377(13)
α/°	90
β/°	90
γ/°	90
Volume/Å^3	2616.9(5)
Z	4
ρ_{calc}g/cm^3	1.193
μ/mm^{-1}	5.550
F(000)	984.0
Crystal size/mm^3	0.06 × 0.041 × 0.028

**Data collection and refinement**	
Radiation	Cu Kα (λ = 1.54184)
2Θ range for data collection/°	9.428 to 155.954
Index ranges	-40 ≤ h ≤ 39, -8 ≤ k ≤ 8, -14 ≤ l ≤ 14
Reflections collected	25530
Independent reflections	5037 [R_{int} = 0.1385, R_{sigma} = 0.1059]
Data/restraints/parameters	5037/1/297
Goodness-of-fit on F^2	1.046
Final R indexes [I>=2σ (I)]	R_1 = 0.0647, wR_2 = 0.1410
Final R indexes [all data]	R_1 = 0.1056, wR_2 = 0.1723
Largest diff. peak/hole / e Å^{-3}	0.36/-0.70
Flack parameter	0.353(11)

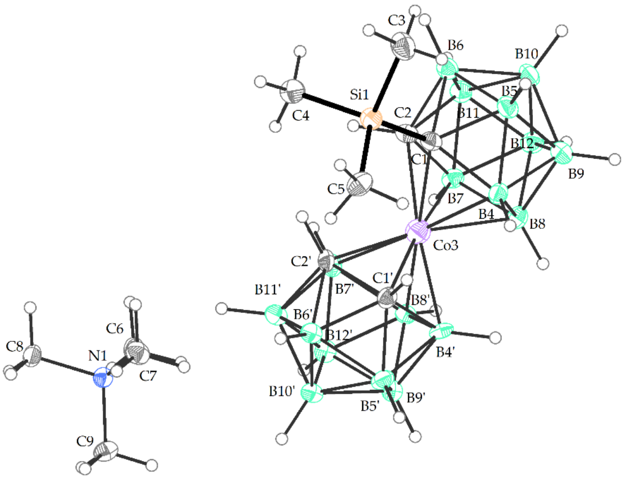

Figure S17. The crystal structure of the $Me_4N[(1\text{-}Me_3Si\text{-}1,2\text{-}C_2B_9H_{10})(1',2'\text{-}C_2B_9H_{11})\text{-}3,3'\text{-}Co(III)]$ (Me_4N**6**) (ORTEP view, 30% probability level).

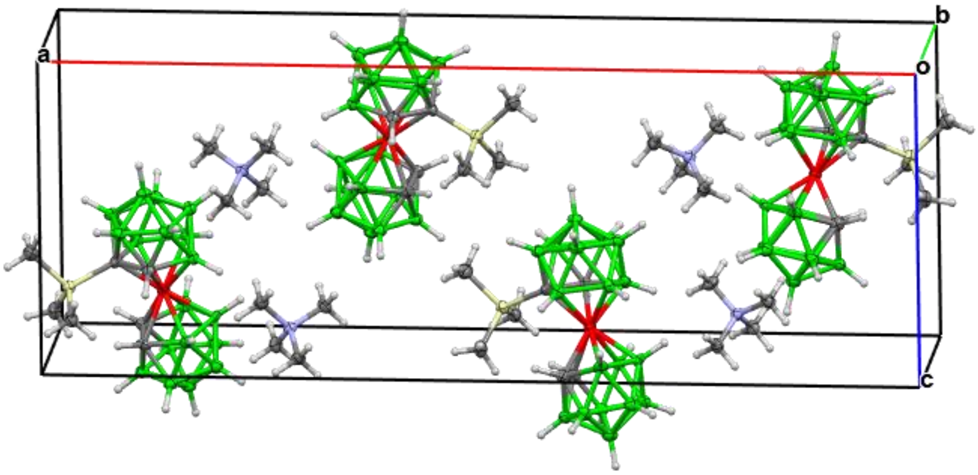

Figure S18. The crystal packing in the structure of $Me_4N[(1\text{-}Me_3Si\text{-}1,2\text{-}C_2B_9H_{10})(1',2'\text{-}C_2B_9H_{11})\text{-}3,3'\text{-}Co(III)]$ (Me_4N**6**). The racemic crystal contains both enantiomers in the unit cell. (ORTEP view, 40% probability level).

Crystal data and structure refinement for $[Me_4N][(1\text{-}H_2N\text{-}CH_2\text{-}1,2\text{-}C_2B_9H_{10})_2\text{-}3,3'\text{-}Co(III)].2HCl$ (VIII)

This unpublished structure is given here for comparison. It demonstrates the uniform type of protonation over the series of C-substituted amino derivatives of the cobalt bis(dicarbollide) ion that proceed via the formation of hydrochlorides; $-NH_2.nHCl$. This is dissimilar to B-substituted compounds containing NH_3^+ groups.[7]

Experimental

The compound was prepared according to a previously published procedure.[8] The structure corresponds to the protonated form isolated from the experiment, when the reaction mixture was acidified with

diluted HCl (3M) and the product was isolated by extraction into Et_2O followed with chromatography. Single crystals corresponding to composition $C_6H_{30}B_{18}CoN_2 \cdot C_4H_{12}N \cdot 2(Cl) \cdot 2(H_2O)$ were grown from CH_2Cl_2 (with addition of MeOH) -Hexane. For details on data collection and refinement see the general Experimental part above.

Table S8. Crystal data and structure refinement for **VIII**

**Crystal data**	
Identification code	CCDC 2374900
Chemical formula	$C_6H_{30}B_{18}CoN_2 \cdot C_4H_{12}N \cdot 2(Cl) \cdot 2(H_2O)$
M_r	564.91
Crystal system, space group	Monoclinic, $P2_1/n$
Temperature (K)	150
a, b, c (Å)	7.4177(2), 14.4885(6), 27.846(1)
β (°)	91.368(1)
V (Å^3)	2991.79(18)
Z	4
Radiation type	Mo$K\alpha$
μ (mm^{-1})	0.77
Crystal size (mm)	0.24 × 0.13 × 0.09
**Data collection**	
Diffractometer	Bruker D8 - Venture
Absorption correction	Multi-scan<br>*SADABS2016*/2 - Bruker AXS area detector scaling and absorption correction Reference: Krause, L., Herbst-Irmer, R., Sheldrick G.M. & Stalke D., J. Appl. Cryst. 48 (2015) 3-10.
T_{min}, T_{max}	0.684, 0.746
No. of measured, independent and observed [$I > 2\sigma(I)$] reflections	69938, 6881, 5401
R_{int}	0.093
$(\sin \theta/\lambda)_{max}$ (Å^{-1})	0.650
**Refinement**	
$R[F^2 > 2\sigma(F^2)]$, $wR(F^2)$, S	0.042, 0.099, 1.04
No. of reflections	6881
No. of parameters	345
No. of restraints	492
H-atom treatment	H atoms treated by a mixture of independent and constrained refinement
$\Delta\rho_{max}$, $\Delta\rho_{min}$ (e Å^{-3})	0.85, −0.62

Computer programs: Bruker Instrument Service vV6.2.3, *APEX3* v2016.5-0 (Bruker AXS), *SAINT* V8.37A (Bruker AXS Inc., 2015), XT, VERSION 2014/5, *SHELXL2019*/1 (Sheldrick, 2019), *PLATON* (Spek, 2009).

Table S9. Hydrogen-bond geometry (Å, º) for **VIII**

D—H···*A*	*D*—H	H···*A*	*D*···*A*	*D*—H···*A*
O2—H2*W*···Cl2	0.99(1)	2.39(1)	3.273(2)	148(1)
O2—H2*V*···Cl1^{i}	0.75(3)	2.52(3)	3.227(2)	158(4)
O1—H3*V*···Cl1	0.82(4)	2.41(4)	3.197(2)	163(4)
O1—H3*W*···Cl2	0.81(4)	2.27(4)	3.070(2)	168(3)
N1—H1*A*···Cl2^{ii}	0.91	2.20	3.1129(19)	178
N1—H1*B*···Cl1^{iii}	0.91	2.36	3.204(2)	154
N1—H1*C*···O1	0.91	1.93	2.732(3)	146
N2—H21···O2	0.91	1.87	2.754(3)	165
N2—H22···Cl2^{iv}	0.91	2.50	3.280(2)	144
N2—H23···Cl1	0.91	2.32	3.190(2)	160

Symmetry codes: (i) *x*+1, *y*, *z*; (ii) *x*−1, *y*, *z*; (iii) −*x*+1/2, *y*+1/2, −*z*+3/2; (iv) −*x*+3/2, *y*−1/2, −*z*+3/2.

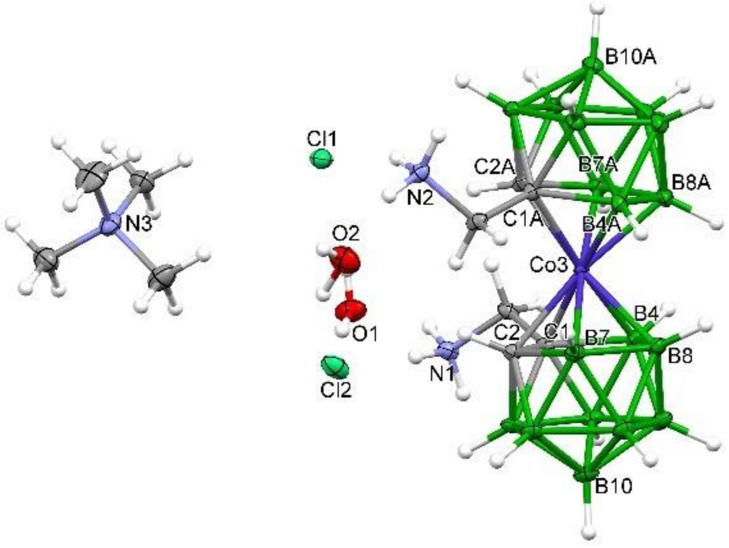

Figure S19. The crystal structure of the protonated form of $[Me_4N][(1,1'\text{-}NH_2\text{-}CH_2\text{-}1,2\text{-}C_2B_9H_{10})_2\text{-}3,3'\text{-}Co(III)].HCl$ (Me_4N**VIII**).HCl (ORTEP view, 40% probability level). The structure contains a hydrochloride moiety and two water molecules.

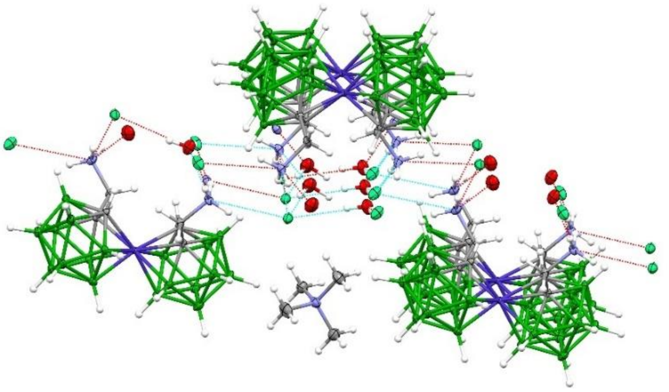

Figure S20. The formation of hydrogen bonds between the amino groups and the hydrochloride moiety in the structure of $Me_4N[(1\text{-}H_2N\text{-}CH_2\text{-}1,2\text{-}C_2B_9H_{10})_2\text{-}3,3'\text{-}Co(III)].2HCl.2H_2O$ (ORTEP view, 50% probability level).

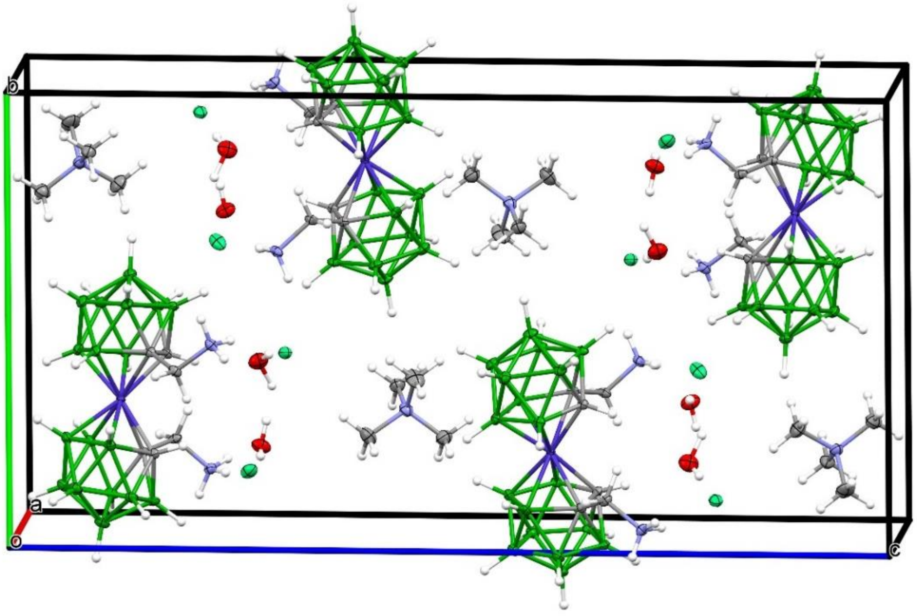

Figure S21. The crystal packing in the structure of $Me_4N[(1\text{-}H_2N\text{-}CH_2\text{-}1,2\text{-}C_2B_9H_{11})_2\text{-}3,3'\text{-}Co(III)].HCl$. (ORTEP view, 40% probability level).

Crystal data and structure refinement for $[Me_4N][(1\text{-}H_2N\text{-}C_2H_4\text{-}1,2\text{-}C_2B_9H_{10})(1',2'\text{-}C_2B_9H_{11})\text{-}3,3'\text{-}Co(III)].HCl$ (Me_4NIII)

This unpublished structure is given here for comparison. It demonstrates the uniform type of protonation over the series of C-substituted amino derivatives of the cobalt bis(dicarbollide) ion that proceed *via* the formation of hydrochlorides $-NH_2.HCl$. This is dissimilar to B-substituted compounds containing NH_3^+ groups.[7]

Experimental

The compound was prepared according to a previously published procedure.[8] The structure corresponds to the protonated form isolated from the experiment, when the reaction mixture was acidified with diluted HCl (3M) and the product was isolated by extraction into Et_2O followed with with chromatography. Single crystals corresponding to composition $C_{16.5}H_{69}B_{36}ClCo_2N_3O_{0.5}$ were grown from CH_2Cl_2 (with the addition of MeOH) -Hexane. A suitable crystal was selected and measured on

a XtaLAB Synergy, Dualflex, HyPix diffractometer, Rigaku. The crystal was kept at 100.00(10) K during data collection. Using Olex2 [1], the structure was solved with the SHELXT [2] structure solution program using Intrinsic Phasing and refined with the SHELXL [3] refinement package using Least Squares minimisation.

Crystal Data for $C_{16.5}H_{69}B_{36}ClCo_2N_3O_{0.5}$ (M =860.21 g/mol): monoclinic, space group C2/c (no. 15), a = 30.1120(3) Å, b = 9.50090(10) Å, c = 34.0551(4) Å, β = 110.8860(10)°, V = 9102.67(18) Å^3, Z = 8, T = 100.00(10) K, μ(Cu Kα) = 6.392 mm^{-1}, $Dcalc$ = 1.255 g/cm^3, 155400 reflections measured (5.556° ≤ 2Θ ≤ 133.184°), 8059 unique (R_{int} = 0.0867, R_{sigma} = 0.0259) which were used in all calculations. The final R_1 was 0.0424 (I > 2σ(I)) and wR_2 was 0.1192 (all data).

Table S10. Crystal data and structure refinement for $Me_4N[(1\text{-}H_2N\text{-}C_2H_4\text{-}1,2\text{-}C_2B_9H_{10})(1',2'\text{-}C_2B_9H_{11})\text{-}3,3'\text{-}Co(III)].HCl$

**Crystal data**	
Identification code	CCDC 2359154
Empirical formula	$C_{16.5}H_{69}B_{36}ClCo_2N_3O_{0.5}$
Formula weight	860.21
Temperature/K	100.00(10)
Crystal system	monoclinic
Space group	C2/c
a/Å	30.1120(3)
b/Å	9.50090(10)
c/Å	34.0551(4)
α/°	90
β/°	110.8860(10)
γ/°	90
Volume/Å^3	9102.67(18)
Z	8
ρ_{calc}g/cm^3	1.255
μ/mm^{-1}	6.392
F(000)	3552.0
Crystal size/mm^3	0.474 × 0.051 × 0.041

**Data collection and refinement**	
Radiation	Cu Kα (λ = 1.54184)
2Θ range for data collection/°	5.556 to 133.184
Index ranges	-35 ≤ h ≤ 35, -11 ≤ k ≤ 10, -40 ≤ l ≤ 40
Reflections collected	155400
Independent reflections	8059 [R_{int} = 0.0867, R_{sigma} = 0.0259]
Data/restraints/parameters	8059/0/601
Goodness-of-fit on F^2	1.038
Final R indexes [I>=2σ (I)]	R_1 = 0.0424, wR_2 = 0.1155
Final R indexes [all data]	R_1 = 0.0475, wR_2 = 0.1192
Largest diff. peak/hole / e Å^{-3}	0.57/-0.50

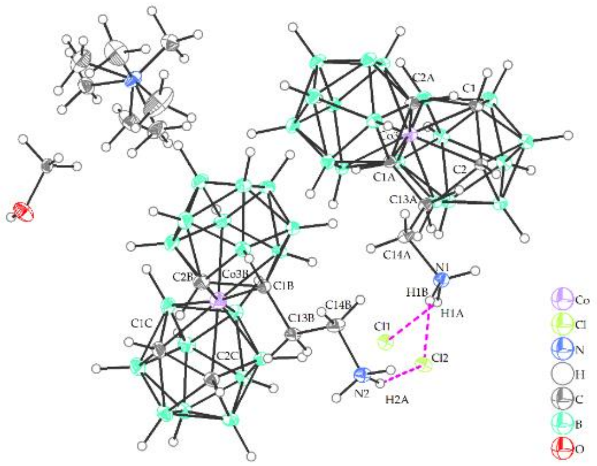

Figure S22. The presence of hydrogen bonds between the amino groups and the chloride anion in the structure of $Me_4N[(1\text{-}H_2N\text{-}C_2H_4\text{-}1,2\text{-}C_2B_9H_{10})(1',2'\text{-}C_2B_9H_{11})\text{-}3,3'\text{-}Co(III)].HCl$ (ORTEP view, 30% probability level). Selected interatomic distances (Å) and angles (°): N1-C14A 1.490 (3), N1-C14B 1.484(3), C13A-C14A 1.522(3), C1-C2 1.608(3), C1A-C2A 1.633(3), C1B C2B 1.647(3), Co(3)-C1 2.042(2), Co(3)-C1A 2.112(2), Co(3)-C2A 2.073(2), C1-B4 1.725(3), C1-B5 1.685(3), C1-B6 1.733(3), C1A-B5A 1.712(3), C1A-B6A 1.734(3), C2-B7 1.699(3), C2-B11 1.705(3), C2-B6 1.733(3), N1 C13A C14A 108.48(19), N1 C14A C13A 108.48(19), N2 C13B C14B 108.79(19), C1A Co3 B8 135.02(9), C13A C1A C2A 118.54(18), C13A C1A B4A 125.14(18), C13A C1A B6A 111.40(12), C14A C1A C1A 115.11(19), C1A B4A B8A 106.74(18).

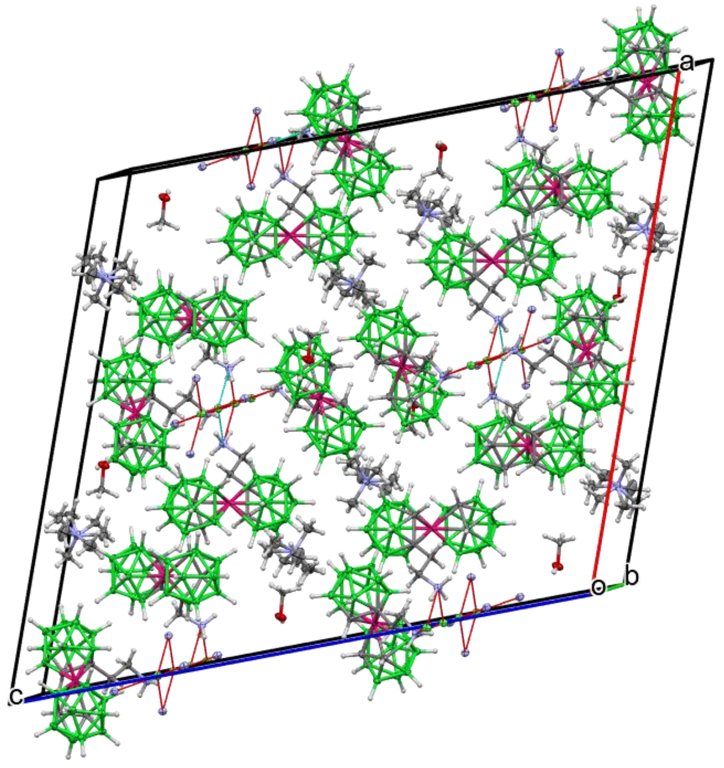

Figure S23. The crystal packing in the structure of $[Me_4N][(1\text{-}H_2N\text{-}C_2H_4\text{-}1,2\text{-}C_2B_9H_{11})(1',2'\text{-}C_2B_9H_{12})\text{-}3,3'\text{-}Co]\cdot HCl$, each Cl^- participates on hydrogen bonding with $C_{cage}\text{-}CH_2\text{-}CH_2\text{-}NH_2$ groups from four neighboring boron clusters. The racemic crystal contains both enantiomers in the unit cell. (ORTEP view, 40% probability level).

NMR Spectra

NMR Spectra of $[Me_4N][(1\text{-}N_3\text{-}1,2\text{-}C_2B_9H_{10})(1',2'\text{-}C_2B_9H_{11})\text{-}3,3'\text{-}Co(III)]$ (Me_4N**2**)

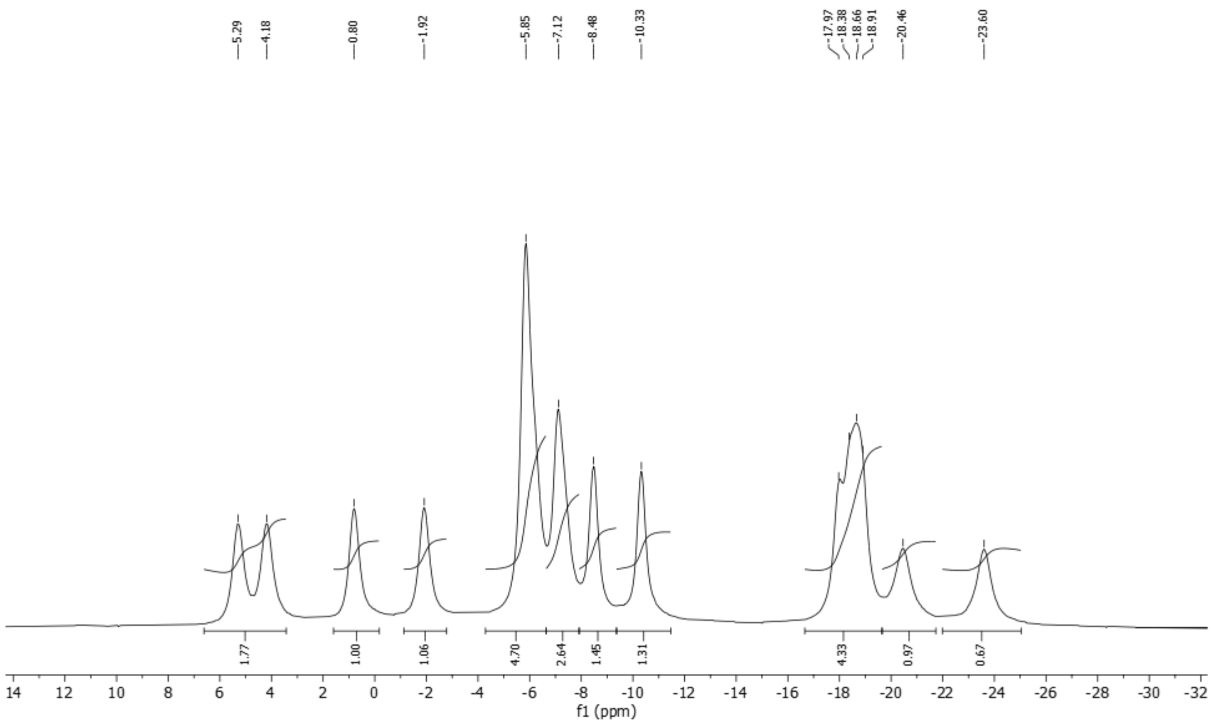

Figure S24. $^{11}B\{^1H\}$ NMR Spectrum of Me_4N**2** in CD_3OD

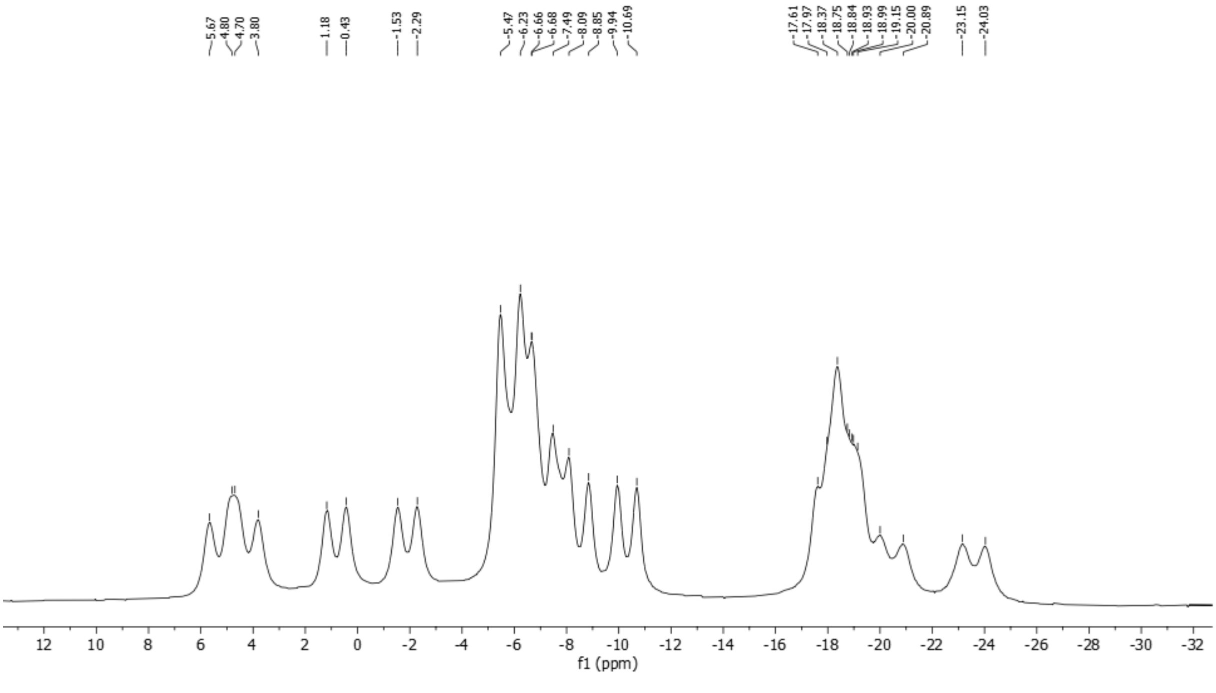

Figure S25. ^{11}B NMR Spectrum of Me_4N**2** in CD_3OD

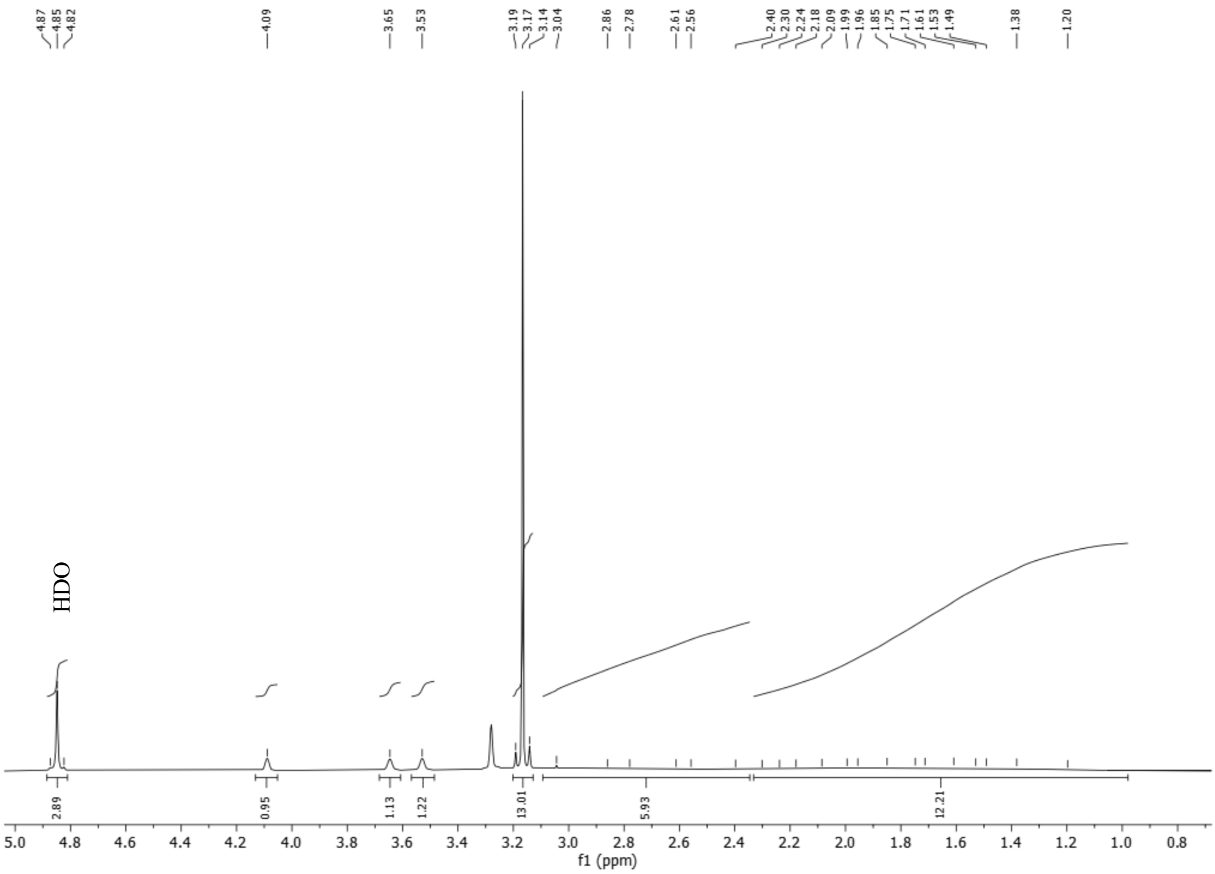

Figure S26. ^{1}H NMR Spectrum of Me_4N**2** in CD_3OD

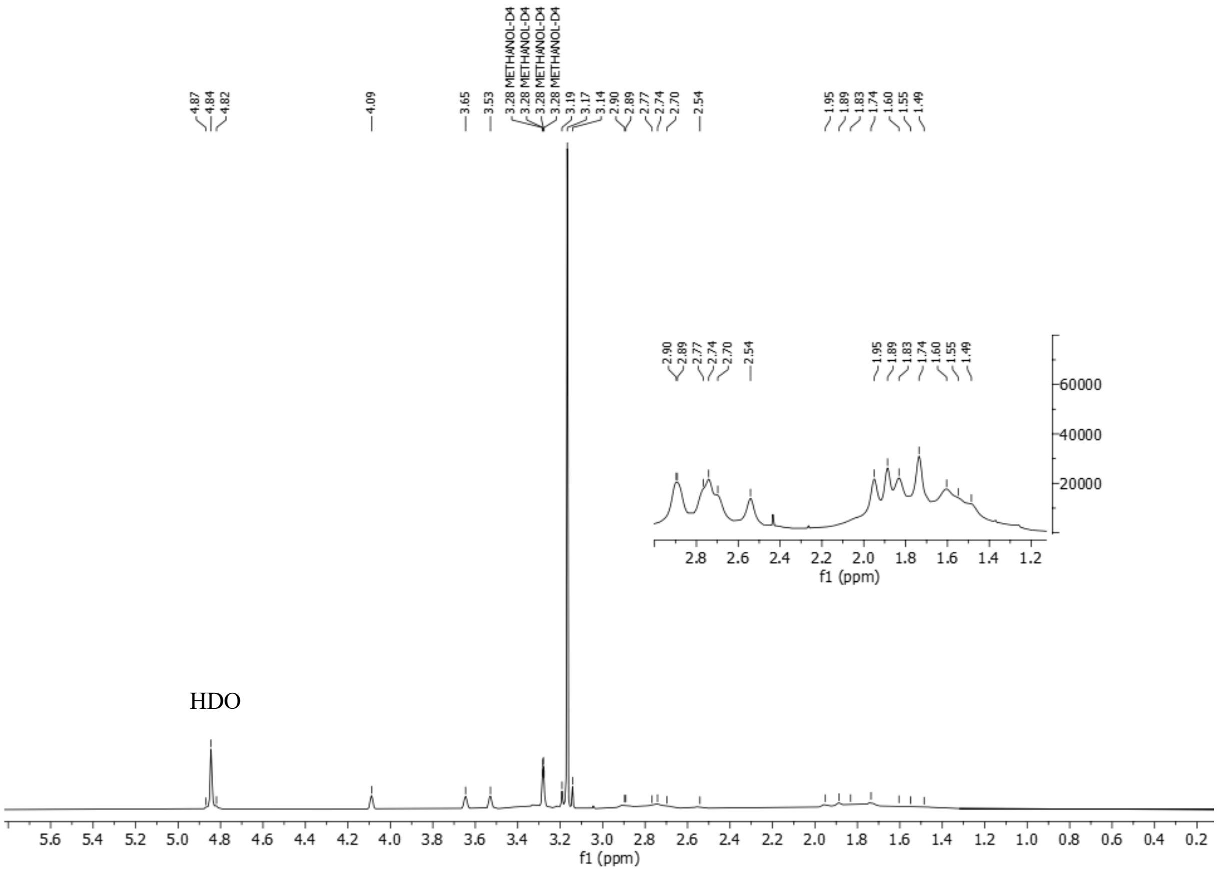

Figure S27. $^1H\{^{11}B\}$ NMR Spectrum of Me_4N**2** in CD_3OD

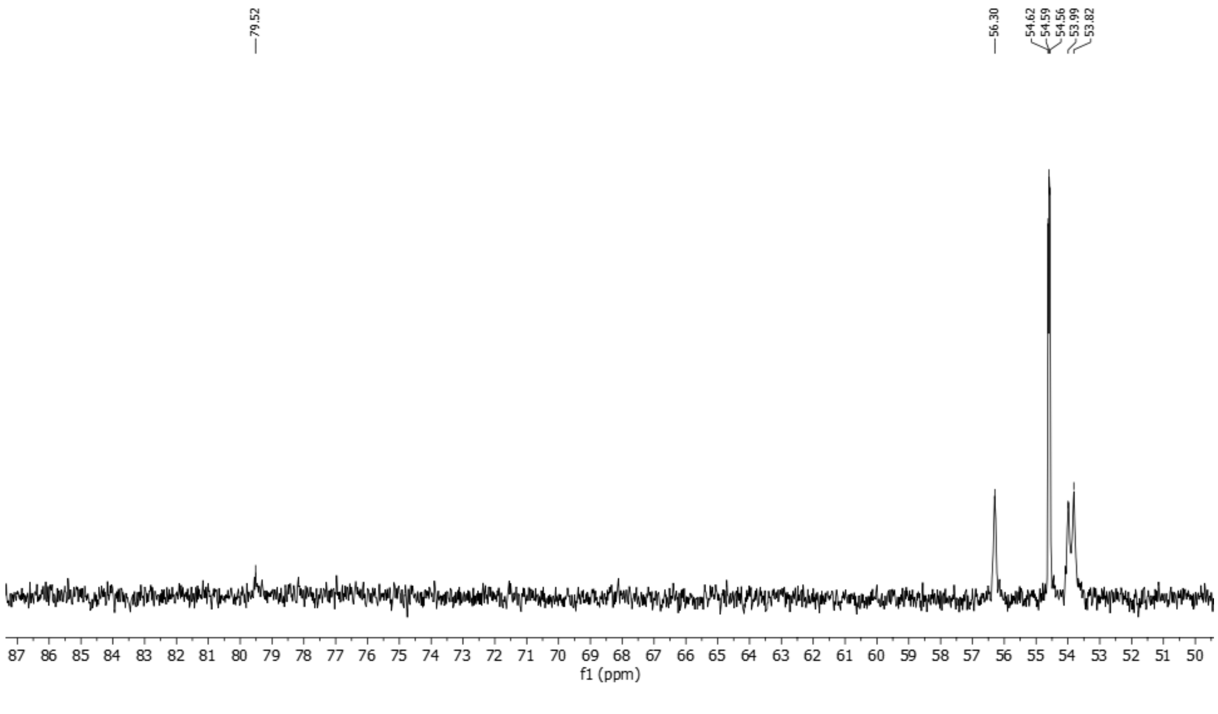

Figure S28. $^{13}C\{^1H\}$ NMR Spectrum of Me_4N**2** in CD_3OD

NMR Spectra of [Me_4N][(1,1'-N_3-1,2-$C_2B_9H_{10}$)$_2$-3,3'-Co(III)] (Me_4N**3)**

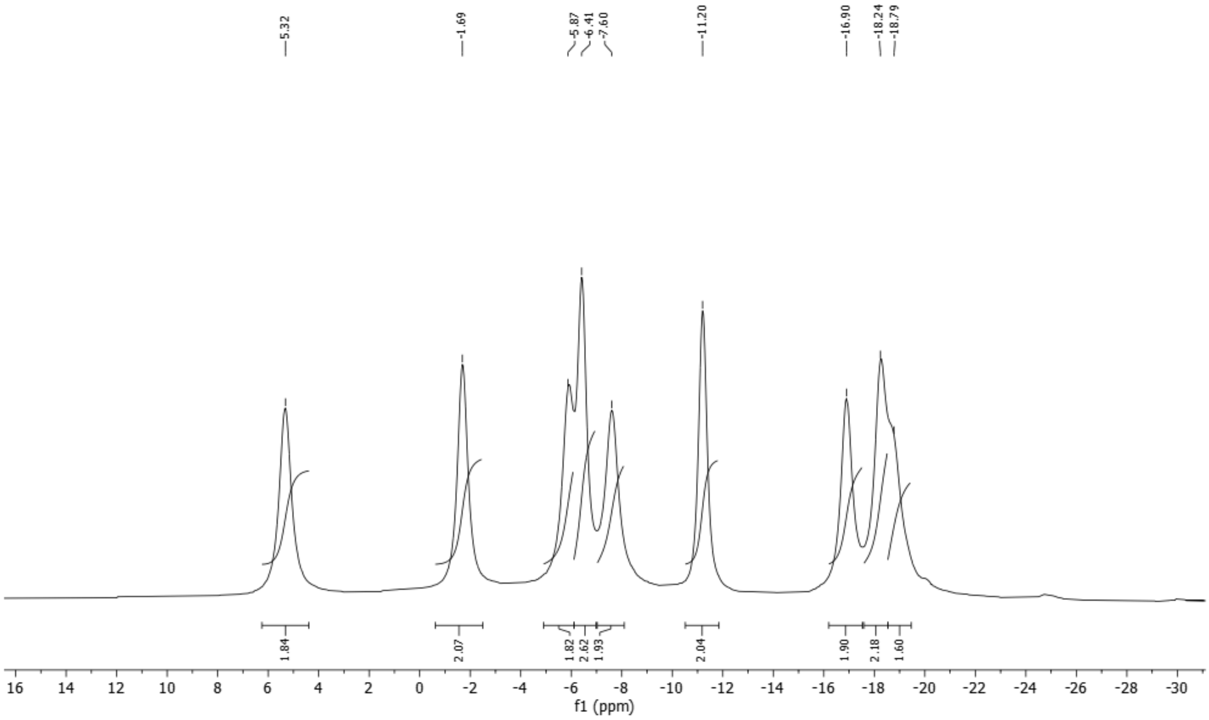

Figure S29. $^{11}B\{^{1}H\}$ NMR Spectrum of Me_4N**3** in CD_3OD

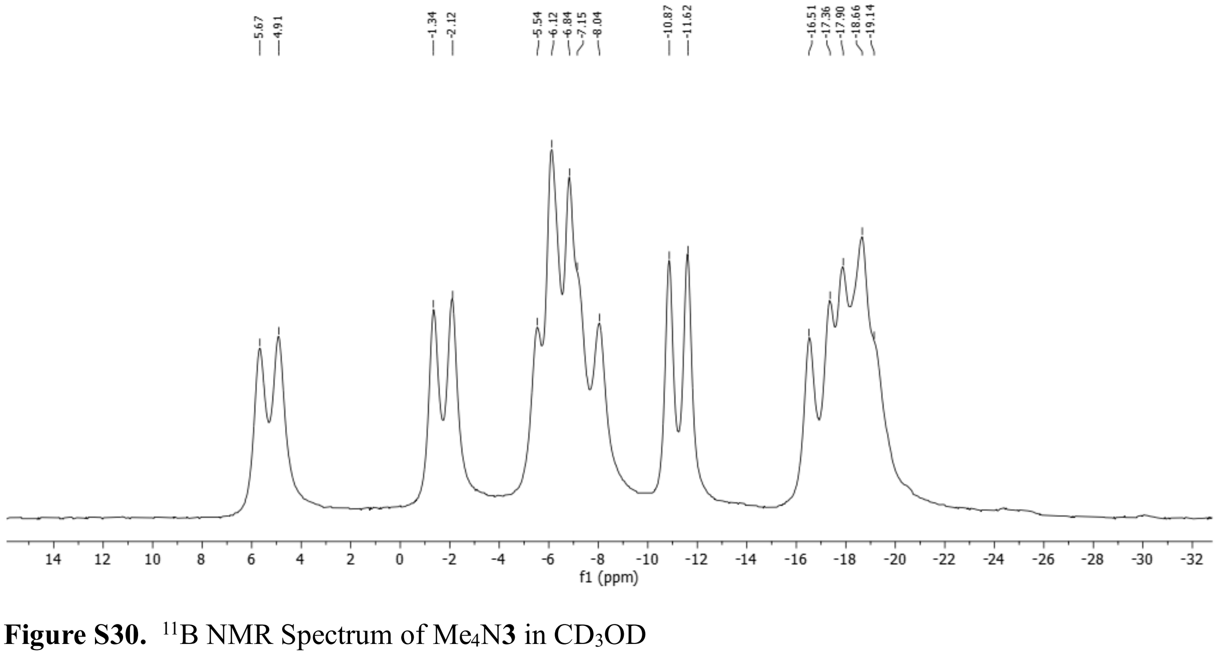

Figure S30. ^{11}B NMR Spectrum of Me_4N**3** in CD_3OD

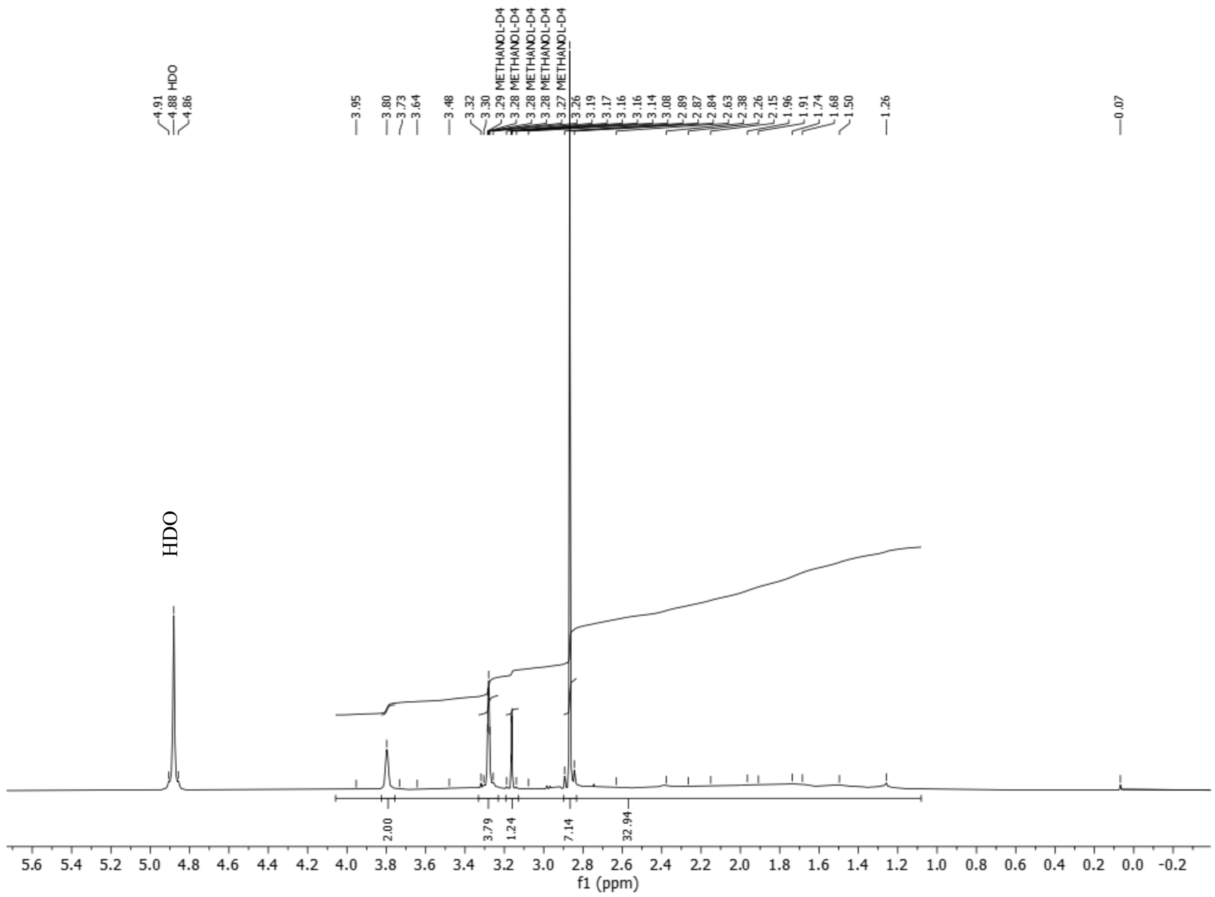

Figure S31. ^{1}H NMR Spectrum of Me_4N**3** in CD_3OD

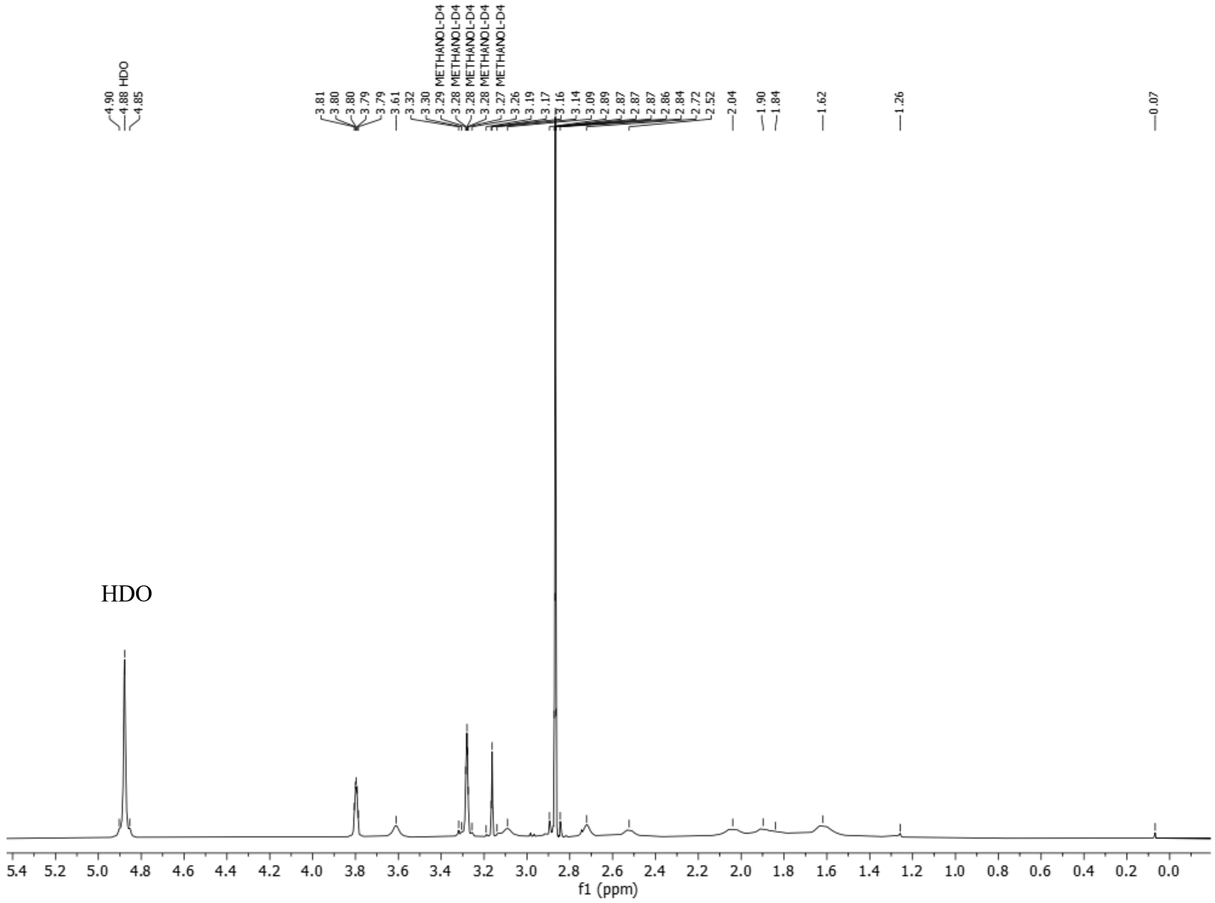

Figure S32. $^{1}H\{^{11}B\}$ NMR Spectrum of Me_4N**3** in CD_3OD

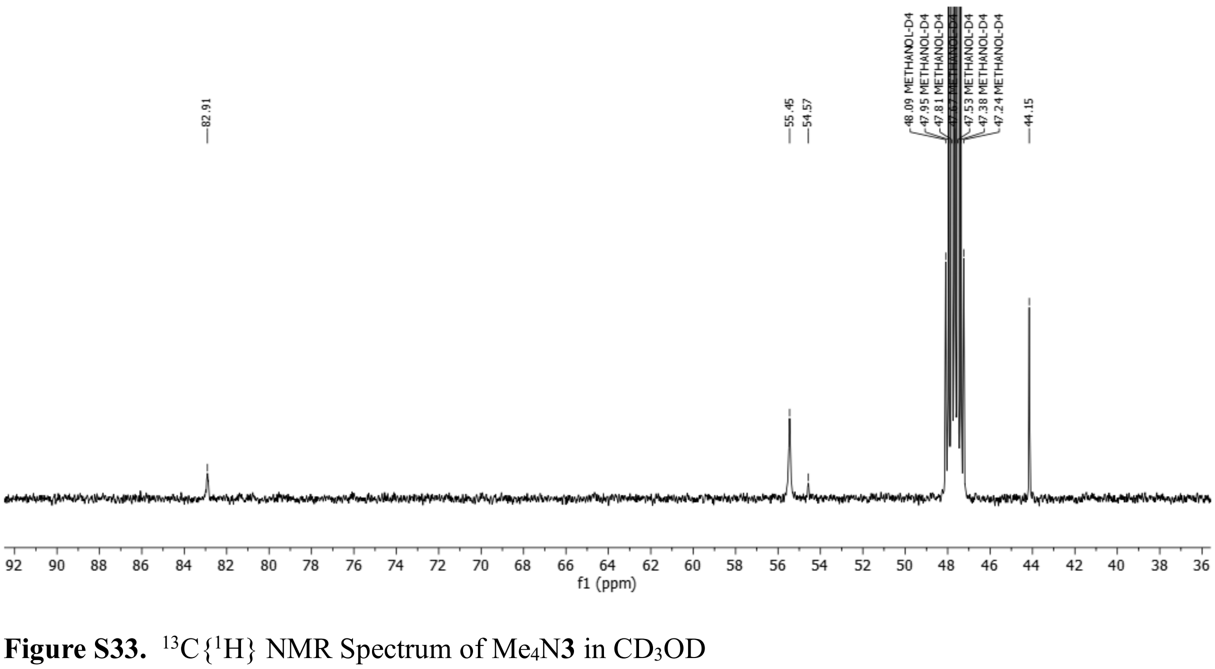

Figure S33. $^{13}C\{^{1}H\}$ NMR Spectrum of Me_4N**3** in CD_3OD

NMR Spectra of [Me_4N][(1-H_2N-1,2-$C_2B_9H_{10}$)(1',2'-$C_2B_9H_{11}$)-3,3'-Co(III)] (**Me_4N4**)

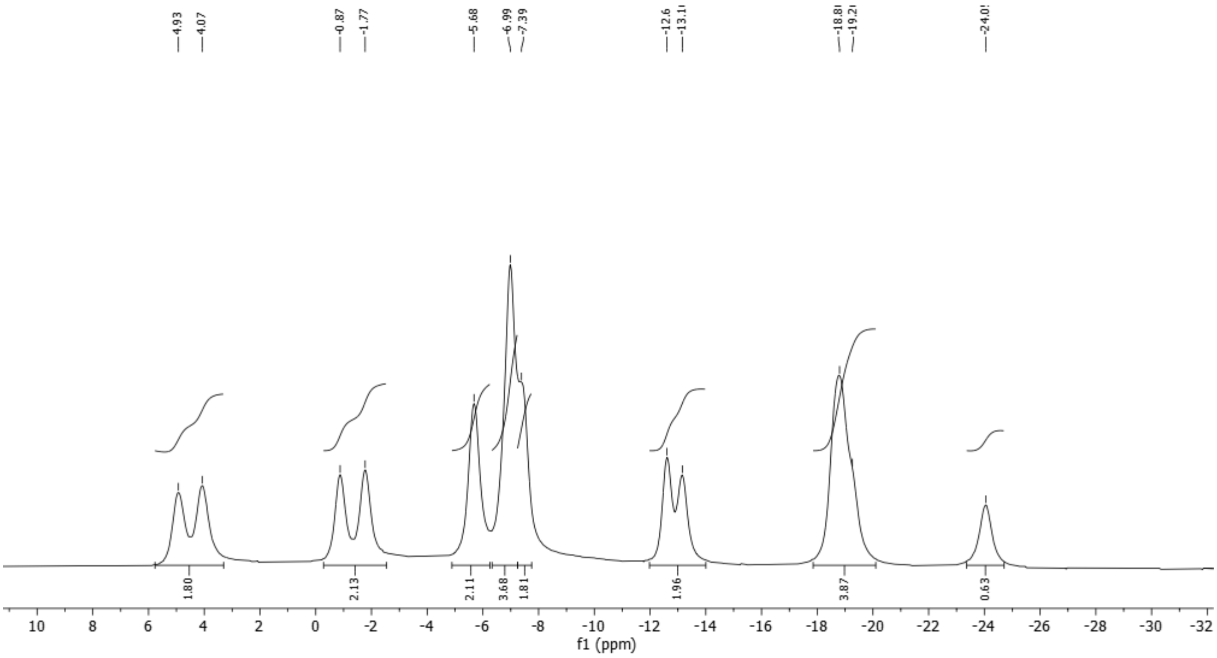

Figure S34. $^{11}B\{^{1}H\}$ NMR Spectrum of Me_4N**4** in $(CD_3)_2CO$

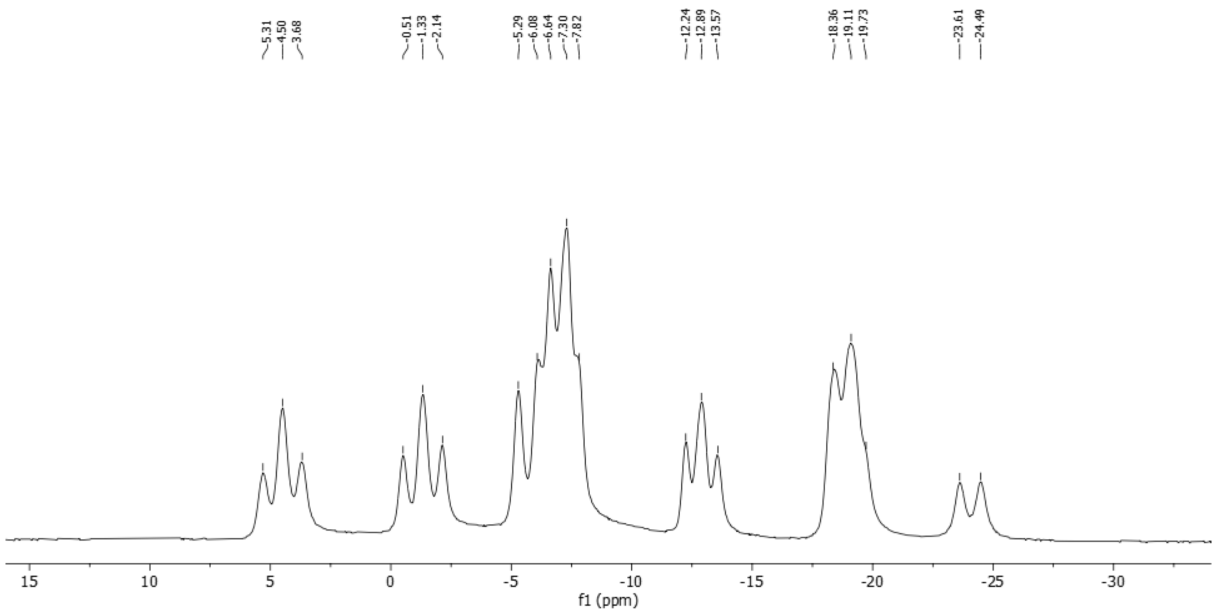

Figure S35. ^{11}B NMR Spectrum of Me_4N**4** in $(CD_3)_2CO$

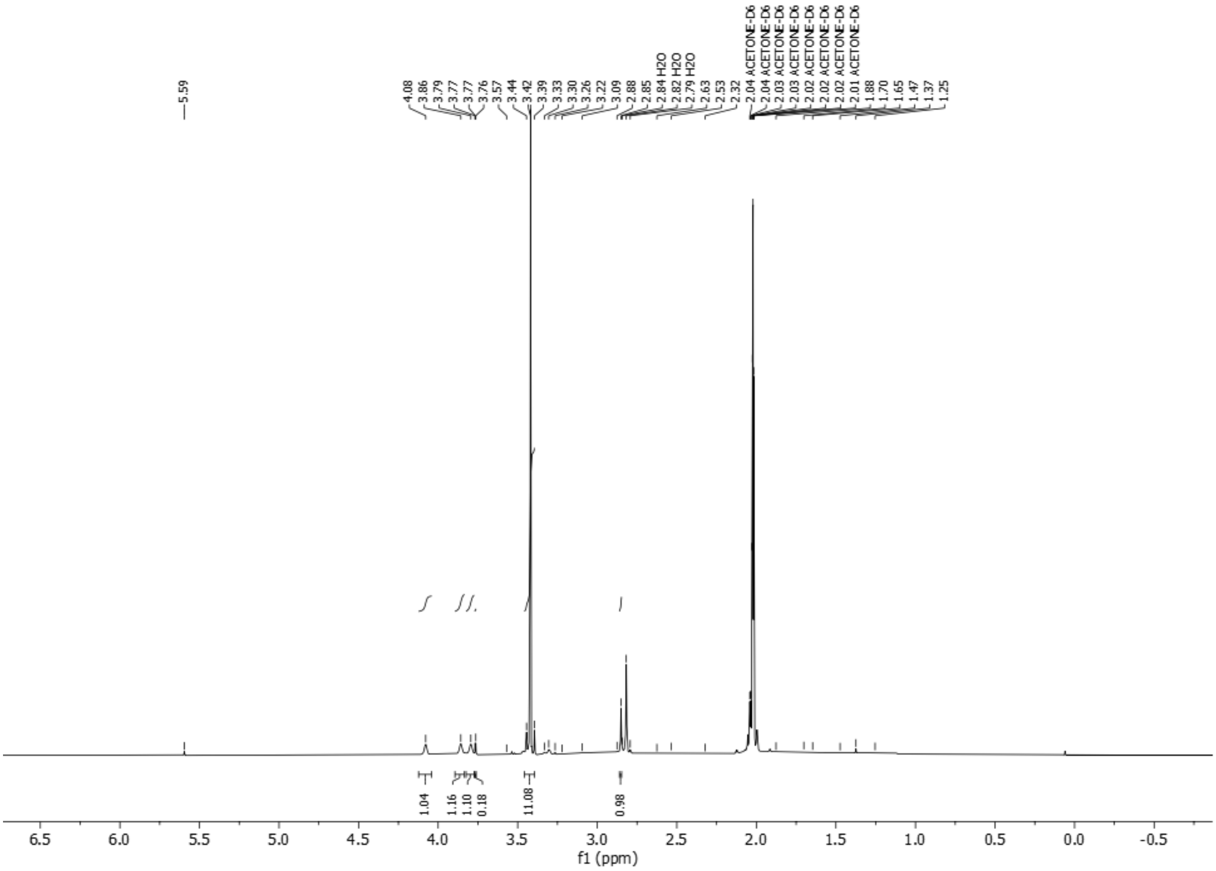

Figure S36. ^{1}H NMR Spectrum of Me_4N**4** in $(CD_3)_2CO$

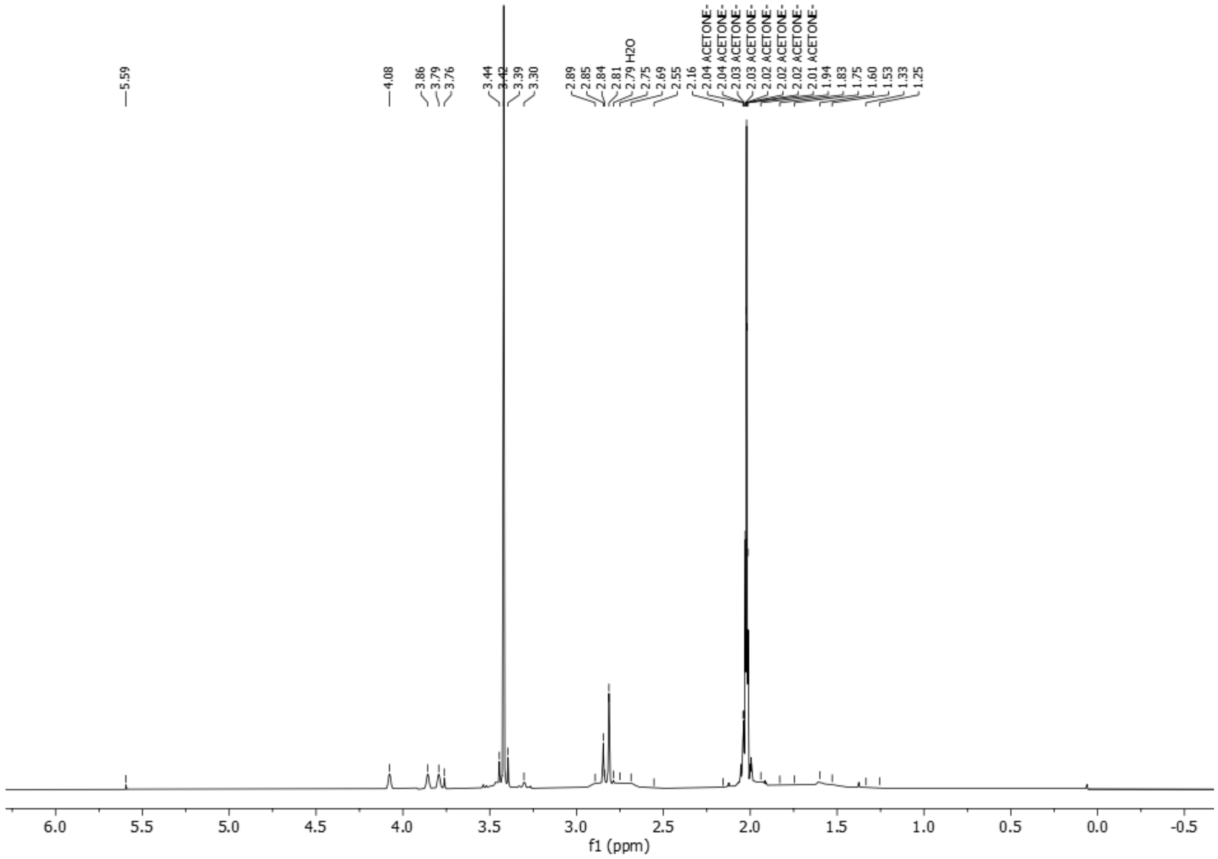

Figure S37. $^{1}H\{^{11}B\}$ NMR Spectrum of Me_4N**4** in $(CD_3)_2CO$

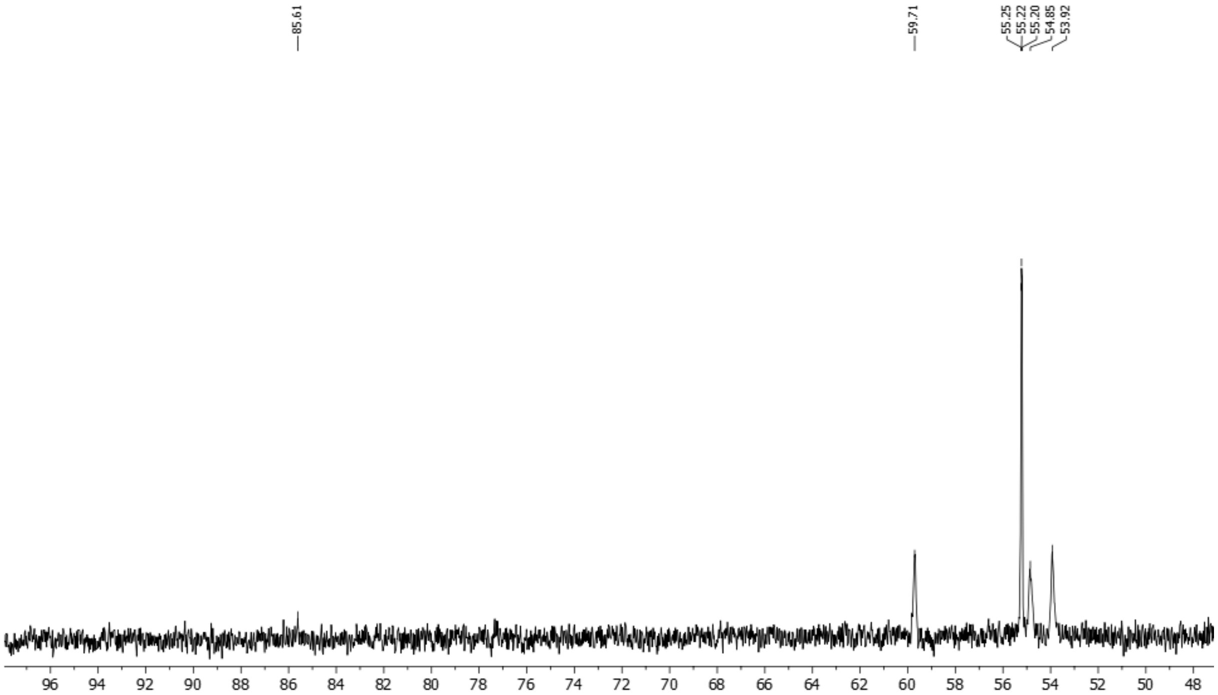

Figure S38. $^{13}C\{^{1}H\}$ NMR Spectrum of Me_4N**4** in $(CD_3)_2CO$

NMR Spectra of [Me4N][(1,1'-NH2-1,2-C2B9H10)2-3,3'-Co(III)] (Me4N**5**)

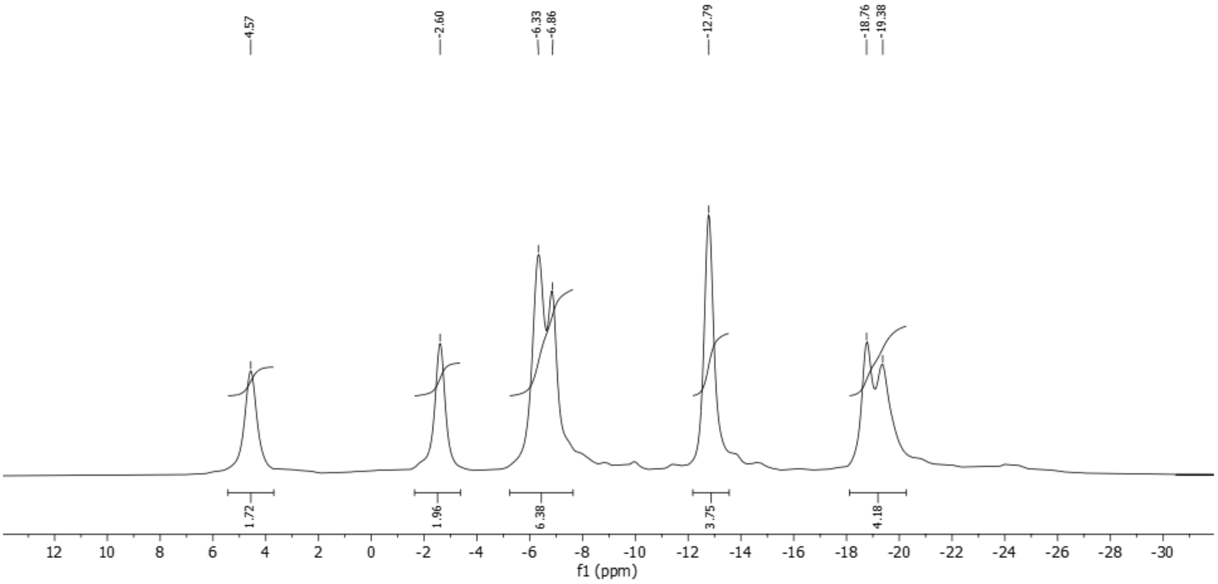

Figure S39. $^{11}B\{^{1}H\}$ NMR Spectrum of Me4N**5** in $(CD_3)_2CO$

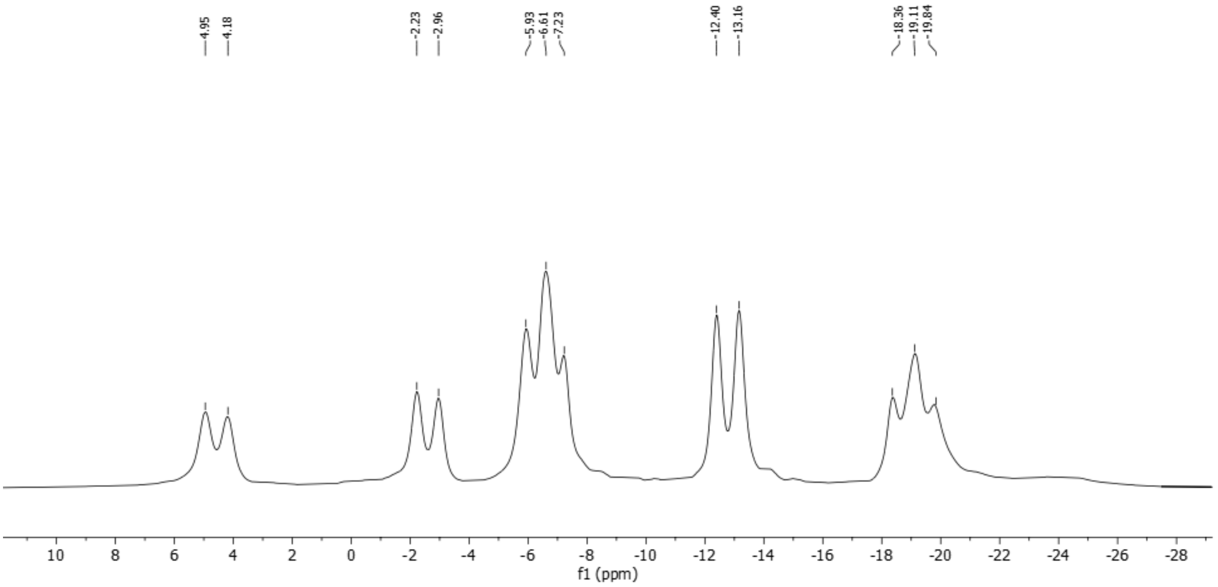

Figure S40. ^{11}B NMR Spectrum of Me4N**5** in $(CD_3)_2CO$

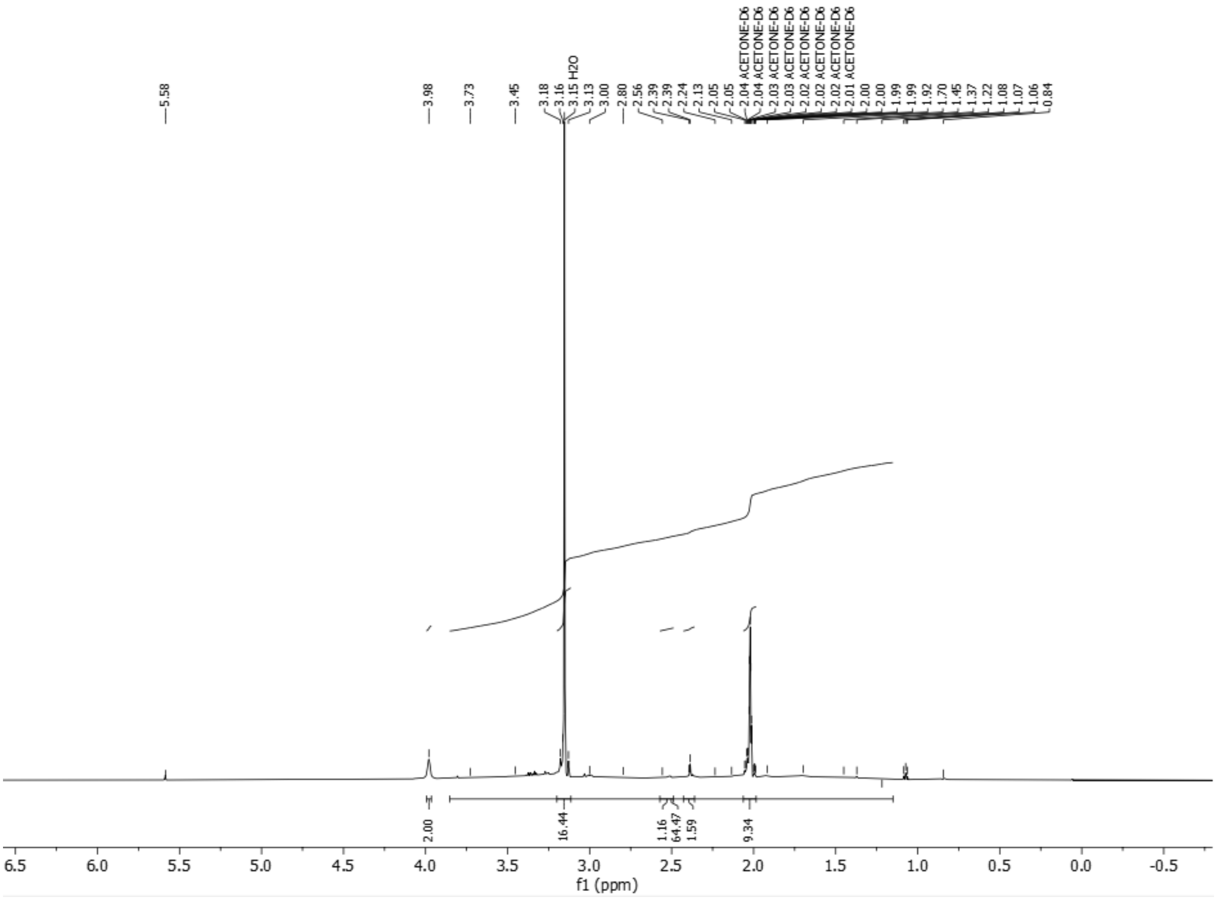

Figure S41. ^{1}H NMR Spectrum of Me_4N**5** in $(CD_3)_2CO$

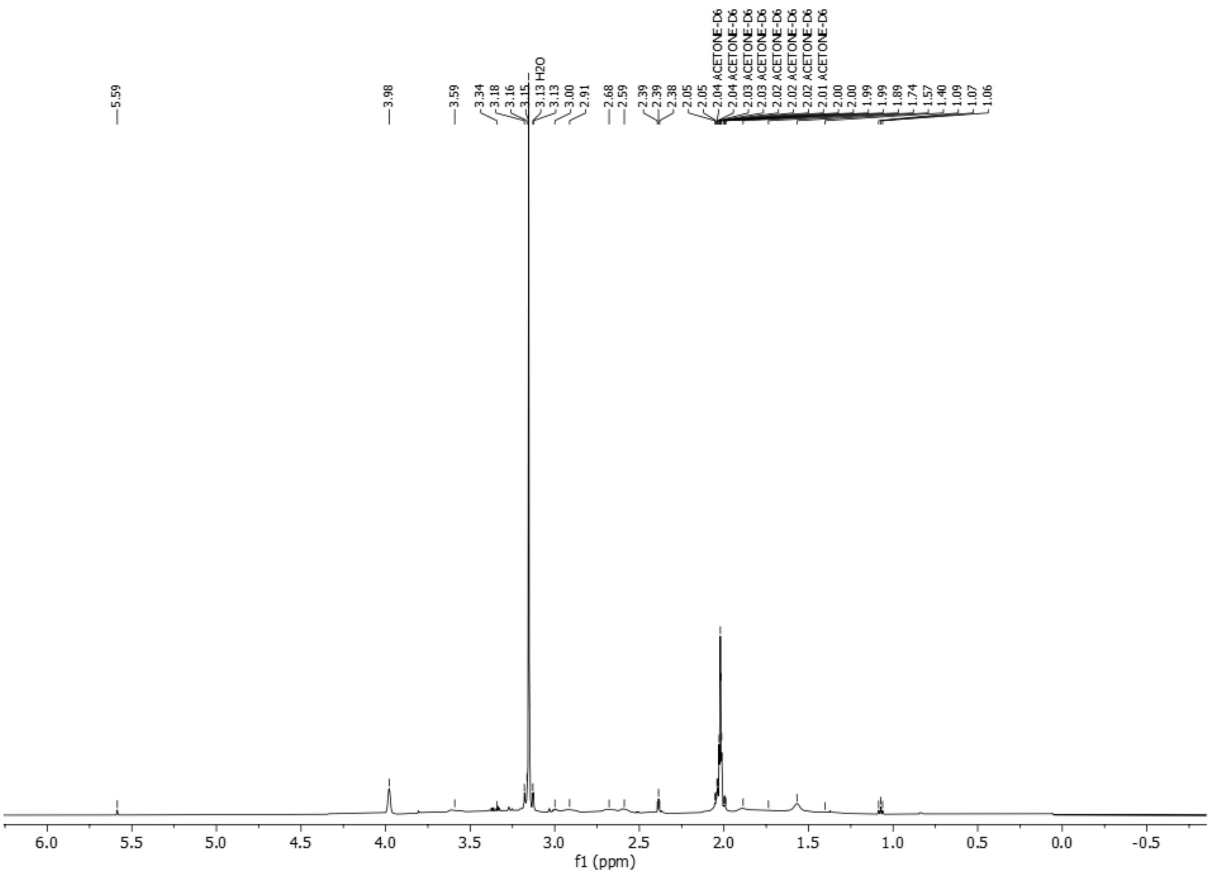

Figure S42. $^{1}H\{^{11}B\}$ NMR Spectrum of Me_4N**5** in $(CD_3)_2CO$

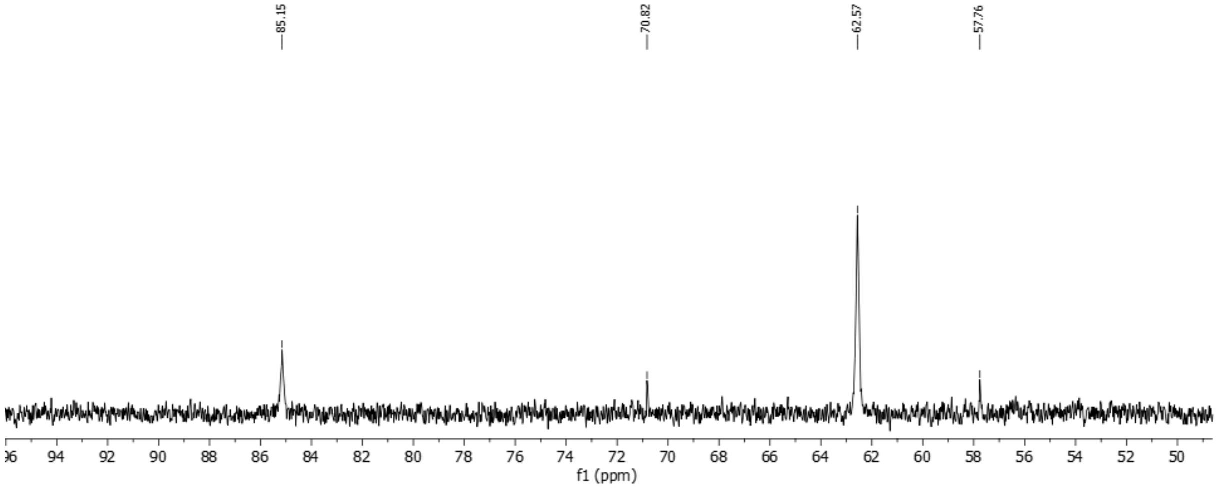

Figure S43. $^{13}C\{^{1}H\}$ NMR Spectrum of Me_4N**5** in $(CD_3)_2CO$

NMR Spectra of [Me_4N][(1-Me_3Si-1,2-$C_2B_9H_{10}$)(1',2'-$C_2B_9H_{11}$)-3,3'-Co(III)] (Me_4N6)

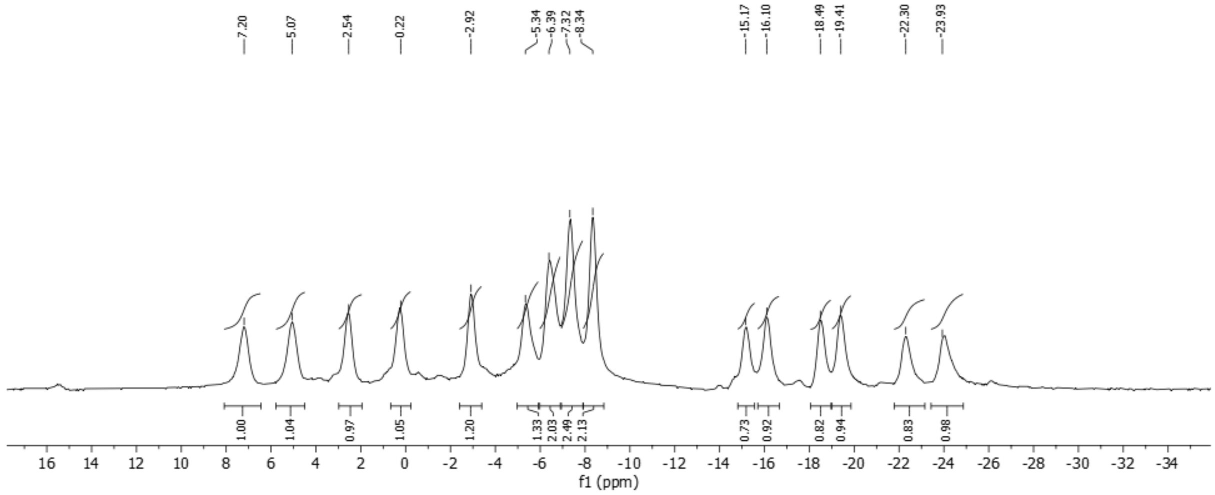

Figure S44. $^{11}B\{^1H\}$ NMR Spectrum of Me_4N**6** in $(CD_3)_2CO$

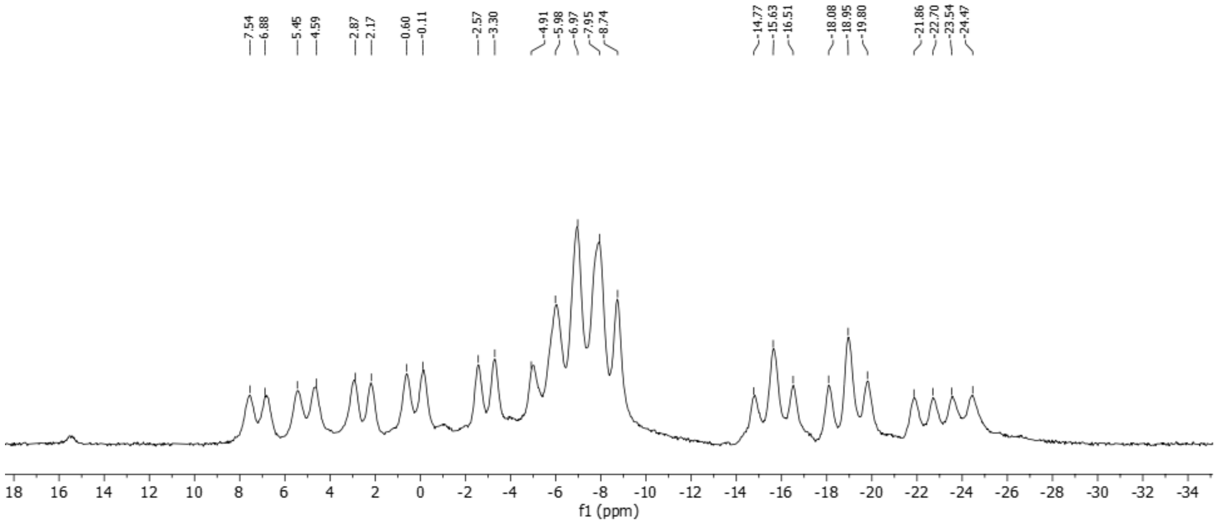

Figure S45. ^{11}B NMR Spectrum of Me_4N**6** in $(CD_3)_2CO$

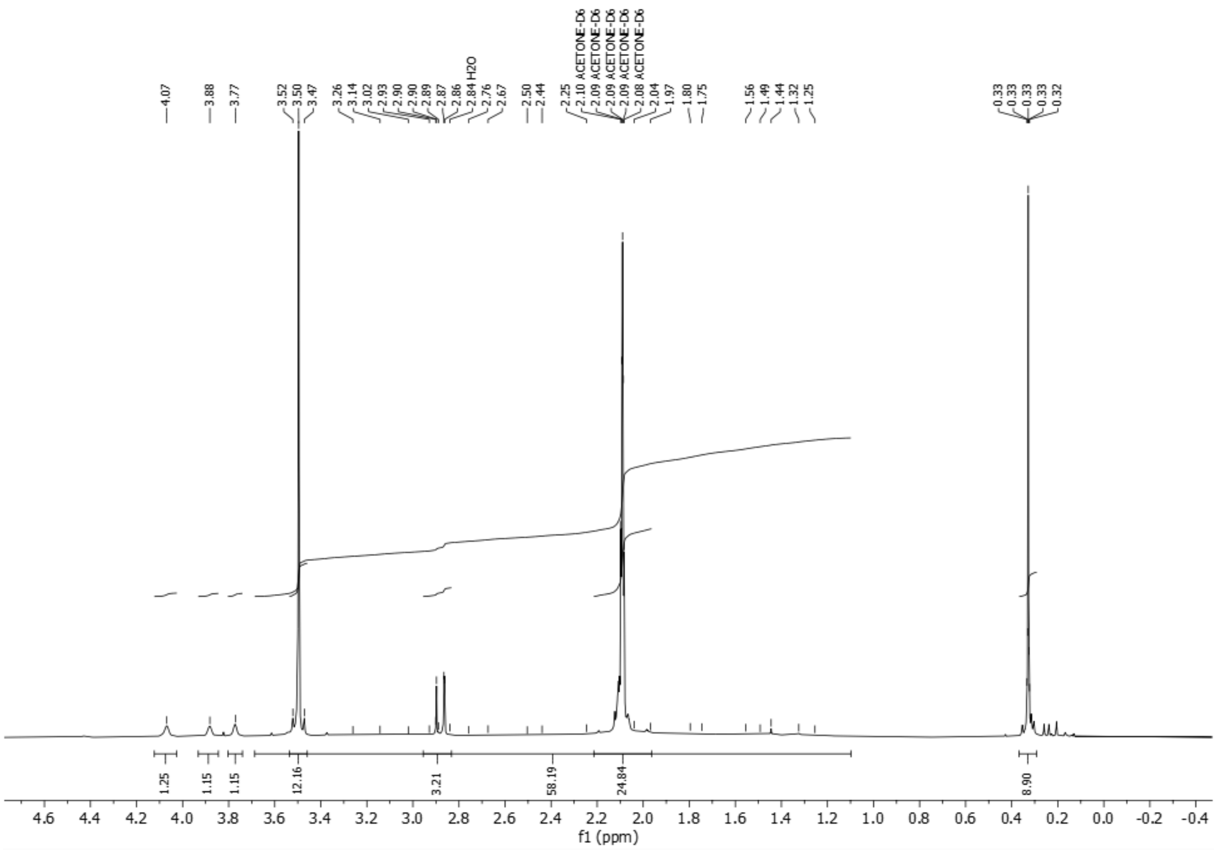

Figure S46. ^{1}H NMR Spectrum of Me_4N**6** in $(CD_3)_2CO$

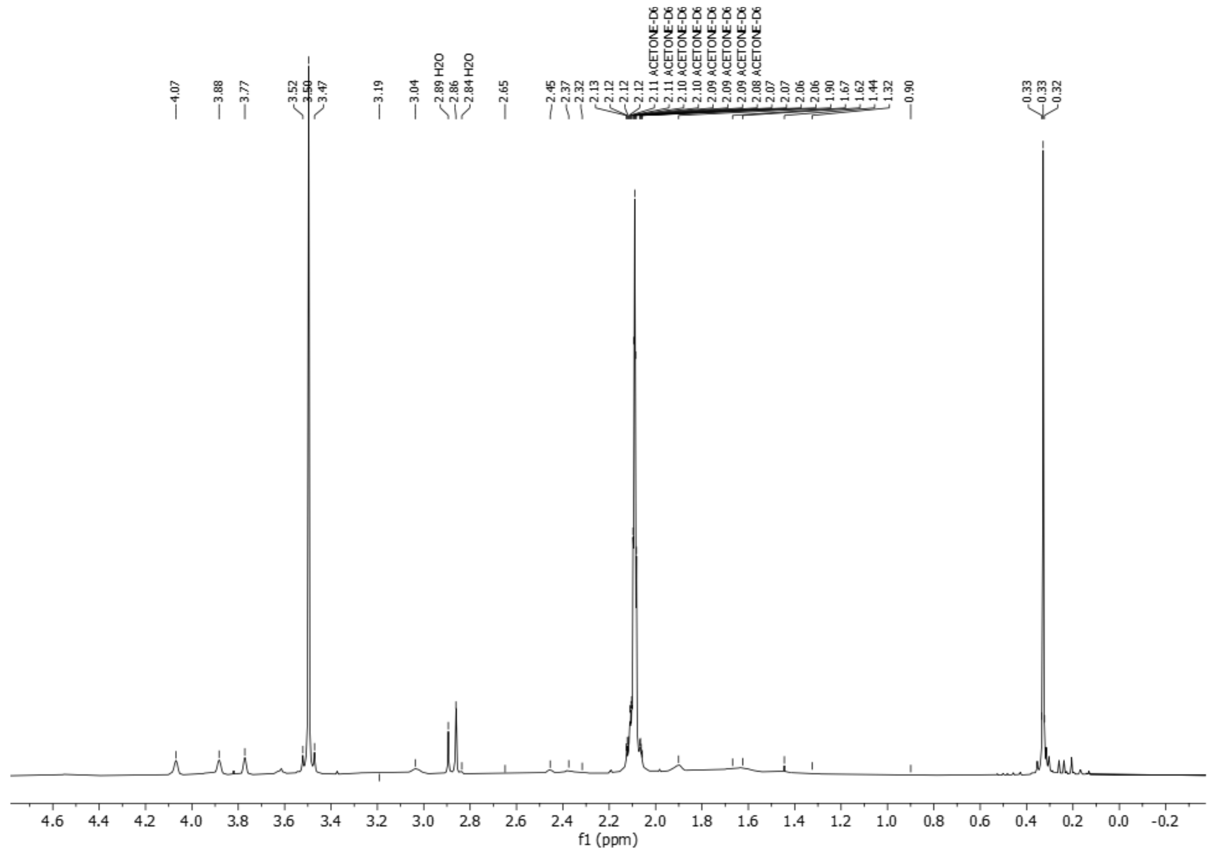

Figure S47. $^{1}H\{^{11}B\}$ NMR Spectrum of Me_4N**6** in $(CD_3)_2CO$

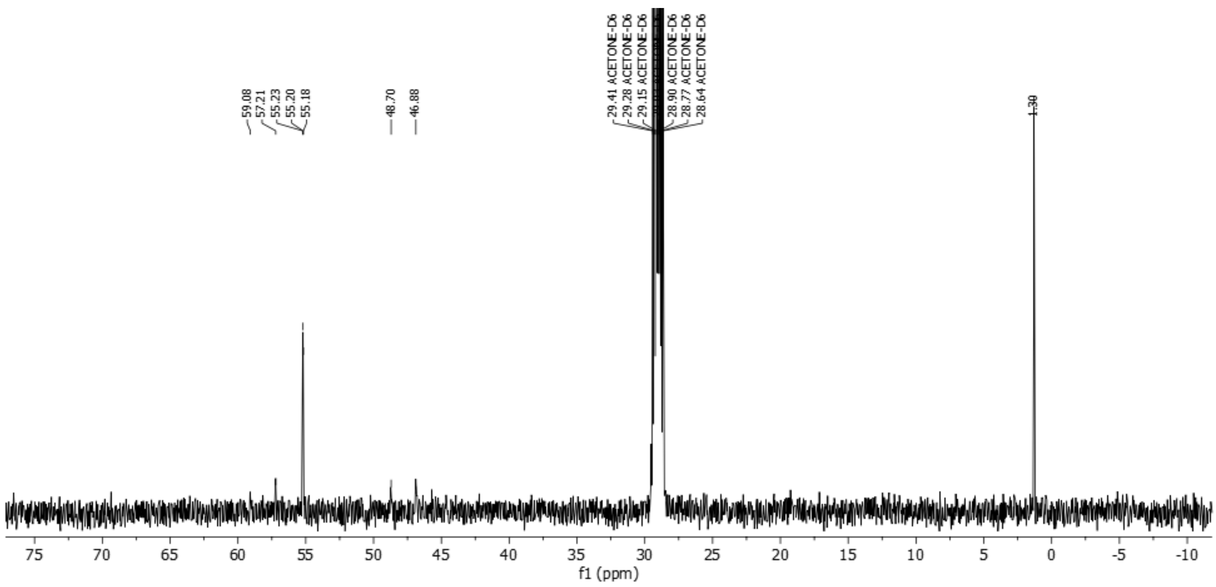

Figure S48. $^{13}C\{^1H\}$ NMR Spectrum of Me_4N**6** in $(CD_3)_2CO$

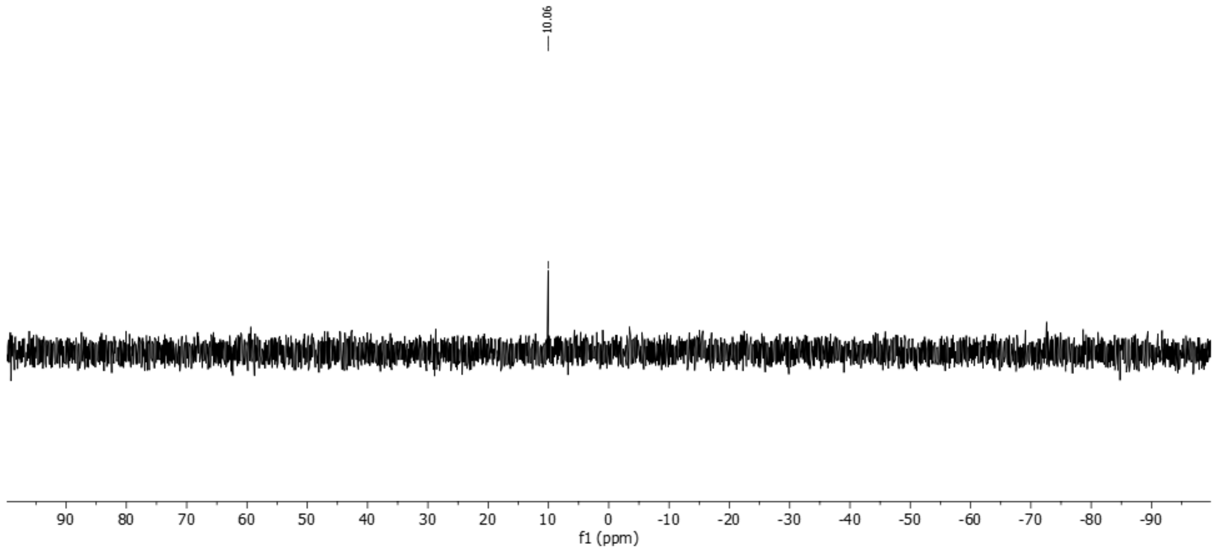

Figure S49. $^{29}Si\{^1H\}$ NMR Spectrum of Me_4N**6** in $(CD_3)_2CO$

NMR Spectra of $[Me_4N][(1,1'-Me_3Si-1,2-C_2B_9H_{10})_2-3,3'-Co(III)]$ (Me_4N7)

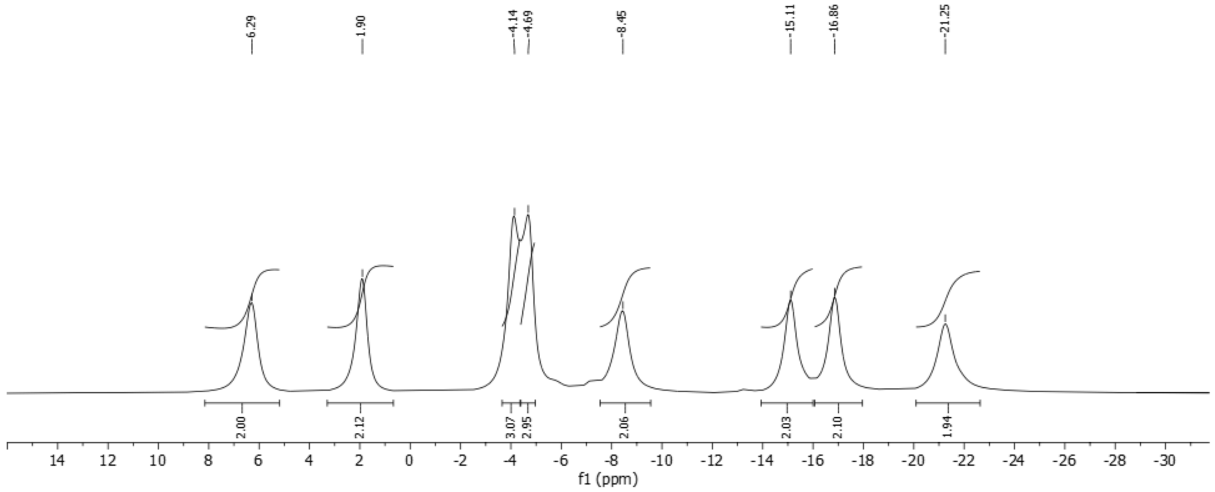

Figure S50. $^{11}B\{^1H\}$ NMR Spectrum of Me_4N**7** in $(CD_3)_2CO$

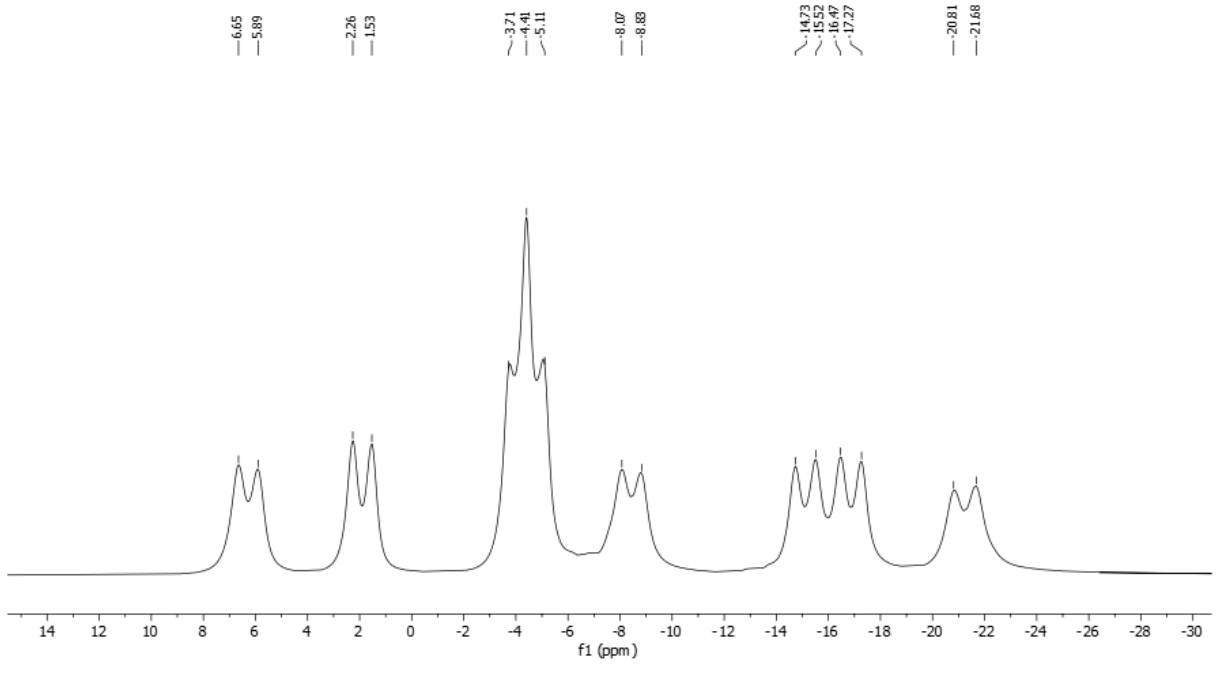

Figure S51. ^{11}B NMR Spectrum of Me_4N**7** in $(CD_3)_2CO$

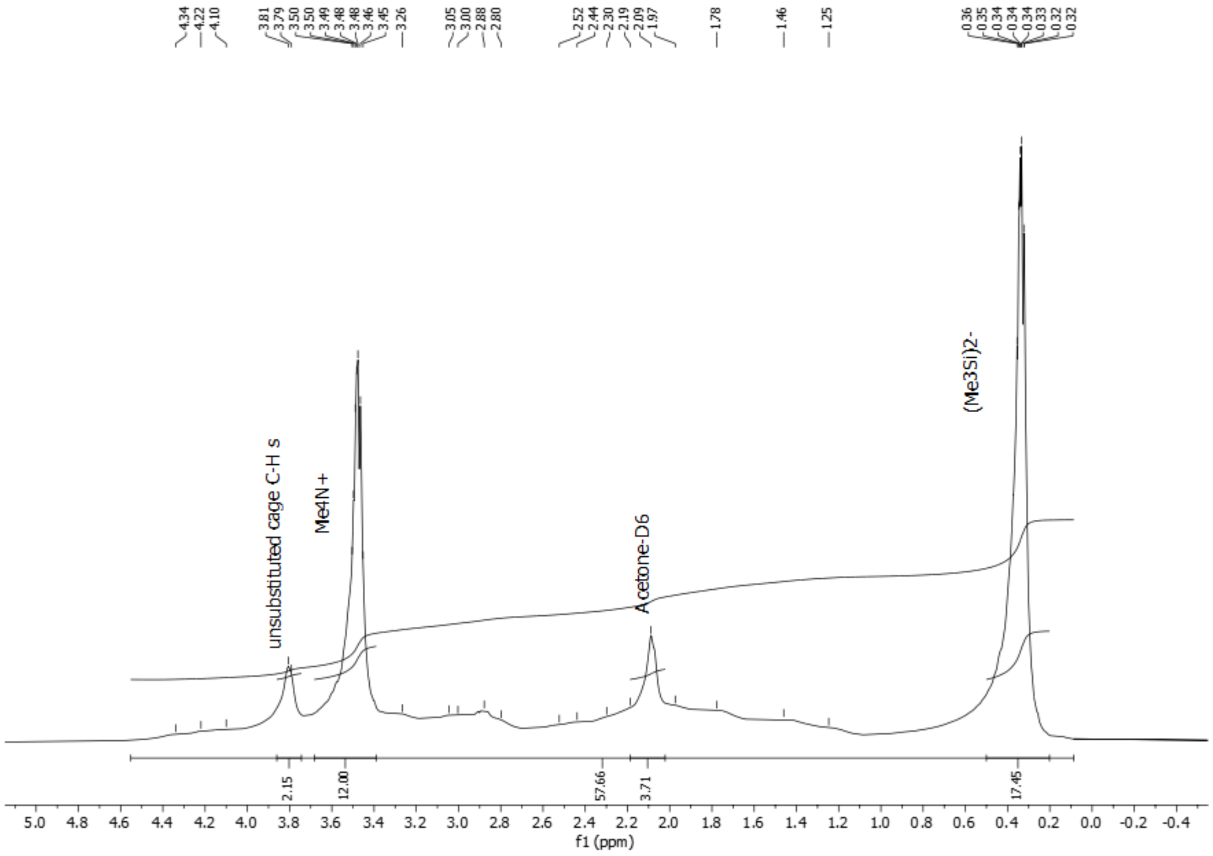

Figure S52. ^{1}H NMR Spectrum of Me_4N**7** in $(CD_3)_2CO$

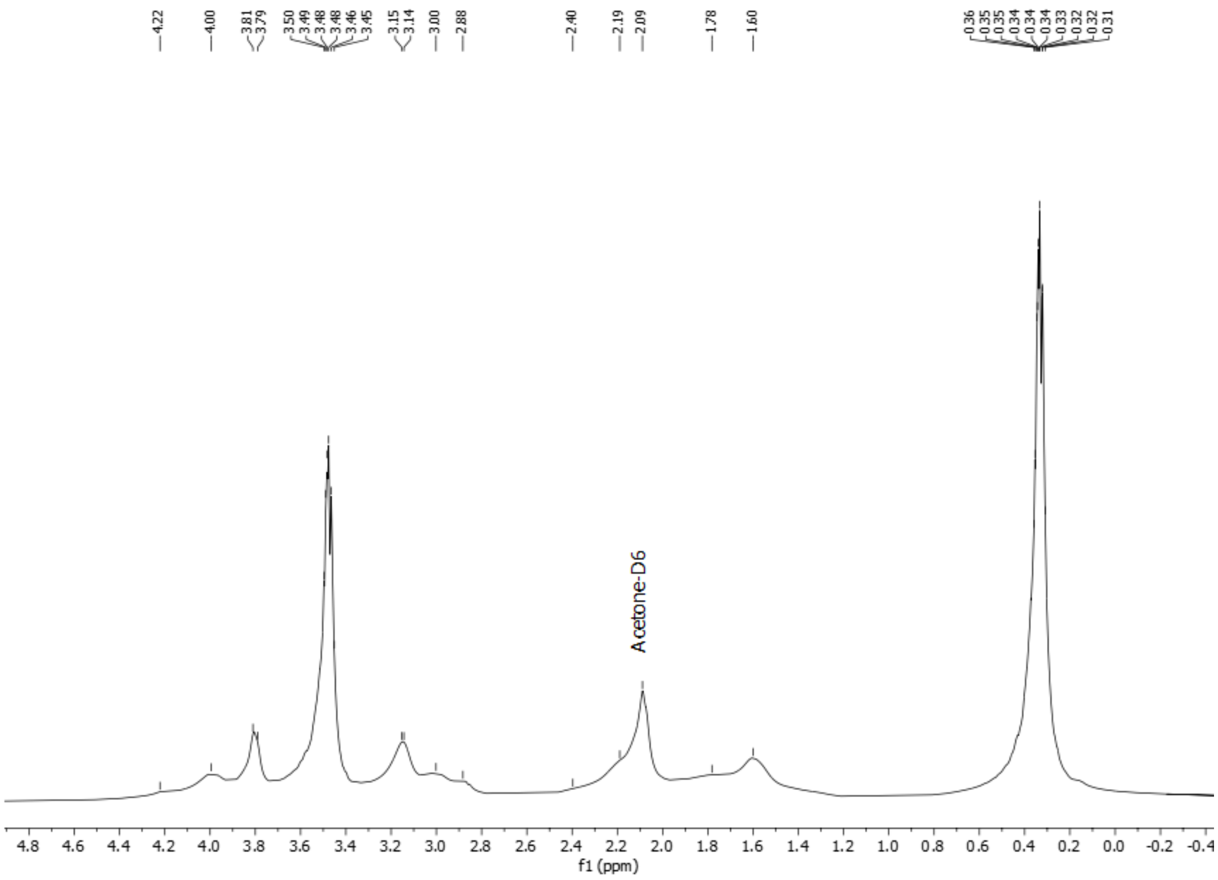

Figure S53. $^{1}H\{^{11}B\}$ NMR Spectrum of Me_4N**7** in $(CD_3)_2CO$

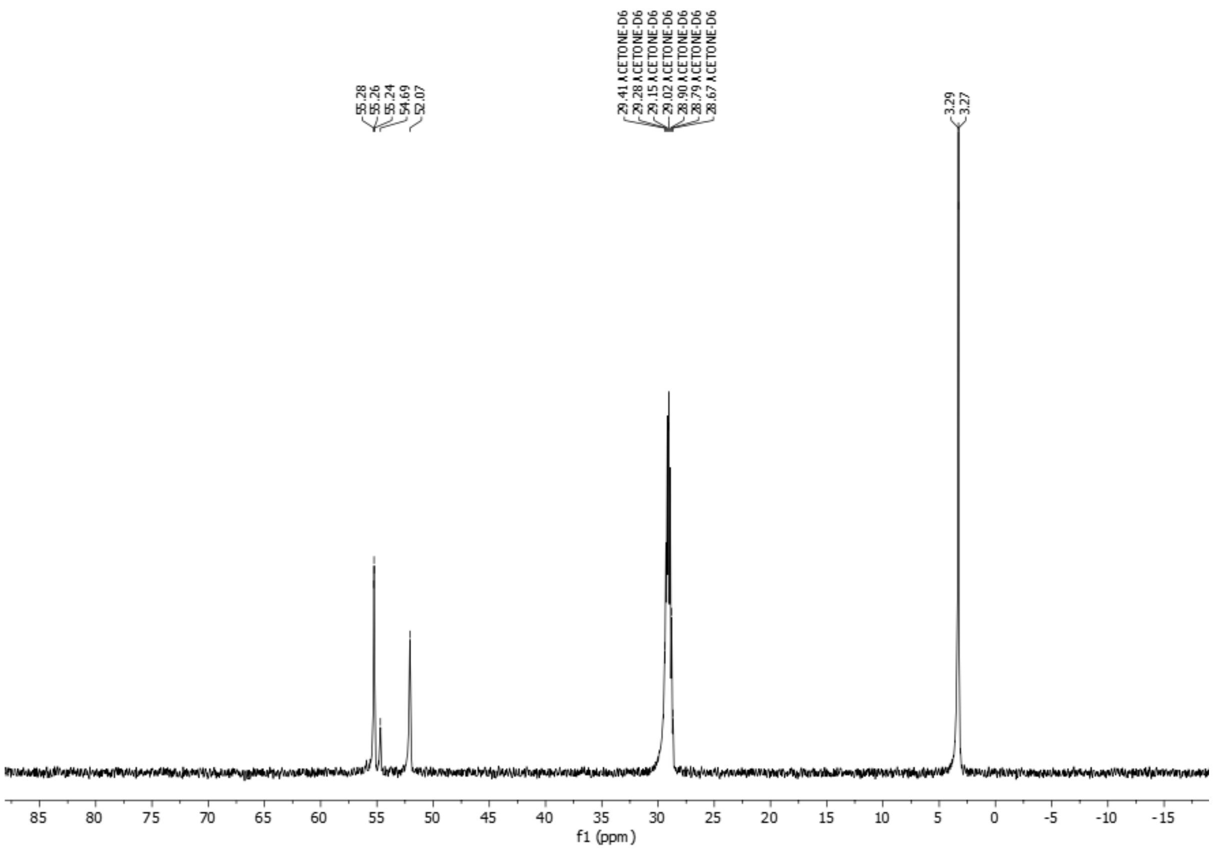

Figure S54. $^{13}C\{^{1}H\}$ NMR Spectrum of Me_4N**7** in $(CD_3)_2CO$

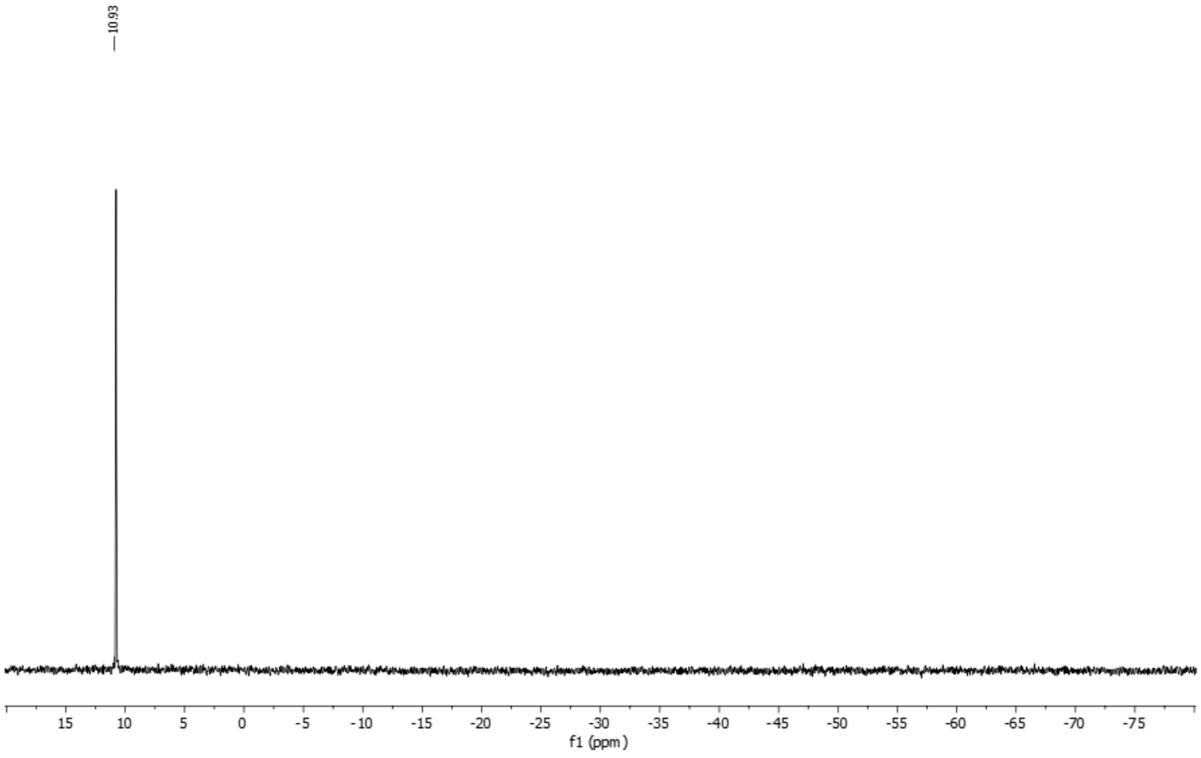

Figure S55. $^{29}Si\{^{1}H\}$ NMR Spectrum of Me_4N**7** in $(CD_3)_2CO$

NMR Spectra of [Me_4N][(1-N_3-C_2H_4-1,2-$C_2B_9H_{10}$)(1’,2’-$C_2B_9H_{11}$)-3,3’-Co(III)] (Me_4N**8**)

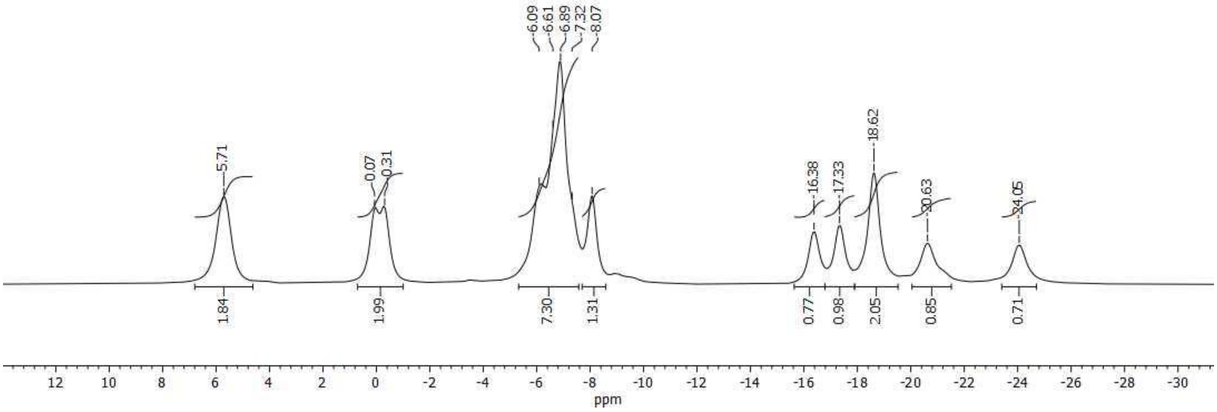

Figure S56. $^{11}B\{^{1}H\}$ NMR spectrum of Me_4N**8** in $(CD_3)_2CO$

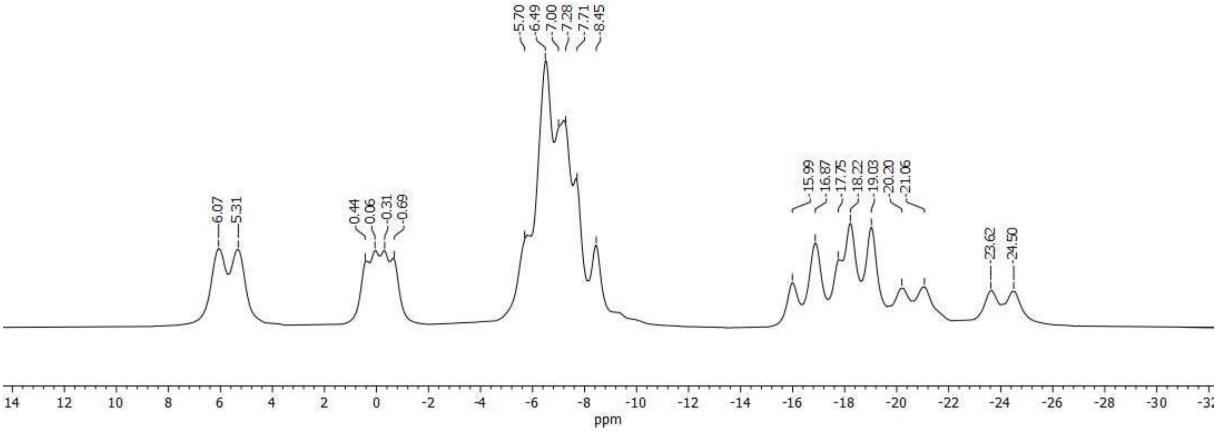

Figure S57. ^{11}B NMR spectrum of Me_4N**8** in $(CD_3)_2CO$

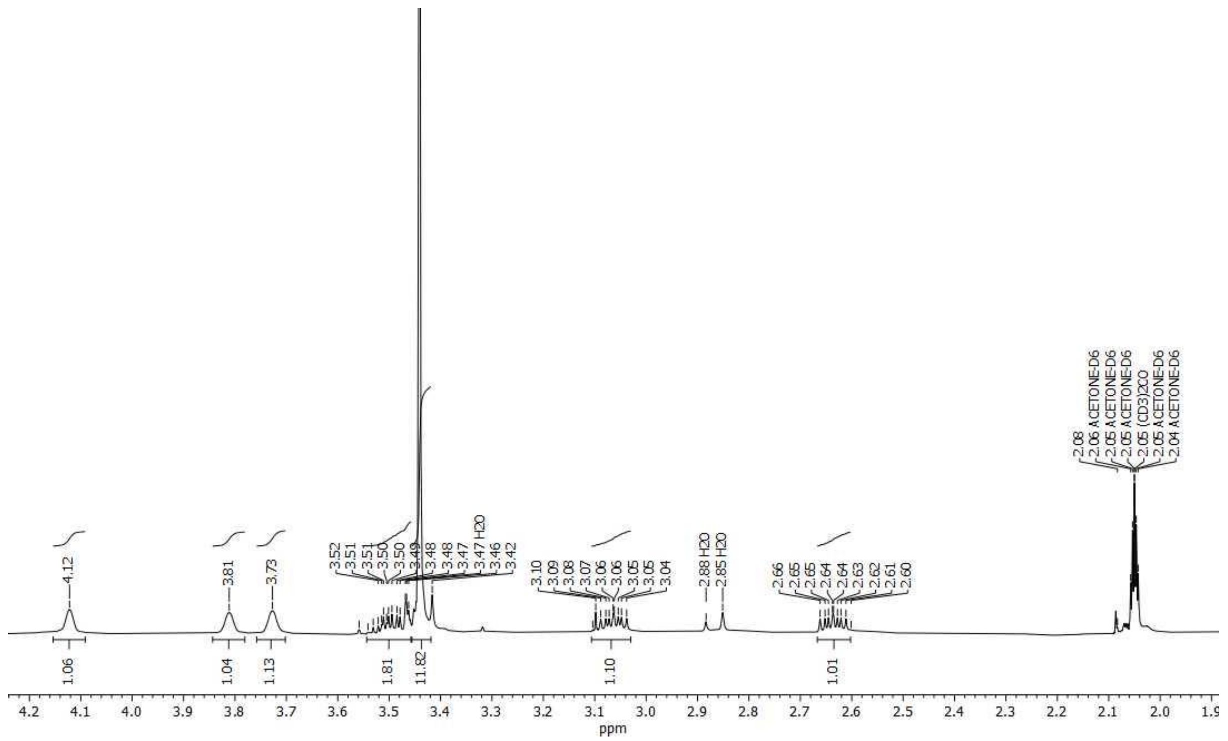

Figure S58. ^{1}H NMR spectrum of Me_4N**8** in $(CD_3)_2CO$

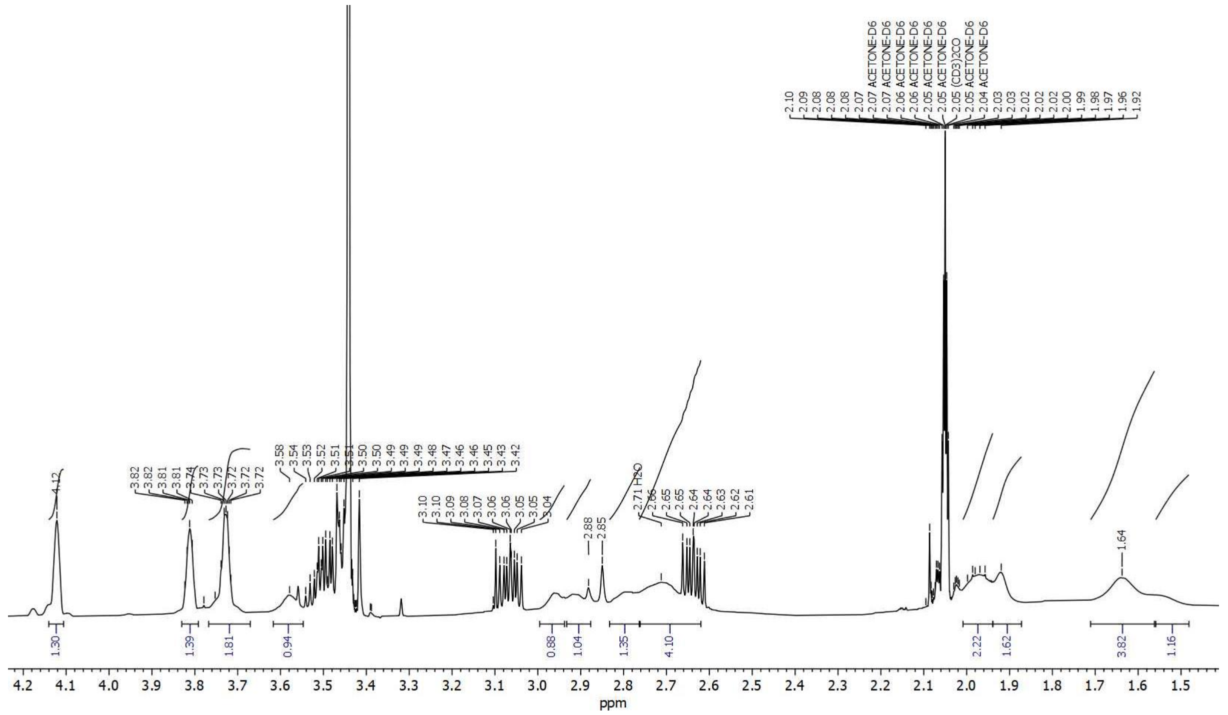

Figure S59. $^{1}H\{^{11}B\}$ NMR Spectrum of Me_4N**8** in $(CD_3)_2CO$

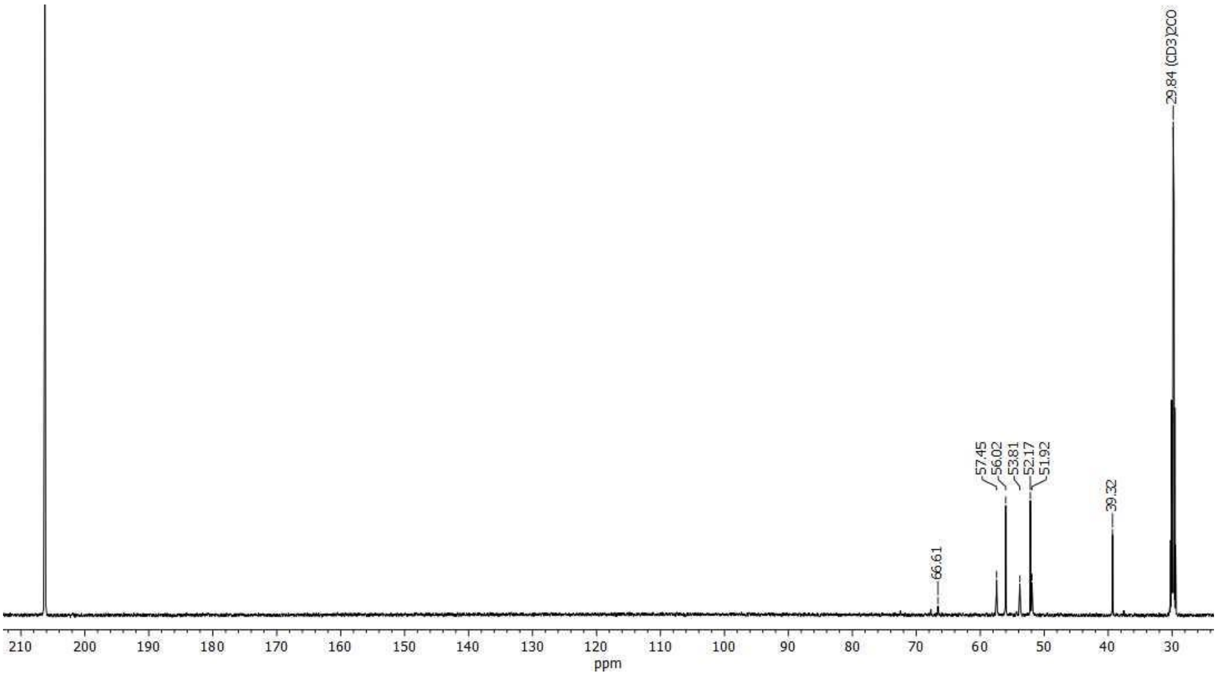

Figure S60. $^{13}C\{^1H\}$ NMR spectrum of Me_4N**8** in $(CD_3)_2CO$

NMR Spectra of [Me$_4$N][(1-N$_3$-C$_3$H$_6$-1,2-C$_2$B$_9$H$_{10}$)(1',2'-C$_2$B$_9$H$_{11}$)-3,3'-Co(III)] (Me$_4$N9)

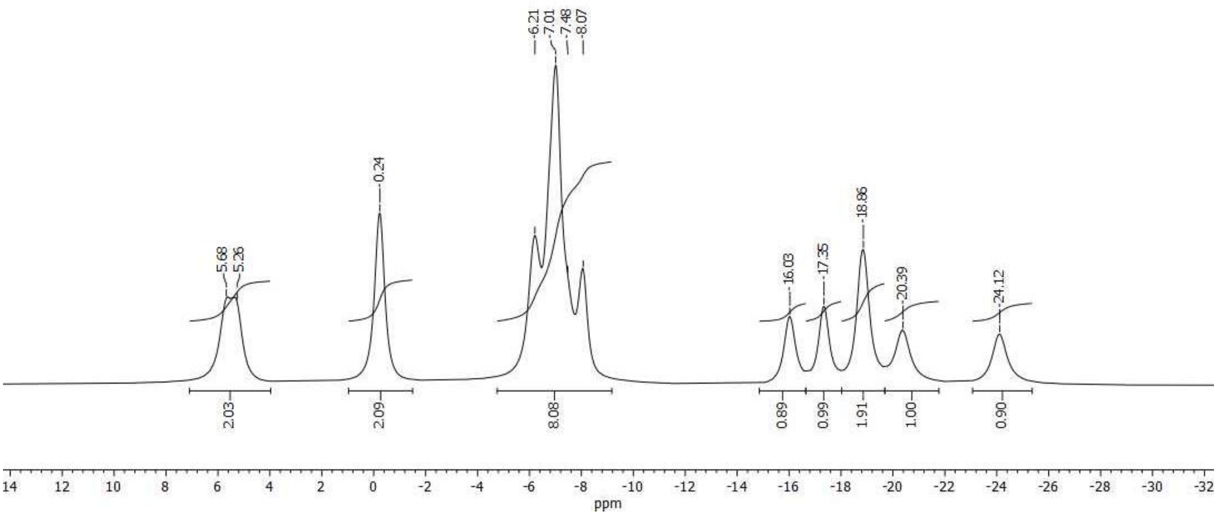

Figure S61. $^{11}B\ \{^1H\}$ NMR spectrum of Me$_4$N**9** in $(CD_3)_2CO$

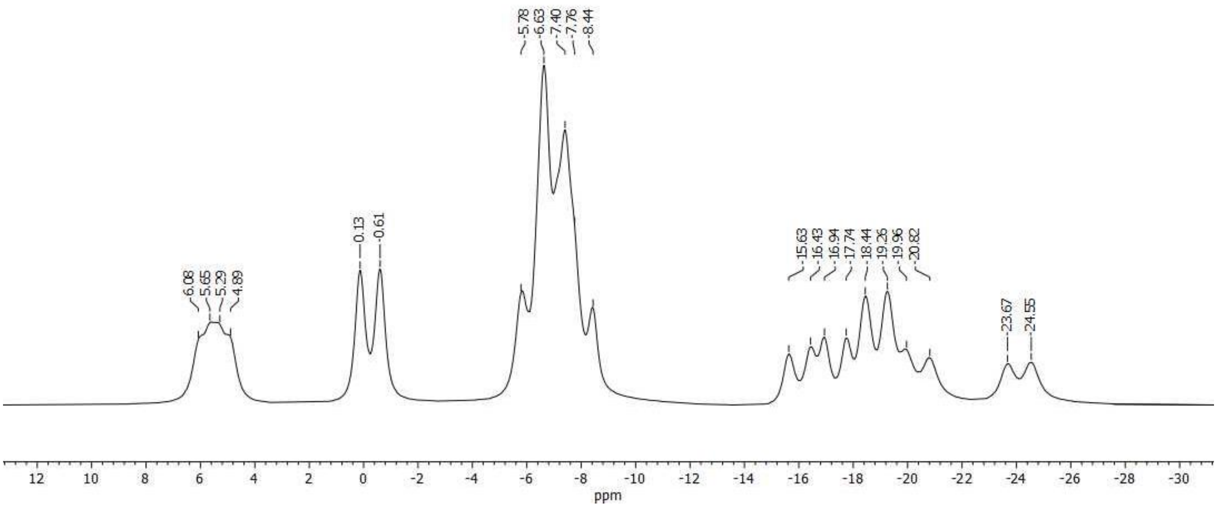

Figure S62. ^{11}B NMR spectrum of Me$_4$N**9** in $(CD_3)_2CO$

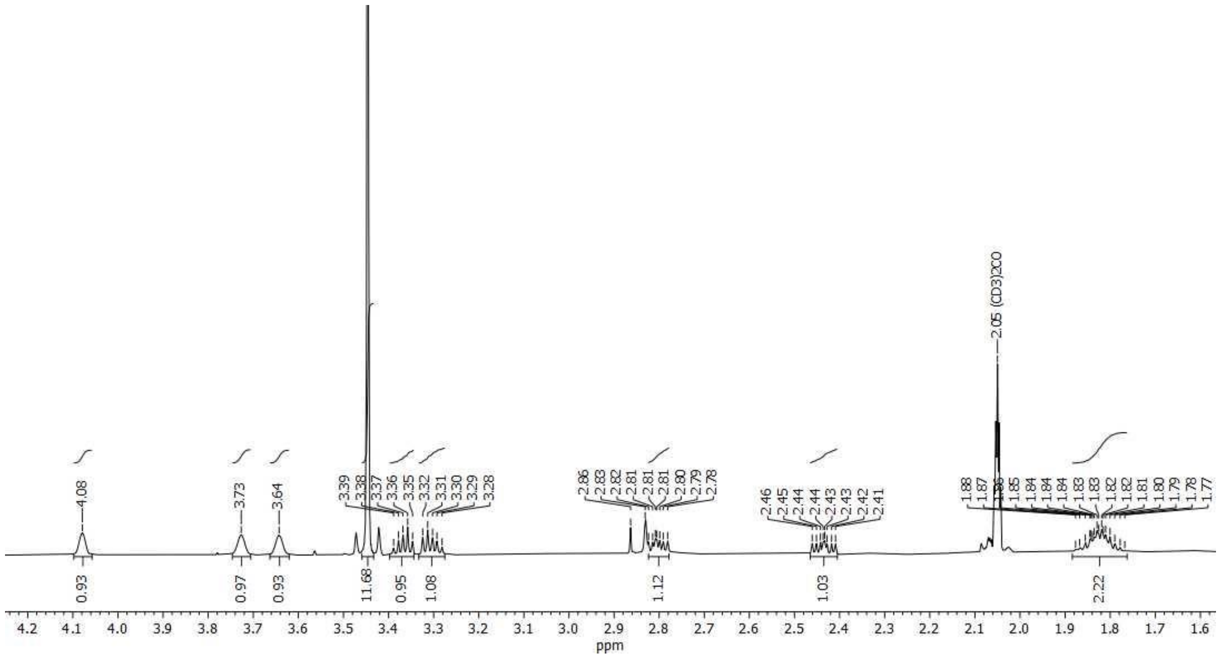

Figure S63. ^{1}H NMR spectrum of Me_4N**9** in $(CD_3)_2CO$

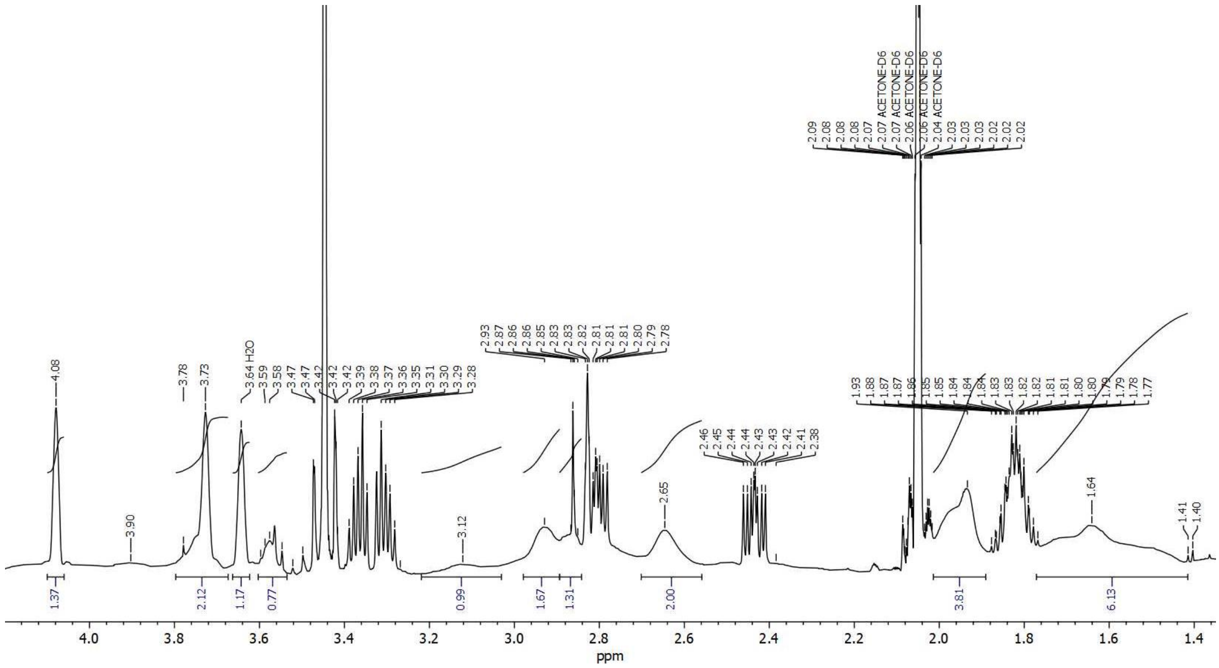

Figure S64. ^{1}H{^{11}B} NMR Spectrum of Me_4N**9** in $(CD_3)_2CO$

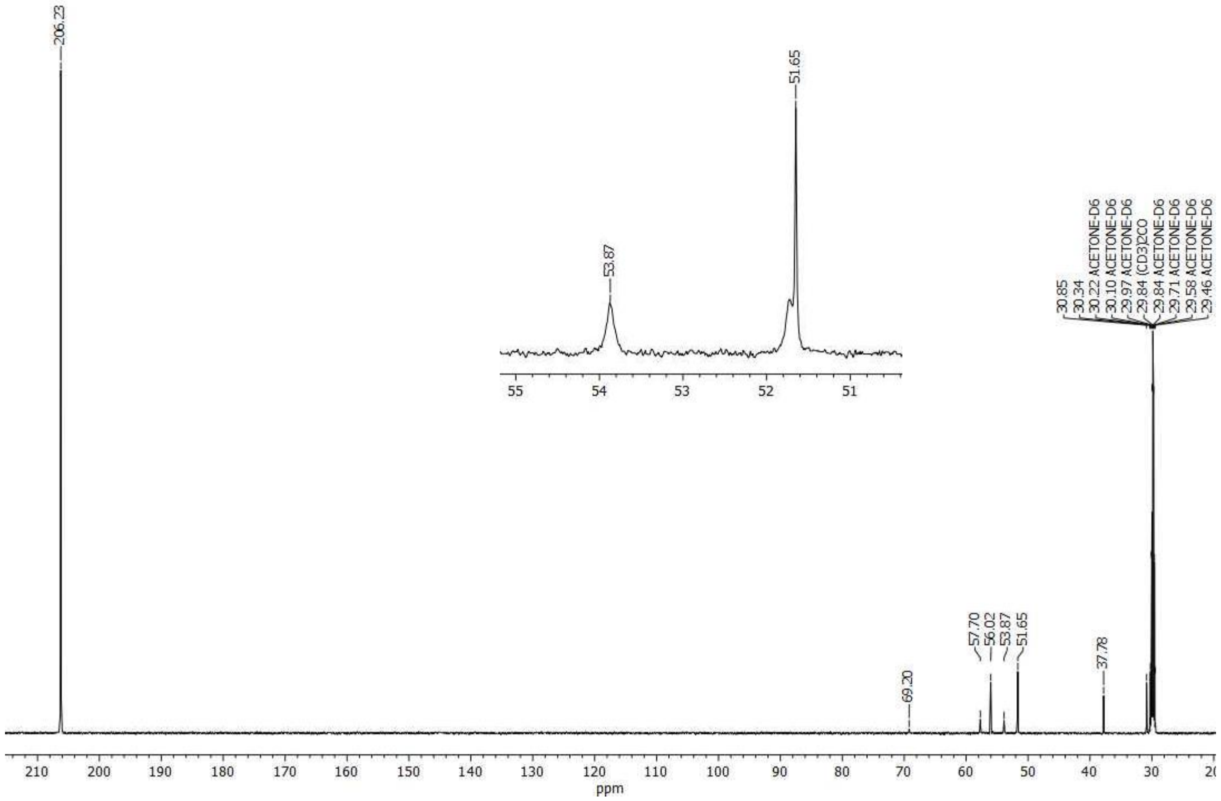

Figure S65. $^{13}C\ \{^1H\}$ NMR spectrum of Me_4N**9** in $(CD_3)_2CO$

NMR Spectra of $[Me_4N][(1,1'-N_3-C_2H_4-1,2-C_2B_9H_{10})_2-3,3'-Co(III)]$ (Me_4N**10**)

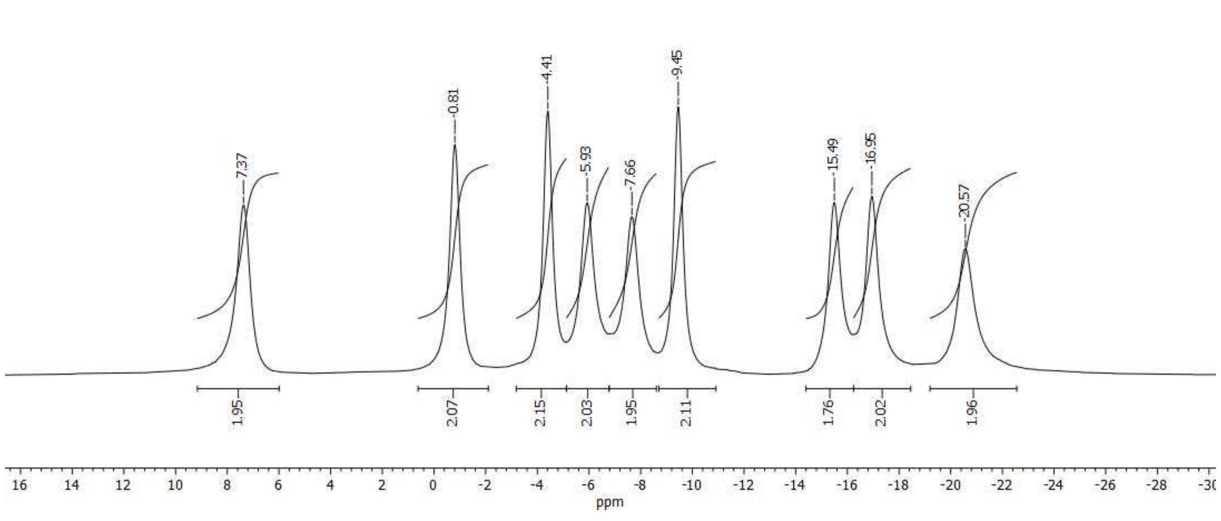

Figure S66. $^{11}B\ \{^1H\}$ NMR spectrum of Me_4N**10** in $(CD_3)_2CO$

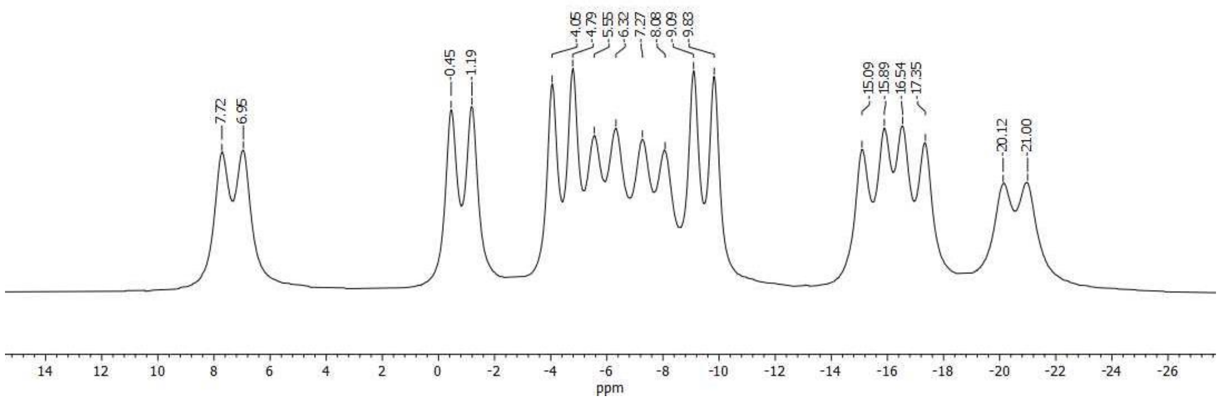

Figure S67. ^{11}B NMR spectrum of Me_4N**10** in $(CD_3)_2CO$

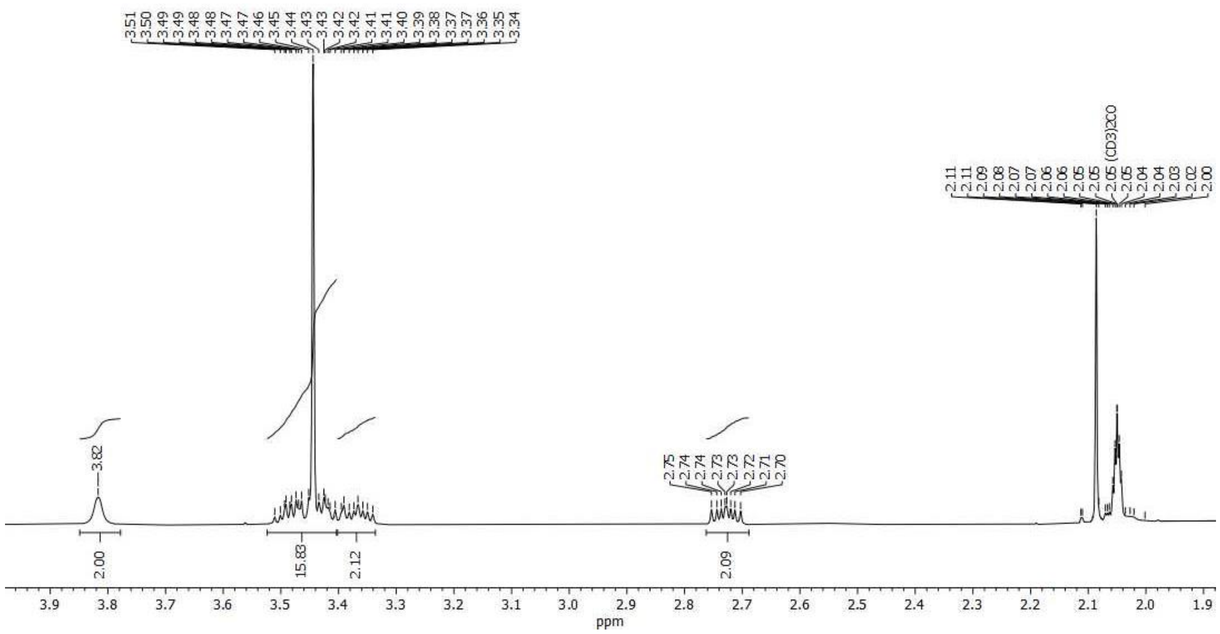

Figure S68. ^{1}H NMR spectrum of Me_4N**10** in $(CD_3)_2CO$

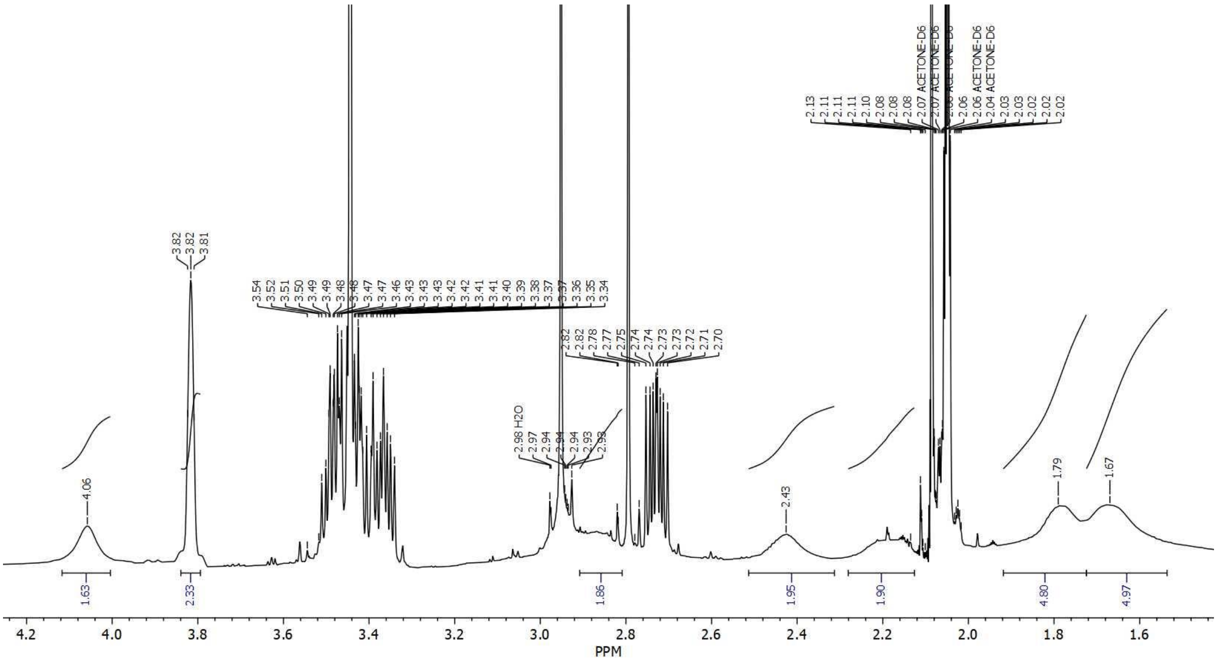

Figure S69. $^{1}H\{^{11}B\}$ NMR Spectrum of Me_4N**10** in $(CD_3)_2CO$

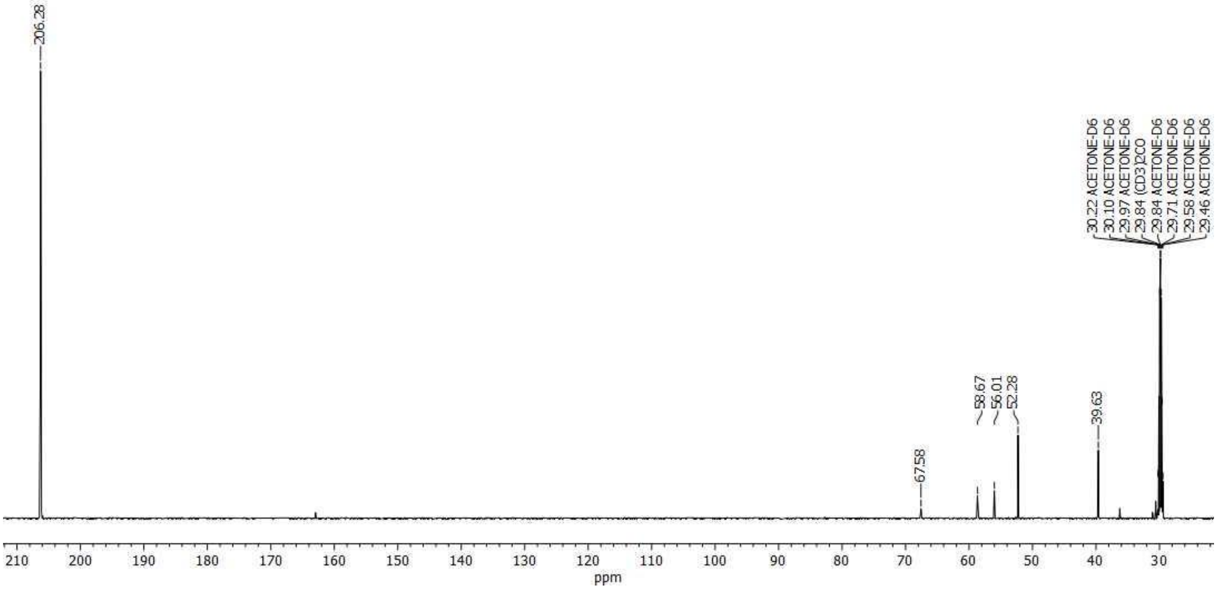

Figure S70. $^{13}C\{^{1}H\}$ NMR spectrum of Me_4N**10** in $(CD_3)_2CO$

NMR Spectra of [Me_4N][(1-(4-Ph-Triazolyl)-C_2H_4-1,2-$C_2B_9H_{10}$)(1',2'-$C_2B_9H_{11}$)-3,3'-Co(III)] (Me_4N**11**)

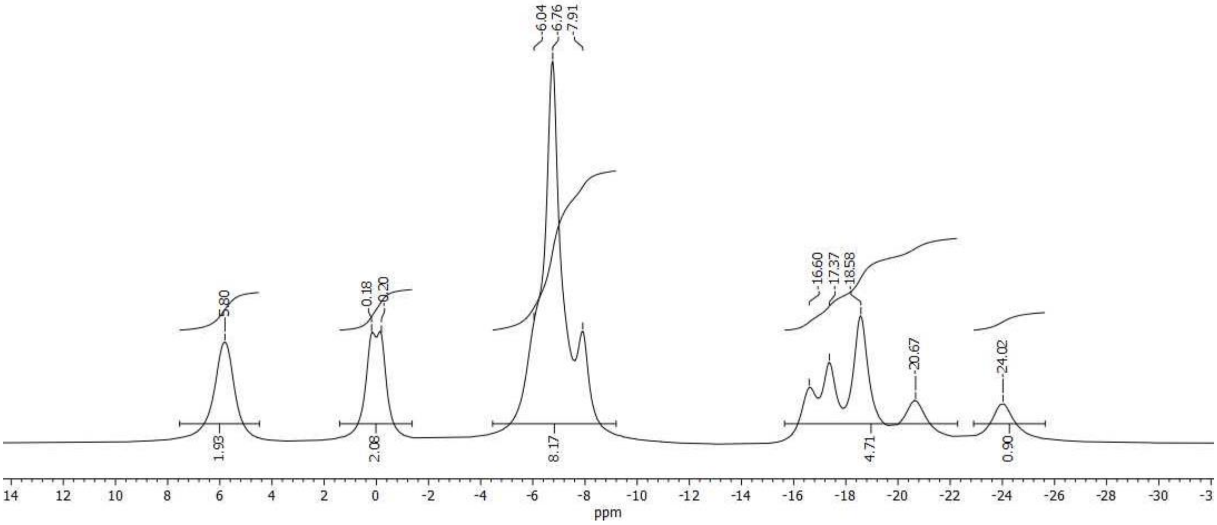

Figure S71. $^{11}B\{^{1}H\}$ NMR spectrum of Me_4N**11** in $(CD_3)_2CO$

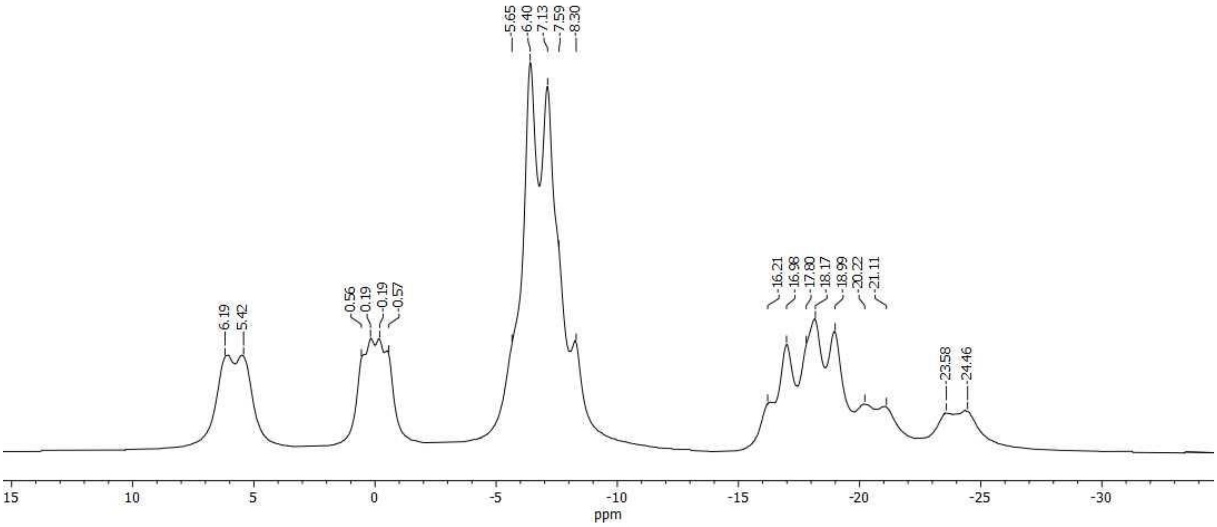

Figure S72. ^{11}B NMR spectrum of Me_4N**11** in $(CD_3)_2CO$

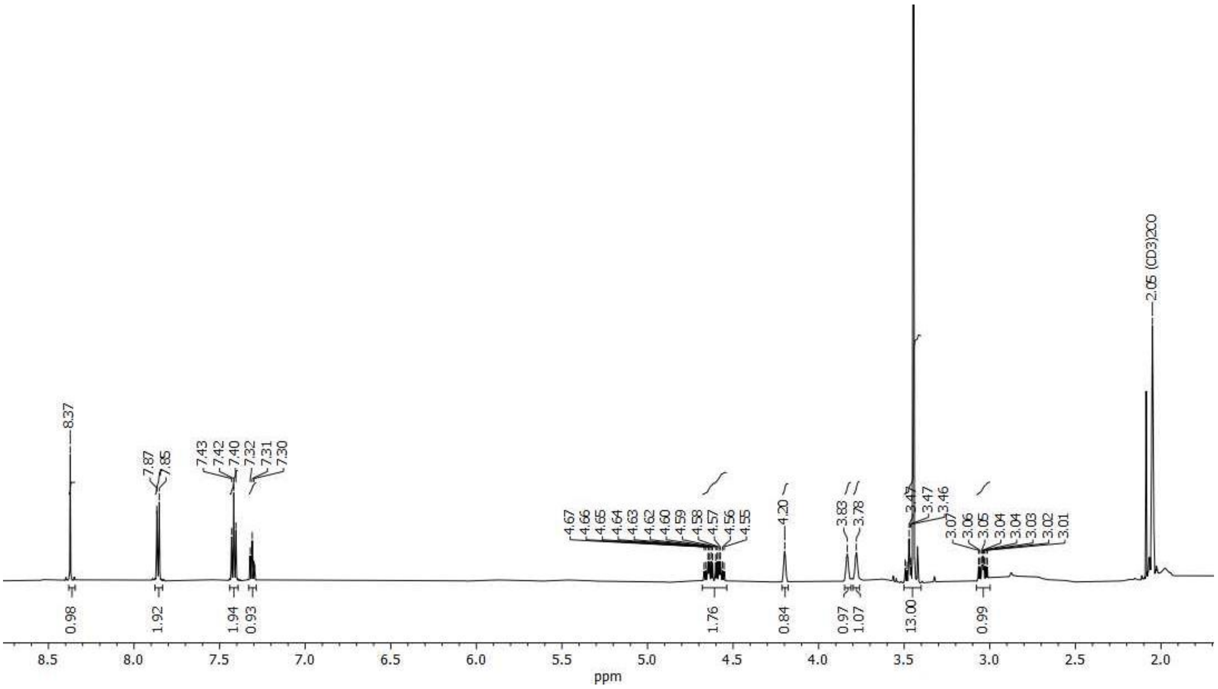

Figure S73. ^{1}H NMR spectrum of Me_4N**11** in $(CD_3)_2CO$

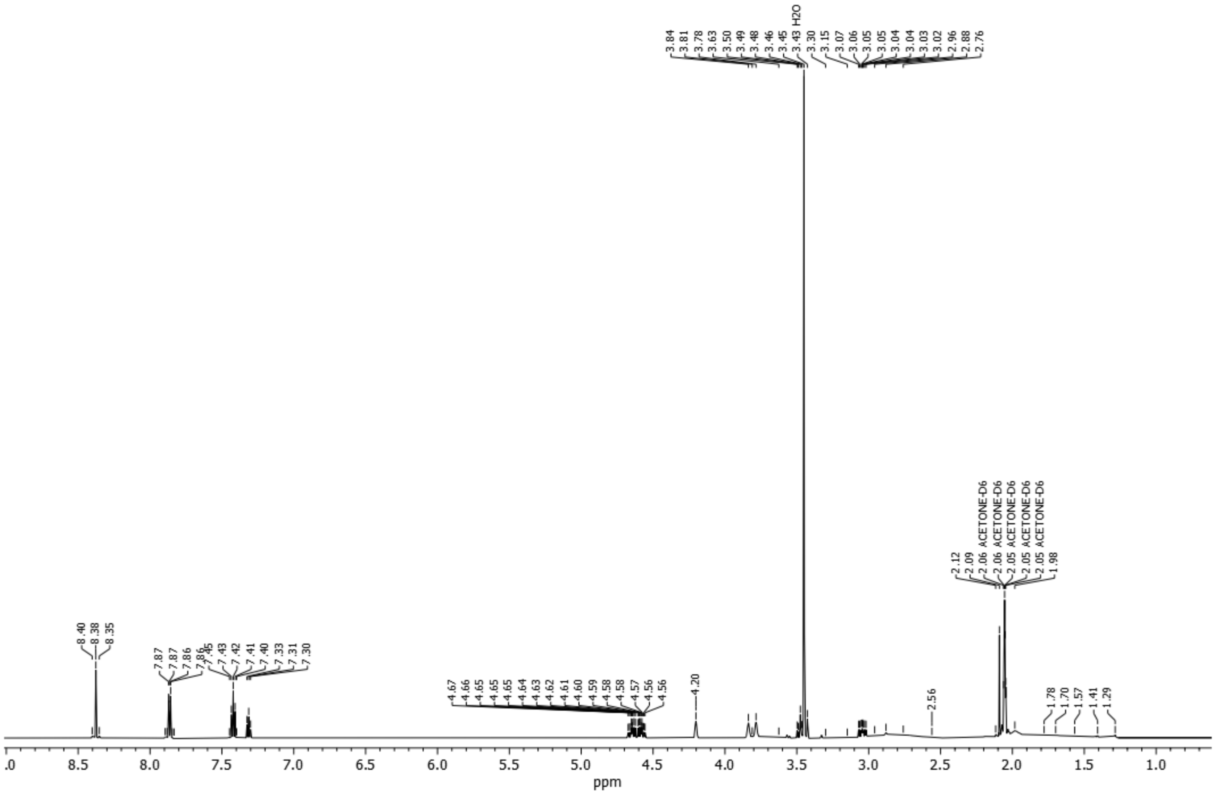

Figure S74. $^{1}H\{^{11}B\}$ NMR Spectrum of Me_4N**11** in $(CD_3)_2CO$

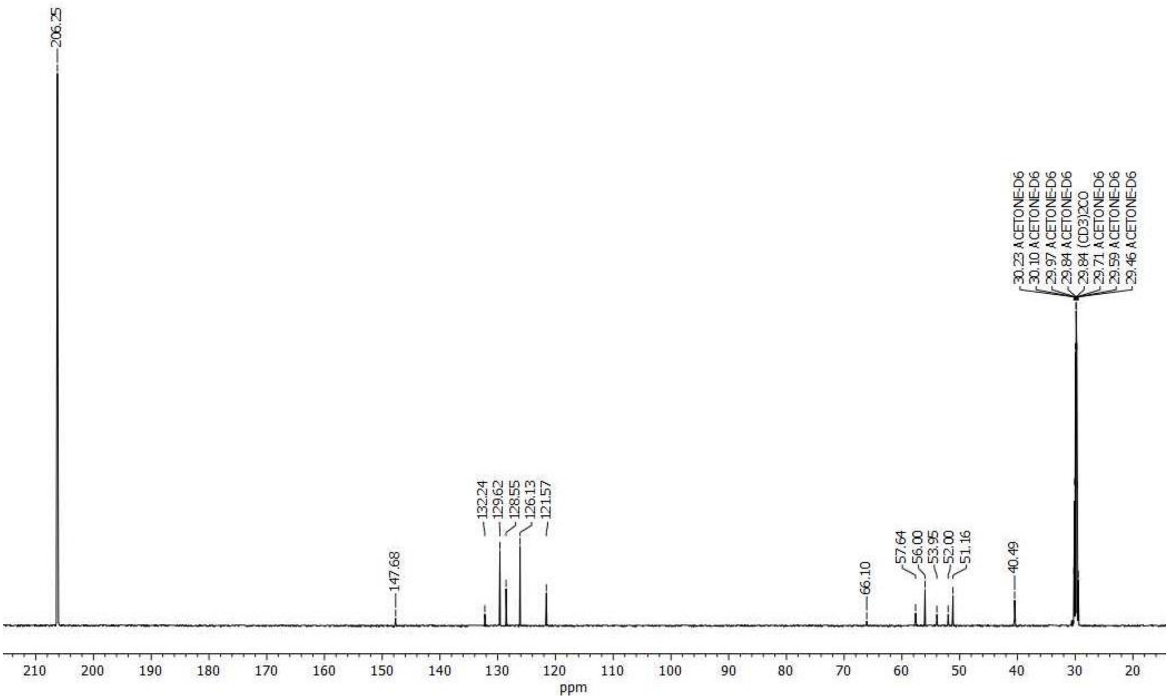

Figure S75. $^{13}C\{^{1}H\}$ NMR spectrum of Me_4N**11** in $(CD_3)_2CO$

NMR Spectra of [Me$_4$N][(1-(4-Ph-Triazolyl)-C$_3$H$_6$-1,2-C$_2$B$_9$H$_{10}$)(1',2'-C$_2$B$_9$H$_{11}$)-3,3'-Co(III)] (Me$_4$N12)

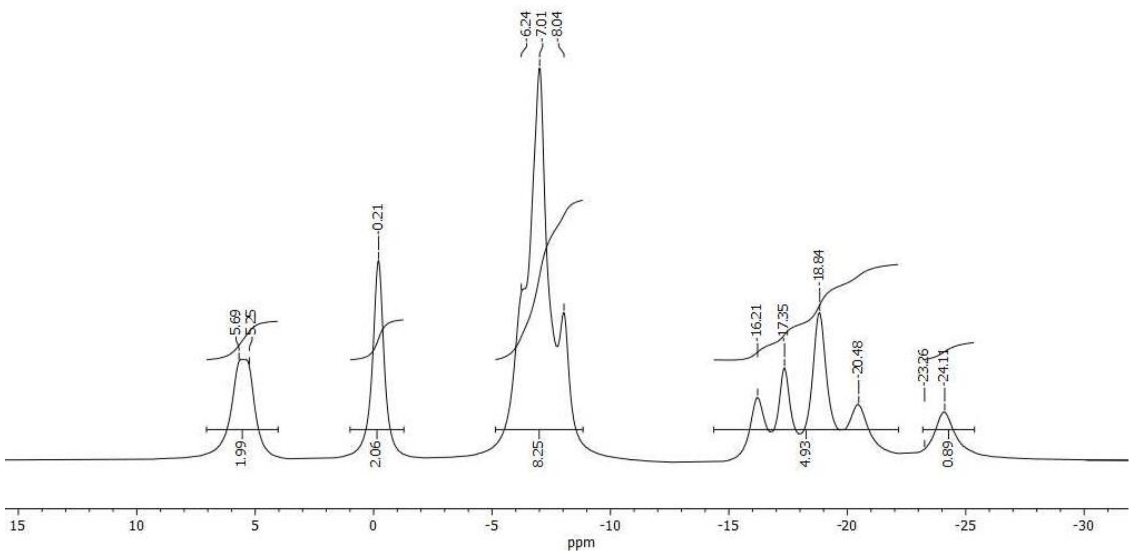

Figure S76. $^{11}B\{^1H\}$ NMR spectrum of Me$_4$N**12** in $(CD_3)_2CO$

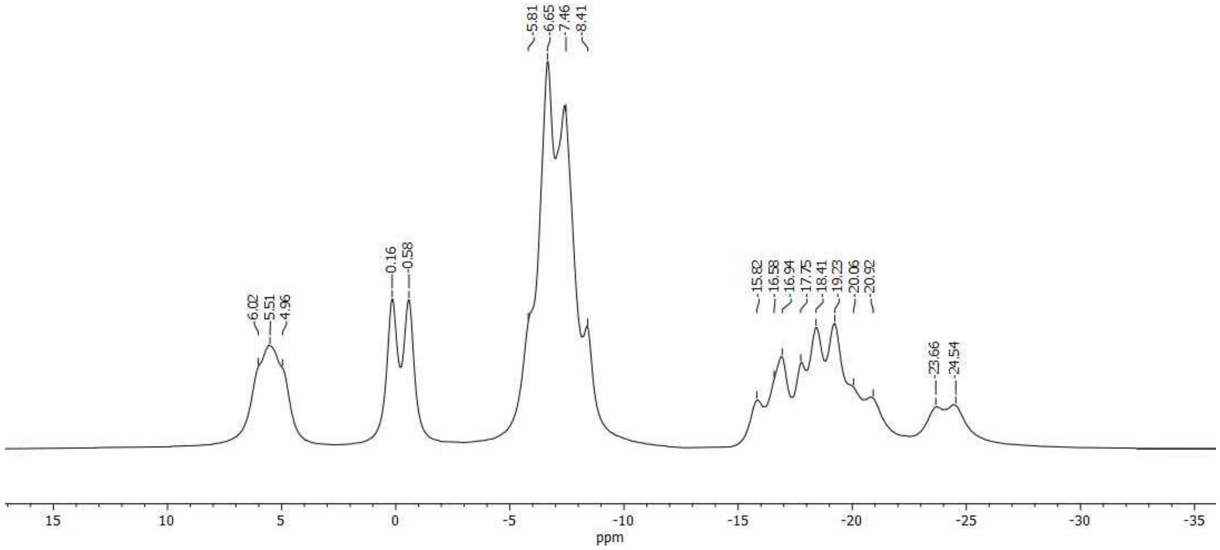

Figure S77. ^{11}B NMR spectrum of Me$_4$N**12** in $(CD_3)_2CO$

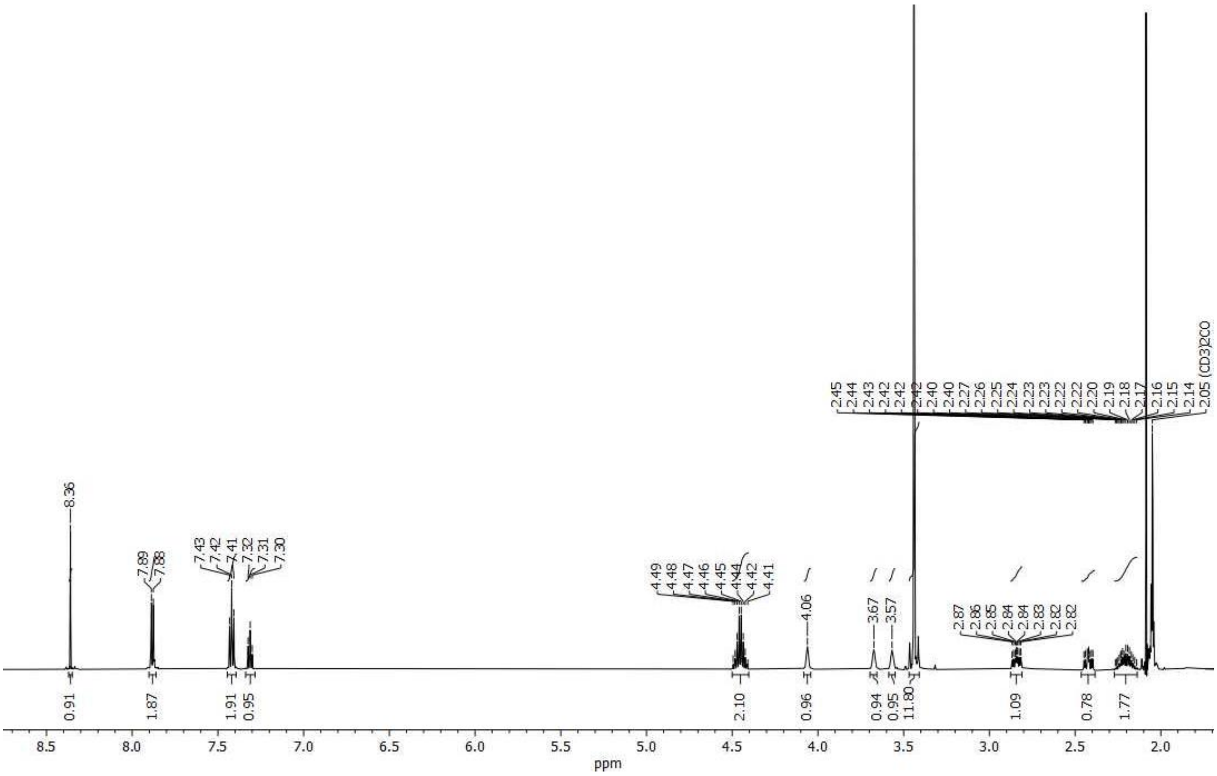

Figure S78. ^{1}H NMR spectrum of Me_4N**12** in $(CD_3)_2CO$

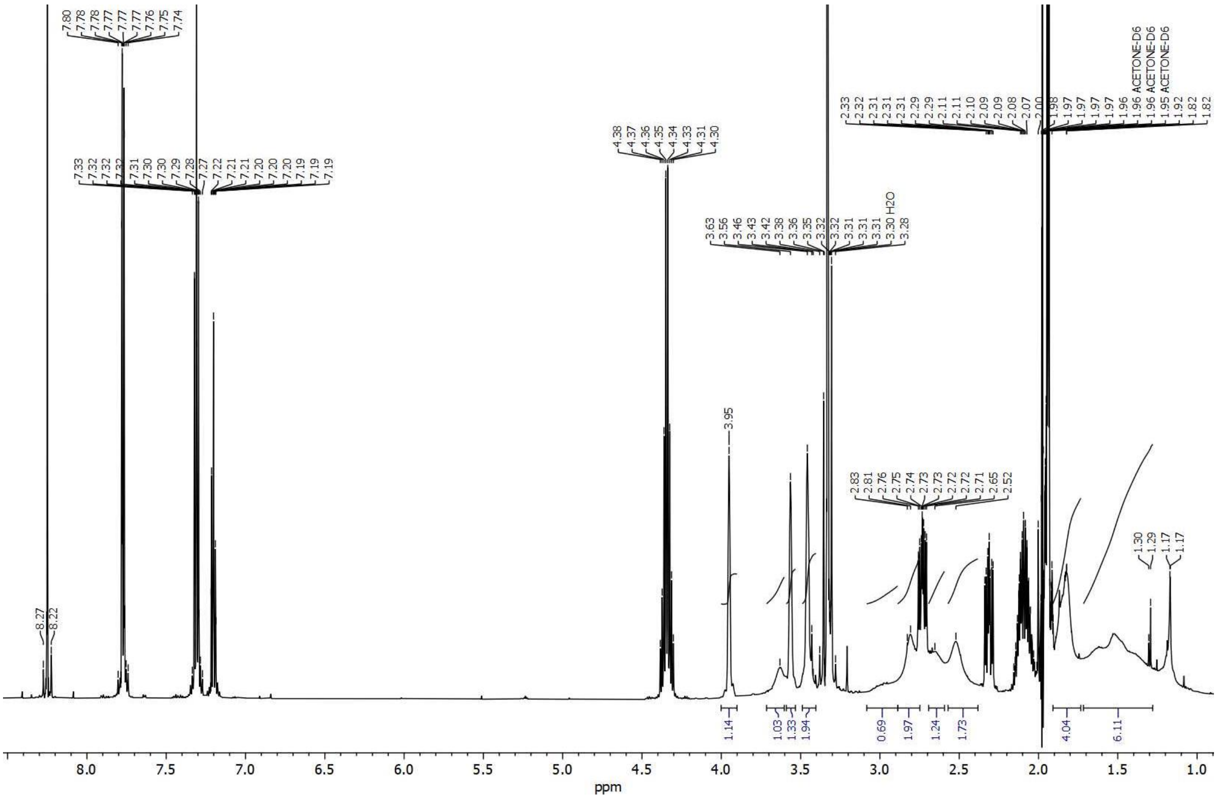

Figure S79. $^{1}H\{^{11}B\}$ NMR spectrum of Me_4N**12** in $(CD_3)_2CO$

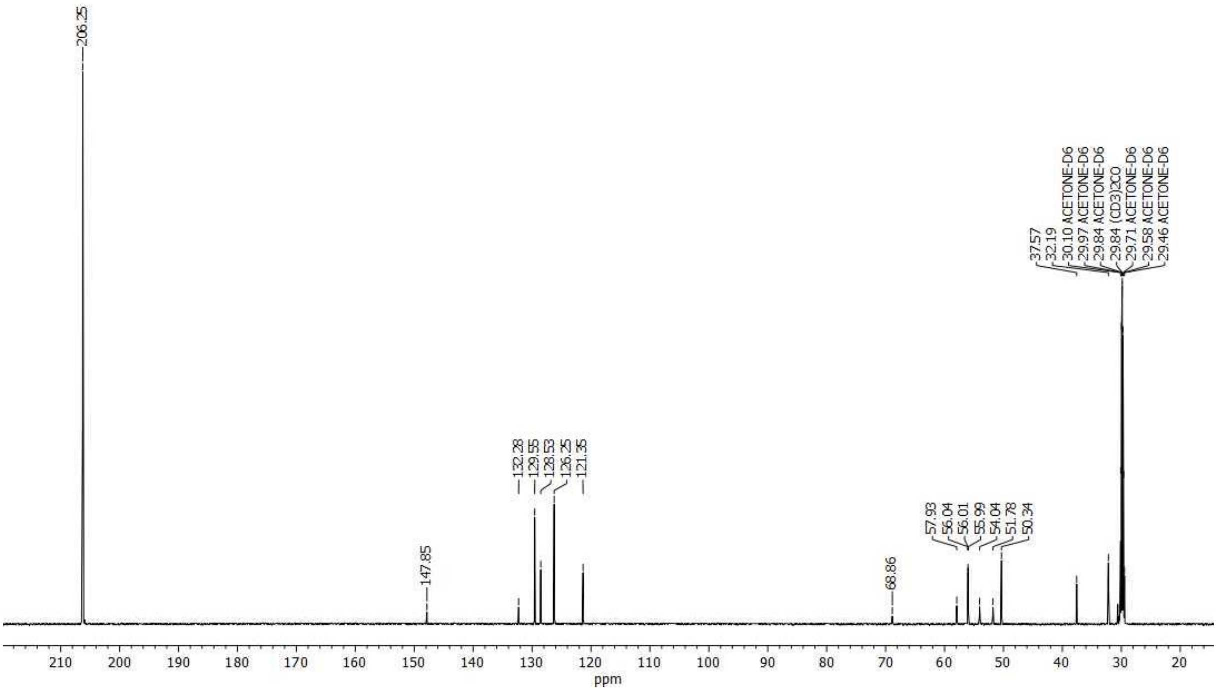

Figure S80. $^{13}C\{^{1}H\}$ NMR spectrum of Me_4N**12** in $(CD_3)_2CO$

NMR Spectra of $[Me_4N][(1\text{-}(CO)N_3\text{-}1,2\text{-}C_2B_9H_{10})(1',2'\text{-}C_2B_9H_{11})\text{-}3,3'\text{-}Co(III)]$ (Me_4N**13**)

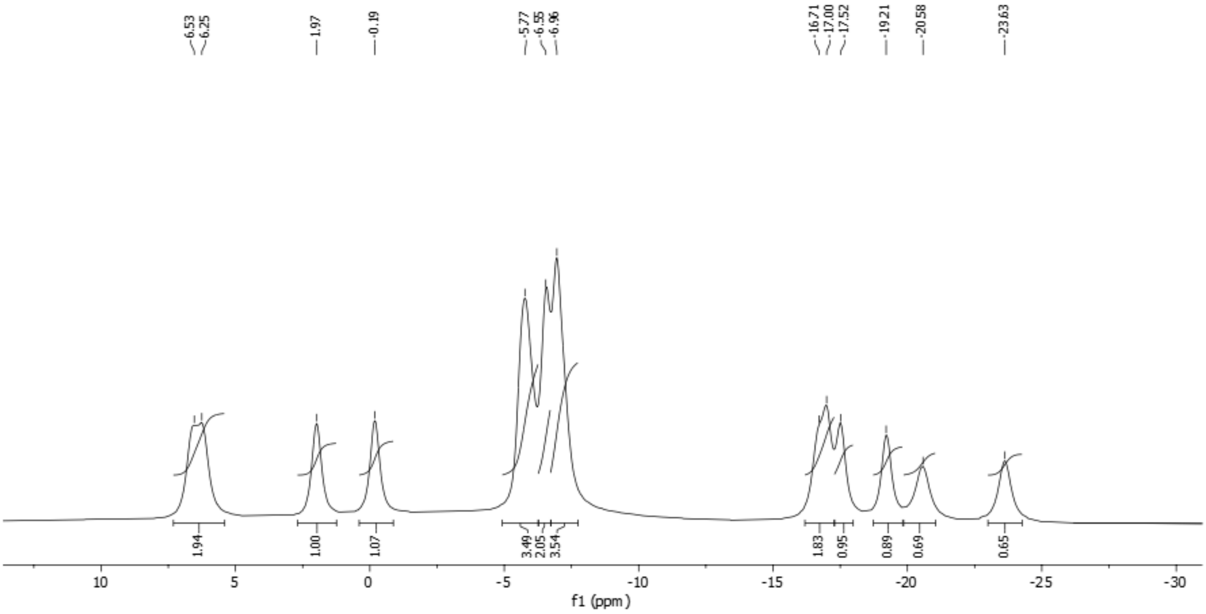

Figure S81. $^{11}B\{^{1}H\}$ NMR spectrum of Me_4N**13** in $(CD_3)_2CO$

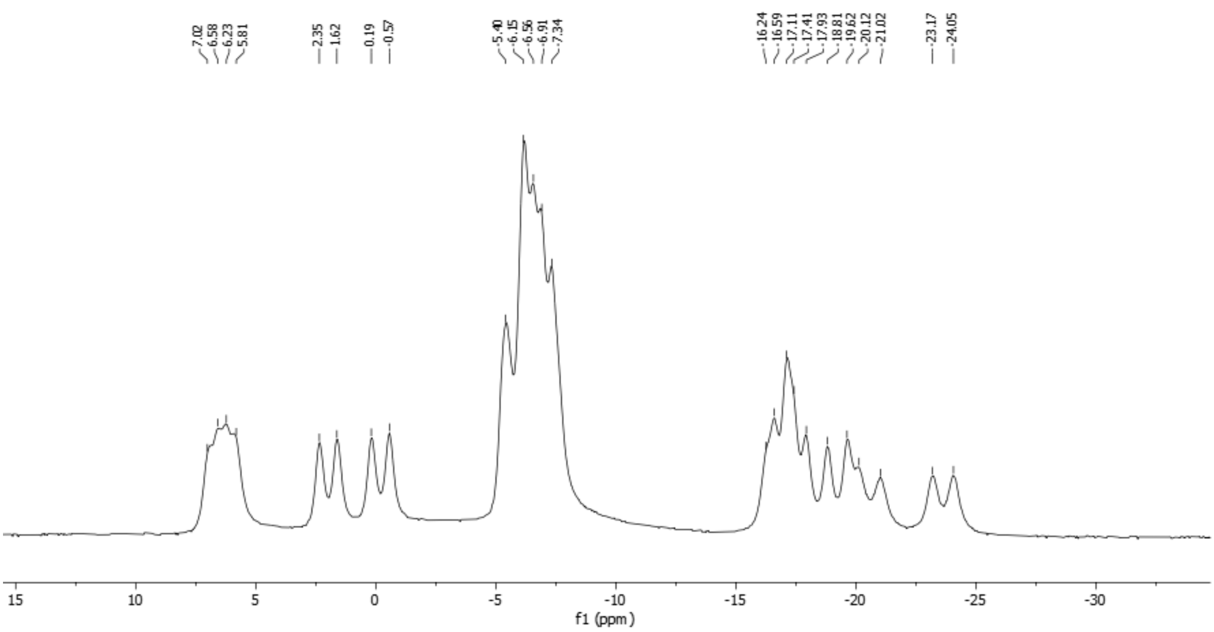

Figure S82. ^{11}B NMR spectrum of Me_4N**13** in $(CD_3)_2CO$

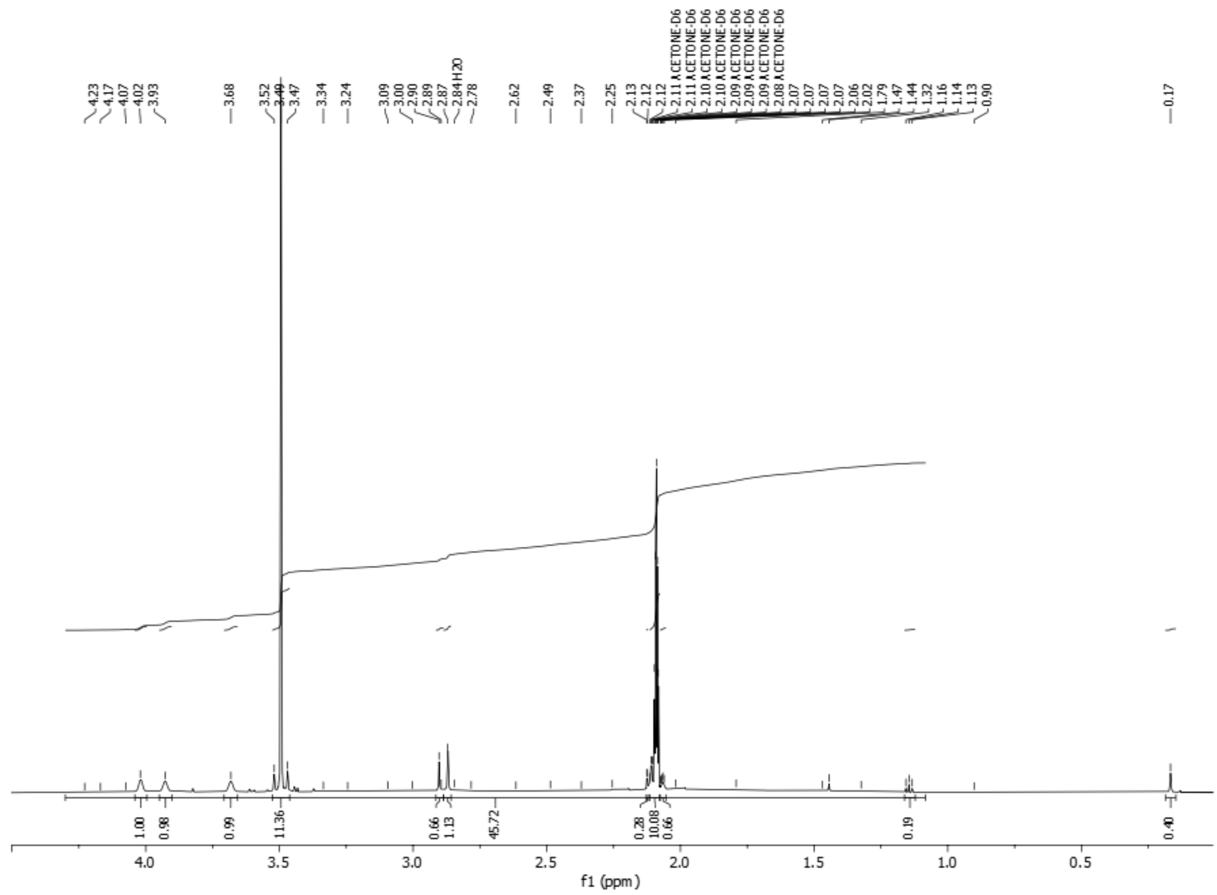

Figure S83. ^{1}H NMR Spectrum of Me_4N**13** in $(CD_3)_2CO$

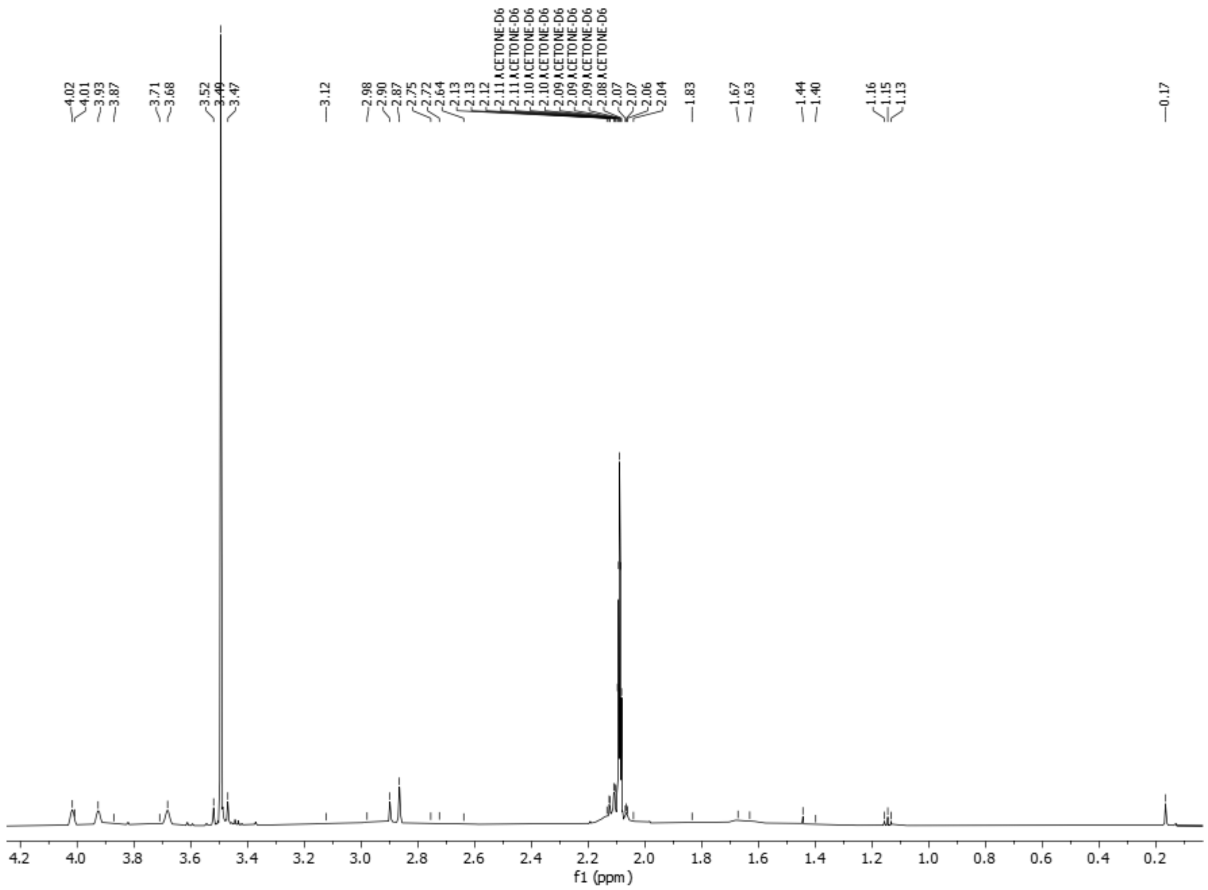

Figure S84. $^{1}H\{^{11}B\}$ NMR Spectrum of Me_4N**13** in $(CD_3)_2CO$

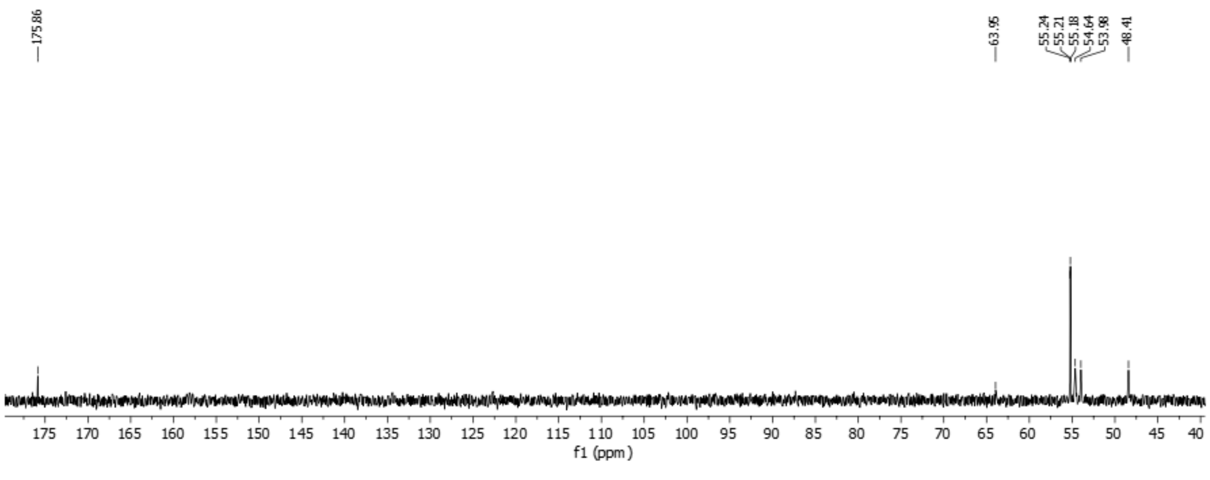

Figure S85. $^{13}C\{^{1}H\}$ NMR spectrum of Me_4N**13** in $(CD_3)_2CO$

III. HR-MS SPECTRA

HRMS Spectrum of $[(1,2-C_2B_9H_{11})_2-3,3'-Co(III)]^-$ (1^-)

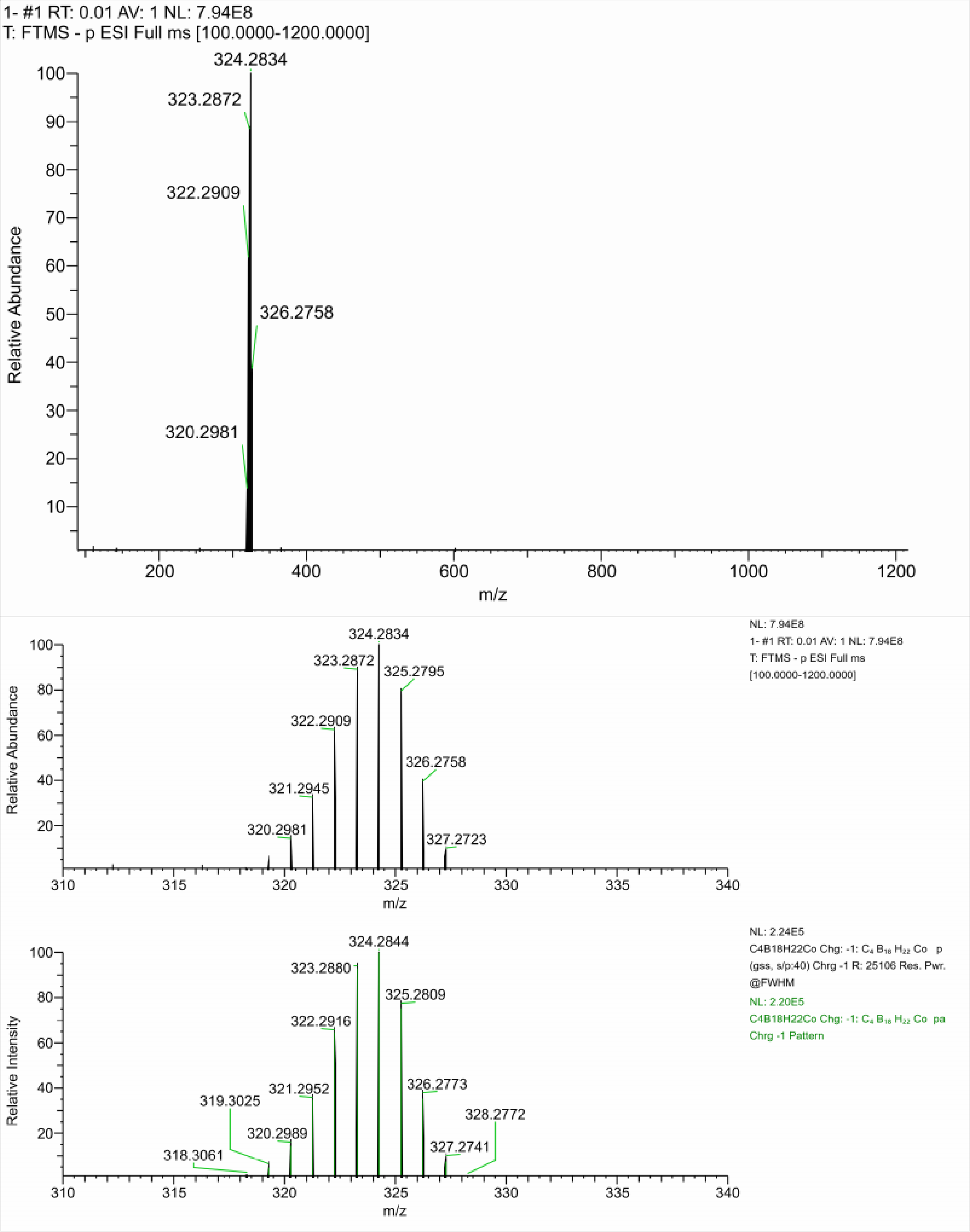

Figure S86. HRMS spectrum of **1**$^-$ with a calculated isotopic pattern.

HRMS Spectrum of [(1-N_3-1,2-$C_2B_9H_{10}$)(1',2'-$C_2B_9H_{11}$)-3,3'-Co(III)]$^-$ (2^-)

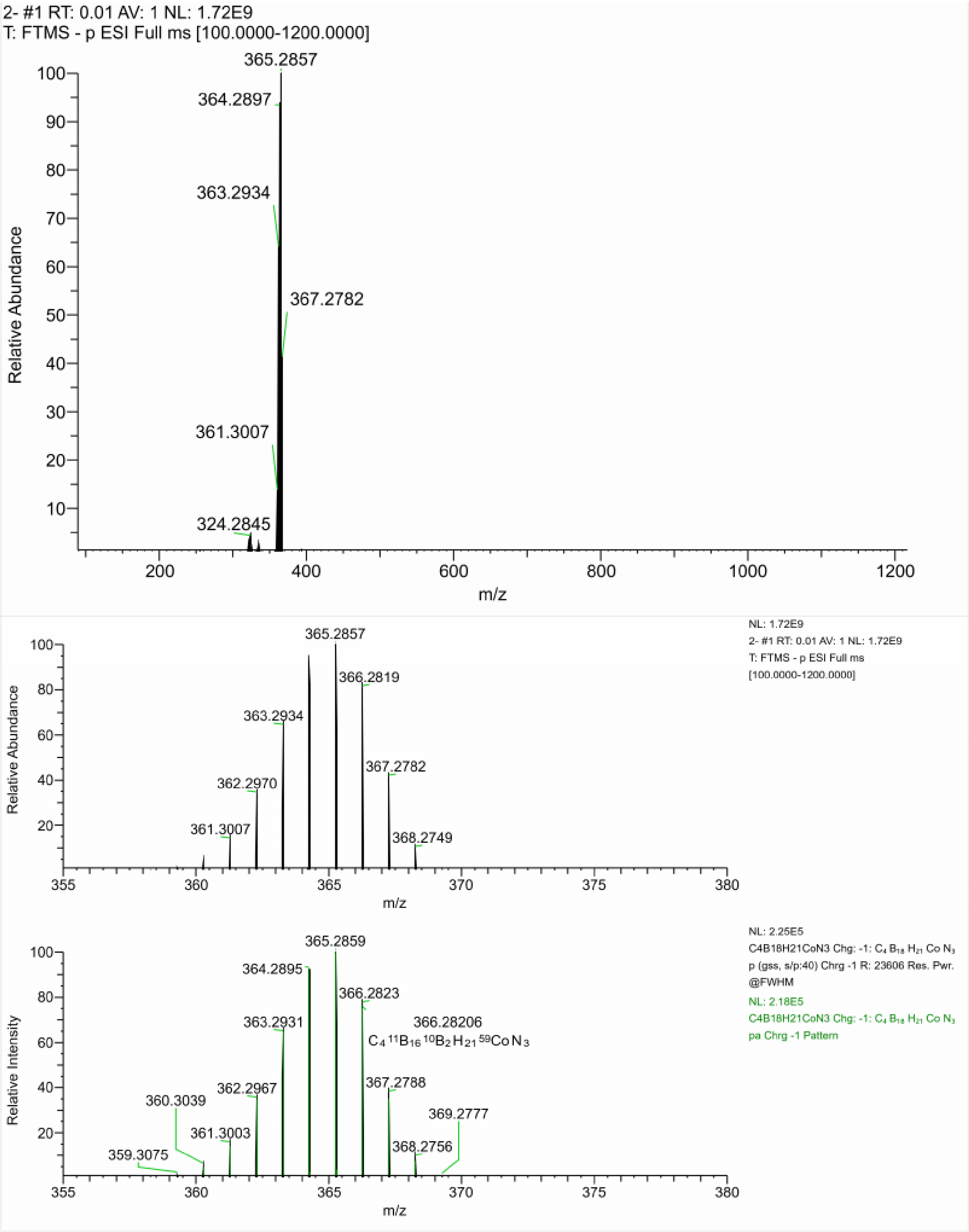

Figure S87. HRMS spectrum of **2**$^-$ with a calculated isotopic pattern.

HRMS Spectrum of $[(1,1'\text{-}N_3\text{-}1,2\text{-}C_2B_9H_{10})_2\text{-}3,3'\text{-}Co(III)]^-$ (3^-)

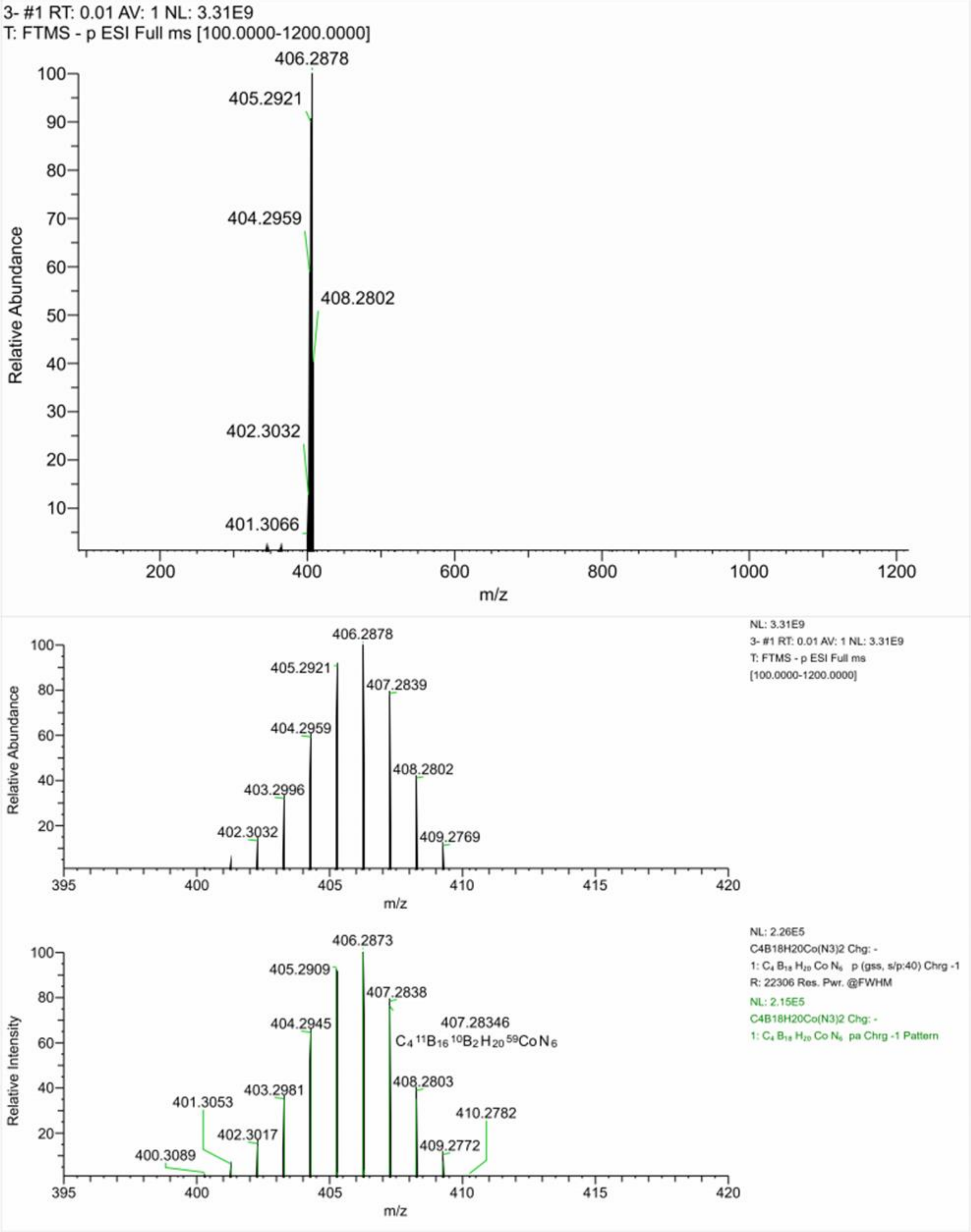

Figure S88. HRMS spectrum of **3**$^-$ with a calculated isotopic pattern.

HRMS Spectrum of $[(1\text{-}H_2N\text{-}1,2\text{-}C_2B_9H_{10})(1',2'\text{-}C_2B_9H_{11})\text{-}3,3'\text{-}Co(III)]^-$ (4^-)

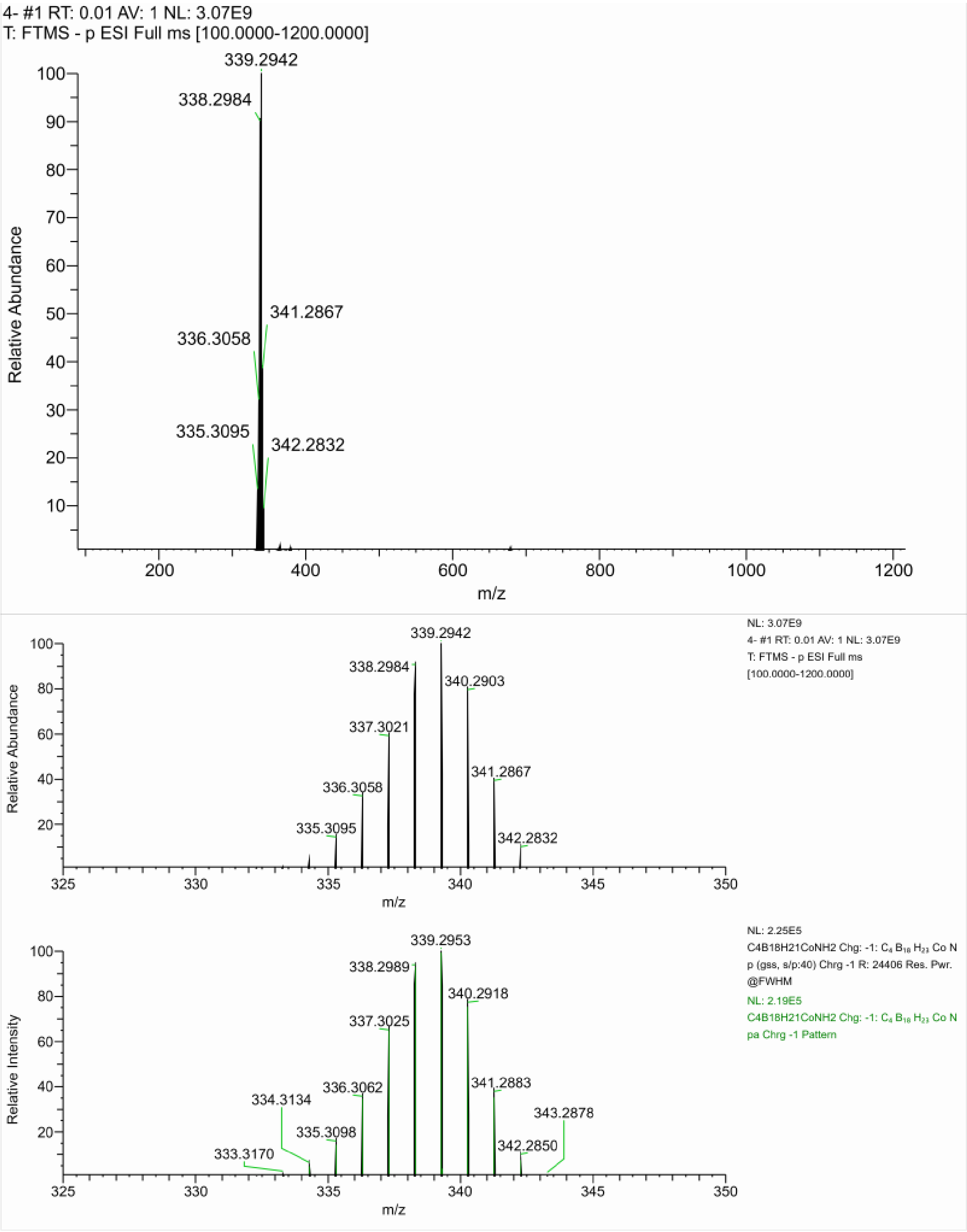

Figure S89. HRMS spectrum of $\mathbf{4^-}$ with a calculated isotopic pattern.

HRMS Spectrum of $[(1,1'\text{-}NH_2\text{-}1,2\text{-}C_2B_9H_{10})_2\text{-}3,3'\text{-}Co(III)]^-$ (5^-)

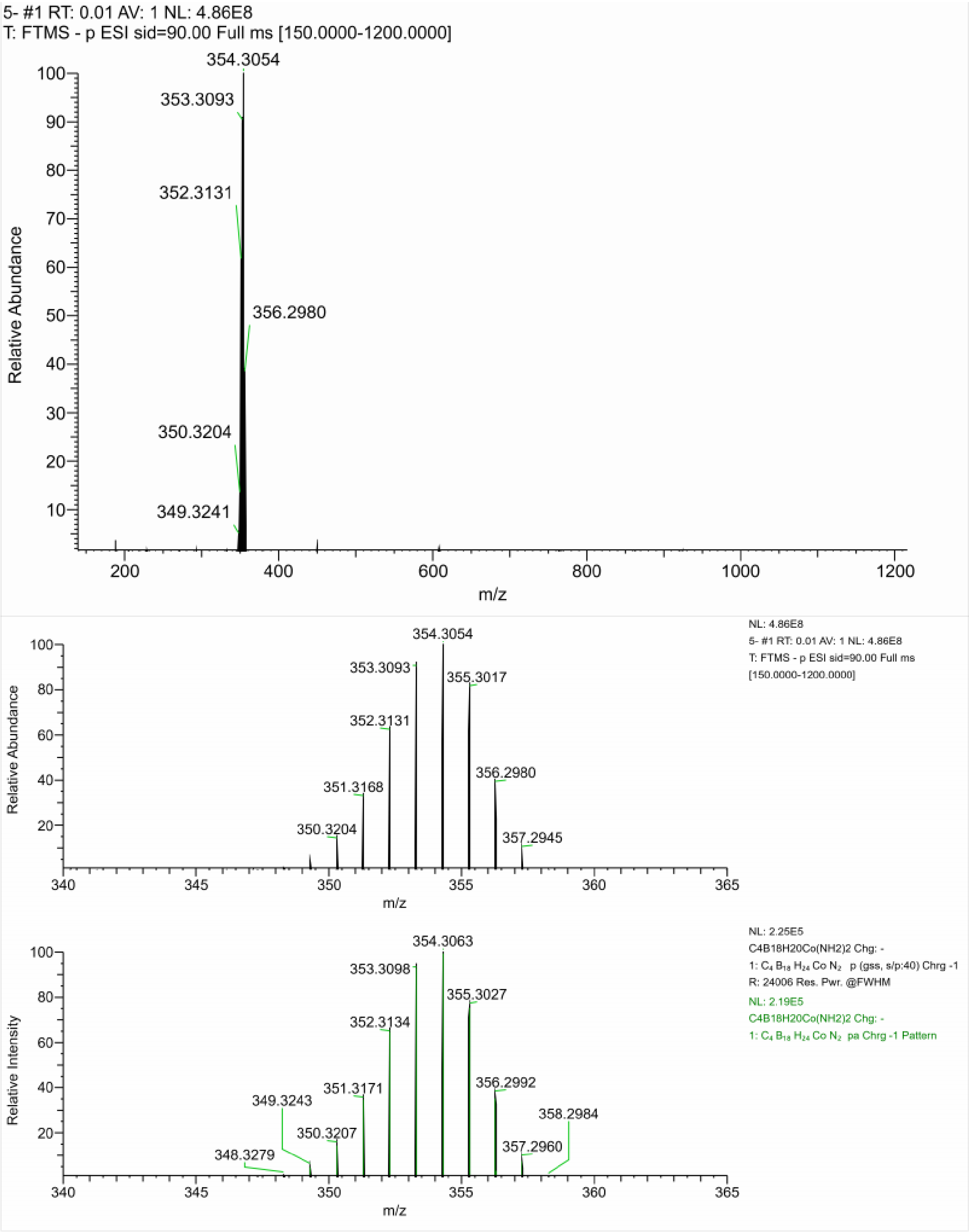

Figure S90. HRMS spectrum of **5**$^-$ with a calculated isotopic pattern.

HRMS Spectrum of [(1-Me$_3$Si-1,2-C$_2$B$_9$H$_{10}$)(1',2'-C$_2$B$_9$H$_{11}$)-3,3'-Co(III)]$^-$ (6$^-$)

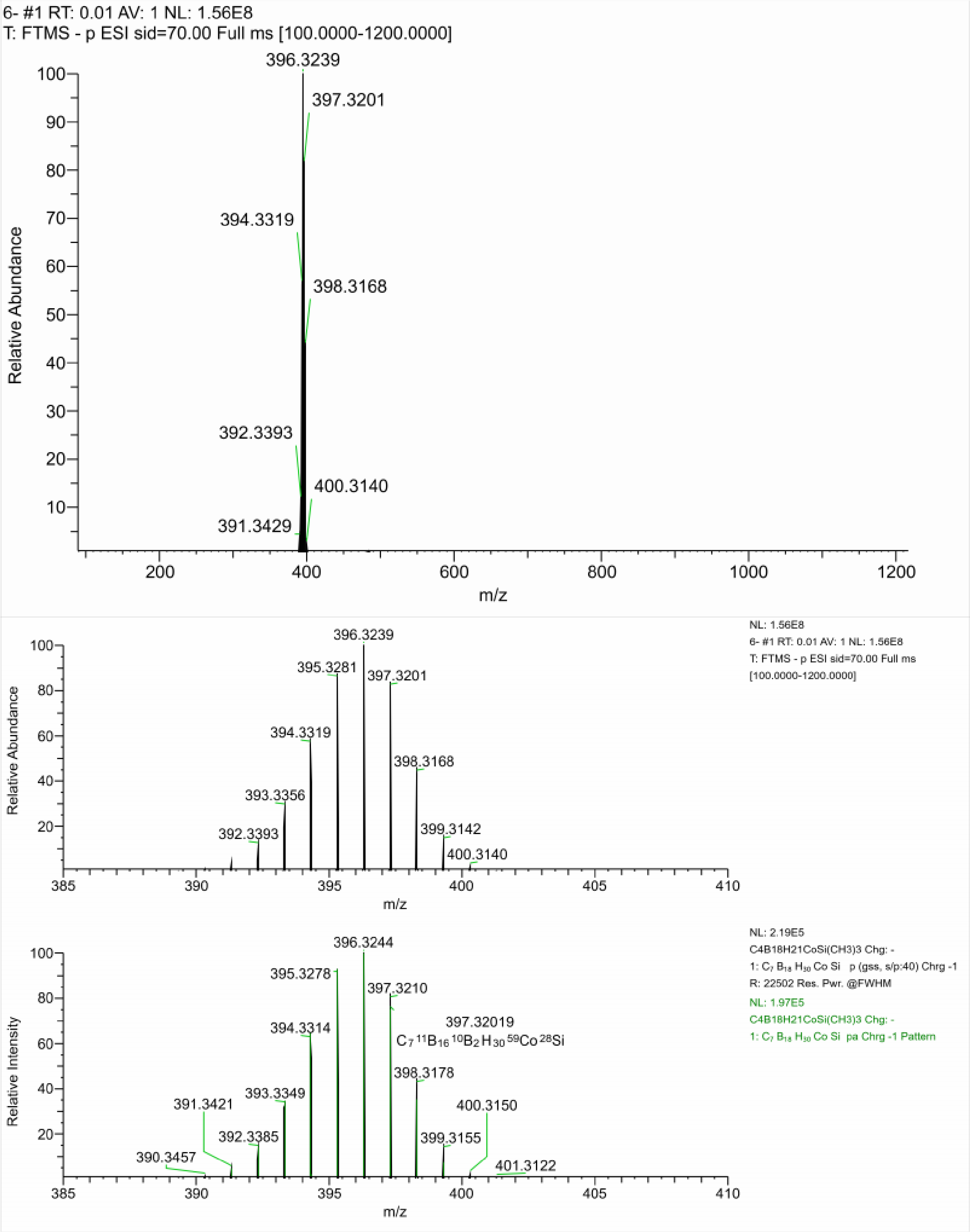

Figure S91. HRMS spectrum of **6**$^-$ with a calculated isotopic pattern.

HRMS Spectrum of $[(1,1'\text{-}Me_3Si\text{-}1,2\text{-}C_2B_9H_{10})_2\text{-}3,3'\text{-}Co(III)]^-$ (7^-)

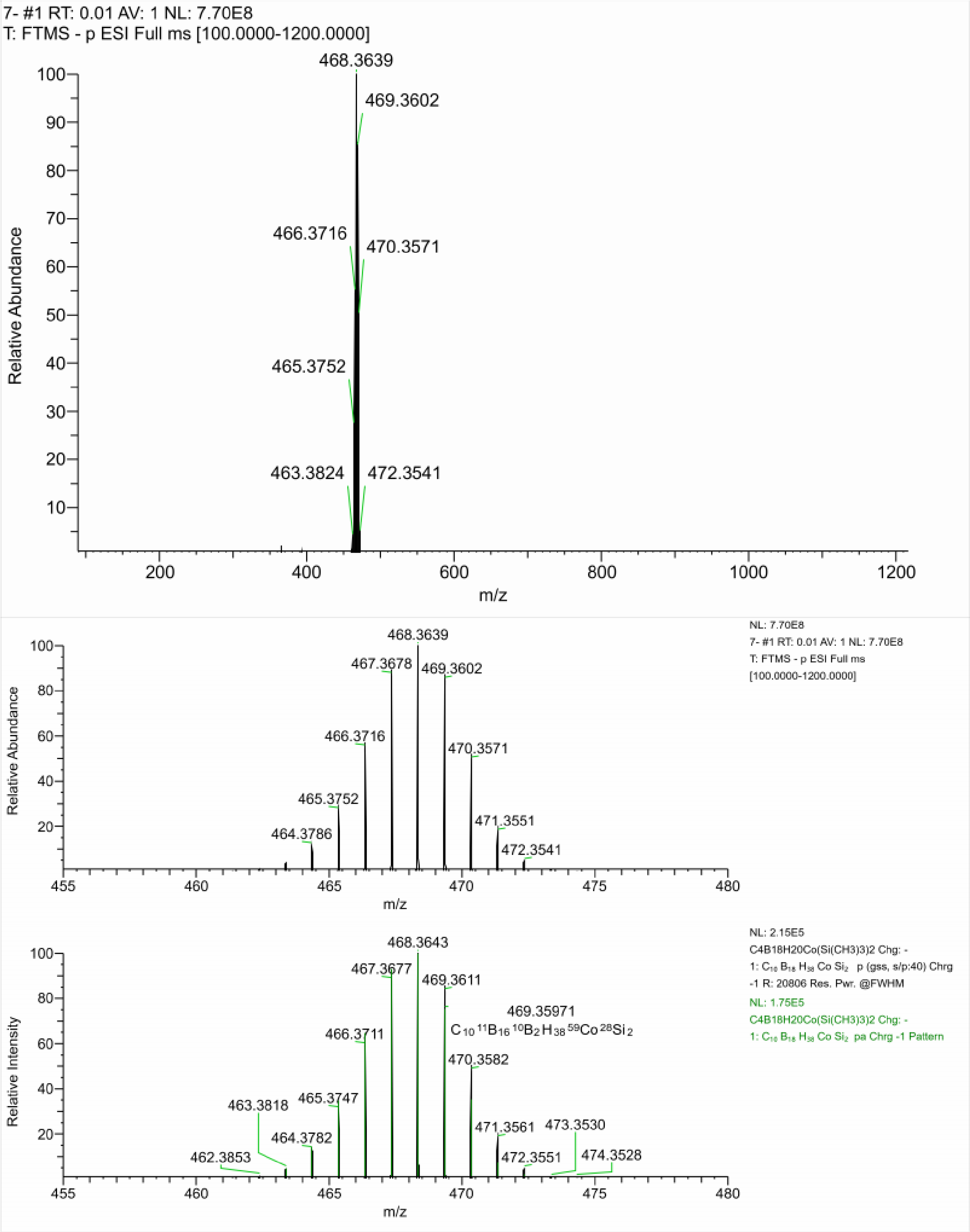

Figure S92. HRMS spectrum of 7^- with a calculated isotopic pattern.

HRMS Spectrum of $[(1\text{-}N_3\text{-}C_2H_4\text{-}1,2\text{-}C_2B_9H_{10})(1',2'\text{-}C_2B_9H_{11})\text{-}3,3'\text{-}Co(III)]^-$ (8^-)

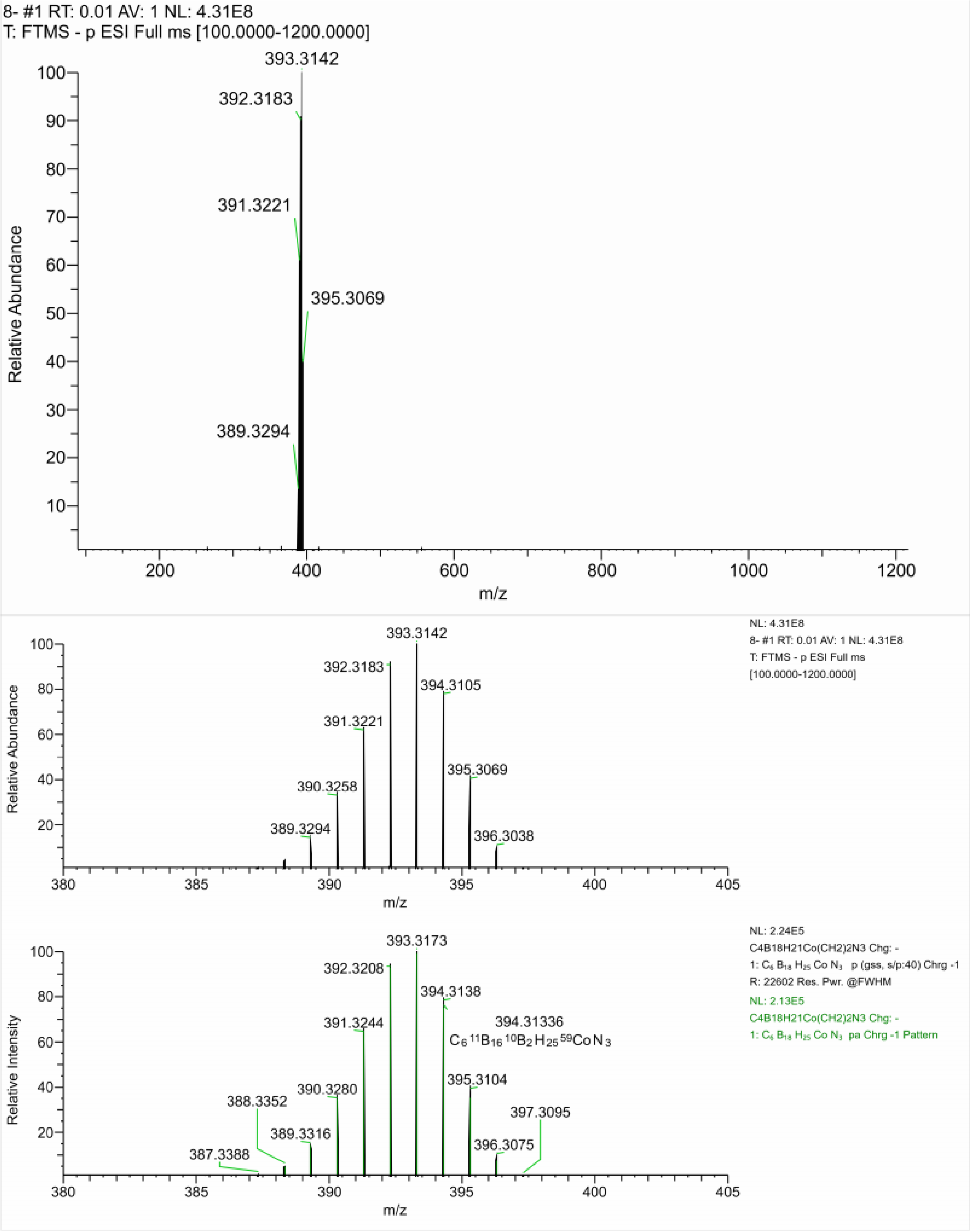

Figure S93. HRMS spectrum of **8**$^-$ with a calculated isotopic pattern.

HRMS Spectrum of $[(1\text{-}N_3\text{-}C_3H_6\text{-}1,2\text{-}C_2B_9H_{10})(1',2'\text{-}C_2B_9H_{11})\text{-}3,3'\text{-}Co(III)]^-$ (9^-)

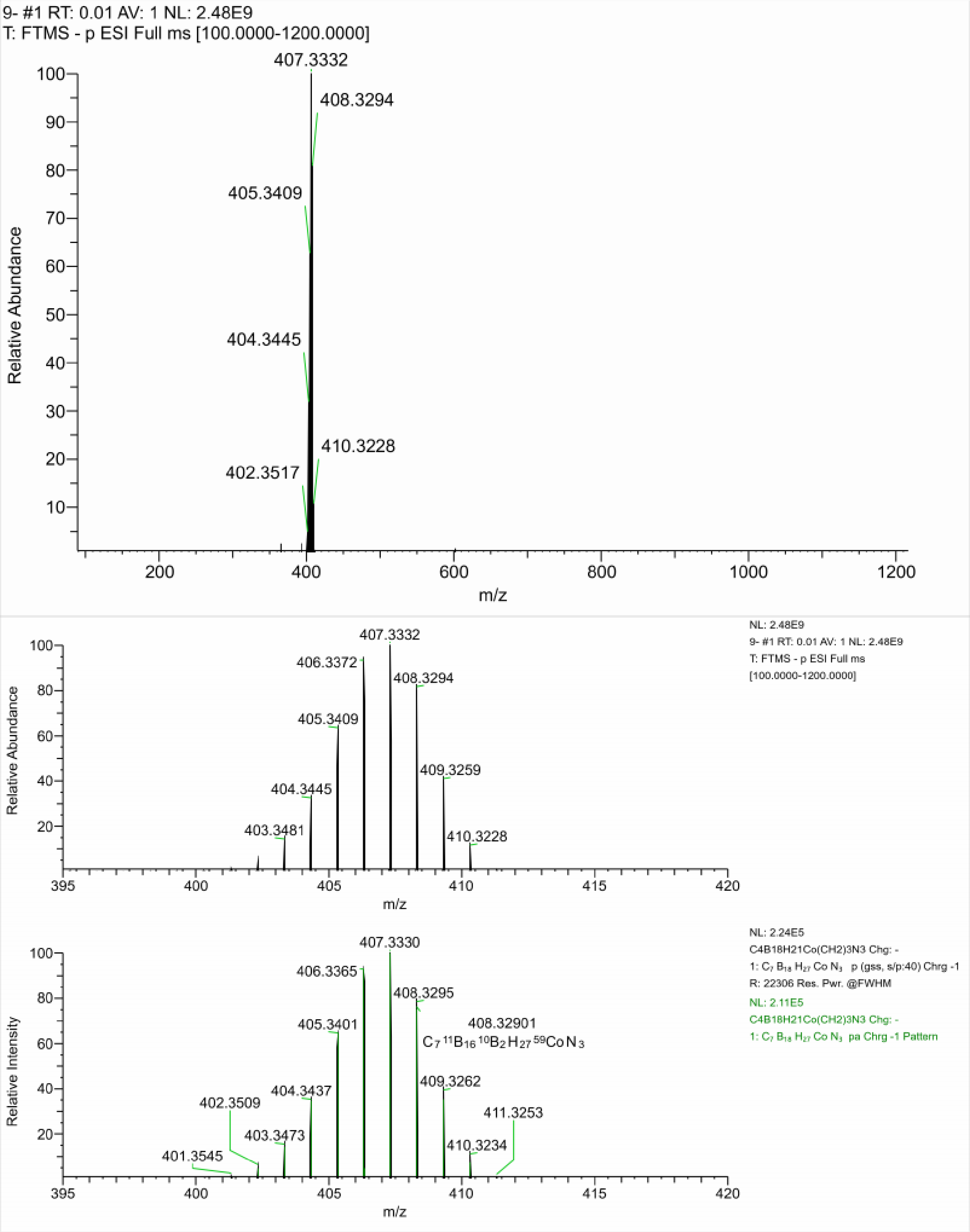

Figure S94. HRMS spectrum of **9**$^-$ with a calculated isotopic pattern.

HRMS Spectrum of [(1,1'-N_3-C_2H_4-1,2-$C_2B_9H_{10}$)$_2$-3,3'-Co(III)]$^-$ (10$^-$)

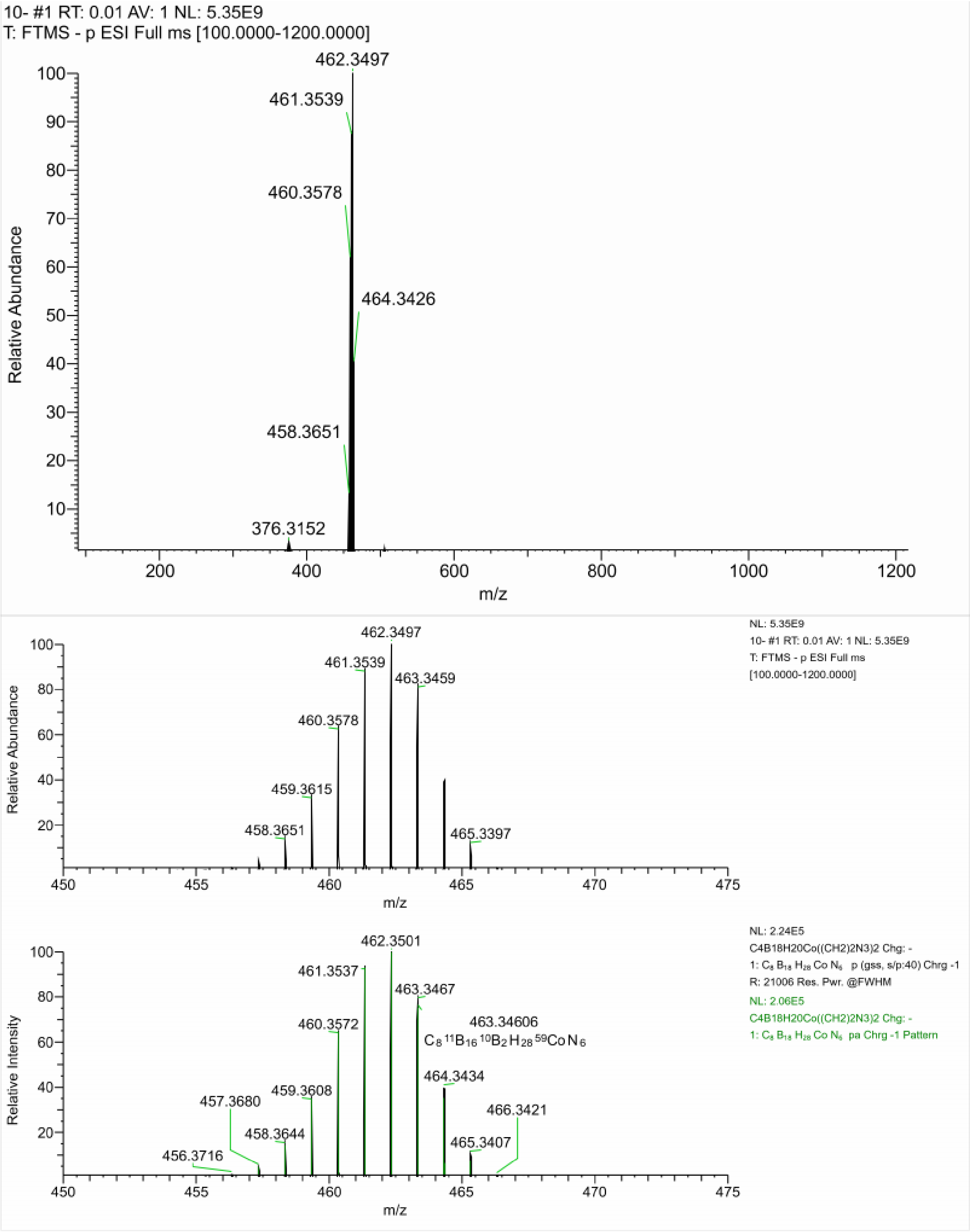

Figure S95. HRMS spectrum of **10**$^-$ with a calculated isotopic pattern.

HRMS Spectrum of [(1-(4-Ph-Triazolyl)-C_2H_4-1,2-$C_2B_9H_{10}$)(1',2'-$C_2B_9H_{11}$)-3,3'-Co(III)]$^-$ (11^-)

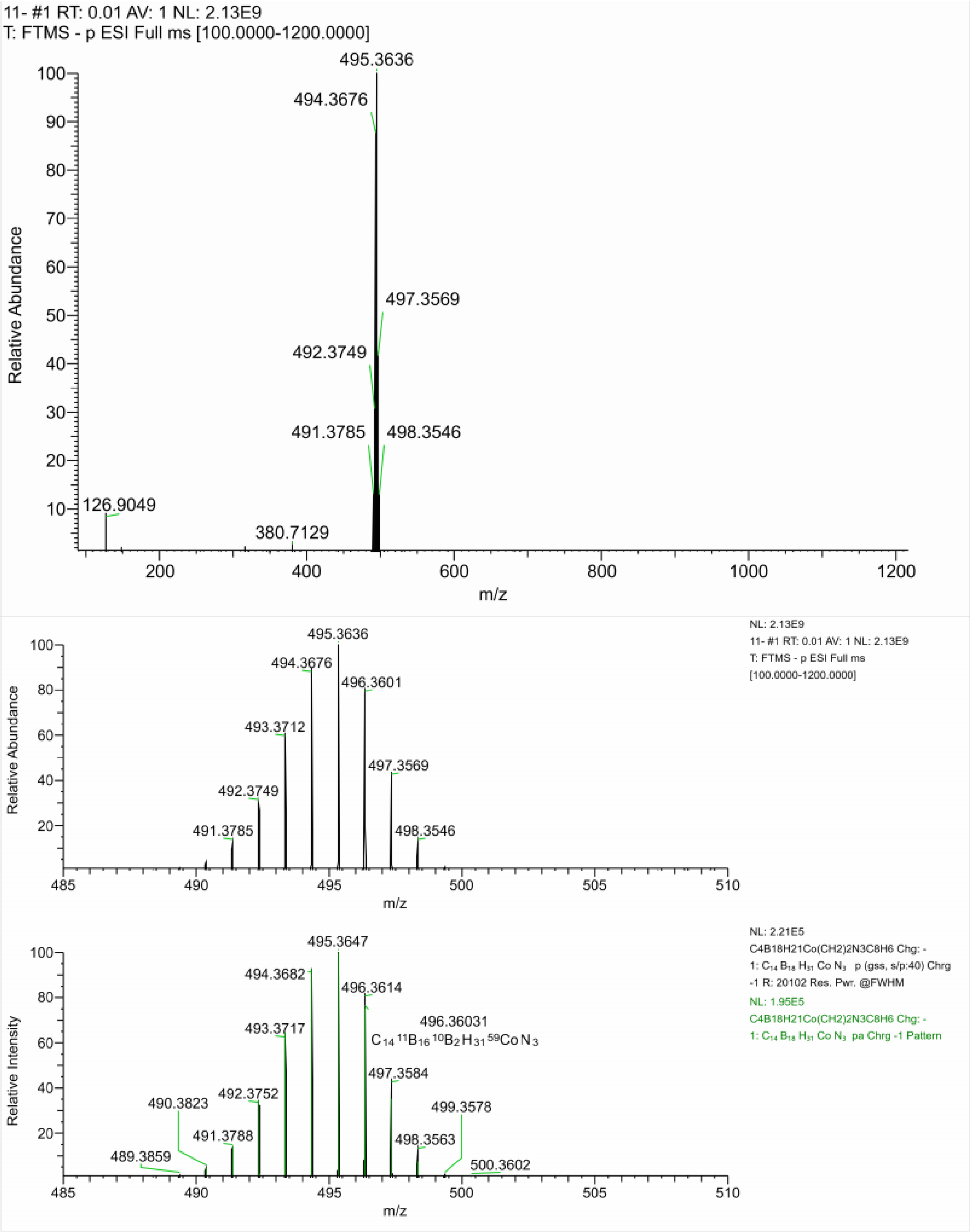

Figure S96. HRMS spectrum of **11**$^-$ with a calculated isotopic pattern.

HRMS Spectrum of [(1-(4-Ph-Triazolyl)-C_3H_6-1,2-$C_2B_9H_{10}$)(1',2'-$C_2B_9H_{11}$)-3,3'-Co(III)]$^-$ (12^-)

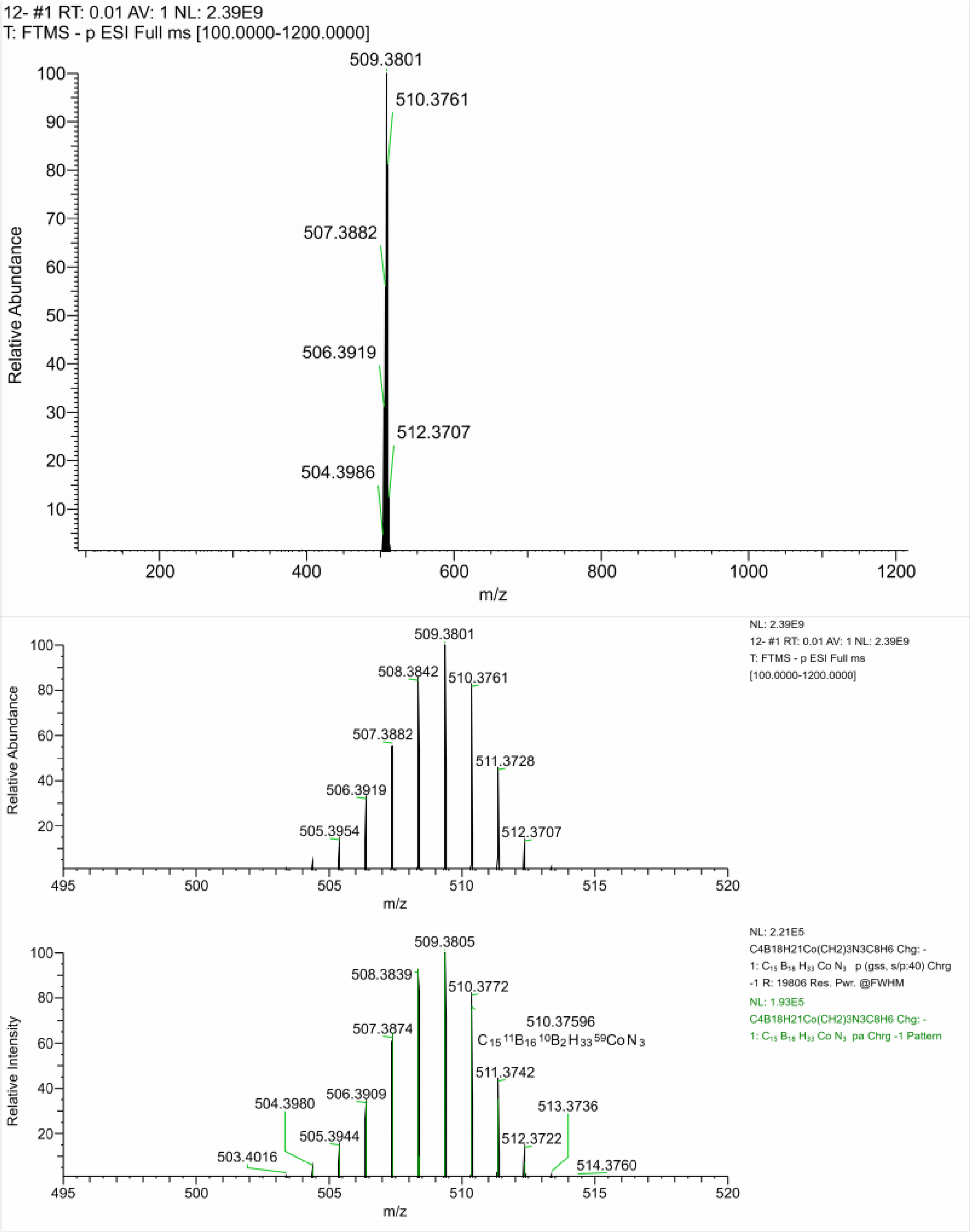

Figure S97. HRMS spectrum of **12**$^-$ with a calculated isotopic pattern.

HRMS Spectrum of $[(1\text{-}(CO)N_3\text{-}1,2\text{-}C_2B_9H_{10})(1',2'\text{-}C_2B_9H_{11})\text{-}3,3'\text{-}Co(III)]^-$ (13^-)

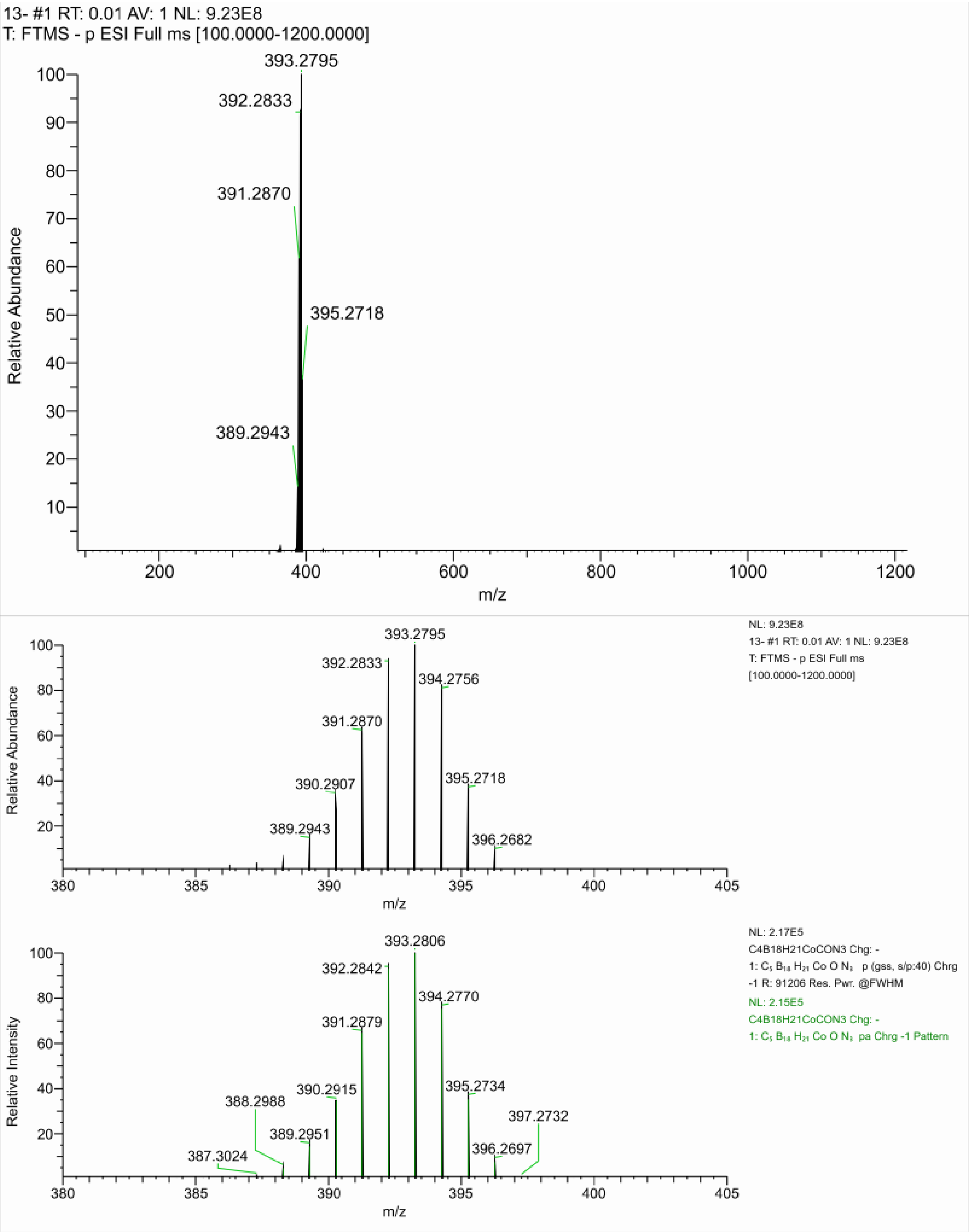

Figure S98. HRMS spectrum of **13**$^-$ with a calculated isotopic pattern.

References

(1) *CrysAlisPRO*; 2022. (accessed 2022).
(2) Busing, W. R.; Levy, H. A. High-speed computation of the absorption correction for single-crystal diffraction measurements. *Acta Crystallogr.* **1957**, *10* (3), 180-182.
(3) Sheldrick, G. M. SHELXT - Integrated space-group and crystal-structure determination. *Acta Cryst. Sect. A* **2015**, *71* (1), 3-8. DOI: 10.1107/S2053273314026370.
(4) Sheldrick, G. M. Crystal structure refinement with SHELXL. *Acta Crystallogr., Sect. C: Cryst. Struct. Commun.* **2015**, *71* (1), 3-8. DOI: 10.1107/S2053229614024218.
(5) Dolomanov, O. V.; Bourhis, L. J.; Gildea, R. J.; Howard, J. A. K.; Puschmann, H. OLEX2: a complete structure solution, refinement and analysis program. *J. Appl. Crystallogr.* **2009**, *42* (2), 339-341. DOI: doi:10.1107/S0021889808042726.
(6) *Diamond—Crystal and Molecular Structure Visualization Crystal Impact*; 2020. http://www.Crystalimpact.Com/Diamond (accessed March 2, 2023).
(7) Sicha, V.; Plesek, J.; Kvicalova, M.; Cisarova, I.; Gruner, B. Boron(8) substituted nitrilium and ammonium derivatives, versatile cobalt bis(1,2-dicarbollide) building blocks for synthetic purposes. *Dalton Trans.* **2009**, (5), 851-860, Article. DOI: 10.1039/b814941k. Sivaev, I. B.; Starikova, Z. A.; Sjoberg, S.; Bregadze, V. I. Synthesis of functional derivatives of the 3,3 '-Co(1,2-C2B2H11)(2) (-) anion. *Journal of Organometallic Chemistry* **2002**, *649* (1-2), 1-8, Article. DOI: 10.1016/s0022-328x(01)01352-3.
(8) Nekvinda, J.; Svehla, J.; Cisarova, I.; Gruner, B. Chemistry of cobalt bis(1,2-dicarbollide) ion; the synthesis of carbon substituted alkylamino derivatives from hydroxyalkyl derivatives via methylsulfonyl or p-toluenesulfonyl esters. *Journal of Organometallic Chemistry* **2015**, *798*, 112-120, Article. DOI: 10.1016/j.jorganchem.2015.06.032.
